# Synthesis and Elaboration of Medium-Sized Ring Building Blocks Prepared via Cascade Ring Expansion Reactions

**DOI:** 10.1021/acs.joc.5c00202

**Published:** 2025-04-01

**Authors:** Haimei Zhou, Peter O’Brien, William P. Unsworth

**Affiliations:** † University of York, Department of Chemistry, Heslington, York YO10 5DD, U.K.

## Abstract

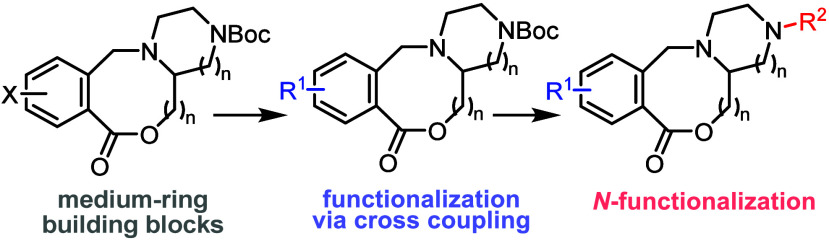

A general approach is described for the synthesis and elaboration of medium-sized ring mono- and difunctionalized 8- or 9-membered ring lactone building blocks. The lactones are prepared via cascade ring expansion reactions and elaborated via Suzuki–Miyaura cross coupling and various *N*-functionalization reactions. This enables efficient access to diverse, medium-sized ring building blocks in a synthetically challenging and under-represented area of the pharmaceutical chemical space.

The quality and diversity of compound libraries, building blocks and scaffolds are a cornerstone of modern drug discovery.^1^ In the building block space, chemists at AstraZeneca^2^ and Pfizer^3^ have carried out surveys of their own collections with a view to enhancing the building blocks used in their programs. Examples of both monofunctionalized (**1a**–**c**) and difunctionalized (**1d**) building blocks, with commonly encountered aryl halide/boronate or amine functionality, are shown in Scheme 1a. Previous work in one of our groups led to the development of a difunctionalized 3D building block **1e** (Scheme 1a) based on a normorphan scaffold and comprising a protected lactam and vinyl BMIDA cross-coupling handle.^4^ More recently, a detailed survey of the chemical space presented by commercially available building blocks has been carried out.^5^ From that study, it is clear that there are several factors that can influence the design of new building blocks. These include ensuring that the building blocks have properties that will not adversely affect the ADME-Tox profile of the potential drug candidate. In addition, the introduction of novel building blocks will allow the exploration of an underrepresented area of pharmaceutical space.

**Scheme 1:**
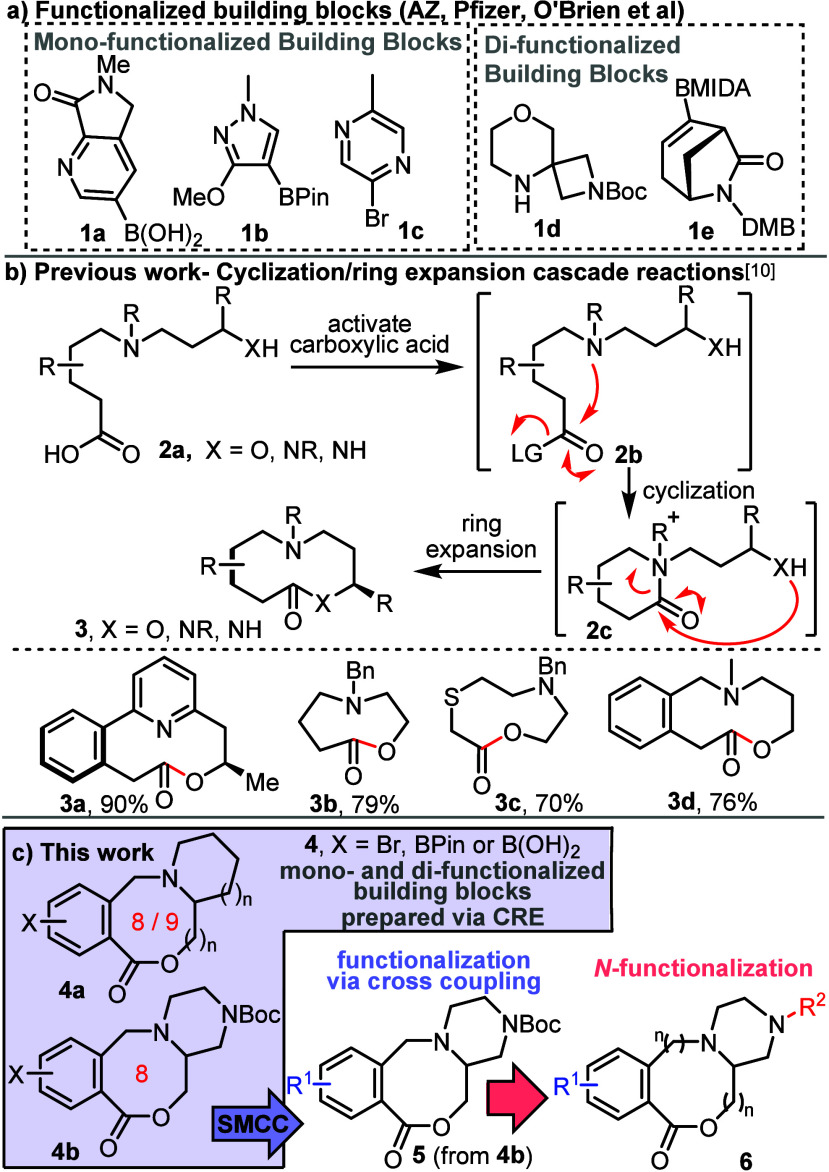
Synthesis and Elaboration of Medium-Sized Ring Lactone Building Blocks Prepared via Cascade Ring Expansion Reactions

Medium-sized rings (8–11-membered rings) are a highly important compound class in medicinal chemistry.^6^ However, compared to analogous normal-sized (5–7-membered) ring scaffolds, they are far less well explored, both in compound screening collections and as building blocks. In large part, this is due to the challenge of synthesizing medium-sized rings via cyclization reactions.^6b,7^ Ring expansion reactions are of much current interest in this context, as they represent a practical way to generate medium-sized ring products, without having to resort to high-dilution reaction conditions.^8,9^

The cyclization/ring expansion (CRE) cascade reaction method recently developed in one of our groups is one such approach (Scheme 1b).^10^ Using CRE, linear carboxylic acids of the form **2a** undergo overall end-to-end cyclization via a cascade reaction involving activation (**2a** → **2b**), cyclization via an internal tertiary amine group to form a charge reactive intermediate (**2b** → **2c**), and ring expansion in situ (**2c** → **3**). The CRE method has been used to form various lactam and lactone products (e.g., **3a–d**) in high yields, at 0.1 M concentration.

In this Note, we report the design, synthesis, and functionalization of medium-ring mono- and difunctionalized building blocks **4** (Scheme 1c). The monofunctionalized building blocks **4a** are 8- or 9-membered ring lactones and are designed as capping compounds with aryl halide or boronate cross-coupling synthetic handles. We also present two examples of 8-membered ring difunctionalized building blocks **4b** (X = Br or BPin) which are scaffold-like building blocks suitable for double functionalization. It was envisaged that, using Suzuki–Miyaura cross coupling (SMCC), building blocks **4** could be monoderivatized to give **5**. Then, after Boc group removal, functionalization occurred a second time to deliver 3-D, medium-ring lead-like compounds **6**, via a range of *N*-functionalizations commonly used in medicinal chemistry^11^ (sulfonylation, amidation, *N*-alkylation, reductive amination, Buchwald-Hartwig amination and S_N_Ar *N*-arylation). Herein, we present the successful realization of this approach, culminating in the development of novel medium-ring mono- and difunctionalized 8- and 9-membered ring lactone building blocks.

We started by preparing monofunctionalized medium-sized ring building blocks **4a–h**, with each substrate bearing a reactive handle able to undergo SMCC.^12^ The building block synthesis started with *N*-alkylation of a suitable amino alcohol (e.g., 2-piperidinemethanol) to form linear substrates **8**, with the requisite alkyl bromides **7** (obtained via Wohl–Ziegler bromination using *N-*bromosuccinimide, see SI for details).^13^ Conversion into medium-sized ring lactones was then accomplished by ester hydrolysis, followed by our standard CRE cascade method,^10^ activating the carboxylic acid using T3P.^10b,14^ This sequence, shown in Scheme 2a, enabled the synthesis of 8-membered aryl bromide-containing lactones **4a–d**, with the same sequence also used to prepare homologues **4e** and **4f** (see SI for full details). In the case of 9-membered ring lactone **4f**, its conversion into boronic acid **4g** and boronic ester **4h** was also demonstrated; these alternative building blocks were made as they were also expected to be amenable to SMCC, but with organohalide couplings partners, which tend to be more easily available than the analogous boronic acids/esters. As a simple demonstration of the elaboration with the aryl bromide building blocks, each of aryl bromides **4a–f** was cross-coupled with PhB(OH)_2_ in an SMCC reaction catalyzed by Pd(dppf)Cl_2_·CH_2_Cl_2_ (Scheme 2b).^15^

**Scheme 2:**
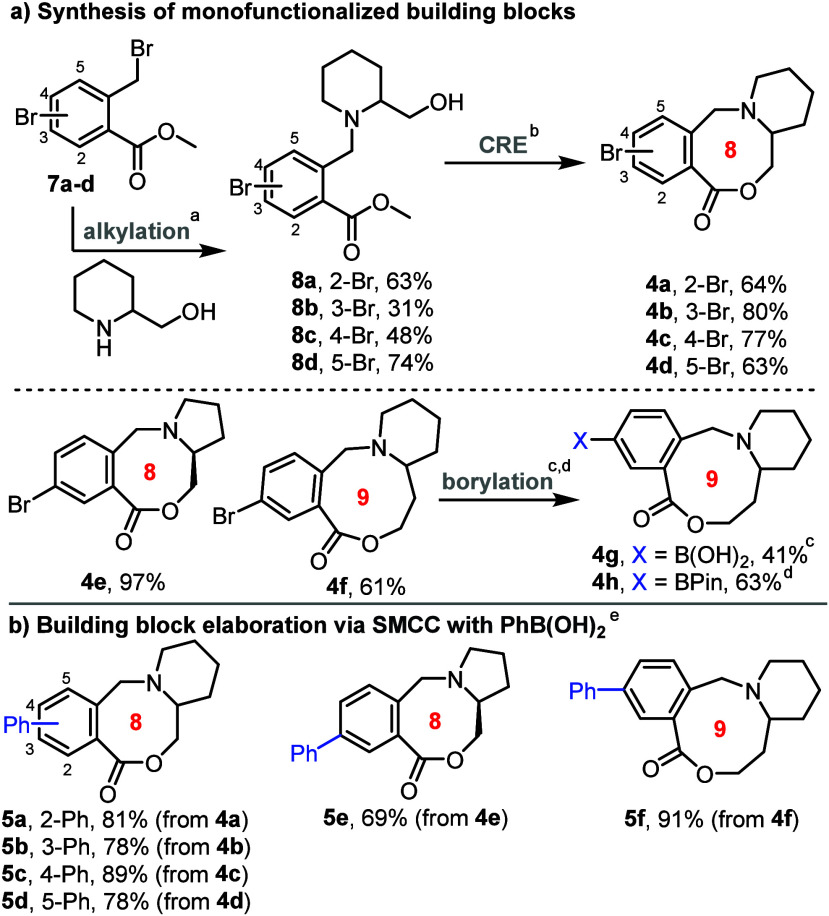
Synthesis and Elaboration of Monofunctionalized Building Blocks Reaction conditions (for full details see SI). **7** (1 equiv), amine (1 equiv), K_2_CO_3_, MeCN, 90 °C, 16–20 h **4** (1 equiv), MeOH, aq. LiOH, 50 °C, 1 h, then CHCl_3_, *i*-Pr_2_NEt, T3P, RT, 18 h **4f** (1 equiv), B_2_Pin_2_ (2 equiv), aq. Na_2_CO_3_, Pd(dppf)Cl_2_·CH_2_Cl_2_ (5 mol %), 1,4-dioxane, 50 °C, 16.5 h **4f** (1 equiv), B_2_Pin_2_ (2.3 equiv), Pd(dppf)Cl_2_ (10 mol %), KOAc, 1,4-dioxane, 60 °C, 23 h **4** (1 equiv) PhB(OH)_2_ (2 equiv), 1,4-dioxane, aq. Na_2_CO_3_, Pd(dppf)Cl_2_·CH_2_Cl_2_ (5 mol %), 50 °C, 16–20 h.

Two difunctionalized lactone building blocks **4i** and **4j** (Scheme 3) were also synthesized using the same methods described earlier in Scheme 2a. Both difunctionalized building blocks were designed to contain a Boc-protected amine, to enable *N*-functionalization following Boc group removal, and either an aryl bromide (**4i**) or aryl boronic ester (**4j**) cross-coupling handle, suitable for deployment in SMCC reactions. Each building block was elaborated at both positions via the installation of a range of medicinally relevant functional groups.

**Scheme 3:**
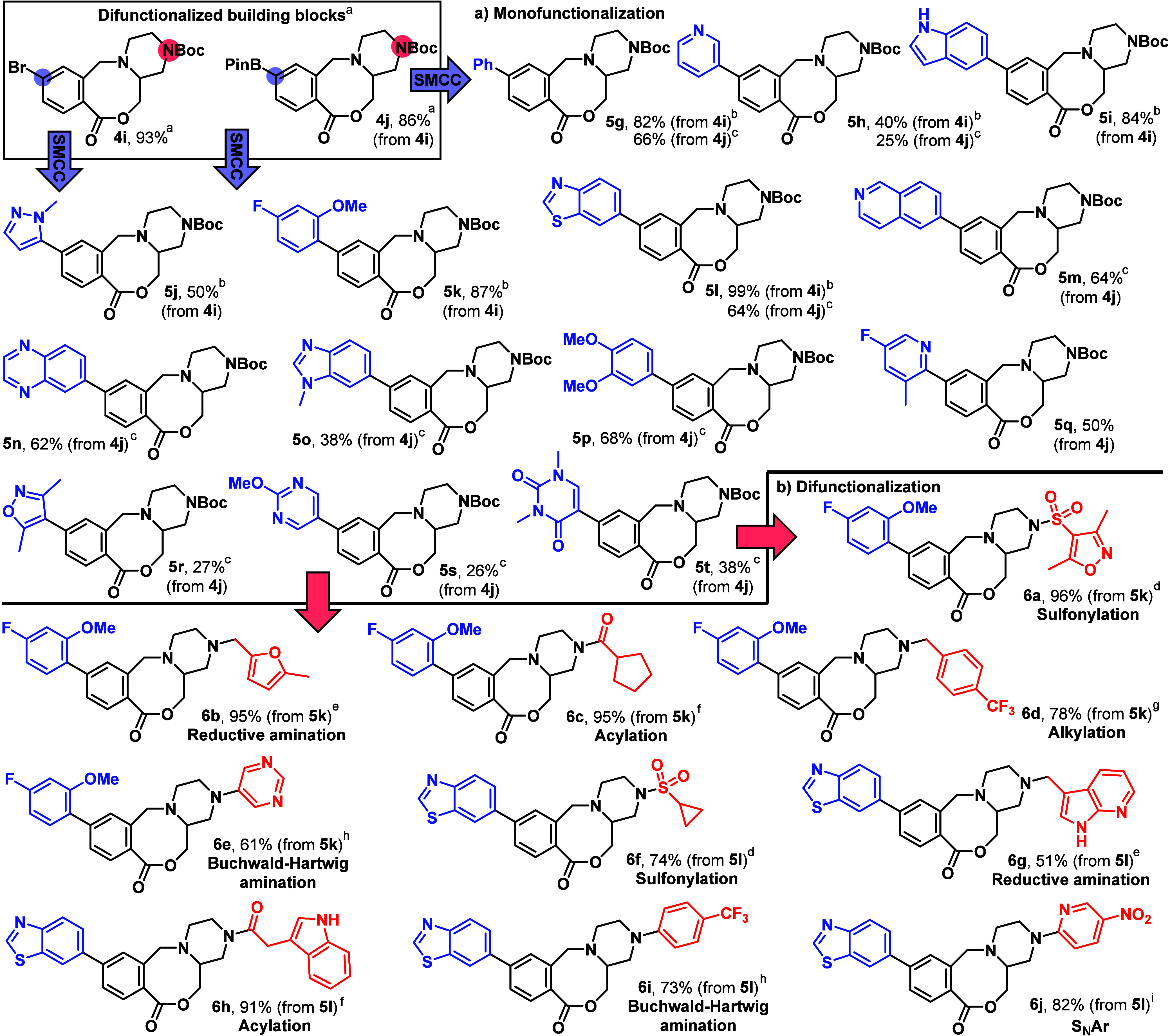
Synthesis and Elaboration of Difunctionalized Building Blocks Reaction conditions (for full details see SI). Prepared on gram scale using the methods summarized in Scheme 2a (see SI for full details). **4i** (1 equiv), ArB(OH)_2_ or ArBPin (1.5–2 equiv), aq. Na_2_CO_3_, Pd(dppf)Cl_2_·CH_2_Cl_2_ (5 mol %), 1,4-dioxane, 50 °C, 16–20 h. **4j** (1 equiv), ArBr (2 equiv), aq. Na_2_CO_3_, Pd(dppf)Cl_2_·CH_2_Cl_2_ (10 mol %), 1,4-dioxane, 50 °C, 16–20 h. **5k/l** (1 equiv), 4 M HCl in 1,4-dioxane, then CH_2_Cl_2_, NEt_3_, sulfonyl chloride (1.2 equiv), DMAP, 0 °C → RT, 18 h. **5k/l** (1 equiv), 4 M HCl in 1,4-dioxane, then THF, aldehyde (1 equiv), AcOH, NaBH(OAc)_3_, RT, 18 h. **5k/l** (1 equiv), 4 M HCl in 1,4-dioxane, then CH_2_Cl_2_, NEt_3_, acid chloride (1.2 equiv), DMAP, 0 °C → RT, 18 h. **5k** (1 equiv), 4 M HCl in 1,4-dioxane, then THF, NEt_3_, 1-(bromomethyl)-4-(trifluoromethyl) benzene (1.2 equiv), 70 °C, 18 h. **5k/l** (1 equiv), 4 M HCl in 1,4-dioxane, then toluene, Cs_2_CO_3_, (±)-BINAP, Pd_2_bda_3_ (10 mol %), ArBr (1.2 equiv), 110 °C, 65 h. **6l** (1 equiv), 4 M HCl in 1,4-dioxane, then CH_3_CN, K_2_CO_3_, 2-chloro-5-nitropyridine (1.2 equiv), 70 °C, 18 h.

First, the SMCC of bromide **4i** with different aryl boronic acids/esters was explored. These cross couplings worked well, affording coupled products **5g–l** in generally good yields. Pleasingly, good yields were obtained with a pyridine, an unprotected indole, and a *N*-methylpyrazole. Next, the range of functionalized medium-ring lactones accessible was expanded further by reacting boronic ester containing building block **4j** with a range of readily available aryl bromides, to form coupled products **5g**, **5h**, and **5l**–**t** (Scheme 3a). This cross-coupling manifold was generally lower yielding, as shown by a comparison of the yields obtained for **5g**, **5h** and **5l**. Nevertheless, using the boronic ester building block **4j**, and noting that no individual reaction optimization was attempted, a range of challenging cross-couplings was accomplished in 26–66% yields using just one set of reaction conditions. A range of heteroaryl groups was successfully incorporated, including isoquinoline, quinoxaline, benzimidazole, pyridine, isoxazole, 2-methoxypyrimidine and a uracil derivative.

Representative monofunctionalized products **5k** and **5l** were then elaborated a second time via a range of *N*-functionalization reactions, with six different reaction classes demonstrated in total (Scheme 3b). In all cases, cleavage of the Boc protecting group was carried out by reaction with 4 M HCl in 1,4-dioxane; this was followed by concentration to form the HCl salt, which was used directly in the following *N*-functionalization reaction without purification. Sulfonylation worked well under standard conditions,^16^ with sulfonamides **6a** and **6f** isolated in high yields from substrates **5k** and **5l** respectively. Reductive amination^17^ also worked well, to afford amines **6b** and azaindole derivative **6g**, as did acylation using acid chlorides^18^ to form amides **6c** and **6h**. Alkylation of the amine^19^ with 1-(bromomethyl)-4-(trifluoromethyl) benzene afforded amine **6d**. Both building blocks were also amenable to Buchwald–Hartwig amination,^20^ exemplified by the formation of functionalized aniline **6e** and **6i** in good yields. Finally, substrate **5l** was converted into functionalized aniline **6j** following a high yielding S_N_Ar reaction.^21^

In summary, we report a general approach for the synthesis and elaboration of medium-ring mono- and difunctionalized 8- or 9-membered ring lactone building blocks. Both types of building blocks have been elaborated using medicinally relevant coupling or *N*-functionlization partners. The modular approach of the building block synthesis means that other medium-ring building blocks can be readily prepared. Ten examples of difunctionalized lead-like compounds are prepared to showcase this medium-ring building approach to scaffolds decorated with medicinal chemistry-like functionality in an underrepresented area of pharmaceutical chemical space.

## Data Availability

The data underlying this study are available in the published article and its Supporting Information.

## Supporting Information Available

Experimental procedures, characterization data and copies of ^1^H and ^13^C NMR spectra for all novel compounds featured in this manuscript. (PDF)
