# Supplementary material for: Synthesis and Elaboration of Medium-Sized Ring Building Blocks Prepared via Cascade Ring Expansion Reactions
Source: J Org Chem. 2025 Apr 1;90(14):5070–4. doi: 10.1021/acs.joc.5c00202 (PMC11998084; doi:10.1021/acs.joc.5c00202)
Supplement: Supplementary file 1 — jo5c00202_si_001.pdf [file jo5c00202_si_001.pdf]

Supporting Information

Synthesis and elaboration of medium-sized ring building blocks pre-pared via cascade ring expansion reactions.

Haimei Zhou, Peter O’Brien* and William P. Unsworth*

*william.unsworth@york.ac.uk; peter.obrien@york.ac.uk

Address for all authors:
University of York, Department of Chemistry, Heslington, York YO10 5DD (UK).

Contents

1.	General Information	S3–5
2.	Compound Characterisation Data and Procedures	S6–45
3.	^{1}H and ^{13}C{^{1}H} NMR Spectra	S43–97
4.	References	S98

1) General Information

Unless otherwise stated, all reactions were carried out at RT under an inert (N_2 or Ar) atmosphere in oven-dried glassware. Except where stated all reagents were purchased from commercial sources: Merck (Sigma Aldrich), Alfa Aesar, Acros Organics, Fisher Chemicals, VWR, TCI, Across chemicals and Fluorochem and were used without further purification. Anhydrous CH_2Cl_2, toluene and MeCN were obtained from an Innovative Technology Inc. PureSolv® solvent purification system. Dry THF was obtained from the SPS laboratory system and used immediately after being dispensed. Dry Et_3N and DIPEA obtained by drying with CaH_2 and then distilling and storing over KOH or 3 Å molecular sieves, under Ar. Anhydrous 1,4-dioxane was purchased from Sigma Aldrich and used as supplied.

^{1}H NMR spectra were recorded at 400 MHz on Bruker AV400 or Bruker AMX 400/JEOL ECS-400 and at 500 MHz on Bruker DRX500 MHz Ultra ShieldTM spectrometry. ^{13}C NMR spectra were recorded at 101 MHz on Bruker AV 400 or Bruker AMX 400 MHz Ultra ShiledTM and 126 MHz on Bruker DRX500 MHz Ultra ShiledTM spectrometry. ^{19}F NMR spectra were recorded at 376 MHz on Bruker AV400 or Bruker AMX 400/JEOL ECS-400 spectrometry. ^{31}P NMR spectra were recorded at 162 MHz on Bruker AV400 or Bruker AMX 400/JEOL ECS-400 spectrometry.

All spectral data was acquired at 298 K (25 °C) unless stated otherwise and samples were dissolved in $CDCl_3$ unless specified otherwise. Chemical shifts (δ) are reported in parts per million (ppm), with residual solvent peaks: $CDCl_3$: δ_H = 7.26, $CDCl_3$: δ_C = 77.0, $(CD_3)_2SO$: δ_H = 2.50, δ_C = 39.5, CD_3OD: δ_H = 3.31, δ_C = 49.0, being used for internal reference. The multiplicity abbreviations used are: s, singlet; d, doublet; t, triplet; q, quartet; p, pentet; m, multiplet; dd, doublet of doublets; dt doublet of triplets; td, triplet of doublets; tt, triplet of triplets; ddd, doublet of doublets of doublets; pd, pentet of doublets; where br indicates a broad signal, and app. indicates an apparent. ^{1}H experiments are reported as: chemical shift in ppm, quoted to the nearest 0.01 ppm, (integration, multiplicity, coupling constant and assignment (where possible)). ^{13}C experiments are reported as: chemical shift in ppm, quoted to the nearest 0.1 ppm, (carbon assignment (where possible) or multiplicity, coupling constant and assignment (where applicable)). ^{19}F experiments are reported as: chemical shift in ppm, quoted to the nearest 0.1 ppm, (multiplicity, coupling constant and assignment (where possible)). ^{31}P

experiments are reported as: chemical shift in ppm, quoted to the nearest 0.1 ppm, (multiplicity, and assignment (where possible)).

Spectra were analysed using MestReNova 12.0.3-21384 software and values of coupling constant (*J*) are reported in Hertz (Hz) to the nearest 0.5 Hz. The term "overlapping" is used to describe resonance peak, which is behind another resonance peak, *i.e.*, compound resonance behind the solvent peak or combination of two resonance peaks. The systematic chemical names were generated using the IUPAC name generator tool option is included within the ChemBioFDaw Ultra 19.1 software.

Infrared (IR) spectra were recorded on a PerkinElmer UATR 2 or Pekin Elmer Spectrum 100 spectrometer fitted with a universal Attenuated Total Reflectance (ATR) accessory; data was recorded as a thin film dispersed from either CH_2Cl_2 or $CDCl_3$, neat or solid state by ATR-FTIR. IR-recorded experiments are reported as: IR (method of recorded) ν_{max} (IR absorption maxima) / unit (cm^{-1}) chemical absorption (assignment (where possible)). The intensity of each absorbance band gives the annotated appearance, and each bond was described as w (weak), m (medium), s (strong), sh (sharp) and with the prefix v (very) and suffix br (broad).

High Resolution Mass Spectra (HRMS) were obtained by the University of York Mass Spectrometry Service, recorded on a Waters XEVO G2-XS TOF, Waters Synapt G2S TOF or Bruker Micro-TOF mass spectrometer, with HRMS mode incorporating a lock-in mass into the mobile phase (leucine enkehalin) or on a Bruker Daltonics, Micro-TOF spectrometer, using Electrospray Ionisation (ESI) or Atmospheric Pressure Chemical Ionisation (APCI), positive or negative generative modes.

Thin Layer Chromatography (TLC) was carried out on Merck silica gel $60F_{254}$ pre-coated aluminium foil sheets and was visualised using UV light (λ = 254 nm, short wavelength) or UV light (λ = 366 nm, long wavelength) and stained with basic aqueous potassium permanganate ($KMnO_4$), ninhydrin or vanillin solution dip. Concentration under reduced pressure or *vacuo* was performed using a Büchi® Rotavapor® R-210 evaporator with jack and water bath, 29/32 joint, 240V rotary evaporator using a mixture of acetone and dry ice or ice/water as the coolant. Flash column chromatography was conducted using Aldrich technical grade silica gel (SiO_2), 60 Å, 230-400 mesh, 40-63 µm particle size, under a light positive pressure of air,

eluting with the specified solvent system.

Melting points were recorded as decomposition temperature range and measured on a Stuart SMP10 or Gallenkamp apparatus using open tubes with no corrections. Before measuring the melting point, in most instances, the solids were purified by recrystallisation after purification by column chromatography, where "(from [solvent])" donating solvent systems were used, e.g. single or multiplet.

2) Compound Characterisation Data and Procedures

Methyl 2-bromo-6-(bromomethyl)benzoate (7a)

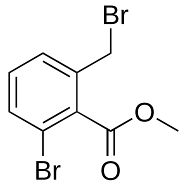

A round-bottomed flask was charged with benzene (20.0 mL), *N*-bromosuccinimide (855mg, 4.80 mmol), benzoyl peroxide (76.0 mg, 0.218 mmol) and methyl 2-bromo-6-methylbenzoate (1.00 g, 4.37 mmol). The solution was stirred and heated at 85°C for 22 hours under argon. Then, the round-bottomed flask was removed from the heating block, after being allowed to cool to RT, the mixture was filtered. The filtrate was transferred into a separating funnel and water (20 mL) was added, and extracted with dichloromethane (3 × 20 mL). The combined organic phases were dried with magnesium sulphate, filtered and concentrated in vacuo to give the crude product. The product was purified by column chromatography (SiO_2, 5:1 → 2:1 hexane : dichloromethane) to afford the title compound (1.01 g, 75%) as a colourless oil. R_f = 0.43 (2:1 hexane : dichloromethane); ν_{max}/cm^{-1} (thin film) 1729, 1444, 1277, 1103, 1059, 954, 773; δ_H (400 MHz, $CDCl_3$) 7.55 (dt, 1H, *J* = 8.0, 1.5 Hz), 7.39 (dt, 1H, *J* = 8.0, 1.5 Hz), 7.26 (td, 1H, *J* = 8.0, 1.5 Hz), 4.50 (s, 2H), 4.01 (s, 3H); $^{13}C\{^1H\}$ NMR (100 MHz, $CDCl_3$) 167.3, 137.2, 135.5, 133.0, 131.2, 129.2, 120.19, 52.9, 29.6 (Ar**C**H_2Br); HRMS (ESI) m/z: $[M + Na]^+$ calcd. for $C_9H_8{}^{79}Br_2NaO_2{}^+$, 328.8783. Found: 330.8753. Characterisation data matched those reported in the literature.[1]

Methyl 5-bromo-2-(bromomethyl)benzoate (7b)

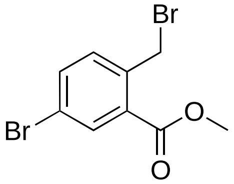

A round-bottomed flask was charged with benzene (80.0 mL), N-bromosuccinimide (4.27 g, 24.0 mmol), benzoyl peroxide (378 mg, 1.09 mmol) and methyl 5-bromo-2-methylbenzoate (5.00 g, 21.8 mmol). The solution was stirred and heated at 85 °C for 16 hours under argon. Then, the round-bottomed flask was removed from the heating block, after being allowed to cool to RT, the mixture was filtered. The filtrate was transferred into a separating funnel and water (50 mL) was added, and extracted with dichloromethane (3 × 50 mL). The combined organic phases were dried with magnesium sulphate, filtered and concentrated in vacuo to give the crude product. The product was purified by column chromatography (SiO_2, 2:1 → 1:1 hexane : dichloromethane) to afford the title compound (4.66 g, 69%) as a colourless oil. R_f = 0.44 (2:1 hexane : dichloromethane); ν_{max}/cm^{-1} (thin film) 1721, 1433, 1285, 1254, 1119, 1076, 967, 856, 829, 800; δ_H (400 MHz, $CDCl_3$) 8.11 (d, 1H, *J* = 2.0 Hz), 7.62 (dd, 1H, *J* = 8.0, 2.0 Hz), 7.34 (d, 1H, *J* = 8.0 Hz), 4.90 (s, 2H), 3.95 (s, 3H); $^{13}C\{^1H\}$ NMR (100 MHz, $CDCl_3$) 165.8, 138.4, 135.6, 134.3, 133.3, 130.7, 122.5, 52.7, 30.6 (Ar**C**H_2Br); HRMS (ESI) m/z: $[M + Na]^+$ calcd. for

$C_9H_8{}^{79}Br_2NaO_2{}^+$, 328.8783. Found: 330.8767. Characterisation data matched those reported in the literature.[2]

Methyl 4-bromo-2-(bromomethyl)benzoate (7c)

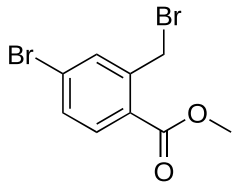

A round-bottomed flask was charged with benzene (60.0 mL), *N*-bromosuccinimide (2.56 g, 14.4 mmol), benzoyl peroxide (227 mg, 0.655 mmol) and methyl 4-bromo-2-methylbenzoate (3.00 g, 13.1 mmol). The solution was stirred and heated at 85 °C for 23 hours under argon. Then, the round-bottomed flask was removed from the heating block, after being allowed to cool to RT, the mixture was filtered. The filtrate was transferred into a separating funnel and water (60 mL) was added, and extracted with dichloromethane (3 × 60 mL). The combined organic phases were dried with magnesium sulphate, filtered and concentrated in vacuo to give the crude product. The product was purified by column chromatography (SiO_2, 5:1 → 2:1 hexane : dichloromethane) to afford the title compound (3.15 g, 78%) as a colourless oil. R_f = 0.56 (2:1 hexane : dichloromethane); ν_{max}/cm^{-1} (thin film) 1717, 1587, 1433, 1259, 1118, 1093, 868, 779, 704; δ_H (400 MHz, $CDCl_3$) 7.84 (d, 1H, *J* = 8.5 Hz), 7.63 (d, 1H, *J* = 2.0 Hz), 7.51 (dd, 1H, *J* = 8.5, 2.0 Hz), 4.89 (s, 2H), 3.94 (s, 3H); $^{13}C\{^1H\}$ NMR (100 MHz, $CDCl_3$) 166.3, 141.3, 134.7, 132.9, 131.8, 127.8, 127.2, 52.6, 30.4. Characterisation data matched those reported in the literature.[3]

Methyl 3-bromo-2-(bromomethyl)benzoate (7d)

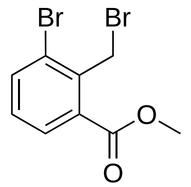

A round-bottomed flask was charged with benzene (20.0 mL), *N*-bromosuccinimide (855mg, 4.80 mmol), benzoyl peroxide (76.0 mg, 0.218 mmol) and methyl 3-bromo-2-methylbenzoate (1.00 g, 4.37 mmol). The solution was stirred and heated at 85 °C for 16.5 hours under argon. Then, the round-bottomed flask was removed from the heating block, after being allowed to cool to RT, the mixture was filtered. The filtrate was transferred into a separating funnel and water (20 mL) was added, and extracted with dichloromethane (3 × 20 mL). The combined organic phases were dried with magnesium sulphate, filtered and concentrated in vacuo to give the crude product. The product was purified by column chromatography (SiO_2, 5:1 → 2:1 hexane : dichloromethane) to afford the title compound (1.22 g, 91%) as a colourless oil. R_f = 0.32 (3:1 hexane : dichloromethane); ν_{max}/cm^{-1} (thin film) 1721, 1434, 1259, 1222, 1113, 970, 759, 705; δ_H (400 MHz, $CDCl_3$) 7.89 (dd, 1H, *J* = 8.0, 1.5 Hz), 7.76 (dd, 1H, *J* = 8.0, 1.5 Hz), 7.23 (t, 1H, *J* = 8.0 Hz), 5.13 (s, 2H), 3.95 (s, 3H); $^{13}C\{^1H\}$ NMR (100 MHz, $CDCl_3$) 166.6, 138.0, 137.2, 131.7, 130.5, 129.6, 127.2, 52.8, 30.2; HRMS (ESI) m/z: $[M + Na]^+$ calcd. for $C_9H_8{}^{79}Br_2NaO_2{}^+$, 328.8783; Found: 330.8729. Characterisation data matched those reported in the literature.[3]

Methyl 2-bromo-6-((2-(hydroxymethyl)piperidin-1-yl)methyl)benzoate (8a)

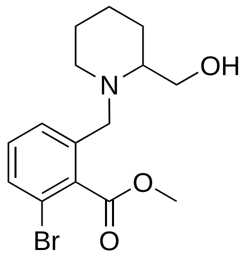

To a stirring solution of potassium carbonate (1.21 g, 8.77 mmol) in acetonitrile (20.0 mL), 2-piperidinemethanol (337 mg, 2.92 mmol) was added followed by methyl 2-bromo-6-(bromomethyl)benzoate **7a** (900 mg, 2.92 mmol). The reaction mixture was refluxed at 90 °C for 17 hours under argon. Then the reaction mixture was allowed to cool to RT, filtered and washed with dichloromethane, concentrated under vacuum and purified by column chromatography (SiO_2, 3:1 → 1:1 hexane : ethyl acetate) to afford the title compound (634 mg, 63%) as a yellow oil. R_f = 0.68 (1:1 hexane : ethyl acetate); ν_{max}/cm^{-1} (thin film) 2936, 1732, 1718, 1446, 1278, 1150, 1107, 1063; δ_H (400 MHz, $CDCl_3$) 7.46 (dd, 1H, *J* = 6.5, 3.0 Hz), 7.22 – 7.11 (m, 2H), 4.30 (d, 1H, *J* = 13.5 Hz), 3.94 (s, 3H), 3.91 (dd, 1H, *J* = 12.0, 3.0 Hz), 3.40 (dd, 1H, *J* = 12.0, 3.0 Hz), 3.09 (d, 1H, *J* = 13.5 Hz), 2.69 (dt, 1H, *J* = 11.5, 3.5 Hz), 2.17 (dq, 1H, *J* = 10.0, 3.5 Hz), 1.91 (td, 1H, *J* = 11.5, 3.5 Hz), 1.75 – 1.58 (m, 3H, C**H₂**), 1.53 – 1.45 (m, 1H), 1.37 – 1.23 (m, 2H); $^{13}C\{^1H\}$ NMR (100 MHz, $CDCl_3$) 169.9, 140.0, 134.7, 131.7, 130.6, 128.4, 120.3, 63.3 (N**C**H), 62.8, 56.9, 52.7, 52.6, 27.7, 24.7, 23.9; HRMS (ESI) m/z: $[M + H]^+$ calcd. for $C_{15}H_{21}{}^{79}BrNO_3^+$, 342.0699; Found: 344.0676.

Methyl 5-bromo-2-((2-(hydroxymethyl)piperidin-1-yl)methyl)benzoate (8b)

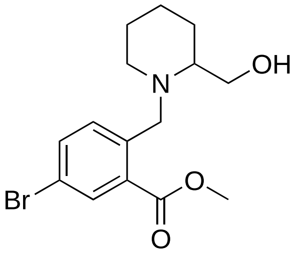

To a stirring solution of potassium carbonate (1.21 g, 8.77 mmol) in acetonitrile (20.0 mL), 2-piperidinemethanol (337 mg, 2.92 mmol) was added followed by methyl 5-bromo-2-(bromomethyl)benzoate **7b** (900 mg, 2.92 mmol). The reaction mixture was refluxed at 90 °C for 19 hours under argon. Then the reaction mixture was allowed to cool to RT, filtered and washed with dichloromethane, concentrated under vacuum and purified by column chromatography (SiO_2, 90:9:1 → 75:24:1 dichloromethane : acetone : triethylamine) to afford the title compound (306 mg, 31%) as a yellow oil. R_f = 0.36 (75:24:1 dichloromethane : acetone : triethylamine); ν_{max}/cm^{-1} (thin film) 2933, 1721, 1435, 1288, 1257, 1143, 1093, 968, 831, 778; δ_H (400 MHz, $CDCl_3$) 7.81 (d, 1H, *J* = 2.0 Hz), 7.53 (dd, 1H, *J* = 8.0, 2.0 Hz), 7.27 (d, 1H, *J* = 8.0 Hz), 4.47 (d, 1H, *J* = 13.5 Hz), 3.96 – 3.86 (m, 4H), 3.45 (dd, 1H, *J* = 12.0, 4.0 Hz), 3.32 (d, 1H, *J* = 13.5 Hz), 2.68 – 2.57 (m, 1H), 2.33 – 2.26 (m, 1H), 1.99 (t, 1H, *J* = 11.0 Hz), 1.72 – 1.63 (m, 3H), 1.54 – 1.47 (m, 1H), 1.37 – 1.25 (m, 2H); $^{13}C\{^1H\}$ NMR (100 MHz, $CDCl_3$) 168.5,

139.4, 134.2, 132.7, 132.5, 132.0, 120.6, 62.9, 62.7, 55.6, 52.7, 51.8, 27.5, 24.6, 23.7; HRMS (ESI) m/z: $[M + H]^+$ calcd. for $C_{15}H_{21}{}^{79}BrNO_3^+$, 342.0699; Found: 342.0697.

Methyl 4-bromo-2-((2-(hydroxymethyl)piperidin-1-yl)methyl)benzoate (8c)

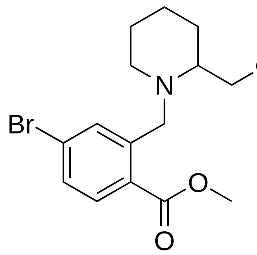

To a stirring solution of potassium carbonate (1.08 g, 7.79 mmol) in acetonitrile (20.0 mL), 2-piperidinemethanol (299 mg, 2.60 mmol) was added followed by methyl 4-bromo-2-(bromomethyl)benzoate **7c** (800 mg, 2.60 mmol). The reaction mixture was refluxed at 90 °C for 18 hours under argon. Then the reaction mixture was allowed to cool to RT, filtered and washed with dichloromethane, concentrated under vacuum and purified by column chromatography (SiO_2, 1:1 hexane : ethyl acetate) to afford the title compound (430 mg, 48%) as a yellow oil. R_f = 0.27 (1:1 hexane : ethyl acetate); ν_{max}/cm^{-1} (thin film) 2933, 1718, 1587, 1434, 1289, 1235, 1090, 868, 768; δ_H (400 MHz, $CDCl_3$) 7.59 – 7.51 (m, 2H), 7.42 (dd, 1H, *J* = 8.5, 2.0 Hz), 4.48 (d, 1H, *J* = 14.0 Hz), 3.91 – 3.83 (m, 4H), 3.45 (dd, 1H, *J* = 11.5, 4.0 Hz), 3.34 (d, 1H, *J* = 14.0 Hz), 2.63 (ddd, 1H, *J* = 11.5, 5.0, 3.0 Hz), 2.30 (dt, 1H, *J* = 8.0, 4.0 Hz), 2.04 – 1.95 (m, 1H), 1.72 – 1.59 (m, 3H), 1.54 – 1.46 (m, 1H), 1.40 – 1.27 (m, 2H); $^{13}C\{^1H\}$ NMR (100 MHz, $CDCl_3$) 169.0, 142.9, 133.1, 131.2, 130.2, 129.7, 126.0, 62.9, 62.7, 55.8, 52.6, 52.0, 27.5, 24.6, 23.7; HRMS (ESI) m/z: $[M + H]^+$ calcd. for $C_{15}H_{21}{}^{79}BrNO_3^+$, 342.0699; Found: 342.0692.

Methyl 3-bromo-2-((2-(hydroxymethyl)piperidin-1-yl)methyl)benzoate (8d)

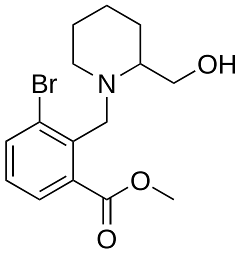

To a stirring solution of potassium carbonate (1.35 g, 9.74 mmol) in acetonitrile (20.0 mL), 2-piperidinemethanol (374 mg, 3.25 mmol) was added followed by methyl 3-bromo-2-(bromomethyl)benzoate **7d** (1.00 g, 3.25 mmol). The reaction mixture was refluxed at 90 °C for 18 hours under argon. Then the reaction mixture was allowed to cool to RT, filtered and washed with dichloromethane, concentrated under vacuum and purified by column chromatography (SiO_2, 5:1 → 2:1 hexane : ethyl acetate) to afford the title compound (818 mg, 74%) as a yellow oil. R_f = 0.54 (1:1 hexane : ethyl acetate); ν_{max}/cm^{-1} (thin film) 2936, 1714, 1432, 1279, 1208, 1150, 1093, 908, 753, 728; δ_H (400 MHz, $CDCl_3$) 7.61 (dd, 1H, *J* = 8.0, 1.5 Hz), 7.40 (dd, 1H, *J* = 8.0, 1.5 Hz), 7.13 (t, 1H, *J* = 8.0 Hz), 4.43 (d, 1H, *J* = 13.5 Hz), 3.90 (s, 3H), 3.81 (dd, 1H, *J* = 12.0, 4.0 Hz), 3.75 (d, 1H, *J* = 13.5 Hz), 3.45 (dd, 1H, *J* = 12.0, 4.0 Hz), 2.60

(ddd, 1H, *J* = 12.0, 7.0, 4.0 Hz), 2.28 (1H, dq, *J* = 7.0, 4.0 Hz), 2.09 (ddd, 1H, *J* = 12.0, 9.0, 4.0 Hz), 1.72 – 1.56 (m, 3H), 1.55 – 1.47 (m, 1H), 1.43 – 1.31 (m, 1H), 1.29 – 1.19 (m, 1H); $^{13}C\{^{1}H\}$ NMR (100 MHz, $CDCl_3$) 170.4, 138.2, 135.0, 134.9, 128.3, 127.6, 125.6, 62.8, 62.3, 54.6, 52.8, 51.1, 26.6, 24.4, 23.3; HRMS (ESI) m/z: $[M + H]^+$ calcd. for $C_{15}H_{21}{}^{79}BrNO_3^+$, 342.0699; Found: 342.0697.

4-Bromo-7,7a,8,9,10,11-hexahydrobenzo[f]pyrido[2,1-c][1,4]oxazocin-5(13H)-one (4a)

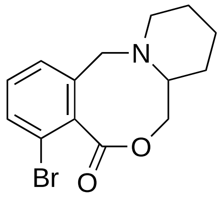

To a stirring solution of methyl 2-bromo-6-((2-(hydroxymethyl)piperidin-1-yl)methyl) benzoate **8a** (500 mg, 1.46 mmol) in methanol (3.2 mL), aqueous lithium hydroxide (0.5 M) was added (9.6 mL, 4.80 mmol) and the reaction mixture was heated to 80 °C for 49 hours. The reaction was then allowed to cool to RT, and the solvent was removed under vacuum using chloroform (5 × 100 mL) to form an azeotropic mixture. The intermediate 2-bromo-6-((2-(hydroxymethyl)piperidin-1-yl)methyl)benzoic acid was dissolved in chloroform (15 mL) and DIPEA (0.47 mL, 2.70 mmol) was added followed by T3P (50% w/v in ethyl acetate, 1.39 g, 2.19 mmol) and the mixture was stirred at room temperature for 16 hours under argon. The reaction mixture was then transferred into a separating funnel and water (30 mL) was added, and extracted with ethyl acetate (3 × 30 mL). The organic phases were then combined, dried with magnesium sulphate, filtered and concentrated in vacuo. The crude product was purified by column chromatography (SiO_2, 2:1 hexane : ethyl acetate) to afford the title compound (291 mg, 64%) as a white solid. R_f = 0.35 (2:1 hexane : ethyl acetate); m.p. 62–66 °C; ν_{max}/cm^{-1} (thin film) 2936, 1733, 1563, 1428, 1344, 1212, 1101, 1064, 992, 757; δ_H (400 MHz, $CDCl_3$) 7.57 (dd, 1H, *J* = 7.5, 1.5 Hz), 7.30 – 7.26 (m, 1H), 7.23 (d, 1H, *J* = 7.5 Hz), 4.09 (dd, 1H, *J* = 13.0, 6.5 Hz), 3.99 (dd, 1H, *J* = 13.0, 1.5 Hz), 3.70–3.62 (m, 2H), 2.98 (dt, 1H, *J* = 11.5, 3.5 Hz), 2.57 – 2.43 (m, 2H), 1.74 (dt, 1H, *J* = 13.0, 4.0 Hz), 1.66 – 1.57 (m, 3H), 1.55 – 1.47 (m, 1H), 1.37 – 1.28 (m, 1H); $^{13}C\{^{1}H\}$ NMR (100 MHz, $CDCl_3$) 168.6, 140.5, 132.4, 131.7, 131.1, 128.3, 120.5, 72.6, 64.5, 60.3, 57.6, 29.2, 25.8, 23.7; HRMS (ESI) m/z: $[M + H]^+$ calcd. for $C_{14}H_{17}{}^{79}BrNO_2^+$, 310.0437; Found: 310.0434.

3-Bromo-7,7a,8,9,10,11-hexahydrobenzo[f]pyrido[2,1-c][1,4]oxazocin-5(13H)-one (4b)

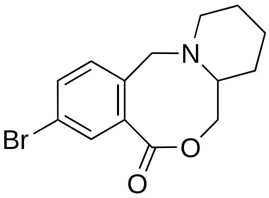

To a stirring solution of methyl 5-bromo-2-((2-(hydroxymethyl)piperidin-1-yl)methyl) benzoate **8b** (200 mg, 0.584 mmol) in methanol (1.3 mL), aqueous lithium hydroxide (0.5 M) was added (1.3 mL, 0.643 mmol) and the reaction mixture was heated to 50 °C for 1 hour. The reaction was then allowed to cool to RT, and the solvent was removed under vacuum using

chloroform (5 × 50 mL) to form an azeotropic mixture. The intermediate 5-bromo-2-((2-(hydroxymethyl)piperidin-1-yl)methyl)benzoic acid was dissolved in chloroform (6.0 mL) and DIPEA (0.19 mL, 1.08 mmol) was added followed by T3P (50% w/v in ethyl acetate, 558 mg, 0.877 mmol) and the mixture was stirred at room temperature for 18 hours under argon. The reaction mixture was then transferred into a separating funnel and water (10 mL) was added, and extracted with dichloromethane (3 × 10 mL). The organic phases were then combined, dried with magnesium sulphate, filtered and concentrated in vacuo. The crude product was purified by column chromatography (SiO_2, 5:1 → 2:1 hexane : ethyl acetate) to afford the title compound (144 mg, 80%) as a white solid. R_f = 0.62 (2:1 hexane : ethyl acetate); m.p. 81–85 °C; ν_{max}/cm^{-1} (thin film) 2937, 1719, 1465, 1369, 1267, 1090, 996, 820; δ_H (400 MHz, $CDCl_3$) 7.61 (d, 1H, *J* = 2.0 Hz), 7.53 (dd, 1H, *J* = 8.0, 2.0 Hz), 7.10 (d, 1H, *J* = 8.0 Hz), 4.04 (dd, 1H, *J* = 12.5, 2.0 Hz), 3.98 (dd, 1H, *J* = 12.5, 5.0 Hz), 3.64 (s, 2H), 2.96 (dt, 1H, *J* = 11.5, 4.0 Hz), 2.51 – 2.38 (m, 2H), 1.74 (dt, 1H, *J* = 13.0, 4.0 Hz), 1.65 – 1.46 (m, 4H), 1.35 – 1.22 (m, 1H); $^{13}C\{^1H\}$ NMR (100 MHz, $CDCl_3$) 170.6, 137.9, 134.3, 132.5, 131.2, 130.5, 121.2, 72.2, 64.4, 60.2, 57.6, 29.0, 25.9, 23.8; HRMS (ESI) m/z: $[M + H]^+$ calcd. for $C_{14}H_{17}{}^{79}BrNO_2{}^+$, 310.0437; Found: 310.0435.

2-Bromo-7,7a,8,9,10,11-hexahydrobenzo[f]pyrido[2,1-c][1,4]oxazocin-5(13H)-one (4c)

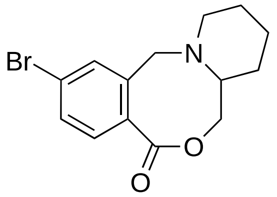

To a stirring solution of methyl 4-bromo-2-((2-(hydroxymethyl)piperidin-1-yl)methyl) benzoate **8c** (400 mg, 1.17 mmol) in methanol (2.6 mL), aqueous lithium hydroxide (0.5 M) was added (2.6 mL, 1.29 mmol) and the reaction mixture was heated to 50 °C for 4.5 hours. The reaction was then allowed to cool to RT, and the solvent was removed under vacuum using chloroform (5 × 50 mL) to form an azeotropic mixture. The intermediate 4-bromo-2-((2-(hydroxymethyl)piperidin-1-yl)methyl)benzoic acid was dissolved in chloroform (12 mL) and DIPEA (0.38 mL, 2.16 mmol) was added followed by T3P (50% w/v in ethyl acetate, 1.12 g, 1.75 mmol) and the mixture was stirred at room temperature for 16 hours under argon. The reaction mixture was then transferred into a separating funnel and water (30 mL) was added, and extracted with dichloromethane (3 × 30 mL). The organic phases were then combined, dried with magnesium sulphate, filtered and concentrated in vacuo. The crude product was purified by column chromatography (SiO_2, 5:1 → 2:1 hexane : ethyl acetate) to afford the title compound (279 mg, 77%) as a white solid. R_f = 0.66 (2:1 hexane : ethyl acetate); m.p. 79–82 °C; ν_{max}/cm^{-1} (thin film) 2937, 1716, 1588, 1464, 1270, 1094, 994, 910, 727; δ_H (400 MHz, $CDCl_3$) 7.43 (dd, 1H, *J* = 8.5, 2.0 Hz), 7.38 (d, 1H, *J* = 2.0 Hz), 7.34 (d, 1H, *J* = 8.5 Hz), 4.11 – 3.92 (m, 2H, OC**H2**), 3.72 – 3.56 (m, 2H), 2.96 (dt, 1H, *J* = 11.0, 3.5 Hz), 2.53 – 2.33 (m, 2H), 1.73 (dt, 1H, *J* = 12.5, 3.5 Hz), 1.65 – 1.44 (m, 4H), 1.35 – 1.20 (m, 1H); $^{13}C\{^1H\}$ NMR (100 MHz, $CDCl_3$) 171.2, 140.9, 131.9, 131.5, 130.9, 128.3, 125.7, 72.3, 64.4, 60.2, 57.6, 28.9, 25.8, 23.8; HRMS (ESI) m/z: $[M + H]^+$ calcd. for $C_{14}H_{17}{}^{79}BrNO_2{}^+$, 310.0437; Found: 310.0444.

1-Bromo-7,7a,8,9,10,11-hexahydrobenzo[f]pyrido[2,1-c][1,4]oxazocin-5(13H)-one (4d)

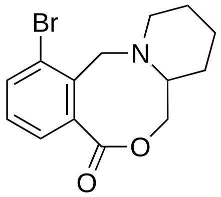

To a stirring solution of methyl 3-bromo-2-((2-(hydroxymethyl)piperidin-1-yl)methyl) benzoate **8d** (500 mg, 1.46 mmol) in methanol (3.2 mL), aqueous lithium hydroxide (0.5 M) was added (3.2 mL, 1.61 mmol) and the reaction mixture was heated to 50 °C for 3 hours. The reaction was then allowed to cool to RT, and the solvent was removed under vacuum using chloroform (5 × 100 mL) to form an azeotropic mixture. The intermediate 3-bromo-2-((2-(hydroxymethyl)piperidin-1-yl)methyl)benzoic acid was dissolved in chloroform (15 mL) and DIPEA (0.47 mL, 2.70 mmol) was added followed by T3P (50% w/v in ethyl acetate, 1.40 g, 2.19 mmol) and the mixture was stirred at room temperature for 16.5 hours under argon. The reaction mixture was then transferred into a separating funnel and water (30 mL) was added, and extracted with dichloromethane (3 × 30 mL). The organic phases were then combined, dried with magnesium sulphate, filtered and concentrated in vacuo. The crude product was purified by column chromatography (SiO_2, 5:1 →3:1 hexane : ethyl acetate) to afford the title compound (285 mg, 63%) as a white solid. R_f = 0.58 (3:1 hexane : ethyl acetate); m.p. 104–107 °C; ν_{max}/cm^{-1} (thin film) 2937, 1722, 1465, 1285, 1093, 751; δ_H (400 MHz, $CDCl_3$) 7.66 (d, 1H, *J* = 8.0 Hz), 7.41 (d, 1H, *J* = 7.5 Hz), 7.18 (t, 1H, *J* = 8.0 Hz), 4.00–3.91 (m, 2H), 3.77 (d, 1H, *J* = 16.0 Hz), 3.61 (d, 1H, *J* = 16.0 Hz), 3.13 (dt, 1H, *J* = 11.5, 3.0 Hz), 2.58 – 2.41 (m, 2H), 1.75 (dt, 1H, *J* = 12.5, 3.5 Hz), 1.66 – 1.43 (m, 4H), 1.36 – 1.20 (m, 1H); $^{13}C\{^1H\}$ NMR (100 MHz, $CDCl_3$) 171.2, 137.9, 135.4, 131.9, 128.9, 128.8, 124.3, 72.0, 64.1, 60.5, 57.0, 28.7, 25.8, 23.8; HRMS (ESI) m/z: $[M + H]^+$ calcd. for $C_{14}H_{17}{}^{79}BrNO_2^+$, 310.0437; Found: 312.0418.

Methyl (S)-5-bromo-2-((2-(hydroxymethyl)pyrrolidin-1-yl)methyl)benzoate (8e)

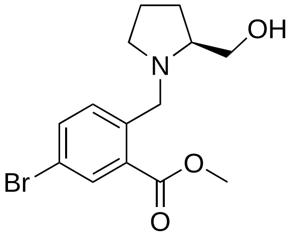

To a stirring solution of potassium carbonate (1.34 g, 9.74 mmol) in acetonitrile (20.0 mL), (R)-pyrrolidin-2-ylmethanol (328 mg, 3.25 mmol) was added followed by methyl 5-bromo-2-(bromomethyl)benzoate **7b** (1.00 g, 3.25 mmol). The reaction mixture was refluxed at 90 °C for 16 hours under argon. Then the reaction mixture was allowed to cool to RT, filtered and washed with dichloromethane, concentrated under vacuum and purified by column chromatography (SiO_2, 99:1 ethyl acetate : triethylamine) to afford the title compound (654 mg, 61%) as a yellow oil. R_f = 0.31 (99:1 ethyl acetate : triethylamine); ν_{max}/cm^{-1} (thin film) 2951, 1723, 1435, 1287, 1258, 1077; δ_H (400 MHz, $CDCl_3$) 7.89 – 7.83 (m, 1H), 7.54 – 7.49 (m, 1H), 7.25 (d, 1H, *J* = 8.0 Hz), 4.38 (d, 1H, *J* = 13.5 Hz), 3.86 (s, 3H), 3.68 – 3.63 (m, 1H), 3.42 – 3.33 (m, 2H), 2.75 (t, 1H, *J* = 6.5 Hz), 2.62 – 2.56 (m, 1H), 2.16 – 2.08 (m, 1H), 1.87 – 1.70 (m,

2H), 1.68 – 1.52 (m, 2H); $^{13}C\{^1H\}$ NMR (100 MHz, $CDCl_3$) 167.8, 139.8, 134.4, 132.9, 132.3, 131.9, 120.7, 65.5, 62.1, 56.7, 54.9, 52.6, 27.2, 23.3; HRMS (ESI) m/z: $[M + H]^+$ calcd. for $C_{14}H_{19}{}^{79}BrNO_3^+$, 328.0543; Found: 328.0536.

(*S*)-8-Bromo-2,3,3a,4-tetrahydro-1H-benzo[f]pyrrolo[2,1-c][1,4]oxazocin-6(11H)-one (4e)

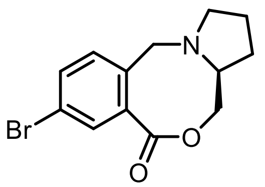

To a stirring solution of methyl (S)-5-bromo-2-((2-(hydroxymethyl)pyrrolidin-1-yl)methyl) benzoate **8e** (500 mg, 1.52 mmol) in methanol (3.3 mL), aqueous lithium hydroxide (0.5 M) was added (3.3 mL, 1.68 mmol) and the reaction mixture was heated to 50 °C for 2 hours. The reaction was then allowed to cool to RT, and the solvent was removed under vacuum using chloroform (5 × 100 mL) to form an azeotropic mixture. The intermediate (S)-5-bromo-2-((2-(hydroxymethyl)pyrrolidin-1-yl)methyl)benzoic acid was dissolved in chloroform (15 mL) and DIPEA (0.49 mL, 2.82 mmol) was added followed by T3P (50% w/v in ethyl acetate, 1.45 g, 2.28 mmol) and the mixture was stirred at room temperature for 17 hours under argon. The reaction mixture was then transferred into a separating funnel and water (30 mL) was added, and extracted with ethyl acetate (3 × 30 mL). The organic phases were then combined, dried with magnesium sulphate, filtered and concentrated in vacuo. The crude product was purified by column chromatography (SiO_2, 2:1 → 1:1 hexane : ethyl acetate) to afford the title compound (439 mg, 97%) as a white solid. R_f = 0.52 (2:1 hexane : ethyl acetate); m.p. 93–96 °C; ν_{max}/cm^{-1} (thin film) 2954, 2817, 1712, 1477, 1344, 1258, 1204, 1092, 1045, 840; δ_H (400 MHz, $CDCl_3$) 7.49 (d, 1H, *J* = 2.0 Hz), 7.45 (dd, 1H, *J* = 8.0, 2.0 Hz), 7.00 (d, 1H, *J* = 8.0 Hz), 4.13 (d, 1H, *J* = 16.5 Hz), 4.01 (dd, 1H, *J* = 12.0, 1.5 Hz), 3.89 – 3.77 (m, 2H), 3.21 – 3.13 (m, 1H), 2.87 – 2.72 (m, 2H), 2.10 – 1.99 (m, 1H), 1.95 – 1.83 (m, 2H), 1.82 – 1.71 (m, 1H); $^{13}C\{^1H\}$ NMR (100 MHz, $CDCl_3$) 171.7, 138.6, 132.9, 132.7, 131.5, 127.3, 120.7, 69.1, 64.2, 55.6, 55.2, 30.2, 23.6; HRMS (ESI) m/z: $[M + H]^+$ calcd. for $C_{13}H_{15}{}^{79}BrNO_2^+$, 296.0281; Found: 296.0275. $[\alpha]_D^{20}$ = +16.4 (c = 1.0, $CHCl_3$)

Methyl 5-bromo-2-((2-(2-hydroxyethyl)piperidin-1-yl)methyl)benzoate (8f)

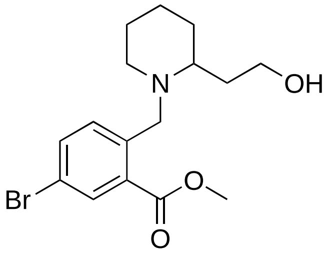

To a stirring solution of potassium carbonate (4.71 g, 34.1 mmol) in acetonitrile (70.0 mL), 2-(piperidin-2-yl)ethan-1-ol (1.47 g, 11.4 mmol) was added followed by methyl 5-bromo-2-(bromomethyl)benzoate **7b** (3.50 mg, 11.4 mmol). The reaction mixture was refluxed at 90 °C for 17 hours under argon. Then the reaction mixture was allowed to cool to RT, filtered and washed with dichloromethane, concentrated under vacuum and purified by column

chromatography (SiO_2, 99:1 ethyl acetate : triethylamine) to afford the title compound (2.77g, 69%) as a yellow oil. R_f = 0.39 (99:1 ethyl acetate : triethylamine); ν_{max}/cm^{-1} (thin film) 2933, 1725, 1435, 1287, 1242, 1094, 1076, 971, 830, 778; δ_H (400 MHz, $CDCl_3$) 7.96 (d, 1H, *J* = 2.0 Hz), 7.57 (dd, 1H, *J* = 8.5, 2.0 Hz), 7.36 (d, 1H, *J* = 8.5 Hz), 4.23 (d, 1H, *J* = 14.0 Hz), 3.92–3.88 (m, 4H, OC**H₃**), 3.76 (dt, 1H, *J* = 11.0, 5.0 Hz), 3.62 (ddd, 1H, *J* = 11.0, 7.5, 5.0 Hz), 2.86 (ddd, 1H, *J* = 13.0, 9.0, 3.0 Hz), 2.78 (s, 1H), 2.32 (s, 1H), 1.98 (ddd, 1H, *J* = 13.0, 7.5, 5.0 Hz), 1.80 (ddd, 1H, *J* = 13.0, 9.0, 4.0 Hz), 1.69 – 1.56 (m, 3H), 1.52 – 1.35 (m, 3H); $^{13}C\{^1H\}$ NMR (100 MHz, $CDCl_3$) 167.1, 140.1, 134.6, 133.2, 132.3, 131.9, 120.5, 61.9, 58.8, 54.6, 52.4, 48.7, 31.7, 27.4, 22.7, 21.7; HRMS (ESI) m/z: [M + H]$^+$ calcd. for $C_{16}H_{23}{}^{79}BrNO_3^+$, 356.0856; Found: 358.0830.

3-Bromo-8,8a,9,10,11,12-hexahydro-7H-benzo[g]pyrido[2,1-d][1,5]oxazonin-5(14H)-one (4f)

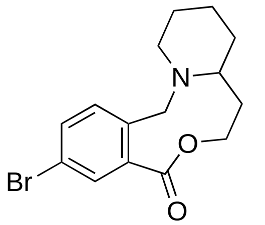

To a stirring solution of methyl 5-bromo-2-((2-(2-hydroxyethyl)piperidin-1-yl)methyl) benzoate **8f** (2.50 g, 7.02 mmol) in methanol (15.4 mL), aqueous lithium hydroxide (0.5 M) was added (15.4 mL, 7.72 mmol) and the reaction mixture was heated to 50 °C for 2 hours. The reaction was then allowed to cool to RT, and the solvent was removed under vacuum using chloroform (5 × 200 mL) to form an azeotropic mixture. The intermediate 5-bromo-2-((2-(2-hydroxyethyl)piperidin-1-yl)methyl)benzoic acid was dissolved in chloroform (70 mL) and DIPEA (2.3 mL, 13.0 mmol) was added followed by T3P (50% w/v in ethyl acetate, 6.70 g, 10.5 mmol) and the mixture was stirred at room temperature for 17 hours under argon. The reaction mixture was then transferred into a separating funnel and water (80 mL) was added, and extracted with ethyl acetate (3 × 80 mL). The organic phases were then combined, dried with magnesium sulphate, filtered and concentrated in vacuo. The crude product was purified by column chromatography (SiO_2, 20:1 → 10:1 hexane : ethyl acetate) to afford the title compound (1.38 g, 61%) as a white solid. R_f = 0.42 (10:1 hexane : ethyl acetate); m.p. 99–102 °C; ν_{max}/cm^{-1} (thin film) 2931, 1726, 1462, 1257, 1138, 1090, 998, 814, 745; δ_H (400 MHz, $CDCl_3$) 7.66 (d, 1H, *J* = 2.5 Hz), 7.42 (dd, 1H, *J* = 8.0, 2.5 Hz), 7.05 (d, 1H, *J* = 8.0 Hz), 5.27 (t, 1H, *J* = 12.0 Hz), 4.55 (d, 1H, *J* = 14.0 Hz), 4.00 (dt, 1H, *J* = 12.0, 3.0 Hz), 2.72 (d, 1H, *J* = 14.0 Hz), 2.68 – 2.44 (m, 3H), 1.88 (td, 1H, *J* = 11.5, 3.0 Hz), 1.70 – 1.57 (m, 2H), 1.46 – 1.30 (m, 3H), 1.27 – 1.15 (m, 2H); $^{13}C\{^1H\}$ NMR (100 MHz, $CDCl_3$) 170.3, 142.0, 135.3, 132.8, 131.6, 130.5, 120.5, 63.3, 58.3, 56.6, 53.4, 31.9, 31.0, 25.6, 24.5; HRMS (ESI) m/z: [M + H]$^+$ calcd. for $C_{15}H_{19}{}^{79}BrNO_2^+$, 324.0594; Found: 324.0580.

(5-Oxo-5,8,8a,9,10,11,12,14-octahydro-7H-benzo[g]pyrido[2,1-d][1,5]oxazonin-3-yl) boronic acid (4g)

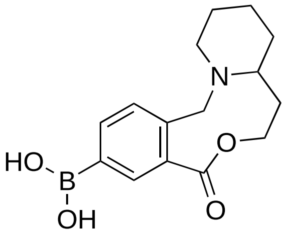

3-Bromo-8,8a,9,10,11,12-hexahydro-7H-benzo[g]pyrido[2,1-d][1,5]oxazonin-5(14H)-one **4f** (1.00 g, 3.08 mmol) and bis(pinacolato)diboron (1.57g, 6.17 mmol) were dissolved in 1,4-dioxane (20 mL). Then Na_2CO_3 (981 mg, 9.25 mmol) which dissolved in water (10 mL) was added followed by $Pd(dppf)Cl_2{\cdot}CH_2Cl_2$ (125 mg, 0.154 mmol) and the resulting mixture was purged with argon for 10 minutes. The mixture was stirred and heated at 50 °C for 16.5 hours under argon. After being allowed to cool to RT, water (50 mL) was added and the two layers were separated. The aqueous layer was extracted with dichloromethane (3 x 50 mL) and the combined organic phases were dried with magnesium sulphate, filtered and concentrated in vacuo to give the crude product. The product was purified by column chromatography (SiO_2, 5:1 hexane : ethyl acetate) to afford the title compound (365 mg, 41%) as a white solid. R_f = 0.23 (5:1 hexane : ethyl acetate); m.p. 201–206 °C; v_{max}/cm^{-1} (thin film) 2931, 1725, 1462, 1262, 1139, 1087, 997, 816, 734, 701; δ_H (400 MHz, $CDCl_3$) 7.78 (s, 1H), 7.54 (d, 1H, *J* = 8.0 Hz), 7.23 (d, 1H, *J* = 8.0 Hz), 5.35 – 5.26 (m, 1H), 4.65 (d, 1H, *J* = 14.0 Hz), 4.00 (d, 1H, *J* = 11.5 Hz), 2.77 (d, 1H, *J* = 14.0 Hz), 2.73 – 2.57 (m, 3H), 1.90 (t, 1H, *J* = 11.0 Hz), 1.73 – 1.57 (m, 2H), 1.47 – 1.19 (m, 5H); $^{13}C\{^1H\}$ NMR (100 MHz, $CDCl_3$) 171.7, 142.2, 139.0, 134.0, 129.5, 128.3, 127.1, 63.1, 58.3, 56.8, 53.5, 32.0, 31.1, 25.7, 24.5; HRMS (ESI) m/z: $[M + H]^+$ calcd. for $C_{15}H_{20}BNO_4^+$, 290.1558; Found: 290.1561.

3-(4,4,5,5-Tetramethyl-1,3,2-dioxaborolan-2-yl)-8,8a,9,10,11,12-hexahydro-7H-benzo[g] pyrido[2,1-d][1,5]oxazonin-5(14H)-one (4h)

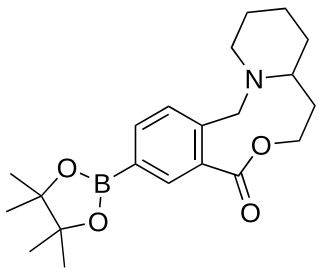

3-Bromo-8,8a,9,10,11,12-hexahydro-7H-benzo[g]pyrido[2,1-d][1,5]oxazonin-5(14H)-one **4f** (100 mg, 0.308 mmol), bis(pinacolato)diboron (156.6 mg, 0.716 mmol), $Pd(dppf)Cl_2$ (22.7 mg, 0.031 mmol) and potassium acetate (60.6 mg, 0.617 mmol) were added to a round-bottomed flask. The round-bottomed flask was purged with argon and then 1,4-dioxane (3 mL) was added. The resulting mixture was stirred and heated at 60 °C for 23 hours under argon. After being allowed to cool to RT, the solids were removed by filtration and washed with dichloromethane (10 mL). The solvents were evaporated under reduced pressure to give the crude product. The crude product was purified by column chromatography (SiO_2, 10:1 hexane

: ethyl acetate) to afford the title compound (72.1 mg, 63%) as a white solid. R_f = 0.59 (10:1 hexane : ethyl acetate); m.p. 120–124 °C; v_{max}/cm^{-1} (thin film) 2979, 1730, 1612, 1355, 1280, 1258, 1122, 1078, 963, 848, 731, 669; δ_H (400 MHz, $CDCl_3$) 8.01 (s, 1H), 7.75 (d, 1H, *J* = 7.5 Hz), 7.19 (d, 1H, *J* = 7.5 Hz), 5.29 (t, 1H, *J* = 12.0 Hz), 4.66 (d, 1H, *J* = 14.0 Hz), 4.19 – 3.82 (m, 1H), 2.76 (d, 1H, *J* = 14.0 Hz), 2.72 – 2.43 (m, 3H, NC**H**), 1.88 (t, 1H, *J* = 11.0 Hz), 1.76 – 1.59 (m, 2H), 1.48 – 1.40 (m, 1H), 1.32 (s, 14H), 1.25 – 1.14 (m, 2H); $^{13}C\{^1H\}$ NMR (100 MHz, $CDCl_3$) 171.9, 146.2, 136.6, 136.6, 135.0, 133.1, 128.4, 84.1, 63.0, 58.4, 57.3, 53.6, 32.1, 31.1, 25.7, 25.0, 24.6; HRMS (ESI) m/z: $[M + H]^+$ calcd. for $C_{21}H_{31}BNO_4^+$, 372.2341; Found: 372.2349.

4-Phenyl-7,7a,8,9,10,11-hexahydrobenzo[f]pyrido[2,1-c][1,4]oxazocin-5(13H)-one (5a)

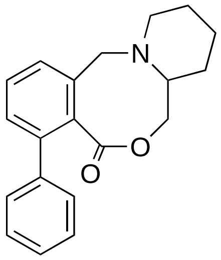

4-Bromo-7,7a,8,9,10,11-hexahydrobenzo[f]pyrido [2,1-c][1,4]oxazocin-5(13H)-one **4a** (100 mg, 0.322 mmol) and phenylboronic acid (78.6 mg, 0.644 mmol) were dissolved in 1,4-dioxane (5.0 mL). Then Na_2CO_3 (103 mg, 0.967 mmol) which dissolved in water (2.5 mL) was added followed by $Pd(dppf)Cl_2 \cdot CH_2Cl_2$ (13.1 mg, 0.016 mmol) and the resulting mixture was purged with argon for 10 minutes. The mixture was stirred and heated at 50 °C for 16 hours under argon. After being allowed to cool to RT, water (20 mL) was added and the two layers were separated. The aqueous layer was extracted with dichloromethane (3 x 20 mL) and the combined organic phases were dried with magnesium sulphate, filtered and concentrated in vacuo to give the crude product. The product was purified by column chromatography (SiO_2, 3:1 → 2:1 hexane : ethyl acetate) to afford the title compound (80.5 mg, 81%) as a white solid. R_f = 0.52 (2:1 hexane : ethyl acetate); m.p. 101–104 °C; v_{max}/cm^{-1} (thin film) 2935, 1731, 1457, 1266, 1234, 1105, 1066, 1010, 755, 728, 699; δ_H (400 MHz, $CDCl_3$) 7.49 (t, 1H, *J* = 7.5 Hz), 7.43 – 7.34 (m, 4H), 7.34 – 7.27 (m, 3H), 4.48 (dd, 1H, *J* = 12.5, 7.0 Hz), 4.14 (d, 1H, *J* = 12.5 Hz), 3.73 (d, 1H, *J* = 14.5 Hz), 3.64 (d, 1H, *J* = 14.5 Hz), 3.03 (dt, 1H, *J* = 11.5, 4.0 Hz), 2.62 (ddd, 1H, *J* = 10.5, 7.0, 3.0 Hz), 2.52 (td, 1H, *J* = 11.5, 4.0 Hz), 1.79 – 1.71 (m, 1H), 1.69 – 1.56 (m, 2H), 1.56 – 1.48 (m, 1H), 1.44 – 1.28 (m, 2H); $^{13}C\{^1H\}$ NMR (100 MHz, $CDCl_3$) 170.5, 141.5, 140.1, 138.1, 131.0, 129.7, 129.3, 129.3, 128.6, 128.3, 127.8, 73.0, 65.0, 60.3, 58.2, 29.5, 26.1, 24.0; HRMS (ESI) m/z: $[M + H]^+$ calcd. for $C_{20}H_{22}NO_2^+$, 308.1645; Found: 308.1647.

3-Phenyl-7,7a,8,9,10,11-hexahydrobenzo[f]pyrido[2,1-c][1,4]oxazocin-5(13H)-one (5b)

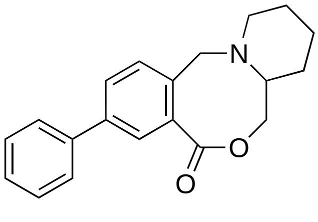

3-Bromo-7,7a,8,9,10,11-hexahydrobenzo[f]pyrido[2,1-c][1,4]oxazocin-5(13H)-one **4b** (100 mg, 0.322 mmol) and phenylboronic acid (78.6 mg, 0.644 mmol) were dissolved in 1,4-

dioxane (5.0 mL). Then Na_2CO_3 (103 mg, 0.967 mmol) which dissolved in water (2.5 mL) was added followed by $Pd(dppf)Cl_2 \cdot CH_2Cl_2$ (13.1 mg, 0.016 mmol) and the resulting mixture was purged with argon for 10 minutes. The mixture was stirred and heated at 50 °C for 18 hours under argon. After being allowed to cool to RT, water (20 mL) was added and the two layers were separated. The aqueous layer was extracted with dichloromethane (3 x 20 mL) and the combined organic phases were dried with magnesium sulphate, filtered and concentrated in vacuo to give the crude product. The product was purified by column chromatography (SiO_2, 5:1 → 2:1 hexane : ethyl acetate) to afford the title compound (77.0 mg, 78%) as a yellow solid. R_f = 0.53 (2:1 hexane : ethyl acetate); m.p. 104–107 °C; v_{max}/cm^{-1} (thin film) 2937, 1718, 1485, 1312, 1228, 1122, 1089, 757, 698; δ_H (400 MHz, $CDCl_3$) 7.74 (d, 1H, *J* = 2.0 Hz), 7.67 (dd, 1H, *J* = 8.0, 2.0 Hz), 7.58 (dd, 2H, *J* = 7.5, 2.0 Hz), 7.43 (t, 2H, *J* = 7.5 Hz), 7.39 – 7.30 (m, 2H), 4.12–4.04 (m, 2H), 3.79 – 3.65 (m, 2H), 3.02 (dt, 1H, *J* = 10.0, 3.5 Hz), 2.49 (td, 2H, *J* = 10.0, 3.5 Hz), 1.76 (dt, 1H, *J* = 13.0, 3.5 Hz), 1.69 – 1.56 (m, 2H), 1.51 (td, 2H, *J* = 10.0, 3.5 Hz), 1.38 – 1.27 (m, 1H); $^{13}C\{^1H\}$ NMR (100 MHz, $CDCl_3$) 172.2, 140.8, 139.6, 137.6, 130.1, 129.9, 129.8, 129.0, 128.3, 127.9, 127.0, 72.5, 64.7, 60.4, 57.7, 29.2, 26.0, 23.9; HRMS (ESI) m/z: $[M + H]^+$ calcd. for $C_{20}H_{22}NO_2^+$, 308.1645; Found: 308.1649.

2-Phenyl-7,7a,8,9,10,11-hexahydrobenzo[f]pyrido[2,1-c][1,4]oxazocin-5(13H)-one (5c)

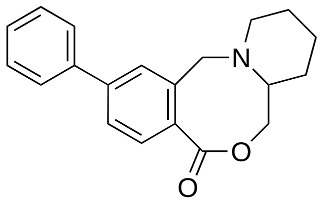

2-Bromo-7,7a,8,9,10,11-hexahydrobenzo[f]pyrido[2,1-c][1,4]oxazocin-5(13H)-one **4c** (100 mg, 0.322 mmol) and phenylboronic acid (78.6 mg, 0.644 mmol) were dissolved in 1,4-dioxane (5.0 mL). Then Na_2CO_3 (103 mg, 0.967 mmol) which dissolved in water (2.5 mL) was added followed by $Pd(dppf)Cl_2 \cdot CH_2Cl_2$ (13.1 mg, 0.016 mmol) and the resulting mixture was purged with argon for 10 minutes. The mixture was stirred and heated at 50 °C for 16 hours under argon. After being allowed to cool to RT, water (20 mL) was added and the two layers were separated. The aqueous layer was extracted with dichloromethane (3 x 20 mL) and the combined organic phases were dried with magnesium sulphate, filtered and concentrated in vacuo to give the crude product. The product was purified by column chromatography (SiO_2, 5:1 → 3:1 hexane : ethyl acetate) to afford the title compound (88.0 mg, 89%) as a white solid. R_f = 0.55 (3:1 hexane : ethyl acetate); m.p. 111–114 °C; v_{max}/cm^{-1} (thin film) 2937, 1713, 1609, 1449, 1277, 1240, 1092, 993, 912, 754, 730, 697; 7.63 – 7.51 (m, 4H), 7.49 – 7.40 (m, 3H), 7.42 – 7.32 (m, 1H), 4.16 – 4.04 (m, 2H), 3.79–3.71 (m, 2H), 3.01 (d, 1H, *J* = 11.5 Hz), 2.49 (td, 2H, *J* = 11.5, 4.0 Hz), 1.75 (dt, 1H, *J* = 13.0, 4.0 Hz), 1.69 – 1.54 (m, 2H), 1.57 – 1.47 (m, 2H), 1.39 – 1.23 (m, 1H); $^{13}C\{^1H\}$ NMR (100 MHz, $CDCl_3$) 172.1, 144.2, 139.8, 139.3, 130.5, 128.9, 128.1, 128.1, 127.8, 127.2, 126.3, 72.4, 64.6, 60.8, 57.7, 29.0, 25.9, 23.8; HRMS (ESI) m/z: $[M + H]^+$ calcd. for $C_{20}H_{22}NO_2^+$, 308.1645; Found: 308.1643.

1-Phenyl-7,7a,8,9,10,11-hexahydrobenzo[f]pyrido[2,1-c][1,4]oxazocin-5(13H)-one (5d)

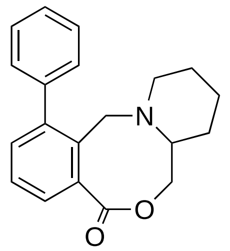

1-Bromo-7,7a,8,9,10,11-hexahydrobenzo[f]pyrido[2,1-c][1,4]oxazocin-5(13H)-one **4d** (100 mg, 0.322 mmol) and phenylboronic acid (78.6 mg, 0.644 mmol) were dissolved in 1,4-dioxane (5.0 mL). Then Na_2CO_3 (103 mg, 0.967 mmol) which dissolved in water (2.5 mL) was added followed by $Pd(dppf)Cl_2 \cdot CH_2Cl_2$ (13.1 mg, 0.016 mmol) and the resulting mixture was purged with argon for 10 minutes. The mixture was stirred and heated at 50 °C for 17.5 hours under argon. After being allowed to cool to RT, water (20 mL) was added and the two layers were separated. The aqueous layer was extracted with dichloromethane (3 x 20 mL) and the combined organic phases were dried with magnesium sulphate, filtered and concentrated in vacuo to give the crude product. The product was purified by column chromatography (SiO_2, 8:1 → 4:1 hexane : ethyl acetate) to afford the title compound (77.5 mg, 78%) as a white solid. R_f = 0.51 (4:1 hexane : ethyl acetate); m.p. 130–133 °C; v_{max}/cm^{-1} (thin film) 2931, 1721, 1464, 1303, 1269, 1109, 1071, 750, 702; δ_H (400 MHz, $CDCl_3$) 7.51 (dd, 1H, *J* = 6.5, 3.0 Hz), 7.46 (d, 2H, *J* = 6.5 Hz), 7.43 – 7.35 (m, 5H, 5 x Ar**H**), 4.25 (dd, 1H, *J* = 12.5, 7.0 Hz), 4.01 (d, 1H, *J* = 12.5 Hz), 3.45 (d, 1H, *J* = 14.5 Hz), 3.29 (d, 1H, *J* = 14.5 Hz), 2.52 (ddd, 1H, *J* = 10.5, 7.0, 2.5 Hz), 2.38 (dt, 1H, *J* = 11.5, 3.5 Hz), 2.14 (td, 1H, *J* = 11.5, 3.5 Hz), 1.80 – 1.70 (m, 1H), 1.59 – 1.41 (m, 3H), 1.38 – 1.21 (m, 2H); $^{13}C\{^1H\}$ NMR (100 MHz, $CDCl_3$) 172.2, 143.4, 140.6, 136.2, 133.3, 130.6, 129.8, 128.3, 127.9, 127.7, 127.5, 73.6, 64.3, 56.9, 56.5, 29.6, 26.1, 24.2; HRMS (ESI) m/z: $[M + H]^+$ calcd. for $C_{20}H_{22}NO_2^+$, 308.1645; Found: 308.1653.

(*S*)-8-Phenyl-2,3,3a,4-tetrahydro-1H-benzo[f]pyrrolo[2,1-c][1,4]oxazocin-6(11H)-one (5e)

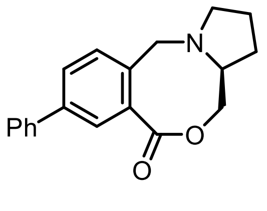

8-Bromo-2,3,3a,4-tetrahydro-1H-benzo[f]pyrrolo[2,1-c][1,4]oxazocin-6(11H)-one **4e** (100 mg, 0.338 mmol) and phenylboronic acid (82.3 mg, 0.675 mmol) were dissolved in 1,4-dioxane (5.0 mL). Then Na_2CO_3 (107 mg, 1.01 mmol) which dissolved in water (2.5 mL) was added followed by $Pd(dppf)Cl_2 \cdot CH_2Cl_2$ (13.7 mg, 0.017 mmol) and the resulting mixture was purged with argon for 10 minutes. The mixture was stirred and heated at 50 °C for 18 hours under argon. After being allowed to cool to RT, water (20 mL) was added and the two layers were separated. The aqueous layer was extracted with ethyl acetate (3 x 20 mL) and the combined organic phases were dried with magnesium sulphate, filtered and concentrated in vacuo to give the crude product. The product was purified by column chromatography (SiO_2,

2:1 → 1:1 hexane : ethyl acetate) to afford the title compound (67.9 mg, 69%) as a white solid. R_f = 0.62 (1:1 hexane : ethyl acetate); m.p. 131–138 °C; ν_{max}/cm^{-1} (thin film) 2966, 1714, 1485, 1345, 1304, 1203, 1093, 1043, 759, 699; δ_H (400 MHz, $CDCl_3$) 7.66 – 7.52 (m, 4H), 7.43 (t, 2H, *J* = 7.5 Hz), 7.38 – 7.30 (m, 1H), 7.20 (d, 1H, *J* = 8.0 Hz), 4.24 (d, 1H, *J* = 17.0 Hz), 4.03 (dd, 1H, *J* = 12.0, 2.0 Hz), 3.95 (dd, 1H, *J* = 12.0, 3.5 Hz), 3.88 (d, 1H, *J* = 17.0 Hz), 3.26 – 3.14 (m, 1H), 2.87 – 2.72 (m, 2H), 2.12 – 2.00 (m, 1H), 1.98 – 1.86 (m, 2H), 1.84 – 1.71 (m, 1H); $^{13}C\{^1H\}$ NMR (100 MHz, $CDCl_3$) 173.6, 140.5, 139.8, 138.3, 131.4, 128.9, 128.5, 127.7, 127.4, 127.0, 126.5, 68.9, 64.3, 55.8, 55.2, 30.1, 23.4; HRMS (ESI) m/z: $[M + H]^+$ calcd. for $C_{19}H_{20}NO_2^+$, 294.1489; Found: 294.1486. $[\alpha]_D^{20}$ = +24.9 (c = 1.0, $CHCl_3$).

3-Phenyl-8,8a,9,10,11,12-hexahydro-7H-benzo[g]pyrido[2,1-d][1,5]oxazonin-5(14H)-one (5f)

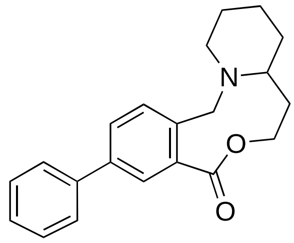

3-Bromo-8,8a,9,10,11,12-hexahydro-7H-benzo[g]pyrido[2,1-d][1,5]oxazonin-5(14H)-one **4f** (100 mg, 0.308 mmol) and phenylboronic acid (75.2 mg, 0.617 mmol) were dissolved in 1,4-dioxane (5.0 mL). Then Na_2CO_3 (98.1 mg, 0.925 mmol) which dissolved in water (2.5 mL) was added followed by $Pd(dppf)Cl_2 \cdot CH_2Cl_2$ (12.5 mg, 0.015 mmol) and the resulting mixture was purged with argon for 10 minutes. The mixture was stirred and heated at 50 °C for 16 hours under argon. After being allowed to cool to RT, water (20 mL) was added and the two layers were separated. The aqueous layer was extracted with dichloromethane (3 x 20 mL) and the combined organic phases were dried with magnesium sulphate, filtered and concentrated in vacuo to give the crude product. The product was purified by column chromatography (SiO_2, 20:1 → 10:1 hexane : ethyl acetate) to afford the title compound (90.2 mg, 91%) as a white solid. R_f = 0.32 (10:1 hexane : ethyl acetate); m.p. 185–191 °C; ν_{max}/cm^{-1} (thin film) 1717, 1587, 1433, 1260, 1118, 1094, 869, 779, 704; δ_H (400 MHz, $CDCl_3$) 7.81 (d, 1H, *J* = 2.0 Hz), 7.62 – 7.59 (m, 2H), 7.56 (dd, 1H, *J* = 8.0, 2.0 Hz), 7.43 (t, 2H, *J* = 7.5 Hz), 7.34 (t, 1H, *J* = 7.5 Hz), 7.26 (d, 1H, *J* = 8.0 Hz), 5.33 (t, 1H, *J* = 11.5 Hz), 4.69 (d, 1H, *J* = 14.0 Hz), 4.04 (dt, 1H, *J* = 11.5, 3.0 Hz), 2.81 (d, 1H, *J* = 14.0 Hz), 2.76 – 2.62 (m, 3H), 1.94 (td, 1H, *J* = 11.5, 3.0 Hz), 1.74 – 1.63 (m, 2H), 1.49 – 1.24 (m, 5H); $^{13}C\{^1H\}$ NMR (100 MHz, $CDCl_3$) 171.9, 142.0, 140.2, 134.0, 129.4, 128.9, 128.9, 128.6, 127.6, 127.4, 127.1, 63.2, 58.4, 57.0, 53.6, 32.1, 31.2, 25.8, 24.6; HRMS (ESI) m/z: $[M + H]^+$ calcd. for $C_{21}H_{24}NO_2^+$, 322.1802; Found: 322.1798.

tert-Butyl 4-(5-bromo-2-(methoxycarbonyl)benzyl)-3-(hydroxymethyl)piperazine-1-carboxylate (8g)

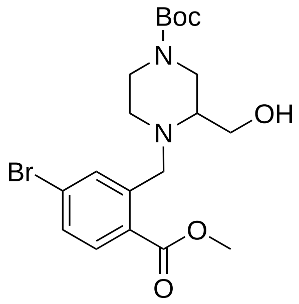

To a stirring solution of potassium carbonate (2.69 g, 19.5 mmol) in acetonitrile (40.0 mL), *tert*-Butyl 3-(hydroxymethyl)piperazine-1-carboxylate (1.40 g, 6.49 mmol) was added followed by methyl 4-bromo-2-(bromomethyl)benzoate **7c** (2.00 g, 6.49 mmol). The reaction mixture was refluxed at 90 °C for 17 hours under argon. Then the reaction mixture was allowed to cool to RT, filtered and washed with dichloromethane, concentrated under vacuum and purified by column chromatography (SiO_2, 5:1 → 2:1 hexane : ethyl acetate) to afford the title compound (2.49 g, 87%) as a colourless oil. R_f = 0.33 (2:1 hexane : ethyl acetate); ν_{max}/cm^{-1} (thin film) 2976, 1720, 1673, 1588, 1431, 1366, 1272, 1169, 1125, 1091, 865, 770, 730; δ_H (400 MHz, $CDCl_3$) 7.57 – 7.43 (m, 2H), 7.34 (dd, 1H, *J* = 8.0, 2.5 Hz), 4.35 (s, 1H), 3.79–3.75 (m, 4H), 3.58 – 3.26 (m, 4H), 3.04 (s, 2H), 2.52 – 2.40 (m, 1H), 2.34 (s, 1H), 2.01 (ddd, 1H, *J* = 11.5, 8.5, 3.0 Hz), 1.34 (s, 9H); $^{13}C\{^1H\}$ NMR (100 MHz, $CDCl_3$) 168.4, 154.9, 141.8, 133.1, 131.3, 130.2, 129.5, 125.9, 79.7, 61.1, 59.5, 55.7, 52.3, 49.5, 44.2, 42.3, 28.3; HRMS (ESI) m/z: $[M + H]^+$ calcd. for $C_{19}H_{28}{}^{79}BrN_2O_5^+$, 443.1176; Found: 443.1162.

tert-Butyl 10-bromo-7-oxo-1,2,4a,5,7,12-hexahydrobenzo[f]pyrazino[2,1-c][1,4] oxazocine-3(4H)-carboxylate (4i)

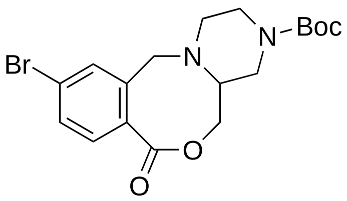

To a stirring solution of methyl *tert*-Butyl 4-(5-bromo-2-(methoxycarbonyl)benzyl)-3-(hydroxymethyl)piperazine-1-carboxylate **8g** (2.20 g, 4.96 mmol) in methanol (10.9 mL), aqueous lithium hydroxide (0.5 M) was added (10.9 mL, 5.46 mmol) and the reaction mixture was heated to 50 °C for 29.5 hours. The reaction was then allowed to cool to RT, and the solvent was removed under vacuum using chloroform (5 × 200 mL) to form an azeotropic mixture. The intermediate 4-Bromo-2-((4-(*tert*-butoxycarbonyl)-2-(hydroxymethyl)piperazin-1-yl)methyl)benzoic acid was dissolved in chloroform (60 mL) and DIPEA (1.60 mL, 9.18 mmol) was added followed by T3P (50% w/v in ethyl acetate, 4.74 g, 7.44 mmol) and the mixture was stirred at room temperature for 15.5 hours under argon. The reaction mixture was then transferred into a separating funnel and water (50 mL) was added, and extracted with dichloromethane (3 × 50 mL). The organic phases were then combined, dried with magnesium

sulphate, filtered and concentrated in vacuo. The crude product was purified by column chromatography (SiO_2, 2:1 hexane : ethyl acetate) to afford the title compound (1.90 g, 93%) as a yellow solid. R_f = 0.53 (2:1 hexane : ethyl acetate); m.p. 99–103 °C; ν_{max}/cm^{-1} (thin film) 2976, 1724, 1688, 1588, 1456, 1271, 1241, 1168, 1114, 1090, 1012, 734; δ_H (400 MHz, $CDCl_3$) 7.42 (dt, 1H, *J* = 8.0, 1.5 Hz), 7.33 (d, 1H, *J* = 1.5 Hz), 7.30 (dd, 1H, *J* = 8.0, 1.5 Hz), 4.08 – 4.03 (m, 1H), 4.02 – 3.75 (m, 3H), 3.74–3.66 (m, 2H), 3.05 – 2.73 (m, 3H), 2.60 – 2.43 (m, 2H), 1.40 (9H, d, *J* = 1.3 Hz); $^{13}C\{^1H\}$ NMR (100 MHz, $CDCl_3$) 171.0, 154.5, 140.1, 131.7, 131.1, 131.0, 127.8, 125.5, 80.1, 68.5, 62.5, 59.8, 54.8, 44.7, 42.9, 28.4 ; HRMS (ESI) m/z: $[M + Na]^+$ calcd. for $C_{18}H_{23}{}^{79}BrN_2NaO_4^+$, 411.0914; Found: 411.0912.

***tert*-Butyl 7-oxo-10-(4,4,5,5-tetramethyl-1,3,2-dioxaborolan-2-yl)-1,2,4a,5,7,12-hexahydrobenzo[f]pyrazino[2,1-c][1,4]oxazocine-3(4H)-carboxylate (4j)**

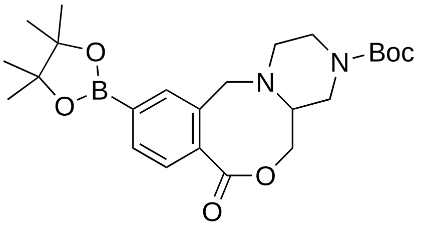

tert-Butyl 10-bromo-7-oxo-1,2,4a,5,7,12-hexahydrobenzo[f]pyrazino[2,1-c][1,4] oxazocine-3(4H)-carboxylate **4i** (1.00 g, 2.43 mmol), bis(pinacolato)diboron (1.23 mg, 4.86 mmol), Pd(dppf)Cl_2 (88.9 mg, 0.122 mmol) and potassium acetate (716 mg, 7.29 mmol) were added to a round-bottomed flask. The round-bottomed flask was purged with argon and then 1,4-dioxane (20 mL) was added. The resulting mixture was stirred and heated at 60 °C for 19 hours under argon. After being allowed to cool to rt, the solids were removed by filtration and washed with dichloromethane (30 mL). The solvents were evaporated under reduced pressure to give the crude product. The crude product was purified by column chromatography (SiO_2, 4:1 hexane : ethyl acetate) to afford the title compound (959 mg, 86%) as a white solid. R_f = 0.50 (2:1 hexane : ethyl acetate); m.p. 162–165 °C; ν_{max}/cm^{-1} (thin film) 2977, 1731, 1695, 1391, 1361, 1275, 1243, 1169, 1114, 1013, 965, 732; δ_H (400 MHz, $CDCl_3$) 7.73 (d, 1H, *J* = 7.5 Hz), 7.63 (s, 1H), 7.45 (d, 1H, *J* = 7.5 Hz), 4.07 (d, 1H, *J* = 12.5 Hz), 4.01 – 3.67 (m, 5H), 3.04 – 2.74 (m, 3H), 2.54 (td, 2H, *J* = 12.0, 4.0 Hz), 1.43 (s, 9H), 1.32 (s, 12H); $^{13}C\{^1H\}$ NMR (100 MHz, $CDCl_3$) 168.8, 151.4, 133.9, 131.7, 130.8, 129.2, 128.2, 126.1, 81.2, 77.0, 65.6, 59.6, 57.2, 51.8, 41.7, 40.8, 25.3, 21.8; HRMS (ESI) m/z: $[M + H]^+$ calcd. for $C_{24}H_{36}BN_2O_6^+$, 459.2661; Found: 459.2678.

tert-Butyl 7-oxo-10-phenyl-1,2,4a,5,7,12-hexahydrobenzo[f]pyrazino[2,1-c][1,4] oxazocine-3(4H)-carboxylate (5g)

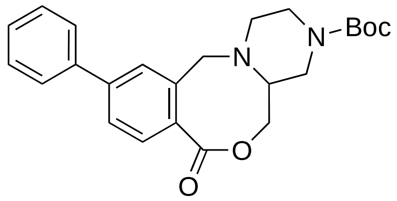

Method 1: *tert*-Butyl 10-bromo-7-oxo-1,2,4a,5,7,12-hexahydrobenzo[f]pyrazino[2,1-c][1,4] oxazocine-3 (4H)-carboxylate **4i** (80.0 mg, 0.194 mmol) and phenylboronic acid (49.4 mg, 0.389 mmol) were dissolved in 1,4-dioxane (4.0 mL). Then Na_2CO_3 (61.8 mg, 0.584 mmol) which dissolved in water (2.0 mL) was added followed by $Pd(dppf)Cl_2{\cdot}CH_2Cl_2$ (7.9 mg, 0.010 mmol) and the resulting mixture was purged with argon for 10 minutes. The mixture was stirred and heated at 50 °C for 17 hours under argon. After being allowed to cool to RT, water (10 mL) was added and the two layers were separated. The aqueous layer was extracted with dichloromethane (3 x 10 mL) and the combined organic phases were dried with magnesium sulphate, filtered and concentrated in vacuo to give the crude product. The product was purified by column chromatography (SiO_2, 5:1 → 3:1 hexane : ethyl acetate) to afford the title compound (65.0 mg, 82%) as a white solid.

Method 2: *tert*-Butyl 7-oxo-10-(4,4,5,5-tetramethyl-1,3,2-dioxaborolan-2-yl)-1,2,4a,5,7,12-hexahydro benzo[f]pyrazino[2,1-c][1,4]oxazocine-3(4H)-carboxylate **4j** (90.0 mg, 0.196 mmol) and bromobenzene (61.7 mg, 0.393 mmol) were dissolved in 1,4-dioxane (4.0 mL). Then Na_2CO_3 (62.4 mg, 0.589 mmol) which dissolved in water (2.0 mL) was added followed by $Pd(dppf)Cl_2{\cdot}CH_2Cl_2$ (8.0 mg, 0.010 mmol) and the resulting mixture was purged with argon for 10 minutes. The mixture was stirred and heated at 50 °C for 18 hours under argon. After being allowed to cool to RT, water (10 mL) was added and the two layers were separated. The aqueous layer was extracted with dichloromethane (3 x 10 mL) and the combined organic phases were dried with magnesium sulphate, filtered and concentrated in vacuo to give the crude product. The product was purified by column chromatography (SiO_2, 5:1 → 3:1 hexane : ethyl acetate) to afford the title compound (52.7 mg, 66%) as a white solid.

Data for **5g**: R_f = 0.56 (2:1 hexane : ethyl acetate); m.p. 155–158 °C; ν_{max}/cm^{-1} (thin film) 2975, 2928, 1720, 1688, 1424, 1271, 1241, 1169, 1121, 1089, 755, 735, 698; δ_H (400 MHz, $CDCl_3$) 7.66 – 7.52 (m, 4H), 7.49 – 7.34 (m, 4H), 4.16 (d, 1H, *J* = 13.0 Hz), 4.05 (dd, 1H, *J* = 13.0, 4.0 Hz), 4.00 – 3.66 (m, 4H), 3.02 – 2.85 (m, 3H), 2.59 (td, 2H, *J* = 11.5, 3.0 Hz), 1.46 (s, 9H); $^{13}C\{^1H\}$ NMR (100 MHz, $CDCl_3$) 172.0, 154.6, 144.2, 139.6, 138.6, 130.8, 129.0, 128.2, 127.7, 127.2, 127.1, 126.5, 80.2, 68.7, 62.7, 60.6, 55.0, 45.9, 43.9, 28.4 ; HRMS (ESI) m/z: $[M + H]^+$ calcd. for $C_{24}H_{29}N_2O_4^+$, 409.2122; Found: 409.2127.

***tert*-Butyl 7-oxo-10-(pyridin-3-yl)-1,2,4a,5,7,12-hexahydrobenzo[f]pyrazino[2,1-c][1,4]oxazocine-3(4H)-carboxylate (5h)**

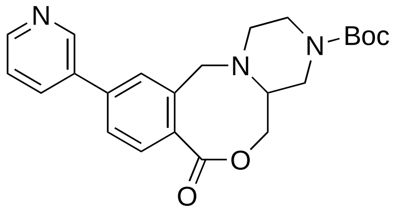

Method 1: *tert*-Butyl 10-bromo-7-oxo-1,2,4a,5,7,12-hexahydrobenzo[f]pyrazino[2,1-c][1,4]oxazocine-3(4H)-carboxylate **4i** (80.0 mg, 0.194 mmol) and pyridin-3-ylboronic acid (47.8 mg, 0.389 mmol) were dissolved in 1,4-dioxane (4.0 mL). Then Na_2CO_3 (61.8 mg, 0.584 mmol) which dissolved in water (2.0 mL) was added followed by $Pd(dppf)Cl_2 \cdot CH_2Cl_2$ (7.9 mg, 0.010 mmol) and the resulting mixture was purged with argon for 10 minutes. The mixture was stirred and heated at 50 °C for 18 hours under argon. After being allowed to cool to RT, water (10 mL) was added and the two layers were separated. The aqueous layer was extracted with ethyl acetate (3 x 10 mL) and the combined organic phases were dried with magnesium sulphate, filtered and concentrated in vacuo to give the crude product. The product was purified by column chromatography (SiO_2, 2:1 → 0:1 hexane : ethyl acetate) to afford the title compound (32.0 mg, 40%) as a yellow solid.

Method 2: *tert*-Butyl 7-oxo-10-(4,4,5,5-tetramethyl-1,3,2-dioxaborolan-2-yl)-1,2,4a,5,7,12-hexahydro benzo[f]pyrazino[2,1-c][1,4]oxazocine-3(4H)-carboxylate **4j** (140 mg, 0.305 mmol) and 3-bromopyridine (96.5 mg, 0.611 mmol) were dissolved in 1,4-dioxane (6.0 mL). Then Na_2CO_3 (97.1 mg, 0.916 mmol) which dissolved in water (3.0 mL) was added followed by $Pd(dppf)Cl_2 \cdot CH_2Cl_2$ (12.2 mg, 0.015 mmol) and the resulting mixture was purged with argon for 10 minutes. The mixture was stirred and heated at 50 °C for 18 hours under argon. After being allowed to cool to RT, water (10 mL) was added and the two layers were separated. The aqueous layer was extracted with dichloromethane (3 x 10 mL) and the combined organic phases were dried with magnesium sulphate, filtered and concentrated in vacuo to give the crude product. The product was purified by column chromatography (SiO_2, 1:1 → 0:1 hexane : ethyl acetate) to afford the title compound (31.1 mg, 25%) as a yellow solid.

Data for **5h**: R_f = 0.30 (ethyl acetate); m.p. 131–135 °C; ν_{max}/cm^{-1} (thin film) 2976, 1721, 1692, 1611, 1456, 1426, 1280, 1243, 1170, 1124, 1021, 729; δ_H (400 MHz, $CDCl_3$) 8.83 (d, 1H, *J* = 2.0 Hz), 8.62 (dd, 1H, *J* = 5.0, 1.5 Hz), 7.87 (dt, 1H, *J* = 8.0, 2.0 Hz), 7.60 (d, 1H, *J* = 8.0 Hz), 7.55 (dd, 1H, *J* = 8.0, 2.0 Hz), 7.41 (d, 1H, *J* = 1.5 Hz), 7.38 (dd, 1H, *J* = 8.0, 5.0 Hz), 4.16 (dd, 1H, *J* = 13.0, 2.0 Hz), 4.05 (dd, 1H, *J* = 13.0, 4.0 Hz), 4.01 – 3.74 (m, 4H), 2.94–2.86 (m, 3H), 2.67 – 2.53 (m, 2H), 1.45 (s, 9H); $^{13}C\{^1H\}$ NMR (100 MHz, $CDCl_3$) 171.7, 154.6, 149.4, 148.3, 140.8, 139.1, 135.3, 134.6, 131.2, 128.7, 127.0, 126.6, 123.8, 80.3,, 68.7, 62.8, 60.6, 55.0, 45.5, 44.0, 28.5 ; HRMS (ESI) m/z: $[M + H]^+$ calcd. for $C_{24}H_{28}N_3O_4^+$, 410.2074; Found: 410.2082.

tert-Butyl 10-(1H-indol-5-yl)-7-oxo-1,2,4a,5,7,12-hexahydrobenzo[f]pyrazino[2,1-c][1,4]oxazocine-3(4H)-carboxylate (5i)

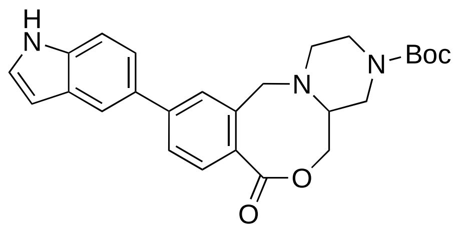

tert-Butyl 10-bromo-7-oxo-1,2,4a,5,7,12-hexahydrobenzo[f]pyrazino[2,1-c][1,4]oxazocine-3(4H)-carboxylate **4i** (80.0 mg, 0.194 mmol) and (1H-indol-5-yl)boronic acid (62.6 mg, 0.389 mmol) were dissolved in 1,4-dioxane (4.0 mL). Then Na_2CO_3 (61.8 mg, 0.584 mmol) which dissolved in water (2.0 mL) was added followed by Pd(dppf)$Cl_2 \cdot CH_2Cl_2$ (7.9 mg, 0.010 mmol) and the resulting mixture was purged with argon for 10 minutes. The mixture was stirred and heated at 50 °C for 18 hours under argon. After being allowed to cool to RT, water (10 mL) was added and the two layers were separated. The aqueous layer was extracted with ethyl acetate (3 x 10 mL) and the combined organic phases were dried with magnesium sulphate, filtered and concentrated in vacuo to give the crude product. The product was purified by column chromatography (SiO_2, 3:1 → 1:1 hexane : ethyl acetate) to afford the title compound (73.1 mg, 84%) as a white solid. R_f = 0.29 (1:1 hexane : ethyl acetate); m.p. 202-206 °C; ν_{max}/cm^{-1} (thin film) 3320, 2977, 1688, 1606, 1456, 1427, 1366, 1282, 1244, 1169, 1123, 1091, 1011, 910, 729; δ_H (400 MHz, $CDCl_3$) 8.71 (s, 1H, N**H**), 7.87 (d, 1H, *J* = 1.5 Hz), 7.62 (dd, 1H, *J* = 8.0, 1.5 Hz), 7.57 (d, 1H, *J* = 8.0 Hz), 7.50 – 7.39 (m, 3H), 7.25 (t, 1H, *J* = 2.5 Hz), 6.60 (t, 1H, *J* = 2.5 Hz), 4.18 (dd, 1H, *J* = 13.0, 2.0 Hz), 4.07 (dd, 1H, *J* = 13.0, 4.5 Hz), 4.03 – 3.71 (m, 4H), 3.10 – 2.79 (m, 3H), 2.67 – 2.53 (m, 2H), 1.48 (s, 9H); $^{13}C\{^1H\}$ NMR (100 MHz, $CDCl_3$) 172.5, 154.7, 145.7, 138.4, 135.9, 131.4, 130.8, 128.5, 127.2, 126.7, 126.6, 125.5, 121.5, 119.5, 111.7, 102.9, 80.3, 68.8, 62.8, 60.7, 54.9, 45.6, 44.1, 28.5; HRMS (ESI) m/z: $[M + H]^+$ calcd. for $C_{26}H_{30}N_3O_4^+$, 448.2231; Found: 448.2238.

tert-Butyl 10-(1-methyl-1H-pyrazol-5-yl)-7-oxo-1,2,4a,5,7,12-hexahydrobenzo[f]pyrazino[2,1-c][1,4]oxazocine-3(4H)-carboxylate (5j)

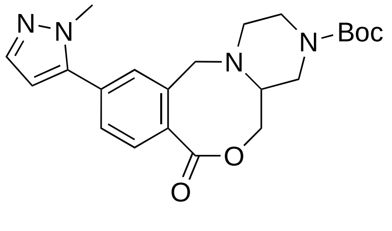

tert-Butyl 10-bromo-7-oxo-1,2,4a,5,7,12-hexahydrobenzo[f]pyrazino[2,1-c][1,4]oxazocine-3(4H)-carboxylate **4i** (80.0 mg, 0.194 mmol) and (1-methyl-1H-pyrazol-5-yl)boronic acid (49.0 mg, 0.389 mmol) were dissolved in 1,4-dioxane (4.0 mL). Then Na_2CO_3 (61.8 mg, 0.584 mmol) which dissolved in water (2.0 mL) was added followed by Pd(dppf)$Cl_2 \cdot CH_2Cl_2$ (7.9 mg, 0.010 mmol) and the resulting mixture was purged with argon for 10 minutes. The mixture was stirred and heated at 50 °C for 18 hours under argon. After being allowed to cool to RT, water (10 mL) was added and the two layers were separated. The aqueous layer was extracted with ethyl acetate (3 x 10 mL) and the combined organic phases were dried with magnesium

sulphate, filtered and concentrated in vacuo to give the crude product. The product was purified by column chromatography (SiO_2, 2:1 → 0:1 hexane : ethyl acetate) to afford the title compound (40.2 mg, 50%) as a yellow solid. R_f = 0.51 (ethyl acetate); m.p. 147–152 °C; $ν_{max}/cm^{-1}$ (thin film) 2924, 1721, 1690, 1458, 1425, 1366, 1280, 1244, 1171, 1123, 1091, 1013, 761; $δ_H$ (400 MHz, $CDCl_3$) 7.59 (d, 1H, *J* = 8.0 Hz), 7.53 (d, 1H, *J* = 2.0 Hz), 7.41 (dd, 1H, *J* = 8.0, 2.0 Hz), 7.27 (d, 1H, *J* = 2.0 Hz), 6.35 (d, 1H, *J* = 2.0 Hz), 4.18 (dd, 1H, *J* = 13.0, 2.0 Hz), 4.06 (dd, 1H, *J* = 13.0, 4.0 Hz), 3.97 – 3.79 (m, 7H), 3.01 – 2.84 (m, 3H), 2.67 – 2.54 (m, 2H), 1.46 (s, 9H); $^{13}C\{^1H\}$ NMR (100 MHz, $CDCl_3$) 171.6, 154.7, 142.2, 138.9, 133.7, 132.0, 130.8, 129.0, 128.5, 127.9, 106.7, 80.4, 68.6, 62.8, 60.5, 55.0, 44.7, 43.0, 37.8, 28.5; HRMS (ESI) m/z: $[M + H]^+$ calcd. for $C_{26}H_{31}N_4O_4^+$, 463.2340; Found: 463.2356.

tert-Butyl 10-(4-fluoro-2-methoxyphenyl)-7-oxo-1,2,4a,5,7,12-hexahydrobenzo[f] pyrazino[2,1-c][1,4]oxazocine-3(4H)-carboxylate (5k)

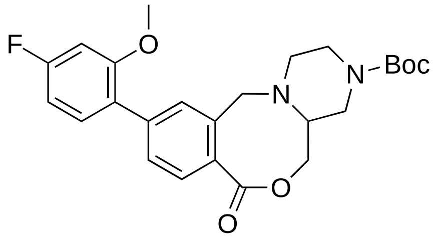

tert-Butyl 10-bromo-7-oxo-1,2,4a,5,7,12-hexahydrobenzo[f]pyrazino[2,1-c][1,4]oxazocine-3(4H)-carboxylate **4i** (120 mg, 0.292 mmol) and (4-fluoro-2-methoxyphenyl)boronic acid (99.3 mg, 0.584 mmol) were dissolved in 1,4-dioxane (6.0 mL). Then Na_2CO_3 (92.7 mg, 0.875 mmol) which dissolved in water (3.0 mL) was added followed by $Pd(dppf)Cl_2{\cdot}CH_2Cl_2$ (12.2 mg, 0.015 mmol) and the resulting mixture was purged with argon for 10 minutes. The mixture was stirred and heated at 50 °C for 18 hours under argon. After being allowed to cool to RT, water (10 mL) was added and the two layers were separated. The aqueous layer was extracted with ethyl acetate (3 x 10 mL) and the combined organic phases were dried with magnesium sulphate, filtered and concentrated in vacuo to give the crude product. The product was purified by column chromatography (SiO_2, 5:1 → 2:1 hexane : ethyl acetate) to afford the title compound (116 mg, 87%) as a white solid. R_f = 0.40 (2:1 hexane : ethyl acetate); m.p. 109–113 °C; $ν_{max}/cm^{-1}$ (thin film) 2975, 1721, 1692, 1607, 1455, 1424, 1281, 1241, 1170, 1155, 1090, 1032, 955, 836, 768, 735; $δ_H$ (400 MHz, $CDCl_3$) 7.48 (d, 1H, *J* = 8.0 Hz), 7.43 (dd, 1H, *J* = 8.0, 1.5 Hz), 7.28 (s, 1H), 7.21 (dd, 1H, *J* = 8.5, 6.5 Hz), 6.74 – 6.64 (m, 2H), 4.16 (dd, 1H, *J* = 13.0, 2.0 Hz), 4.03 (dd, 1H, *J* = 13.0, 4.0 Hz), 3.98 – 3.72 (m, 7H), 3.05 – 2.77 (m, 3H), 2.55 (td, 2H, *J* = 11.5, 3.0 Hz), 1.43 (s, 9H); $^{13}C\{^1H\}$ NMR (100 MHz, $CDCl_3$) 172.0, 163.4 ($J_{F\text{-}C}$ = 246 Hz), 157.6 ($J_{F\text{-}C}$ = 9.8 Hz), 154.5, 140.7, 137.8, 131.4 ($J_{F\text{-}C}$ = 9.7 Hz), 129.9, 129.1, 128.9, 127.3, 125.0 ($J_{F\text{-}C}$ = 3.1 Hz), 107.3 ($J_{F\text{-}C}$ = 21.0 Hz), 99.5 ($J_{F\text{-}C}$ = 25.6 Hz), 80.0, 68.5, 62.6, 60.5, 55.8, 54.7, 44.8, 43.8, 28.4; HRMS (ESI) m/z: $[M + H]^+$ calcd. for $C_{25}H_{30}FN_2O_5^+$, 457.2133; Found: 457.2130.

6-(4,4,5,5-Tetramethyl-1,3,2-dioxaborolan-2-yl)benzo[d]thiazole (S1)

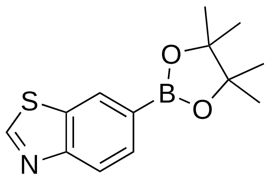

6-Bromobenzo[d]thiazole (800 mg, 3.74 mmol), bis(pinacolato)diboron (1.04 g, 4.11 mmol), Pd(dppf)Cl_2 (55.0 mg, 0.075 mmol) and potassium acetate (1.10 g, 11.2 mmol) were added to a round-bottomed flask. The round-bottomed flask was purged with argon and then 1,4-dioxane (20 mL) was added. The resulting mixture was stirred and heated at 60 °C for 18 hours under argon. After being allowed to cool to RT, the solids were removed by filtration and washed with dichloromethane (10 mL). The solvents were evaporated under reduced pressure to give the crude product. The crude product was purified by column chromatography (SiO_2, 10:1 hexane : ethyl acetate) to afford the title compound (898 mg, 92%) as a white solid. R_f = 0.32 (10:1 hexane : ethyl acetate); m.p. 93–95 °C; ν_{max}/cm^{-1} (thin film) 2977, 1596, 1474, 1441, 1386, 1345, 1287, 1143, 1095, 964, 894, 856, 674; δ_H (400 MHz, $CDCl_3$) 9.04 (s, 1H), 8.45 (s, 1H), 8.12 (d, 1H, *J* = 8.0 Hz), 7.93 (dd, 1H, *J* = 8.0, 1.0 Hz), 1.36 (s, 12H); $^{13}C\{^1H\}$ NMR (100 MHz, $CDCl_3$) 155.5, 155.26, 133.44, 132.1, 129.1, 123.0, 84.24, 25.0; HRMS (ESI) m/z: $[M + H]^+$ calcd. for $C_{13}H_{17}BNO_2S^+$, 262.1068; Found: 262.1070. Characterisation data matched those reported in the literature.[4]

Benzo[d]thiazol-6-ylboronic acid (S2)

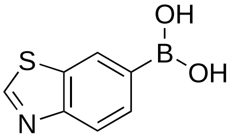

6-(4,4,5,5-Tetramethyl-1,3,2-dioxaborolan-2-yl)benzo[d]thiazole **S1** (400 mg, 1.53 mmol) was dissolved in 6 M HCl aqueous solution (4.0 mL). The solution was heated to reflux at 120 °C for 3 hours, after which the solution was concentrated in vacuo to give the crude product. The product was triturated in DCM (4.0 mL) for 20 mins, filtered and concentrated in vacuo to afford the title compound (314 mg, HCl salt, 95%) as a white solid; m.p. 267–273 °C; ν_{max}/cm^{-1} (thin film) 3347, 2635, 1596, 1397, 1337, 1310, 1191, 1119, 1057, 1035, 841, 768, 691, 668; δ_H (400 MHz, Methanol-*D₄*) 10.24 (s, 1H), 8.55 (s, 1H), 8.07 (s, 2H); δ_C (101 MHz, Methanol-D_4) 163.1, 145.4, 135.1, 132.4, 130.2, 119.0 (Ar**C**H); HRMS (ESI) calcd. for $C_7H_7BNO_2S^+$, 180.0286. Found: $[MH]^+$ 180.0294. Characterisation data matched those reported in the literature.[5]

***tert*-Butyl 10-(benzo[d]thiazol-6-yl)-7-oxo-1,2,4a,5,7,12-hexahydrobenzo[f]pyrazino [2,1-c][1,4]oxazocine-3(4H)-carboxylate (5l)**

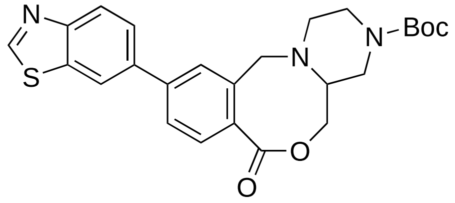

Method 1: *tert*-Butyl 10-bromo-7-oxo-1,2,4a,5,7,12-hexahydrobenzo[f]pyrazino[2,1-c][1,4]oxazocine-3(4H)-carboxylate **4i** (90.0 mg, 0.219 mmol) and benzo[d]thiazol-6-ylboronic acid **S2** (HCl salt, 70.7 mg, 0.328 mmol) were dissolved in 1,4-dioxane (2.0 mL). Then Na_2CO_3 (69.5 mg, 0.656 mmol) which dissolved in water (1.0 mL) was added followed by $Pd(dppf)Cl_2 \cdot CH_2Cl_2$ (8.9 mg, 0.011 mmol) and the resulting mixture was purged with argon for 10 minutes. The mixture was stirred and heated at 50 °C for 18 hours under argon. After being allowed to cool to RT, water (10 mL) was added and the two layers were separated. The aqueous layer was extracted with ethyl acetate (3 x 10 mL) and the combined organic phases were dried with magnesium sulphate, filtered and concentrated in vacuo to give the crude product. The product was purified by column chromatography (SiO_2, 3:1 → 1:1 hexane : ethyl acetate) to afford the title compound (93.6 mg, 94%) as a yellow solid.

Method 2: *tert*-Butyl 10-bromo-7-oxo-1,2,4a,5,7,12-hexahydrobenzo[f]pyrazino[2,1-c][1,4]oxazocine-3(4H)-carboxylate **4i** (90.0 mg, 0.219 mmol) and 6-(4,4,5,5-tetramethyl-1,3,2-dioxaborolan-2-yl)benzo[d]thiazole **S1** (114 mg, 0.438 mmol) were dissolved in 1,4-dioxane (2.0 mL). Then Na_2CO_3 (69.5 mg, 0.656 mmol) which dissolved in water (1.0 mL) was added followed by $Pd(dppf)Cl_2 \cdot CH_2Cl_2$ (8.9 mg, 0.011 mmol) and the resulting mixture was purged with argon for 10 minutes. The mixture was stirred and heated at 50 °C for 18 hours under argon. After being allowed to cool to RT, water (10 mL) was added and the two layers were separated. The aqueous layer was extracted with ethyl acetate (3 x 10 mL) and the combined organic phases were dried with magnesium sulphate, filtered and concentrated in vacuo to give the crude product. The product was purified by column chromatography (SiO_2, 3:1 → 1:1 hexane : ethyl acetate) to afford the title compound (98.7 mg, 99%) as a yellow solid.

Method 3: *tert*-Butyl 7-oxo-10-(4,4,5,5-tetramethyl-1,3,2-dioxaborolan-2-yl)-1,2,4a,5,7,12-hexahydro benzo[f]pyrazino[2,1-c][1,4]oxazocine-3(4H)-carboxylate **4j** (90.0 mg, 0.196 mmol) and 6-bromobenzo[d]thiazole (84.1 mg, 0.393 mmol) were dissolved in 1,4-dioxane (4.0 mL). Then Na_2CO_3 (62.4 mg, 0.589 mmol) which dissolved in water (2.0 mL) was added followed by $Pd(dppf)Cl_2 \cdot CH_2Cl_2$ (8.0 mg, 0.010 mmol) and the resulting mixture was purged with argon for 10 minutes. The mixture was stirred and heated at 50 °C for 18 hours under argon. After being allowed to cool to RT, water (10 mL) was added and the two layers were separated. The aqueous layer was extracted with ethyl acetate (3 x 10 mL) and the combined organic phases were dried with magnesium sulphate, filtered and concentrated in vacuo to give the crude product. The product was purified by column chromatography (SiO_2, 3:1 → 1:1 hexane : ethyl acetate) to afford the title compound (58.7 mg, 64%) as a yellow solid.

Data for **5l**: R_f = 0.30 (1:1 hexane : ethyl acetate); m.p. 170–173 °C; v_{max}/cm^{-1} (thin film) 2976, 1718, 1688, 1465, 1427, 1366, 1280, 1242, 1170, 1122, 1090, 1011, 868, 836, 732; δ_H (400 MHz, $CDCl_3$) 9.01 (s, 1H), 8.23 – 8.09 (m, 2H), 7.71 (dt, 1H, *J* = 8.5, 2.5 Hz), 7.58 (d, 2H, *J* = 3.0 Hz), 7.46 (s, 1H), 4.15 (d, 1H, *J* = 13.0 Hz), 4.05 (dd, 1H, *J* = 13.0, 4.0 Hz), 4.02 – 3.72 (m, 4H), 3.08 – 2.79 (m, 3H), 2.58 (td, 2H, *J* = 10.0, 6.0 Hz), 1.44 (s, 9H); $^{13}C\{^1H\}$ NMR (100 MHz, $CDCl_3$) 171.8, 154.8, 154.5, 153.0, 143.4, 138.8, 137.3, 134.7, 131.0, 128.1, 127.4, 126.8, 125.8, 123.9, 120.4, 80.2,, 68.7, 62.7, 60.5, 55.0, 45.7, 43.9, 28.4 ; HRMS (ESI) m/z: $[M + H]^+$ calcd. for $C_{25}H_{28}N_3O_4S^+$, 466.1795; Found: 466.1830.

***tert*-Butyl 10-(isoquinolin-6-yl)-7-oxo-1,2,4a,5,7,12-hexahydrobenzo[f]pyrazino[2,1-c][1,4]oxazocine-3(4H)-carboxylate (5m)**

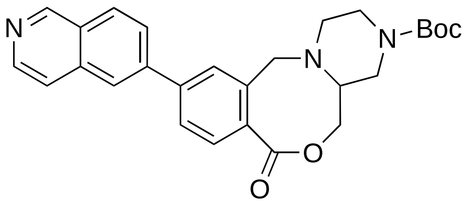

tert-Butyl 7-oxo-10-(4,4,5,5-tetramethyl-1,3,2-dioxaborolan-2-yl)-1,2,4a,5,7,12-hexahydro benzo[f]pyrazino[2,1-c][1,4]oxazocine-3(4H)-carboxylate **4j** (90.0 mg, 0.196 mmol) and 6-bromoisoquinoline (81.8 mg, 0.393 mmol) were dissolved in 1,4-dioxane (4.0 mL). Then Na_2CO_3 (62.4 mg, 0.589 mmol) which dissolved in water (2.0 mL) was added followed by $Pd(dppf)Cl_2{\cdot}CH_2Cl_2$ (8.0 mg, 0.010 mmol) and the resulting mixture was purged with argon for 10 minutes. The mixture was stirred and heated at 50 °C for 18 hours under argon. After being allowed to cool to RT, water (10 mL) was added and the two layers were separated. The aqueous layer was extracted with ethyl acetate (3 x 10 mL) and the combined organic phases were dried with magnesium sulphate, filtered and concentrated in vacuo to give the crude product. The product was purified by column chromatography (SiO_2, 2:1 → 0:1 hexane : ethyl acetate) to afford the title compound (58.0 mg, 64%) as a yellow solid. R_f = 0.34 (ethyl acetate); m.p. 216–219 °C; v_{max}/cm^{-1} (thin film) 2976, 1718, 1687, 1421, 1279, 1243, 1169, 1122, 1090, 911, 829, 728; δ_H (400 MHz, $CDCl_3$) δ 9.31 (s, 1H), 8.58 (d, 1H, *J* = 5.5 Hz), 8.08 (d, 1H, *J* = 8.5 Hz), 8.02 (d, 1H, *J* = 1.5 Hz), 7.85 (dd, 1H, *J* = 8.5, 1.5 Hz), 7.75 – 7.64 (m, 3H), 7.56 (d, 1H, *J* = 1.5 Hz), 4.20 (dd, 1H, *J* = 13.0, 2.0 Hz), 4.09 (dd, 1H, *J* = 13.0, 4.0 Hz), 4.04 – 3.72 (m, 4H), 3.11 – 2.82 (m, 3H), 2.70 – 2.55 (m, 2H), 1.47 (s, 9H); $^{13}C\{^1H\}$ NMR (100 MHz, $CDCl_3$) 171.7, 154.6, 152.4, 143.8, 141.5, 139.0, 136.0, 131.1, 128.7, 128.5, 128.0, 127.5, 127.0, 126.8, 124.8, 120.7, 80.3, 68.7, 62.8, 60.6, 55.0, 45.6, 44.0, 28.5; HRMS (ESI) m/z: $[M + H]^+$ calcd. for $C_{27}H_{30}N_3O_4^+$, 460.2231; Found: 460.2233.

tert-Butyl 7-oxo-10-(quinoxalin-6-yl)-1,2,4a,5,7,12-hexahydrobenzo[f]pyrazino[2,1-c][1,4]oxazocine-3(4H)-carboxylate (5n)

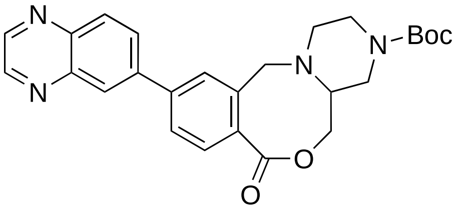

tert-Butyl 7-oxo-10-(4,4,5,5-tetramethyl-1,3,2-dioxaborolan-2-yl)-1,2,4a,5,7,12-hexahydro benzo[f]pyrazino[2,1-c][1,4]oxazocine-3(4H)-carboxylate **4j** (90.0 mg, 0.196 mmol) and 6-bromoquinoxaline (82.2 mg, 0.393 mmol) were dissolved in 1,4-dioxane (4.0 mL). Then Na_2CO_3 (62.4 mg, 0.589 mmol) which dissolved in water (2.0 mL) was added followed by $Pd(dppf)Cl_2{\cdot}CH_2Cl_2$ (8.0 mg, 0.010 mmol) and the resulting mixture was purged with argon for 10 minutes. The mixture was stirred and heated at 50 °C for 18 hours under argon. After being allowed to cool to RT, water (10 mL) was added and the two layers were separated. The aqueous layer was extracted with ethyl acetate (3 x 10 mL) and the combined organic phases were dried with magnesium sulphate, filtered and concentrated in vacuo to give the crude product. The product was purified by column chromatography (SiO_2, 2:1 → 0:1 hexane : ethyl acetate) to afford the title compound (56.2 mg, 62%) as a yellow solid. R_f = 0.56 (ethyl acetate); m.p. 155–159 °C; v_{max}/cm^{-1} (thin film) 2975, 1720, 1687, 1424, 1366, 1279, 1241, 1167, 1121, 1089, 1021, 866, 837, 764, 732; δ_H (400 MHz, $CDCl_3$) 8.89 – 8.78 (m, 2H), 8.28 (d, 1H, *J* = 2.0 Hz), 8.15 (d, 1H, *J* = 8.5 Hz), 7.99 (dd, 1H, *J* = 8.5, 2.0 Hz), 7.69 (dd, 1H, *J* = 8.0, 2.0 Hz), 7.61 (d, 1H, *J* = 8.0 Hz), 7.57 (d, 1H, *J* = 2.0 Hz), 4.17 (dd, 1H, *J* = 13.0, 2.0 Hz), 4.06 (dd, 1H, *J* = 13.0, 4.0 Hz), 4.02 – 3.74 (m, 4H), 3.01 – 2.86 (m, 3H), 2.60 (td, 2H, *J* = 11.5, 3.0 Hz), 1.43 (s, 9H); $^{13}C\{^1H\}$ NMR (100 MHz, $CDCl_3$) 171.6, 154.6, 145.7, 145.3, 143.1, 142.7, 142.4, 141.2, 139.0, 131.1, 130.2, 129.4, 128.8, 127.5, 127.5, 126.9, 80.2, 68.8, 62.7, 60.5, 55.0, 45.7, 43.9, 28.4; HRMS (ESI) m/z: $[M + Na]^+$ calcd. for $C_{26}H_{28}N_4NaO_4^+$, 483.2003; Found: 483.2015.

tert-Butyl 10-(1-methyl-1H-benzo[d]imidazol-6-yl)-7-oxo-1,2,4a,5,7,12-hexahydrobenzo[f] pyrazino[2,1-c][1,4]oxazocine-3(4H)-carboxylate (5o)

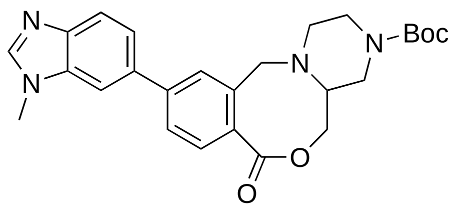

tert-Butyl 7-oxo-10-(4,4,5,5-tetramethyl-1,3,2-dioxaborolan-2-yl)-1,2,4a,5,7,12-hexahydro benzo[f]pyrazino[2,1-c][1,4]oxazocine-3(4H)-carboxylate **4j** (90.0 mg, 0.196 mmol) and 6-bromo-1-methyl-1H-benzo[d]imidazole (82.9 mg, 0.393 mmol) were dissolved in 1,4-dioxane (4.0 mL). Then Na_2CO_3 (62.4 mg, 0.589 mmol) which dissolved in water (2.0 mL) was added followed by $Pd(dppf)Cl_2{\cdot}CH_2Cl_2$ (8.0 mg, 0.010 mmol) and the resulting mixture was purged with argon for 10 minutes. The mixture was stirred and heated at 50 °C for 18 hours under argon. After being allowed to cool to RT, water (10 mL) was added and the two layers were

separated. The aqueous layer was extracted with ethyl acetate (3 x 10 mL) and the combined organic phases were dried with magnesium sulphate, filtered and concentrated in vacuo to give the crude product. The product was purified by column chromatography (SiO_2, 10:1 → 3:1 dichloromethane : acetone) to afford the title compound (34.0 mg, 38%) as a brown solid. R_f = 0.21 (3:1 dichloromethane : acetone); m.p. 184–189 °C; ν_{max}/cm^{-1} (thin film) 3404, 2974, 1690, 1607, 1461, 1424, 1365, 1283, 1242, 1169, 1122, 1090, 1012, 820, 768; δ_H (400 MHz, $CDCl_3$) 7.93 (s, 1H), 7.84 (d, 1H, *J* = 8.0 Hz), 7.60 (dd, 1H, *J* = 8.0, 1.5 Hz), 7.55 (dd, 2H, *J* = 5.0, 3.0 Hz), 7.50 (dd, 1H, *J* = 8.0, 1.5 Hz), 7.45 (d, 1H, *J* = 1.5 Hz), 4.16 (dd, 1H, *J* = 13.0, 2.0 Hz), 4.08 – 4.02 (m, 1H), 4.00 – 3.77 (m, 7H), 2.91 – 2.84 (m, 3H), 2.58 (td, 2H, *J* = 11.5, 3.0 Hz), 1.44 (s, 9H); $^{13}C\{^1H\}$ NMR (100 MHz, $CDCl_3$) 172.0, 154.6, 144.9, 144.6, 143.3, 138.7, 135.3, 135.1, 131.0, 127.6, 127.4, 126.9, 122.3, 120.6, 108.3, 80.3, 68.8, 62.8, 60.7, 55.0, 44.9, 44.1, 31.4, 28.4; HRMS (ESI) m/z: $[M + H]^+$ calcd. for $C_{26}H_{31}N_4O_4^+$, 463.2340; Found: 463.2356.

***tert*-Butyl 10-(3,4-dimethoxyphenyl)-7-oxo-1,2,4a,5,7,12-hexahydrobenzo[f] pyrazino[2,1-c][1,4]oxazocine-3(4H)-carboxylate (5p)**

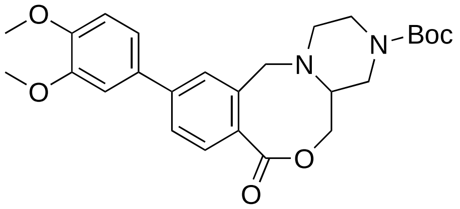

tert-Butyl 7-oxo-10-(4,4,5,5-tetramethyl-1,3,2-dioxaborolan-2-yl)-1,2,4a,5,7,12-hexahydro benzo[f]pyrazino[2,1-c][1,4]oxazocine-3(4H)-carboxylate **4j** (140 mg, 0.305 mmol) and 4-bromo-1,2-dimethoxybenzene (133 mg, 0.611 mmol) were dissolved in 1,4-dioxane (6.0 mL). Then Na_2CO_3 (97.1 mg, 0.916 mmol) which dissolved in water (3.0 mL) was added followed by $Pd(dppf)Cl_2 \cdot CH_2Cl_2$ (12.2 mg, 0.015 mmol) and the resulting mixture was purged with argon for 10 minutes. The mixture was stirred and heated at 50 °C for 18 hours under argon. After being allowed to cool to RT, water (10 mL) was added and the two layers were separated. The aqueous layer was extracted with ethyl acetate (3 x 10 mL) and the combined organic phases were dried with magnesium sulphate, filtered and concentrated in vacuo to give the crude product. The product was purified by column chromatography (SiO_2, 2:1 → 1:1 hexane : ethyl acetate) to afford the title compound (97.2 mg, 68%) as a white solid. R_f = 0.42 (1:1 hexane : ethyl acetate); m.p. 99–103 °C; ν_{max}/cm^{-1} (thin film) 2974, 2836, 1718, 1689, 1606, 1521, 1494, 1462, 1425, 1267, 1250, 1222, 1170, 1122, 1090, 1023, 915, 764, 729; δ_H (400 MHz, $CDCl_3$) 7.57 – 7.43 (m, 2H), 7.34 (s, 1H), 7.12 (dd, 1H, *J* = 8.5, 2.0 Hz), 7.06 (d, 1H, *J* = 2.0 Hz), 6.91 (d, 1H, *J* = 8.5 Hz), 4.13 (dd, 1H, *J* = 13.0, 2.0 Hz), 4.02 (dd, 1H, *J* = 13.0, 4.0 Hz), 3.97 – 3.73 (10H, m), 3.06 – 2.79 (m, 3H), 2.61 – 2.47 (m, 2H), 1.43 (s, 9H); $^{13}C\{^1H\}$ NMR (100 MHz, $CDCl_3$) 171.9, 154.5, 149.3, 149.3, 143.9, 138.5, 132.4, 130.8, 127.2, 126.5, 126.1, 119.7, 111.5, 110.3, 80.1, 68.6, 62.7, 60.5, 56.0, 56.0, 54.9, 44.8, 43.0, 28.4; HRMS (ESI) m/z: $[M + H]^+$ calcd. for $C_{26}H_{33}N_2O_6^+$, 469.2333; Found: 469.2337.

tert-Butyl 10-(5-fluoro-3-methylpyridin-2-yl)-7-oxo-1,2,4a,5,7,12-hexahydrobenzo[f] pyrazino[2,1-c][1,4]oxazocine-3(4H)-carboxylate (5q)

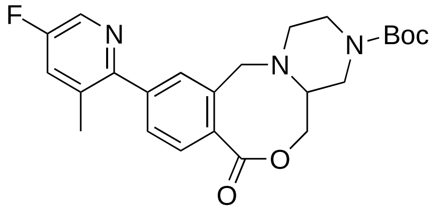

tert-Butyl 7-oxo-10-(4,4,5,5-tetramethyl-1,3,2-dioxaborolan-2-yl)-1,2,4a,5,7,12-hexahydro benzo[f]pyrazino[2,1-c][1,4]oxazocine-3(4H)-carboxylate **4j** (140 mg, 0.305 mmol) and 2-bromo-5-fluoro-3-methylpyridine (116 mg, 0.611 mmol) were dissolved in 1,4-dioxane (6.0 mL). Then Na_2CO_3 (97.1 mg, 0.916 mmol) which dissolved in water (3.0 mL) was added followed by Pd(dppf)Cl_2·CH_2Cl_2 (12.2 mg, 0.015 mmol) and the resulting mixture was purged with argon for 10 minutes. The mixture was stirred and heated at 50 °C for 18 hours under argon. After being allowed to cool to RT, water (10 mL) was added and the two layers were separated. The aqueous layer was extracted with dichloromethane (3 x 10 mL) and the combined organic phases were dried with magnesium sulphate, filtered and concentrated in vacuo to give the crude product. The product was purified by column chromatography (SiO_2, 3:1 → 1:1 hexane : ethyl acetate) to afford the title compound (67.0 mg, 50%) as a white solid. R_f = 0.47 (1:1 hexane : ethyl acetate); m.p. 126–128 °C; ν_{max}/cm^{-1} (thin film) 2975, 2929, 1722, 1691, 1461, 1427, 1366, 1276, 1242, 1171, 1122, 1090, 1011, 886, 735; δ_H (400 MHz, $CDCl_3$) 8.36 (d, 1H, J = 3.0 Hz), 7.54 (d, 1H, J = 8.0 Hz), 7.42 (dd, 1H, J = 8.0, 1.5 Hz), 7.37 – 7.30 (m, 2H), 4.16 (dd, 1H, J = 13.0, 2.0 Hz), 4.01 (dd, 1H, J = 13.0, 4.0 Hz), 3.98 – 3.74 (m, 4H), 3.08 – 2.79 (m, 3H), 2.63 – 2.48 (m, 2H), 2.34 (s, 3H), 1.43 (s, 9H); $^{13}C\{^1H\}$ NMR (100 MHz, $CDCl_3$) 171.9, 158.8 (d, $J_{F\text{-}C}$ = 257 Hz), 154.6, 153.4 (d, $J_{F\text{-}C}$ = 3.7 Hz), 142.5, 138.5, 135.3 (d, $J_{F\text{-}C}$ = 22.9 Hz), 133.0 (d, $J_{F\text{-}C}$ = 3.9 Hz), 130.1, 128.8, 128.6, 128.2, 125.2 (d, $J_{F\text{-}C}$ = 17.9 Hz), 80.2, 68.4, 62.7, 60.6, 54.9, 44.8, 43.8, 28.4, 20.1; HRMS (ESI) m/z: $[M + H]^+$ calcd. for $C_{24}H_{29}FN_3O_4^+$, 442.2137; Found: 442.2137.

tert-Butyl 10-(3,5-dimethylisoxazol-4-yl)-7-oxo-1,2,4a,5,7,12-hexahydrobenzo[f]pyrazino [2,1-c][1,4]oxazocine-3(4H)-carboxylate (5r)

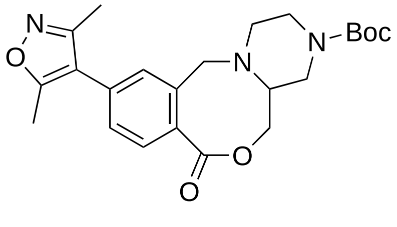

tert-Butyl 7-oxo-10-(4,4,5,5-tetramethyl-1,3,2-dioxaborolan-2-yl)-1,2,4a,5,7,12-hexahydro benzo[f]pyrazino[2,1-c][1,4]oxazocine-3(4H)-carboxylate **4j** (140 mg, 0.305 mmol) and 4-bromo-3,5-dimethylisoxazole (108 mg, 0.611 mmol) were dissolved in 1,4-dioxane (6.0 mL). Then Na_2CO_3 (97.1 mg, 0.916 mmol) which dissolved in water (3.0 mL) was added followed by Pd(dppf)Cl_2·CH_2Cl_2 (12.2 mg, 0.015 mmol) and the resulting mixture was purged with argon for 10 minutes. The mixture was stirred and heated at 50 °C for 18 hours under argon. After being allowed to cool to RT, water (10 mL) was added and the two layers were separated. The

aqueous layer was extracted with dichloromethane (3 x 10 mL) and the combined organic phases were dried with magnesium sulphate, filtered and concentrated in vacuo to give the crude product. The product was purified by column chromatography (SiO_2, 3:1 → 1:1 hexane : ethyl acetate) to afford the title compound (35.3 mg, 27%) as a white solid. R_f = 0.16 (2:1 hexane : ethyl acetate); m.p. 111–115 °C; ν_{max}/cm^{-1} (thin film) 2924, 2853, 1721, 1692, 1456, 1425, 1366, 1281, 1241, 1171, 1123, 1091, 1048, 1014, 873, 756; δ_H (400 MHz, $CDCl_3$) δ 7.58 (d, 1H, *J* = 8.0 Hz), 7.24 (dd, 1H, *J* = 8.0, 2.0 Hz), 7.08 (d, 1H, *J* = 2.0 Hz), 4.20 (dd, 1H, *J* = 13.0, 2.0 Hz), 4.06 (dd, 1H, *J* = 13.0, 4.0 Hz), 4.00 – 3.78 (m, 4H), 3.06 – 2.86 (m, 3H), 2.66 – 2.55 (m, 2H), 2.42 (s, 3H), 2.28 (s, 3H), 1.46 (s, 9H); $^{13}C\{^1H\}$ NMR (100 MHz, $CDCl_3$) 171.8, 165.9, 158.4, 154.7, 138.9, 133.8, 131.0, 128.6, 128.5, 128.3, 111.7, 80.4, 68.6, 62.8, 60.6, 54.9, 44.9, 43.2, 28.5, 11.8, 11.0; HRMS (ESI) m/z: $[M + Na]^+$ calcd. for $C_{23}H_{29}N_3NaO_5^+$, 450.1999; Found: 450.2010.

***tert*-Butyl 10-(2-methoxypyrimidin-5-yl)-7-oxo-1,2,4a,5,7,12-hexahydrobenzo[f]pyrazino [2,1-c][1,4]oxazocine-3(4H)-carboxylate (5s)**

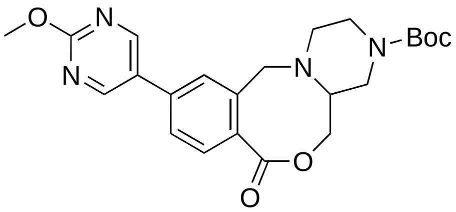

tert-Butyl 7-oxo-10-(4,4,5,5-tetramethyl-1,3,2-dioxaborolan-2-yl)-1,2,4a,5,7,12-hexahydro benzo[f]pyrazino[2,1-c][1,4]oxazocine-3(4H)-carboxylate **4j** (140 mg, 0.305 mmol) and 5-bromo-2-methoxypyrimidine (116 mg, 0.611 mmol) were dissolved in 1,4-dioxane (6.0 mL). Then Na_2CO_3 (97.1 mg, 0.916 mmol) which dissolved in water (3.0 mL) was added followed by $Pd(dppf)Cl_2 \cdot CH_2Cl_2$ (12.2 mg, 0.015 mmol) and the resulting mixture was purged with argon for 10 minutes. The mixture was stirred and heated at 50 °C for 18 hours under argon. After being allowed to cool to RT, water (10 mL) was added and the two layers were separated. The aqueous layer was extracted with dichloromethane (3 x 10 mL) and the combined organic phases were dried with magnesium sulphate, filtered and concentrated in vacuo to give the crude product. The product was purified by column chromatography (SiO_2, 1:1 → 1:2 hexane : ethyl acetate) to afford the title compound (35.0 mg, 26%) as a yellow solid. R_f = 0.31 (1:2 hexane : ethyl acetate); m.p. 104–107 °C; ν_{max}/cm^{-1} (thin film) 2975, 2932, 1720, 1693, 1596, 1473, 1411, 1334, 1281, 1243, 1170, 1124, 1091, 1034, 802, 766; δ_H (400 MHz, $CDCl_3$) 8.72 (s, 2H), 7.60 (d, 1H, *J* = 8.0 Hz), 7.49 (dd, 1H, *J* = 8.0, 2.0 Hz), 7.35 (d, 1H, *J* = 2.0 Hz), 4.16 (dd, 1H, *J* = 13.0, 2.0 Hz), 4.07–4.03 (m, 4H), 3.96 – 3.78 (m, 4H), 3.08 – 2.79 (m, 3H), 2.60 (2H, ddd, *J* = 15.0, 9.0, 3.5 Hz), 1.45 (s, 9H); $^{13}C\{^1H\}$ NMR (100 MHz, $CDCl_3$) 171.5, 165.6, 157.5, 157.0, 154.6, 139.3, 137.5, 131.4, 128.8, 127.0, 126.2, 125.9, 80.3, 68.7, 62.8, 60.5, 55.4, 55.1, 45.6, 43.9, 28.5; HRMS (ESI) m/z: $[M + Na]^+$ calcd. for $C_{23}H_{28}N_4NaO_5^+$, 463.1952; Found: 463.1954.

tert-Butyl 10-(1,3-dimethyl-2,4-dioxo-1,2,3,4-tetrahydropyrimidin-5-yl)-7-oxo-1,2,4a,5,7,12-hexahydrobenzo[f]pyrazino[2,1-c][1,4]oxazocine-3(4H)-carboxylate (5t)

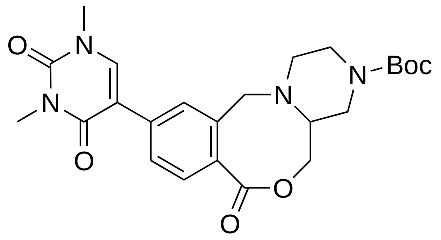

tert-Butyl 7-oxo-10-(4,4,5,5-tetramethyl-1,3,2-dioxaborolan-2-yl)-1,2,4a,5,7,12-hexahydro benzo[f]pyrazino[2,1-c][1,4]oxazocine-3(4H)-carboxylate **4j** (140 mg, 0.305 mmol) and 5-bromo-1,3-dimethylpyrimidine-2,4(1H,3H)-dione (134 mg, 0.611 mmol) were dissolved in 1,4-dioxane (6.0 mL). Then Na_2CO_3 (97.1 mg, 0.916 mmol) which dissolved in water (3.0 mL) was added followed by Pd(dppf)$Cl_2 \cdot CH_2Cl_2$ (12.2 mg, 0.015 mmol) and the resulting mixture was purged with argon for 10 minutes. The mixture was stirred and heated at 50 °C for 18 hours under argon. After being allowed to cool to RT, water (10 mL) was added and the two layers were separated. The aqueous layer was extracted with dichloromethane (3 x 10 mL) and the combined organic phases were dried with magnesium sulphate, filtered and concentrated in vacuo to give the crude product. The product was purified by column chromatography (SiO_2, 1:1 → 0:1 hexane : ethyl acetate) to afford the title compound (54.9 mg, 38%) as a brown solid. R_f = 0.30 (ethyl acetate); m.p. 117–123 °C; ν_{max}/cm^{-1} (thin film) 2976, 1698, 1650, 1455, 1429, 1365, 1281, 1243, 1170, 1122, 1021, 918, 756, 730; δ_H (400 MHz, $CDCl_3$) 7.51 – 7.33 (m, 4H), 4.11 (d, 1H, _J_ = 13.0 Hz), 3.97 (dd, 1H, _J_ = 13.0, 4.0 Hz), 3.94 – 3.68 (m, 4H), 3.47 (s, 3H), 3.37 (s, 3H), 3.02 – 2.76 (m, 3H), 2.53 (td, 2H, _J_ = 12.5, 4.5 Hz), 1.42 (s, 9H); $^{13}C\{^1H\}$ NMR (100 MHz, $CDCl_3$) 171.8, 162.1, 154.5, 151.2, 141.5, 138.2, 136.0, 130.4, 128.1, 128.1, 127.1, 112.6, 80.2, 68.5, 62.6, 60.4, 54.8, 44.7, 43.8, 37.3, 28.4, 28.3; HRMS (ESI) m/z: $[M + H]^+$ calcd. for $C_{24}H_{31}N_4O_6^+$, 471.2238; Found: 471.2243.

10-(4-Fluoro-2-methoxyphenyl)-1,2,3,4,4a,5-hexahydrobenzo[f]pyrazino[2,1-c][1,4] oxazocin-7(12H)-one (S3)

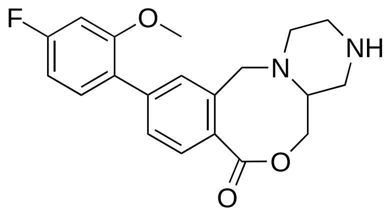

tert-Butyl 10-(4-fluoro-2-methoxyphenyl)-7-oxo-1,2,4a,5,7,12-hexahydrobenzo[f]pyrazino [2,1-c][1,4]oxazocine-3(4H)-carboxylate **5k** (50 mg, 0.110 mmol) was dissolved in 4 M HCl in dioxane (4.0 mL). The solution was stirred at room temperature for 1 hour, after which the solution was concentrated in vacuo to yield the salt product. Sodium bicarbonate solution was added while stirring until the pH is adjusted to 7-8. The aqueous layer was extracted with ethyl acetate (3 x 10 mL) and the combined organic phases were dried with magnesium sulphate, filtered and concentrated in vacuo to give the crude product. The product was purified by column chromatography (SiO_2, 50:1 → 10:1 dichloromethane : methanol) to

afford the title compound (29.0 mg, 74%) as a white solid. R_f = 0.10 (10:1 dichloromethane : methanol); m.p. 206–210 °C; $ν_{max}/cm^{-1}$ (thin film) 2949, 2760, 1715, 1607, 1455, 1378, 1347, 1278, 1231, 1148, 1098, 1031, 955, 829, 803, 765; $δ_H$ (400 MHz, $CDCl_3$) 7.54 – 7.47 (m, 1H), 7.44 (dd, 1H, *J* = 8.0, 1.5 Hz), 7.31 – 7.20 (m, 2H), 6.77 – 6.65 (m, 2H), 5.59 (s, 1H, N**H**), 4.22 (dd, 1H, *J* = 13.0, 2.0 Hz), 4.04 (dd, 1H, *J* = 13.0, 3.5 Hz), 3.93 – 3.80 (m, 2H), 3.79 (s, 3H), 3.17 – 3.05 (m, 2H), 3.04 – 2.87 (m, 3H), 2.86 – 2.65 (m, 2H); $^{13}C\{^1H\}$ NMR (100 MHz, $CDCl_3$) 172.2, 163.6 (d, J_{F-C} = 246 Hz), 157.7 (d, J_{F-C} = 10.0 Hz), 140.8, 138.0, 131.5 (d, J_{F-C} = 10.1 Hz), 130.2, 129.0, 128.8, 127.2, 125.1 (d, J_{F-C} = 3.5 Hz), 107.4 (d, J_{F-C} = 21.0 Hz), 99.6 (d, J_{F-C} = 25.7 Hz), 68.3, 62.5, 60.9, 55.9, 54.3, 46.5, 45.0; HRMS (ESI) m/z: $[M + H]^+$ calcd. for $C_{20}H_{22}FN_2O_3^+$, 357.1609; Found: 357.1613.

3-((3,5-Dimethylisoxazol-4-yl)sulfonyl)-10-(4-fluoro-2-methoxyphenyl)-1,2,3,4,4a,5-hexahydrobenzo[f]pyrazino[2,1-c][1,4]oxazocin-7(12H)-one (6a)

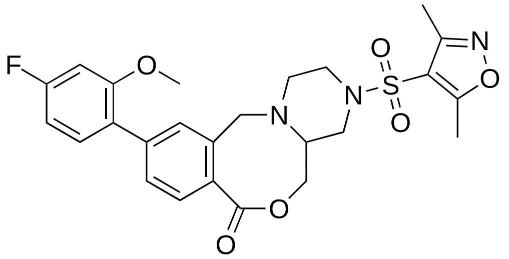

tert-Butyl 10-(4-fluoro-2-methoxyphenyl)-7-oxo-1,2,4a,5,7,12-hexahydrobenzo[f]pyrazino [2,1-c][1,4]oxazocine-3(4H)-carboxylate **5k** (50 mg, 0.110 mmol) was dissolved in 4 M HCl in dioxane (4.0 mL). The solution was stirred at room temperature for 1 hour, after which the solution was concentrated in vacuo to yield the salt product. This salt was dissolved in anhydrous DCM (3.0 mL) at room temperature and the solution was cooled to 0 °C using an ice bath. Triethylamine (0.05 mL, 0.329 mmol) was added dropwise, followed by 3,5-dimethylisoxazole-4-sulfonyl chloride (26.8 mg, 0.137 mmol) and DMAP (1.4 mg, 0.011 mmol). The solution was allowed to gradually warm to room temperature while being stirred for 18 hours. The solution was diluted with ethyl acetate (10 mL) and washed with brine (10 mL). The aqueous layer was then extracted with ethyl acetate (3 x 10 mL). The organic layers were then combined, dried with magnesium sulphate, filtered and concentrated in vacuo to give the crude product. The product was purified by column chromatography (SiO_2, 2:1 → 1:1 hexane : ethyl acetate) to afford the title compound (54.4 mg, 96%) as a white solid. R_f = 0.55 (1:1 hexane : ethyl acetate); m.p. 177–183 °C; $ν_{max}/cm^{-1}$ (thin film) 2855, 1710, 1597, 1454, 1407, 1325, 1308, 1281, 1268, 1185, 1152, 1123, 1109, 1090, 1025, 975, 952, 913, 831, 766, 690, 664; $δ_H$ (400 MHz, $CDCl_3$) 7.50 (d, 1H, *J* = 8.0 Hz), 7.46 (dd, 1H, *J* = 8.0, 1.5 Hz), 7.27 (d, 1H, *J* = 1.5 Hz), 7.22 (dd, 1H, *J* = 8.5, 6.5 Hz), 6.77 – 6.68 (m, 2H), 4.25 (d, 1H, *J* = 13.0 Hz), 4.05 (dd, 1H, *J* = 13.0, 3.0 Hz), 3.88 (d, 2H, *J* = 3.0 Hz), 3.79 (s, 3H), 3.65 – 3.57 (m, 1H), 3.54 (dd, 1H, *J* = 8.0, 2.0 Hz), 3.05 – 2.96 (m, 1H), 2.85 – 2.69 (m, 4H), 2.63 (s, 3H), 2.38 (s, 3H); $^{13}C\{^1H\}$ NMR (100 MHz, $CDCl_3$) 174.0 (**C**), 171.8 (**C**), 163.6 (d, J_{F-C} = 252 Hz), 157.9, 157.6 (d, J_{F-C} = 9.6 Hz), 141.0, 137.4, 131.5 (d, J_{F-C} = 9.9 Hz), 130.3, 129.1, 128.7, 126.9, 125.0, 113.2, 107.4 (d, J_{F-C} = 21.1 Hz), 99.7 (d, J_{F-C} = 25.9 Hz), 67.9, 62.3, 60.3, 55.9, 54.2, 47.1, 45.3, 13.1, 11.5; HRMS (ESI) m/z: $[M + H]^+$ calcd. for $C_{25}H_{27}FN_3O_6S^+$, 516.1599; Found: 516.1617.

10-(4-Fluoro-2-methoxyphenyl)-3-((5-methylfuran-2-yl)methyl)-1,2,3,4,4a,5-hexahydrobenzo[f]pyrazino[2,1-c][1,4]oxazocin-7(12H)-one (6b)

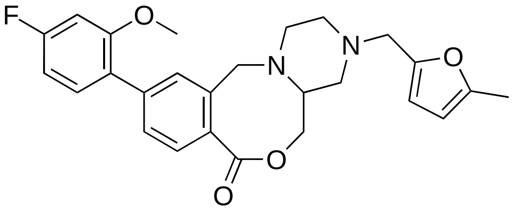

tert-Butyl 10-(4-fluoro-2-methoxyphenyl)-7-oxo-1,2,4a,5,7,12-hexahydrobenzo[f]pyrazino [2,1-c][1,4]oxazocine-3(4H)-carboxylate **5k** (80 mg, 0.175 mmol) was dissolved in 4 M HCl in dioxane (6.0 mL). The solution was stirred at room temperature for 1 hour, after which the solution was concentrated in vacuo to yield the salt product. This salt was dissolved in anhydrous THF (2.0 mL), 5-methylfuran-2-carbaldehyde (19.3 mg, 0.175 mmol) was added, followed by acetic acid (0.01 mL, 0.175 mmol) and the solution was stirred for 10 minutes. Then sodium triacetoxyborohydride (74.3 mg, 0.350 mmol) was added and the reaction mixture was stirred at room temperature for 18 hours. The mixture was diluted with ethyl acetate (10 mL) and washed with sodium bicarbonate solution (10 mL). The aqueous layer was then extracted with ethyl acetate (3 x 10 mL). The organic layers were then combined, dried with magnesium sulphate, filtered and concentrated in vacuo to give the crude product. The product was purified by column chromatography (SiO_2, 2:1 → 1:1 hexane : ethyl acetate) to afford the title compound (75.2 mg, 95%) as a white solid. R_f = 0.16 (1:1 hexane : ethyl acetate); m.p. 97–101 °C; ν_{max}/cm^{-1} (thin film) 1718, 1606, 1512, 1488, 1458, 1278, 1153, 1114, 1025, 955, 836, 735; δ_H (400 MHz, $CDCl_3$) 7.49 (d, 1H, J = 8.0 Hz), 7.43 (dd, 1H, J = 8.0, 1.5 Hz), 7.30 – 7.20 (m, 2H), 6.76 – 6.67 (m, 2H), 6.06 (d, 1H, J = 3.0 Hz), 5.87 (dd, 1H, J = 2.5, 1.5 Hz), 4.15 (dd, 1H, J = 13.0, 2.0 Hz), 4.01 (dd, 1H, J = 13.0, 3.5 Hz), 3.89 – 3.76 (m, 5H), 3.47 (s, 2H), 2.89 (dt, 1H, J = 12.0, 3.0 Hz), 2.83 – 2.66 (m, 4H), 2.36 – 2.17 (m, 5H, 2 x $NCH_a\mathbf{H_b}$, $C\mathbf{H_3}$); $^{13}C\{^1H\}$ NMR (100 MHz, $CDCl_3$) 172.4, 163.5 (d, J_{F-C} = 245 Hz), 157.6 (d, J_{F-C}= 9.6 Hz), 152.2, 149.2, 140.6, 138.1, 131.5 (d, J_{F-C} = 9.9 Hz), 130.1, 128.9, 128.8, 127.5, 125.2 (d, J_{F-C} = 3.0 Hz), 110.1, 107.4 (d, J_{F-C} = 21.1 Hz), 106.1, 99.6 (d, J_{F-C} = 25.7 Hz), 69.2, 62.8, 60.5, 55.8, 55.1, 54.9, 54.8, 52.7, 13.8; HRMS (ESI) m/z: $[M + H]^+$ calcd. for $C_{26}H_{28}FN_2O_4^+$, 451.2028; Found: 451.2031.

3-(Cyclopentanecarbonyl)-10-(4-fluoro-2-methoxyphenyl)-1,2,3,4,4a,5hexahydrobenzo [f]pyrazino[2,1-c][1,4]oxazocin-7(12H)-one (6c)

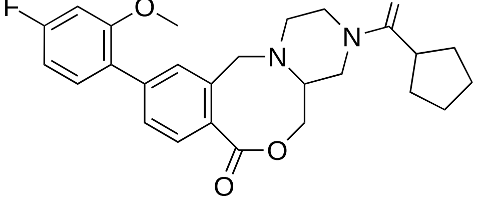

tert-Butyl 10-(4-fluoro-2-methoxyphenyl)-7-oxo-1,2,4a,5,7,12-hexahydrobenzo[f]pyrazino [2,1-c][1,4]oxazocine-3(4H)-carboxylate **5k** (50 mg, 0.110 mmol) was dissolved in 4 M HCl in dioxane (4.0 mL). The solution was stirred at room temperature for 1 hour, after which the

solution was concentrated in vacuo to yield the salt product. This salt was dissolved in anhydrous DCM (3.0 mL) at room temperature and the solution was cooled to 0 °C using an ice bath. Triethylamine (0.05 mL, 0.329 mmol) was added dropwise, followed by cyclopentane carbonyl chloride (18.2 mg, 0.137 mmol) and DMAP (1.4 mg, 0.011 mmol). The solution was allowed to gradually warm to room temperature while being stirred for 18 hours. The solution was diluted with ethyl acetate (10 mL) and washed with brine (10 mL). The aqueous layer was then extracted with ethyl acetate (3 x 10 mL). The organic layers were then combined, dried with magnesium sulphate, filtered and concentrated in vacuo to give the crude product. The product was purified by column chromatography (SiO_2, 1:1 hexane : ethyl acetate) to afford the title compound (47.5 mg, 95%) as a white solid. R_f = 0.37 (1:1 hexane : ethyl acetate); m.p. 90–98 °C; ν_{max}/cm^{-1} (thin film) 2951, 1719, 1636, 1607, 1450, 1279, 1228, 1153, 1112, 1091, 954, 836, 734; δ_H (400 MHz, $CDCl_3$) 7.51 (dd, 1H, *J* = 8.0, 2.5 Hz), 7.45 (dd, 1H, *J* = 8.0, 2.0 Hz), 7.28 (d, 1H, *J* = 8.0 Hz), 7.23 (t, 1H, *J* = 7.5 Hz), 6.81 – 6.63 (m, 2H), 4.54 – 4.37 (m, 1H), 4.24 (ddd, 1H, *J* = 13.0, 6.5, 2.0 Hz), 4.13 – 4.01 (m, 1H), 3.92 – 3.65 (m, 6H), 3.28 (tt, 1H, *J* = 13.0, 2.5 Hz), 2.95 (tt, 1H, *J* = 10.5, 2.5 Hz), 2.92 – 2.74 (m, 2H), 2.67 – 2.46 (m, 2H), 1.91 – 1.64 (m, 6H, 3 x C**H₂**), 1.63 – 1.47 (m, 2H); $^{13}C\{^1H\}$ NMR (100 MHz, $CDCl_3$) 174.7 (rotamer A), 174.5 (rotamer B), 172.2 (rotamer A), 172.0 (rotamer B), 164.8 (rotamer A), 162.3 (rotamer B), 157.6 (d, $J_{F\text{-}C}$ = 9.6 Hz, both rotamers), 140.9 (rotamer A), 140.7 (rotamer B), 137.9 (rotamer A), 137.8 (rotamer B), 131.5 (rotamer A), 131.4 (rotamer B), 130.2 (rotamer A), 130.1 (rotamer B), 129.0 (both rotamers), 128.8 (rotamer A), 128.7 (rotamer B), 127.4 (rotamer A), 127.1 (rotamer B), 125.1 (rotamer A), 125.0 (rotamer B), 107.4 (d, $J_{F\text{-}C}$ = 21.1 Hz, both rotamers), 99.6 (d, $J_{F\text{-}C}$ = 25.7 Hz, both rotamers), 68.3 (rotamer A), 68.2 (rotamer B), 63.4 (rotamer A), 62.7 (rotamer B), 60.7 (rotamer A), 60.6 (rotamer B), 55.9 (both rotamers), 55.4 (N**C**H_2, rotamer A), 54.9 (rotamer B), 44.6 (both rotamers), 41.0 (both rotamers), 30.4 (both rotamers), 30.0 (rotamer A), 30.0 (rotamer B), 26.1 (rotamer A), 26.1 (rotamer B); HRMS (ESI) m/z: $[M + H]^+$ calcd. for $C_{26}H_{30}FN_2O_4^+$, 453.2184; Found: 453.2180.

10-(4-Fluoro-2-methoxyphenyl)-3-(4-(trifluoromethyl)benzyl)-1,2,3,4,4a,5-hexahydrobenzo[f]pyrazino[2,1-c][1,4]oxazocin-7(12H)-one (6d)

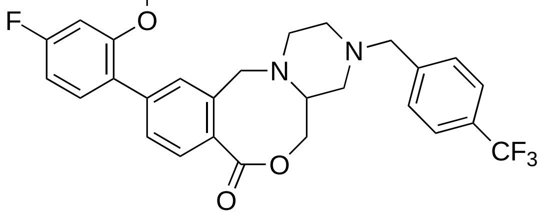

tert-Butyl 10-(4-fluoro-2-methoxyphenyl)-7-oxo-1,2,4a,5,7,12-hexahydrobenzo[f]pyrazino [2,1-c][1,4]oxazocine-3(4H)-carboxylate **5k** (50 mg, 0.110 mmol) was dissolved in 4 M HCl in dioxane (4.0 mL). The solution was stirred at room temperature for 1 hour, after which the solution was concentrated in vacuo to yield the salt product. This salt was dissolved in anhydrous THF (5.0 mL), 1-(bromomethyl)-4-(trifluoromethyl) benzene (25.5 mg, 0.137 mmol) was added followed by triethylamine (0.05 mL, 0.329 mmol). The reaction mixture was refluxed at 70 °C for 18 hours under argon. Then the reaction mixture was allowed to cool to RT, filtered and washed with dichloromethane, concentrated under vacuum and purified by column chromatography (SiO_2, 2:1 → 1:1 hexane : ethyl acetate) to afford the title compound

(44.3 mg, 78%) as a white solid. R_f = 0.49 (1:1 hexane : ethyl acetate); m.p. 125–128 °C; v_{max}/cm^{-1} (thin film) 2982, 1720, 1607, 1325, 1281, 1156, 1118, 1066, 955, 836; δ_H (400 MHz, $CDCl_3$) 7.57 (d, 2H, *J* = 8.0 Hz), 7.52 (d, 1H, *J* = 8.0 Hz), 7.48 – 7.42 (m, 3H), 7.29 (d, 1H, *J* = 1.5 Hz), 7.25 (dd, 1H, *J* = 8.0, 6.5 Hz), 6.78 – 6.69 (m, 2H), 4.15 (dd, 1H, *J* = 13.0, 2.0 Hz), 4.02 (dd, 1H, *J* = 13.0, 3.5 Hz), 3.92 – 3.78 (m, 5H), 3.55 (d, 2H, *J* = 3.0 Hz), 2.94 – 2.85 (m, 1H), 2.77 (dd, 1H, *J* = 11.0, 3.0 Hz), 2.74 – 2.68 (m, 2H), 2.64 (ddd, 1H, *J* = 11.0, 3.0, 1.5 Hz), 2.38 – 2.27 (m, 2H); $^{13}C\{^1H\}$ NMR (100 MHz, $CDCl_3$) 172.4, 164.8, 162.3, 157.7 (d, J_{F-C} = 9.9 Hz), 142.4, 140.6, 138.1, 131.5 (d, J_{F-C} = 9.8 Hz), 130.2, 129.6 (q, J_{F-C} = 31.9 Hz), 129.3, 129.0, 128.9, 125.4 (d, J_{F-C} = 3.7 Hz), 125.3, 124.1 (q, J_{F-C} = 270 Hz), 107.4 (d, J_{F-C} = 21.1 Hz), 99.7 (d, J_{F-C} = 25.7 Hz), 69.1, 63.1, 62.3, 60.6, 55.9, 55.4, 54.9, 53.0; HRMS (ESI) m/z: $[M + H]^+$ calcd. for $C_{28}H_{27}F_4N_2O_3^+$, 515.1952; Found: 515.1950.

10-(4-Fluoro-2-methoxyphenyl)-3-(pyrimidin-5-yl)-1,2,3,4,4a,5-hexahydrobenzo[f] pyrazino[2,1-c][1,4]oxazocin-7(12H)-one (6e)

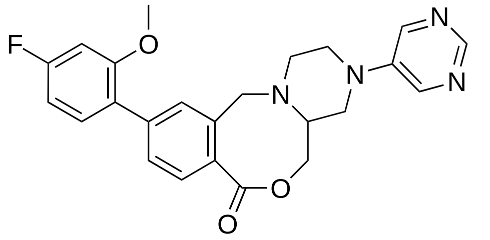

tert-Butyl 10-(4-fluoro-2-methoxyphenyl)-7-oxo-1,2,4a,5,7,12-hexahydrobenzo[f]pyrazino [2,1-c][1,4]oxazocine-3(4H)-carboxylate **5k** (50 mg, 0.110 mmol) was dissolved in 4 M HCl in dioxane (4.0 mL). The solution was stirred at room temperature for 1 hour, after which the solution was concentrated in vacuo to yield the salt product. This salt and 5-bromopyrimidine (21.8 mg, 0.137 mmol) were dissolved in toluene (2.0 mL). Then Cs_2CO_3 (107 mg, 0.330 mmol) and (±)-BINAP (15.8 mg, 0.016 mmol) were added followed by $Pd_2(dba)_3$ (10.1 mg, 0.011 mmol) and the resulting mixture was purged with argon for 10 minutes. The mixture was stirred and heated at 110 °C for 65 hours under argon. After being allowed to cool to RT, water (10 mL) was added and the two layers were separated. The aqueous layer was extracted with ethyl acetate (3 x 10 mL) and the combined organic phases were dried with magnesium sulphate, concentrated under vacuum and purified by column chromatography (SiO_2, 2:1 → 0:1 hexane : ethyl acetate) to afford the title compound (28.9 mg, 61%) as a yellow solid. R_f = 0.48 (ethyl acetate); m.p. 176–180 °C; v_{max}/cm^{-1} (thin film) 2925, 2845, 1718, 1606, 1568, 1513, 1488, 1448, 1280, 1241, 1192, 1153, 1115, 1089, 1031, 955, 837, 726; δ_H (400 MHz, $CDCl_3$) 8.72 (s, 1H), 8.38 (s, 2H), 7.54 (d, 1H, *J* = 8.0 Hz), 7.48 (dd, 1H, *J* = 8.0, 1.5 Hz), 7.33 (d, 1H, *J* = 1.5 Hz), 7.26 – 7.22 (m, 1H), 6.79 – 6.68 (m, 2H), 4.28 (dd, 1H, *J* = 13.0, 2.0 Hz), 4.16 (dd, 1H, *J* = 13.0, 4.0 Hz), 3.90 (d, 2H, *J* = 3.0 Hz), 3.80 (s, 3H), 3.57 (dd, 1H, *J* = 11.5, 2.5 Hz), 3.48 (ddd, 1H, *J* = 11.5, 3.5, 1.5 Hz), 3.14 – 2.95 (m, 3H), 2.90 – 2.77 (m, 2H); $^{13}C\{^1H\}$ NMR (100 MHz, $CDCl_3$) 172.0, 164.5 (d, J_{F-C} = 246 Hz), 162.4, 157.7 (d, J_{F-C} = 9.6 Hz), 150.3, 144.1, 144.0, 141.0, 137.7, 131.6 (d, J_{F-C} = 9.8 Hz), 130.2, 129.2, 127.3, 125.1 (d, J_{F-C} = 3.1 Hz), 107.5 (d, J_{F-C} = 9.8 Hz,Ar**C**), 99.7 (d, J_{F-C} = 25.7 Hz), 68.7, 62.6, 60.6, 55.9, 54.6, 49.6, 47.7; HRMS (ESI) m/z: $[M + Na]^+$ calcd. for $C_{24}H_{23}FN_4NaO_3^+$, 457.1646; Found: 457.1652.

10-(Benzo[d]thiazol-6-yl)-1,2,3,4,4a,5-hexahydrobenzo[f]pyrazino[2,1-c][1,4] oxazocin-7(12H)-one (S4)

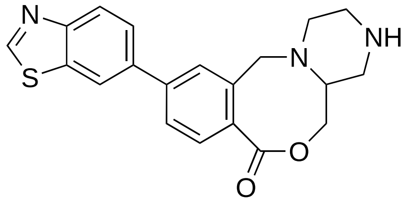

tert-Butyl 10-(benzo[d]thiazol-6-yl)-7-oxo-1,2,4a,5,7,12-hexahydrobenzo[f]pyrazino[2,1-c][1,4]oxazocine-3(4H)-carboxylate **5l** (50 mg, 0.110 mmol) was dissolved in 4 M HCl in dioxane (4.0 mL). The solution was stirred at room temperature for 1 hour, after which the solution was concentrated in vacuo to yield the salt product. Sodium bicarbonate solution was added while stirring until the pH is adjusted to 7-8. The aqueous layer was extracted with ethyl acetate (3 x 10 mL) and the combined organic phases were dried with magnesium sulphate, filtered and concentrated in vacuo to give the crude product. The product was purified by column chromatography (SiO_2, 10:1 dichloromethane: methanol) to afford the title compound (25.1 mg, 62%) as a white solid. R_f = 0.40 (50:1 → 10:1 methanol : dichloromethane); m.p. 144–149 °C; ν_{max}/cm^{-1} (thin film) 2923, 1712, 1607, 1462, 1440, 1378, 1354, 1277, 1255, 1233, 1126, 1095, 1023, 1002, 867, 833, 807, 767; δ_H (400 MHz, DMSO-*D6*) 9.45 (s, 1H), 8.57 (d, 1H, *J* = 2.0 Hz), 8.20 (d, 1H, *J* = 8.5 Hz), 7.91 (dd, 1H, *J* = 8.5, 2.0 Hz), 7.81 – 7.74 (m, 2H), 7.52 (d, 1H, *J* = 8.0 Hz), 4.20 (d, 1H, *J* = 13.0 Hz), 4.08 – 3.98 (m, 2H), 3.88 (d, 1H, *J* = 16.5 Hz), 3.19 (d, 2H, *J* = 10.5 Hz), 3.10 (d, 1H, *J* = 11.5 Hz), 2.88 – 2.68 (m, 4H); $^{13}C\{^1H\}$ NMR (101 MHz, DMSO-*D6*) 171.1, 157.2, 152.9, 141.7, 139.3, 136.1, 134.7, 130.8, 127.6, 126.6, 125.9, 125.4, 123.4, 120.8, 67.3, 60.2, 59.2, 51.6, 44.4, 43.3; HRMS (ESI) m/z: $[M + H]^+$ calcd. for $C_{20}H_{20}N_3O_2S^+$, 366.1271; Found: 366.1275.

10-(Benzo[d]thiazol-6-yl)-3-(cyclopropylsulfonyl)-1,2,3,4,4a,5-hexahydrobenzo[f] pyrazino[2,1-c][1,4]oxazocin-7(12H)-one (6f)

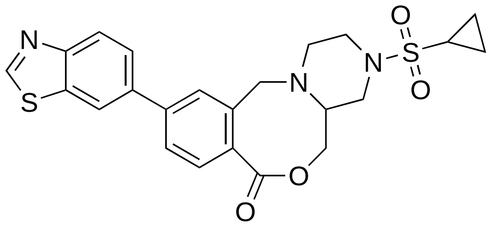

tert-Butyl 10-(benzo[d]thiazol-6-yl)-7-oxo-1,2,4a,5,7,12-hexahydrobenzo[f]pyrazino[2,1-c][1,4]oxazocine-3(4H)-carboxylate **5l** (50 mg, 0.110 mmol) was dissolved in 4 M HCl in dioxane (4.0 mL). The solution was stirred at room temperature for 1 hour, after which the solution was concentrated in vacuo to yield the salt product. This salt was dissolved in anhydrous DCM (3.0 mL) at room temperature and the solution was cooled to 0 °C using an ice bath. Triethylamine (0.05 mL, 0.329 mmol) was added dropwise, followed by cyclopropanesulfonyl chloride (19.3 mg, 0.137 mmol) and DMAP (1.4 mg, 0.011 mmol). The solution was allowed to gradually warm to room temperature while being stirred for 18 hours. The solution was diluted with ethyl acetate (10 mL) and washed with brine (10 mL). The aqueous layer was then extracted with ethyl acetate (3 x 10 mL). The organic layers were then combined, dried

with magnesium sulphate, filtered and concentrated in vacuo to give the crude product. The product was purified by column chromatography (SiO_2, 1:1 → 0:1 hexane : ethyl acetate) to afford the title compound (38.2 mg, 74%) as a white solid. R_f = 0.51 (ethyl acetate); m.p. 206–210 °C; ν_{max}/cm^{-1} (thin film) 2923, 2853, 1717, 1608, 1464, 1335, 1309, 1287, 1153, 1119, 1010, 990, 769, 734; δ_H (400 MHz, $CDCl_3$) 9.04 (s, 1H), 8.20 (d, 1H, *J* = 8.5 Hz), 8.16 (d, 1H, *J* = 2.0 Hz), 7.73 (dd, 1H, *J* = 8.5, 2.0 Hz), 7.67 – 7.56 (m, 2H), 7.46 (d, 1H, *J* = 1.5 Hz), 4.28 (dd, 1H, *J* = 13.0, 2.0 Hz), 4.05 (dd, 1H, *J* = 13.0, 3.0 Hz), 4.02 – 3.87 (m, 2H), 3.63 (2H, ddt, *J* = 17.0, 11.5, 2.5 Hz), 3.18 – 2.95 (m, 3H), 2.90 – 2.69 (m, 2H), 2.27 (tt, 1H, *J* = 8.0, 5.0 Hz), 1.22 – 1.10 (m, 2H), 1.07 – 0.94 (m, 2H); $^{13}C\{^1H\}$ NMR (100 MHz, $CDCl_3$) 171.9, 154.9, 153.2, 143.3, 138.8, 137.3, 134.8, 131.4, 127.7, 126.9, 126.5, 125.8, 124.1, 120.5, 67.9, 62.6, 60.6, 54.7, 47.8, 45.0, 25.5, 4.6, 4.5; HRMS (ESI) m/z: $[M + H]^+$ calcd. for $C_{23}H_{24}N_3O_4S_2^+$, 470.1203; Found: 470.1237.

3-((1H-Pyrrolo[2,3-b]pyridin-3-yl)methyl)-10-(benzo[d]thiazol-6-yl)-1,2,3,4,4a,5-hexahydrobenzo[f]pyrazino[2,1-c][1,4]oxazocin-7(12H)-one (6g)

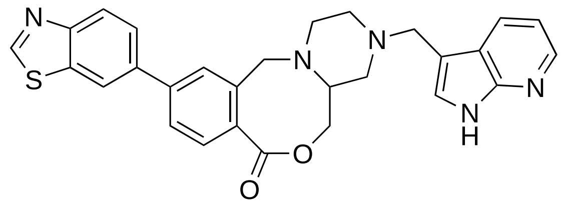

tert-Butyl 10-(benzo[d]thiazol-6-yl)-7-oxo-1,2,4a,5,7,12-hexahydrobenzo[f]pyrazino[2,1-c][1,4]oxazocine-3(4H)-carboxylate **5l** (50 mg, 0.110 mmol) was dissolved in 4 M HCl in dioxane (4.0 mL). The solution was stirred at room temperature for 1 hour, after which the solution was concentrated in vacuo to yield the salt product. This salt was dissolved in anhydrous THF (2.0 mL), 1H-pyrrolo[2,3-b]pyridine-3-carbaldehyde (12.6 mg, 0.110 mmol) was added, followed by acetic acid (0.007 mL, 0.110 mmol) and the solution was stirred for 10 minutes. Then sodium triacetoxyborohydride (46.4 mg, 0.219 mmol) was added and the reaction mixture was stirred at room temperature for 18 hours. The mixture was diluted with ethyl acetate (10 mL) and washed with sodium bicarbonate solution (10 mL). The aqueous layer was then extracted with ethyl acetate (3 x 10 mL). The organic layers were then combined, dried with magnesium sulphate, filtered and concentrated in vacuo to give the crude product. The product was purified by column chromatography (SiO_2, 90:9:1 dichloromethane : methanol : triethylamine) to afford the title compound (27.8 mg, 51%) as a yellow solid. R_f = 0.58 (90:9:1 dichloromethane : methanol : triethylamine); m.p. 237–241 °C; ν_{max}/cm^{-1} (thin film) 3130, 2528, 1702, 1606,1466, 1438, 1383, 1350, 1281, 1236, 1129, 1095, 988, 865, 805, 760; δ_H (400 MHz, DMSO-D_6) 10.63 (s, 1H, N**H**), 9.28 (s, 1H, NC**H**S), 8.52 (d, 1H, *J* = 5.0 Hz), 8.47 – 8.36 (m, 2H), 8.30 (d, 1H, *J* = 8.0 Hz), 7.97 (dd, 1H, *J* = 9.0, 2.0 Hz), 7.90 – 7.76 (m, 2H), 7.71 (s, 1H), 7.54 (d, 1H, *J* = 3.0 Hz), 7.31 (dd, 1H, *J* = 8.0, 5.0 Hz), 4.36 (d, 1H, *J* = 13.0 Hz), 4.25 (dd, 1H, *J* = 13.0, 3.5 Hz), 4.12 (q, 2H, *J* = 16.0 Hz), 4.00 – 3.72 (m, 3H), 3.17 (d, 1H, *J* = 11.5 Hz), 3.09 – 2.91 (m, 3H), 2.69 – 2.44 (m, 2H); $^{13}C\{^1H\}$ NMR (101 MHz, DMSO-D_6) 171.1, 157.2 (N**C**HS), 152.9, 148.3, 143.2, 141.6, 139.3, 136.1, 134.7, 130.9, 129.8, 127.5, 127.3, 126.3, 125.8, 125.3, 123.4, 120.8, 119.8, 116.0, 101.2, 79.2, 66.8, 59.4, 58.6, 51.0, 50.1, 49.7; HRMS (ESI) m/z: $[M + H]^+$ calcd. for $C_{28}H_{26}N_5O_2S^+$, 496.1802; Found: 496.1820.

3-(2-(1H-Indol-3-yl)acetyl)-10-(benzo[d]thiazol-6-yl)-1,2,3,4,4a,5-hexahydrobenzo[f]pyrazino[2,1-c][1,4]oxazocin-7(12H)-one (6h)

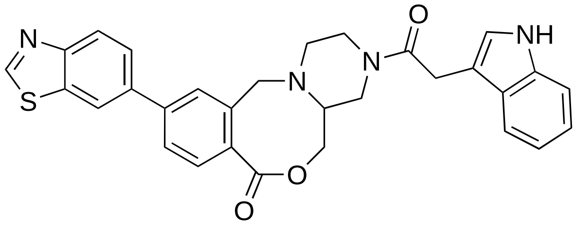

tert-Butyl 10-(benzo[d]thiazol-6-yl)-7-oxo-1,2,4a,5,7,12-hexahydrobenzo[f]pyrazino[2,1-c][1,4]oxazocine-3(4H)-carboxylate **5l** (50 mg, 0.110 mmol) was dissolved in 4 M HCl in dioxane (4.0 mL). The solution was stirred at room temperature for 1 hour, after which the solution was concentrated in vacuo to yield the salt product. This salt was dissolved in anhydrous DCM (2.0 mL), DIPEA (0.06 mL, 0.330 mmol) was added dropwise, followed by the indole acid chloride (21.1 mg, 0.120 mmol) and T3P (50% w/v in ethyl acetate, 105 mg, 0.165 mmol). The reaction solution was then stirred at room temperature for 18 hours under argon. The solution was diluted with ethyl acetate (10 mL) and washed with brine (10 mL). The aqueous layer was then extracted with ethyl acetate (3 x 10 mL). The organic layers were then combined, dried with magnesium sulphate, filtered and concentrated in vacuo to give the crude product. The product was purified by column chromatography (SiO_2, 10:1 → 3:1 dichloromethane : acetone) to afford the title compound (52.4 mg, 91%) as a grey solid. R_f = 0.53 (3:1 dichloromethane : acetone); m.p. 167–174 °C; ν_{max}/cm^{-1} (thin film) 3269, 2923, 1710, 1617, 1457, 1346, 1278, 1227, 1010, 834, 807, 743; δ_H (400 MHz, DMSO-*D₆*) 10.90 (s, 1H), 9.43 (s, 1H), 8.55 (d, 1H, *J* = 3.0 Hz), 8.17 (d, 1H, *J* = 8.5 Hz), 7.89 (dt, 1H, *J* = 8.5, 2.0 Hz), 7.74 (dd, 2H, *J* = 13.0, 7.0 Hz), 7.56 (t, 1H, *J* = 8.0 Hz), 7.48 (d, 1H, *J* = 8.0 Hz), 7.33 (d, 1H, *J* = 8.0 Hz), 7.23 (dd, 1H, *J* = 19.0, 2.0 Hz), 7.11 – 7.03 (m, 1H), 6.96 (t, 1H, *J* = 7.0 Hz), 4.30 (d, 1H, *J* = 13.0 Hz), 4.18 – 3.94 (m, 4H), 3.85 – 3.68 (m, 3H), 3.11 – 2.87 (m, 2H), 2.70 – 2.57 (m, 1H), 2.44 – 2.28 (m, 2H); $^{13}C\{^1H\}$ NMR (101 MHz, DMSO-D_6) 171.2 (both rotamers), 169.4 (rotamer A), 169.2 (rotamer B), 157.1 (N**C**HS, both), 152.8(both), 141.6(both), 139.4 (both), 136.1 (both), 134.6 (both), 130.6 (both), 130.1(both), 127.8 (rotamer A), 127.7 (rotamer B), 127.2 (rotamer A), 127.1 (rotamer B), 126.6 (both), 125.8 (both), 125.4 (both), 123.6 (rotamer A), 123.5 (rotamer B), 123.4 (both), 121.1 (both), 120.8 (both), 118.8 (rotamer A), 118.7 (rotamer B), 118.4 (both), 111.4 (both), 108.1 (rotamer A), 108.0 (rotamer B), 67.9 (rotamer A), 67.7 (rotamer B), 62.6 (rotamer A), 62.1 (rotamer B), 59.4 (rotamer A), 59.2 (rotamer B), 54.4 (rotamer A), 54.1 (rotamer B), 46.9 (rotamer A), 45.4 (rotamer B), 42.7 (rotamer A), 41.1 (rotamer B), 30.7 (rotamer A), 30.6 (rotamer B); HRMS (ESI) m/z: $[M + Na]^+$ calcd. for $C_{30}H_{26}N_4NaO_3S^+$, 545.1618; Found: 545.1611.

10-(Benzo[d]thiazol-6-yl)-3-(4-(trifluoromethyl)phenyl)-1,2,3,4,4a,5-hexahydrobenzo [f]pyrazino[2,1-c][1,4]oxazocin-7(12H)-one (6i)

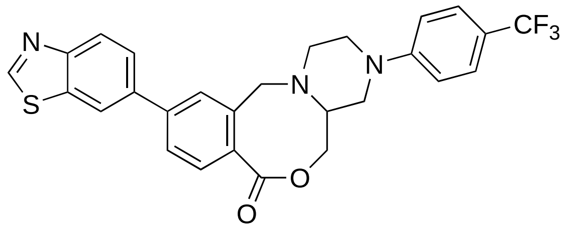

tert-Butyl 10-(benzo[d]thiazol-6-yl)-7-oxo-1,2,4a,5,7,12-hexahydrobenzo[f]pyrazino[2,1-c][1,4]oxazocine-3(4H)-carboxylate **5l** (50 mg, 0.110 mmol) was dissolved in 4 M HCl in dioxane (4.0 mL). The solution was stirred at room temperature for 1 hour, after which the solution was concentrated in vacuo to yield the salt product. This salt and 1-bromo-4-(trifluoromethyl)benzene (30.8 mg, 0.137 mmol) were dissolved in toluene (2.0 mL). Then Cs_2CO_3 (107 mg, 0.330 mmol) and (±)-BINAP (15.8 mg, 0.016 mmol) were added followed by $Pd_2(dba)_3$ (10.1 mg, 0.011 mmol) and the resulting mixture was purged with argon for 10 minutes. The mixture was stirred and heated at 110 °C for 65 hours under argon. After being allowed to cool to RT, water (10 mL) was added and the two layers were separated. The aqueous layer was extracted with ethyl acetate (3 x 10 mL) and the combined organic phases were dried with magnesium sulphate, concentrated under vacuum and purified by column chromatography (SiO_2, 3:1 → 1:1 hexane : ethyl acetate) to afford the title compound (41.1 mg, 73%) as a yellow solid. R_f = 0.50 (1:1 hexane : ethyl acetate); m.p. 145–149 °C; ν_{max}/cm^{-1} (thin film) 2836, 1718, 1613, 1525, 1466, 1330, 1237, 1162, 1113, 1071, 1012, 830, 732; δ_H (400 MHz, $CDCl_3$) 9.09 (s, 1H), 8.25 (d, 1H, *J* = 8.5 Hz), 8.19 (d, 1H, *J* = 2.0 Hz), 7.76 (dd, 1H, *J* = 8.5, 2.0 Hz), 7.64 (s, 2H), 7.53 (s, 1H), 7.49 (d, 2H, *J* = 8.5 Hz), 6.93 (d, 2H, *J* = 8.5 Hz), 4.26 (dd, 1H, *J* = 13.0, 2.0 Hz), 4.18 (dd, 1H, *J* = 13.0, 4.0 Hz), 4.01 – 3.87 (m, 2H), 3.69 – 3.61 (m, 1H), 3.54 (dt, 1H, *J* = 11.5, 2.5 Hz), 3.12 – 2.93 (m, 3H), 2.92 – 2.81 (m, 2H); $^{13}C\{^1H\}$ NMR (101 MHz, $CDCl_3$) 171.8, 155.3, 152.9, 143.5, 138.8, 137.6, 134.8, 131.1, 128.2, 127.5, 127.0, 126.6 (q, J_{F-C} = 35.9 Hz), 126.1, 126.0, 124.0, 121.2, 120.6, 115.1, 69.2, 62.8, 60.6, 55.0, 50.3, 48.3. Note: The CF_3 signal is not reported as it could not be confidently assigned, due to several overlapping signals in the region it would be expected to appear (ca. 125 ppm); HRMS (ESI) m/z: $[M + H]^+$ calcd. for $C_{27}H_{23}F_3N_3O_2S^+$, 510.1458; Found: 510.1464.

10-(Benzo[d]thiazol-6-yl)-3-(5-nitropyridin-2-yl)-1,2,3,4,4a,5-hexahydrobenzo[f] pyrazino[2,1-c][1,4]oxazocin-7(12H)-one (6j)

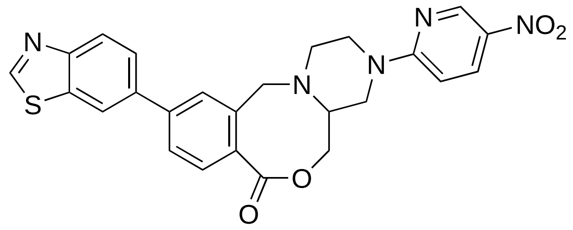

tert-Butyl 10-(benzo[d]thiazol-6-yl)-7-oxo-1,2,4a,5,7,12-hexahydrobenzo[f]pyrazino[2,1-c][1,4]oxazocine-3(4H)-carboxylate **5l** (50 mg, 0.110 mmol) was dissolved in 4 M HCl in dioxane (4.0 mL). The solution was stirred at room temperature for 1 hour, after which the solution was concentrated in vacuo to yield the salt product. This salt was dissolved in anhydrous MeCN (3.0 mL), 2-chloro-5-nitropyridine (21.9 mg, 0.138 mmol) was added followed by potassium carbonate (45.5 mg, 0.329 mmol). The reaction mixture was heated to 70 °C for 18 hours under argon. Then the reaction mixture was allowed to cool to RT, filtered and washed with dichloromethane, concentrated under vacuum and purified by column chromatography (SiO_2, 2:1 → 1:1 hexane : ethyl acetate) to afford the title compound (44.1 mg, 82%) as a white solid. R_f = 0.22 (1:1 hexane : ethyl acetate); m.p. 256–258 °C; ν_{max}/cm^{-1} (thin film) 3061, 2837, 1716, 1574, 1512, 1486, 1468, 1430, 1334, 1314, 1298, 1266, 1234,1095, 1085, 999, 977, 866, 822, 802, 762, 658 ; δ_H (400 MHz, DMSO-*D₆*) 9.45 (s, 1H), 8.98 (d, 1H, *J* = 3.0 Hz), 8.59 (d, 1H, *J* = 2.0 Hz), 8.26 (dd, 1H, *J* = 9.5, 3.0 Hz), 8.20 (d, 1H, *J* = 8.5 Hz), 7.92 (dd, 1H, *J* = 8.5, 2.0 Hz), 7.83 – 7.75 (m, 2H), 7.53 (d, 1H, *J* = 8.5 Hz), 7.02 (d, 1H, *J* = 9.5 Hz), 4.59 – 4.36 (m, 2H), 4.26 (dd, 1H, *J* = 13.0, 2.5 Hz), 4.15 (dd, 1H, *J* = 13.0, 3.5 Hz), 4.08 (d, 1H, *J* = 16.5 Hz), 3.86 (d, 1H, *J* = 16.5 Hz), 3.19 – 3.04 (m, 3H), 2.74 – 2.60 (m, 2H); $^{13}C\{^1H\}$ NMR (101 MHz, DMSO-D_6) 171.3, 160.0, 157.1, 152.9, 146.0, 141.7, 139.5, 136.2, 134.7, 134.4, 132.9, 130.6, 127.9, 126.7, 125.9, 125.4, 123.4, 120.8, 105.8, 68.0, 62.0, 59.2, 53.7, 44.5 ; HRMS (ESI) m/z: $[M + H]^+$ calcd. for $C_{25}H_{22}N_5O_4S^+$, 488.1387; Found: 488.1398.

3) ^{1}H and ^{13}C{^{1}H} NMR Spectra

In this section, all ^{1}H NMR spectra are 400 MHz and all ^{13}C{^{1}H} NMR spectra are 101 MHz.

Methyl 2-bromo-6-(bromomethyl)benzoate (7a) - δ_H (400 MHz) and ^{13}C{^{1}H} NMR (101 MHz)

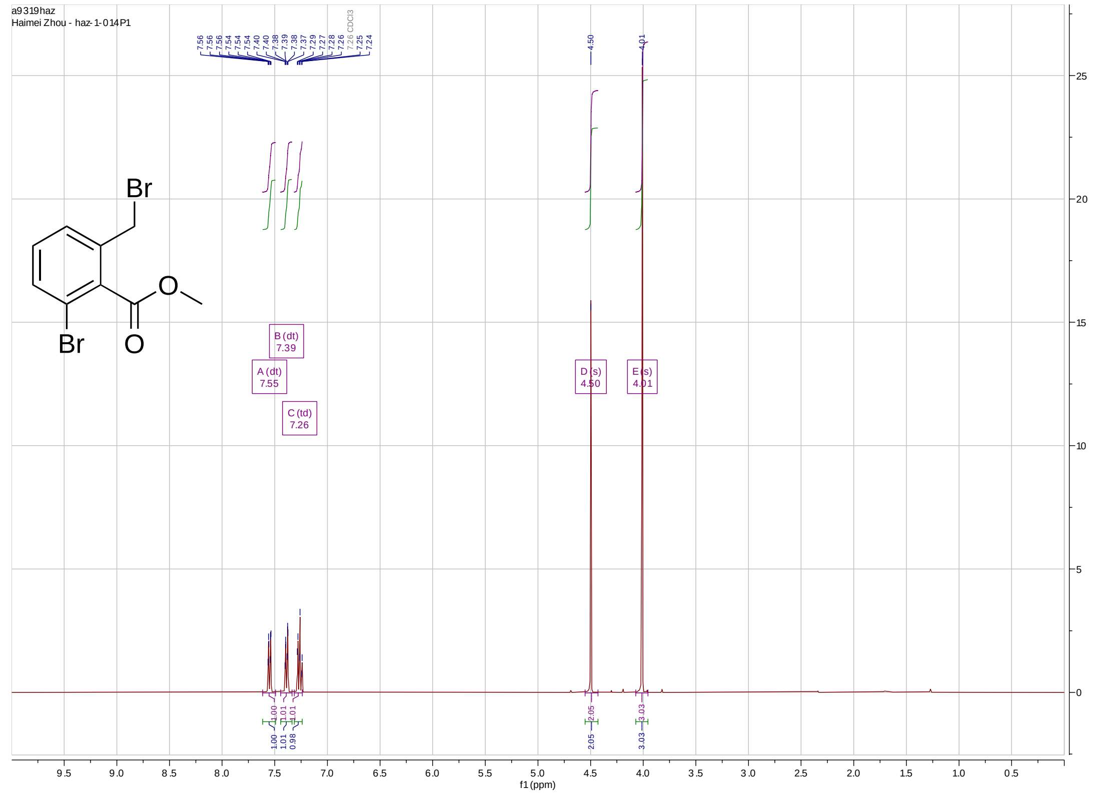

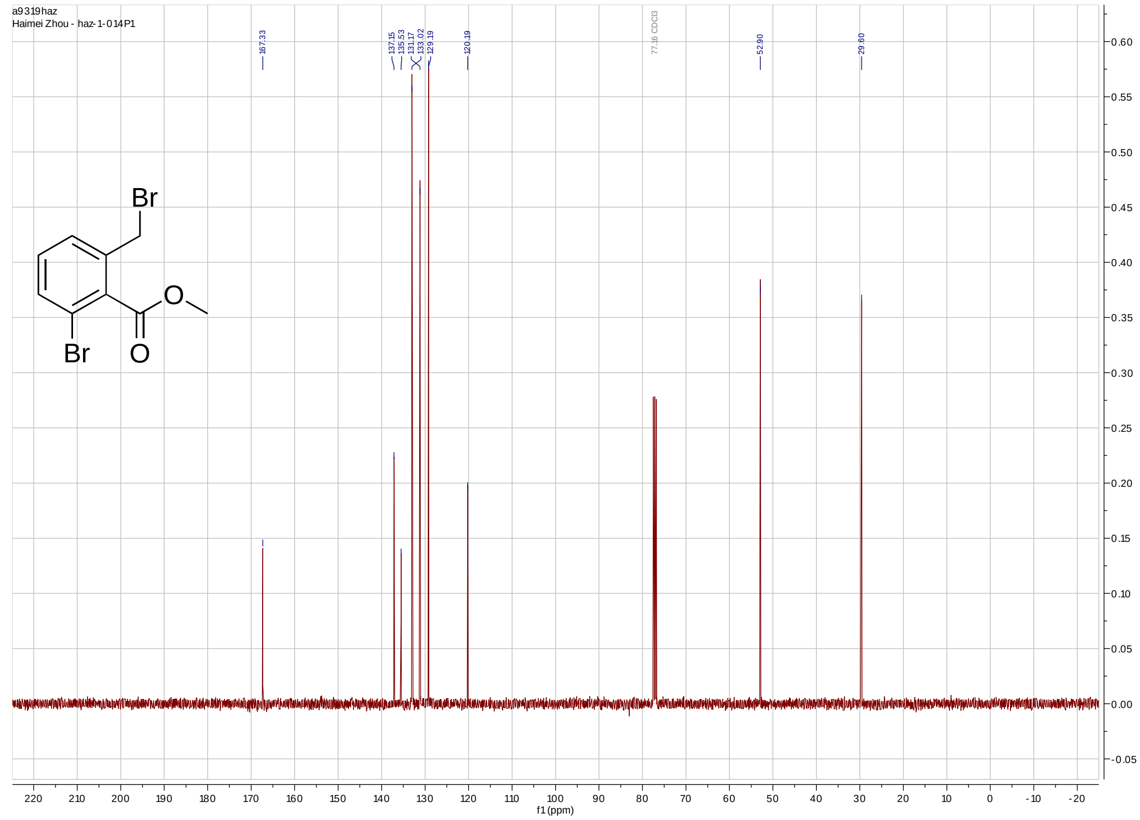

Methyl 5-bromo-2-(bromomethyl)benzoate (7b) - δ_H (400 MHz) and $^{13}C\{^1H\}$ NMR (101 MHz)

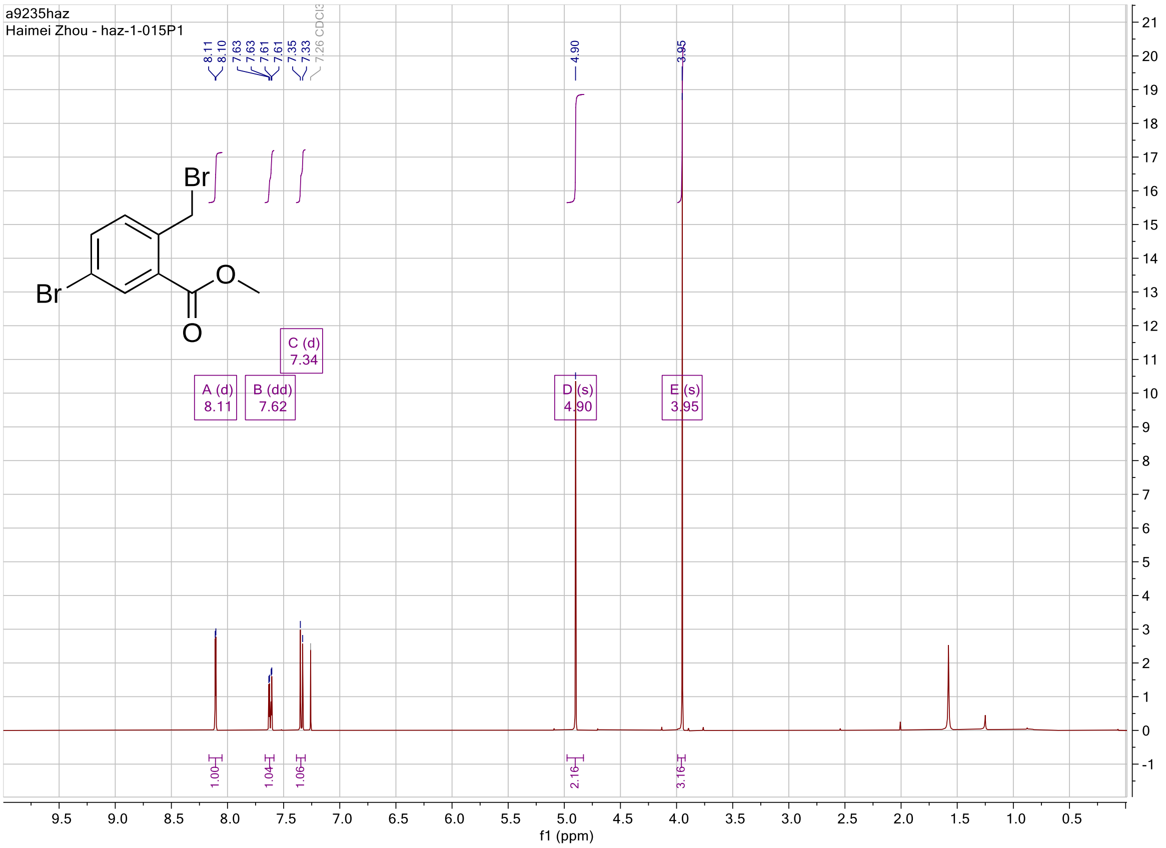

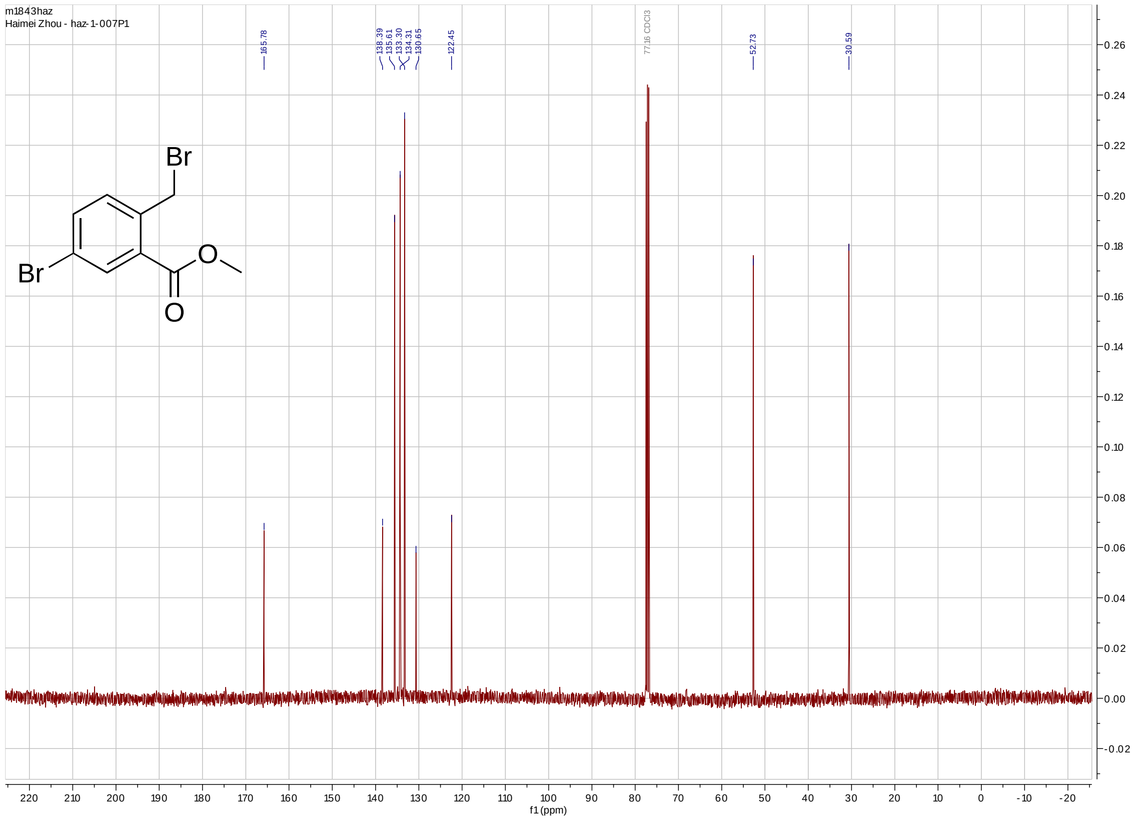

Methyl 4-bromo-2-(bromomethyl)benzoate (7c) - δ_H (400 MHz) and $^{13}C\{^1H\}$ NMR (101 MHz)

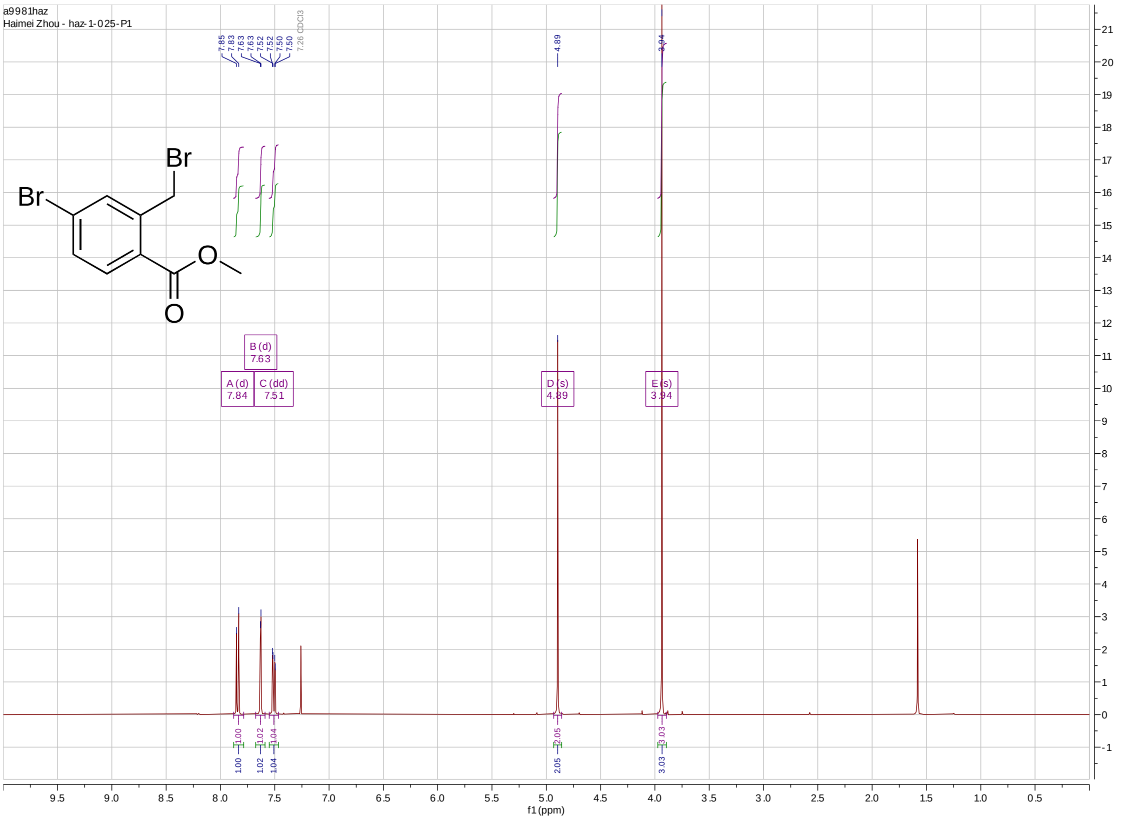

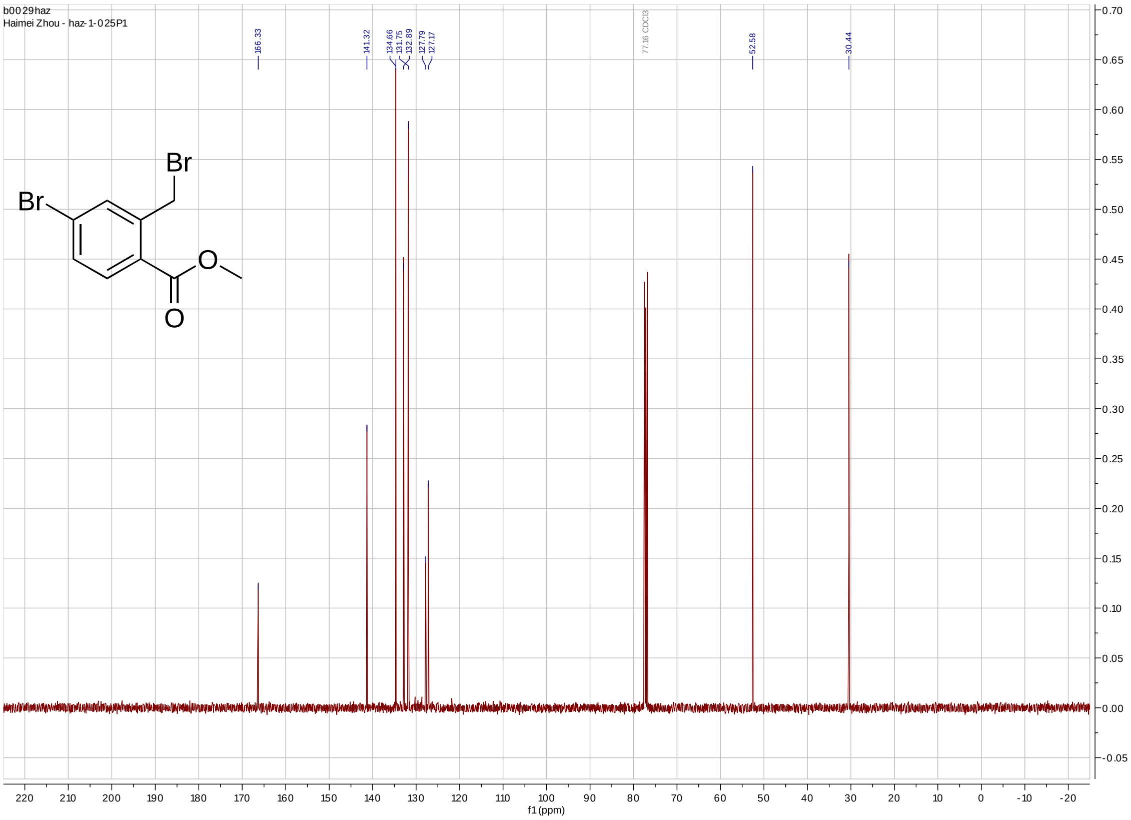

Methyl 3-bromo-2-(bromomethyl)benzoate (7d) - δ_H (400 MHz) and $^{13}C\{^1H\}$ NMR (101 MHz)

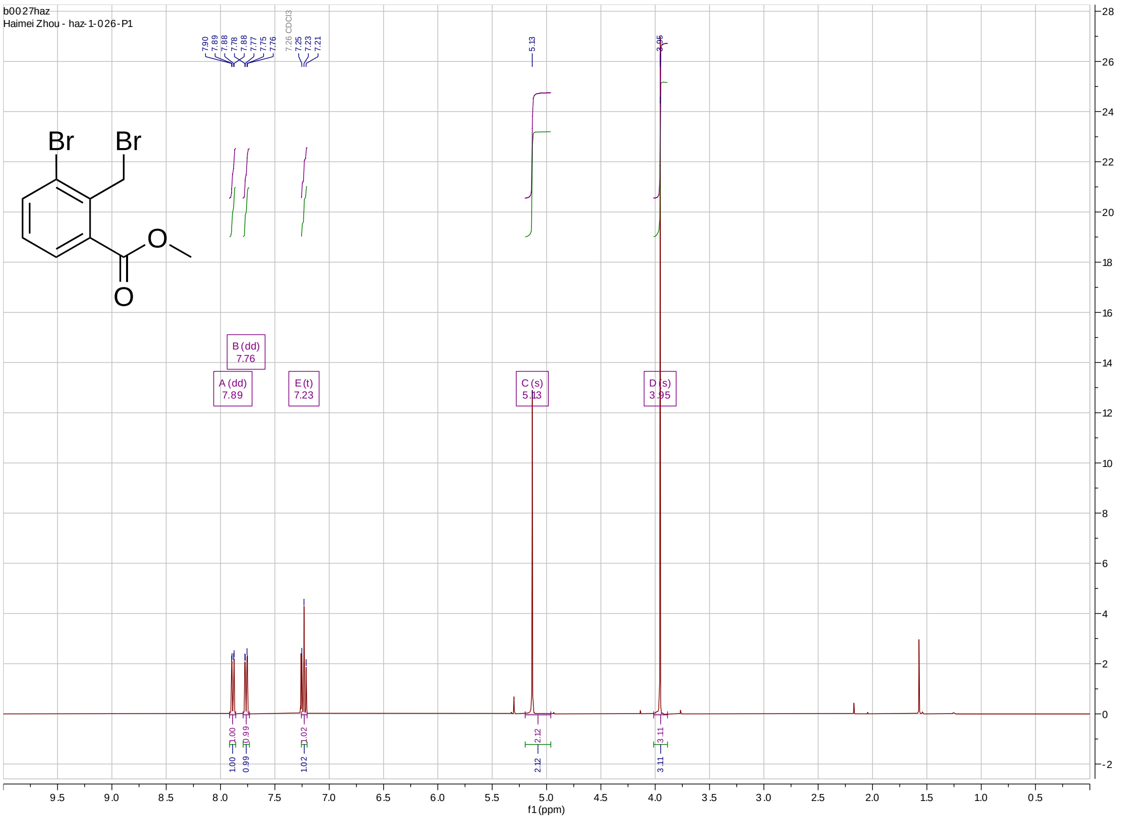

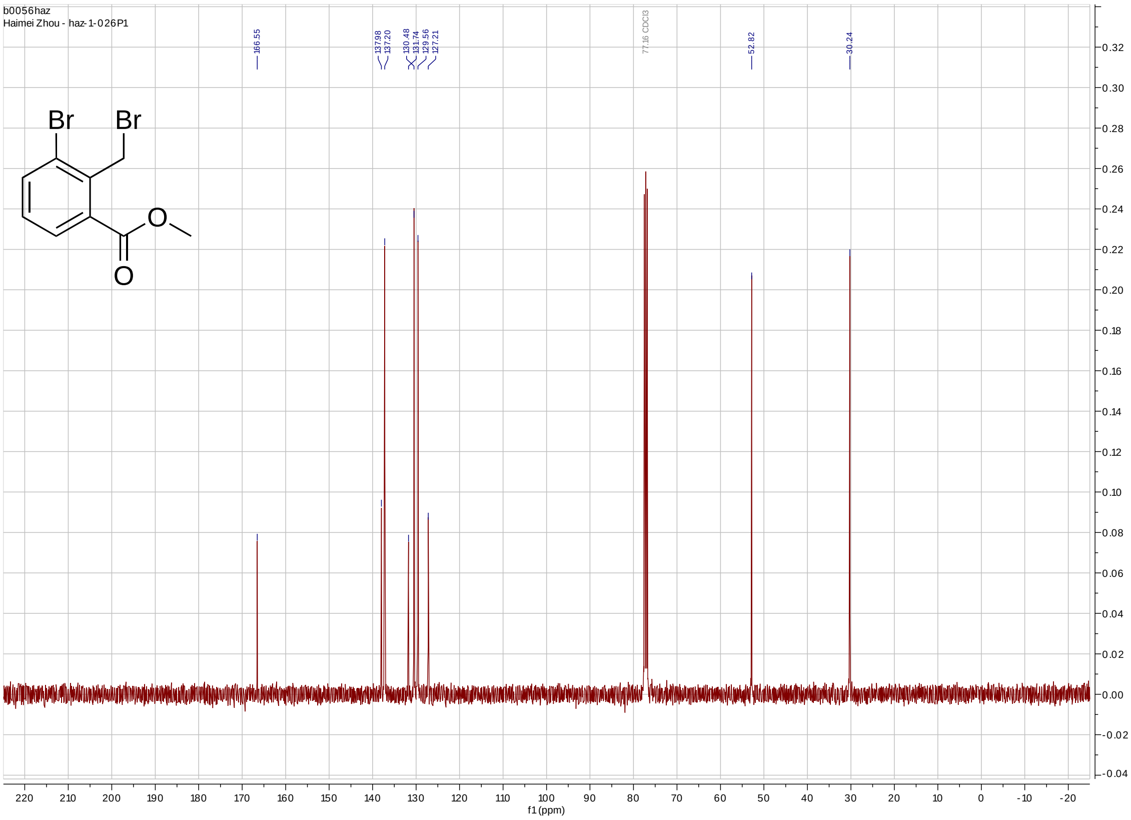

Methyl 2-bromo-6-((2-(hydroxymethyl)piperidin-1-yl)methyl)benzoate (8a) - δ_H (400 MHz) and $^{13}C\{^1H\}$ NMR (101 MHz)

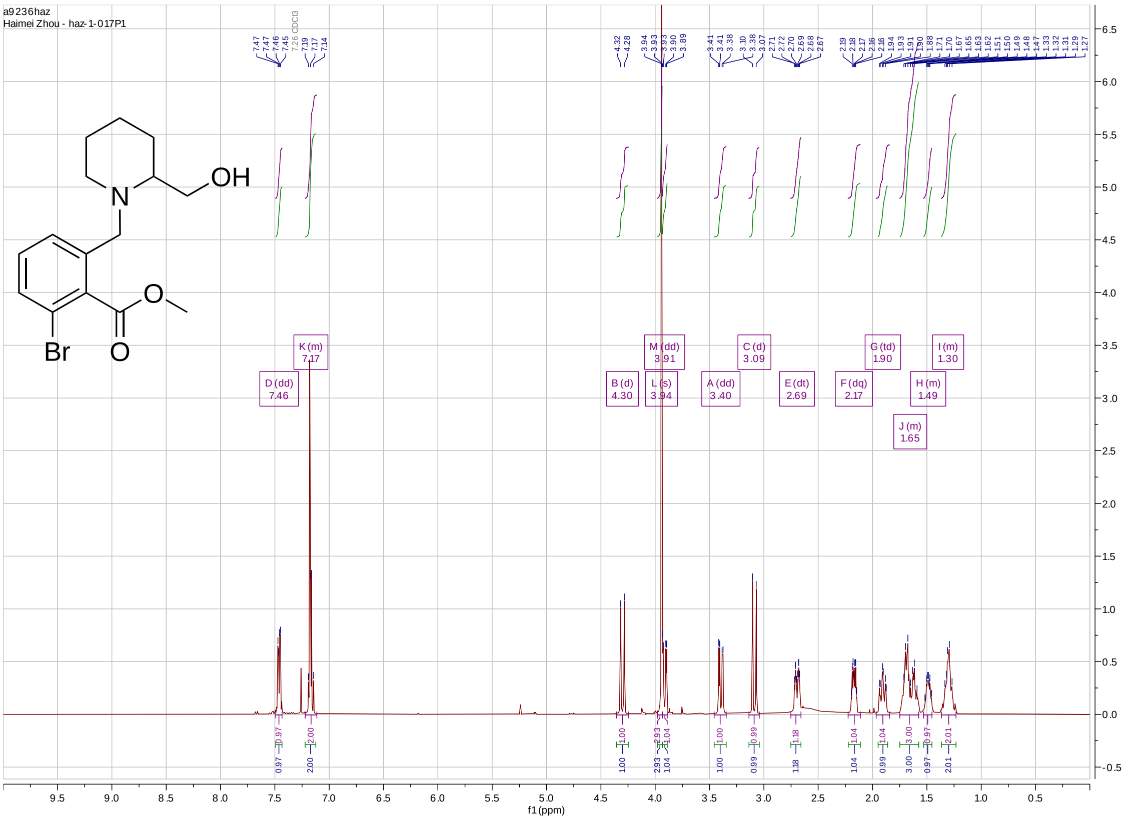

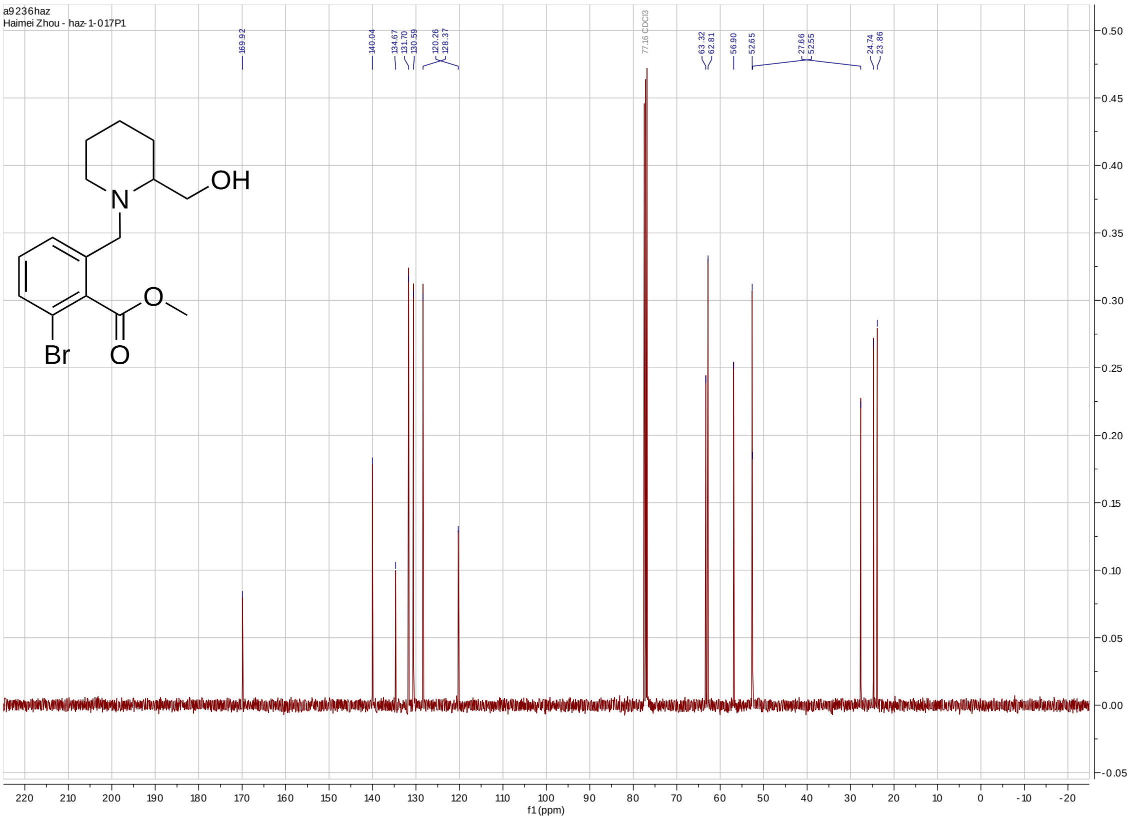

Methyl 5-bromo-2-((2-(hydroxymethyl)piperidin-1-yl)methyl)benzoate (8b) - δ_H (400 MHz) and $^{13}C\{^1H\}$ NMR (101 MHz)

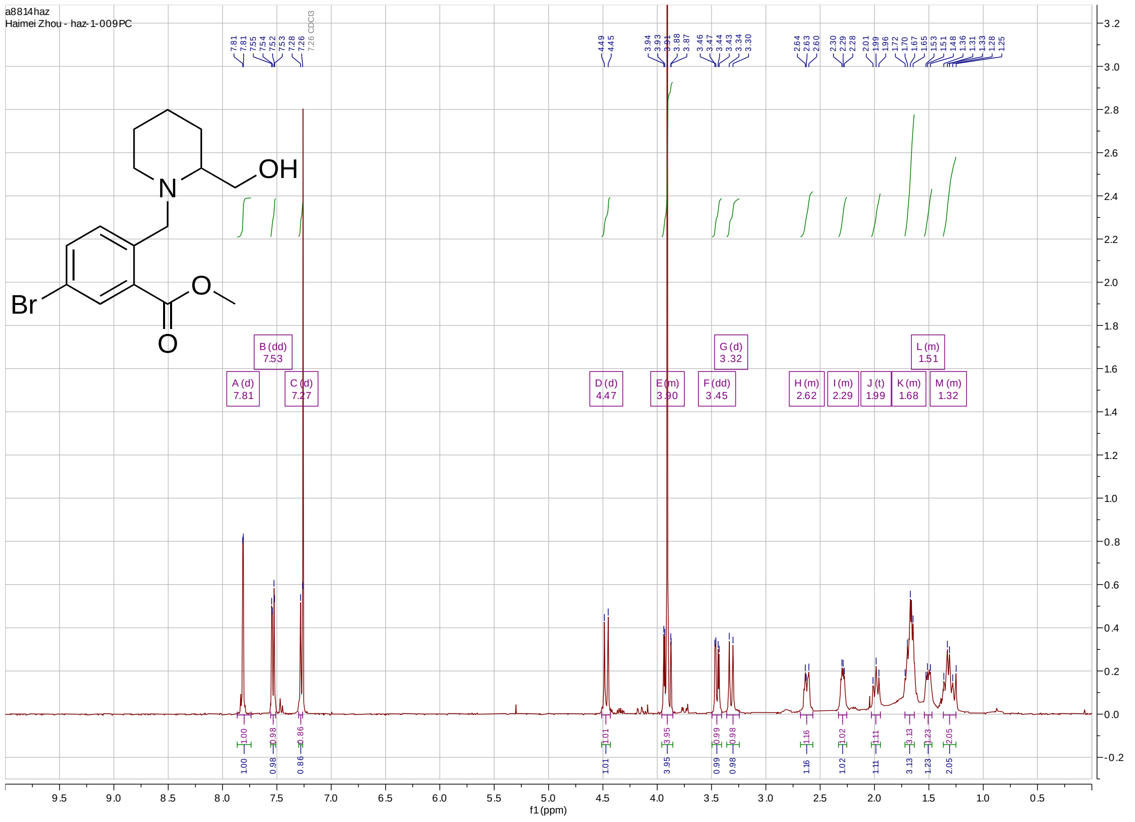

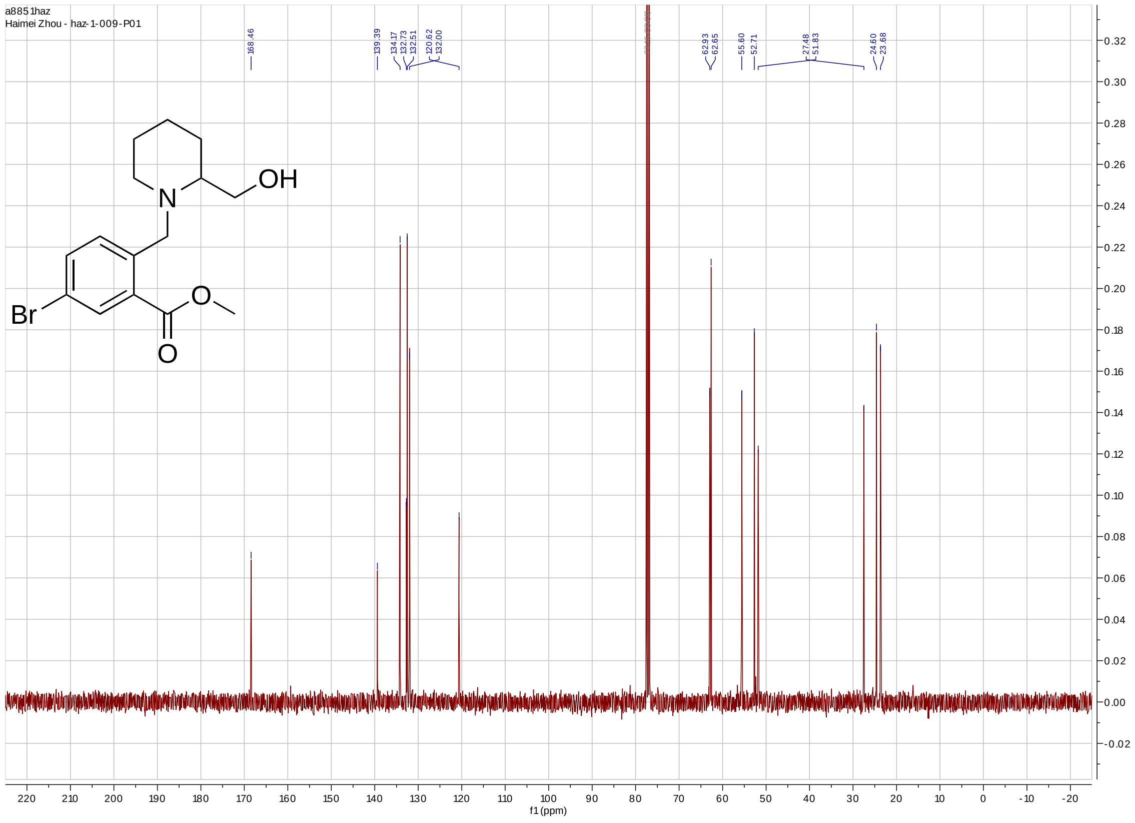

Methyl 4-bromo-2-((2-(hydroxymethyl)piperidin-1-yl)methyl)benzoate (8c) - δ_H (400 MHz) and $^{13}C\{^1H\}$ NMR (101 MHz)

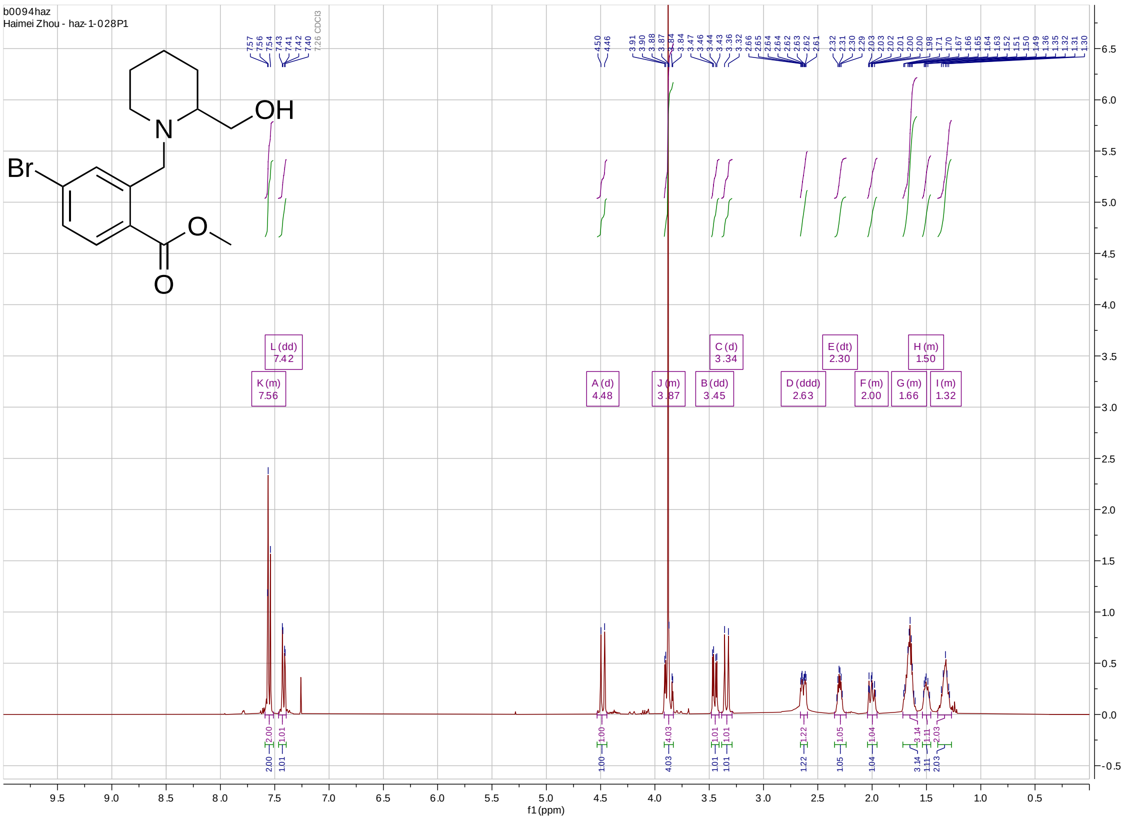

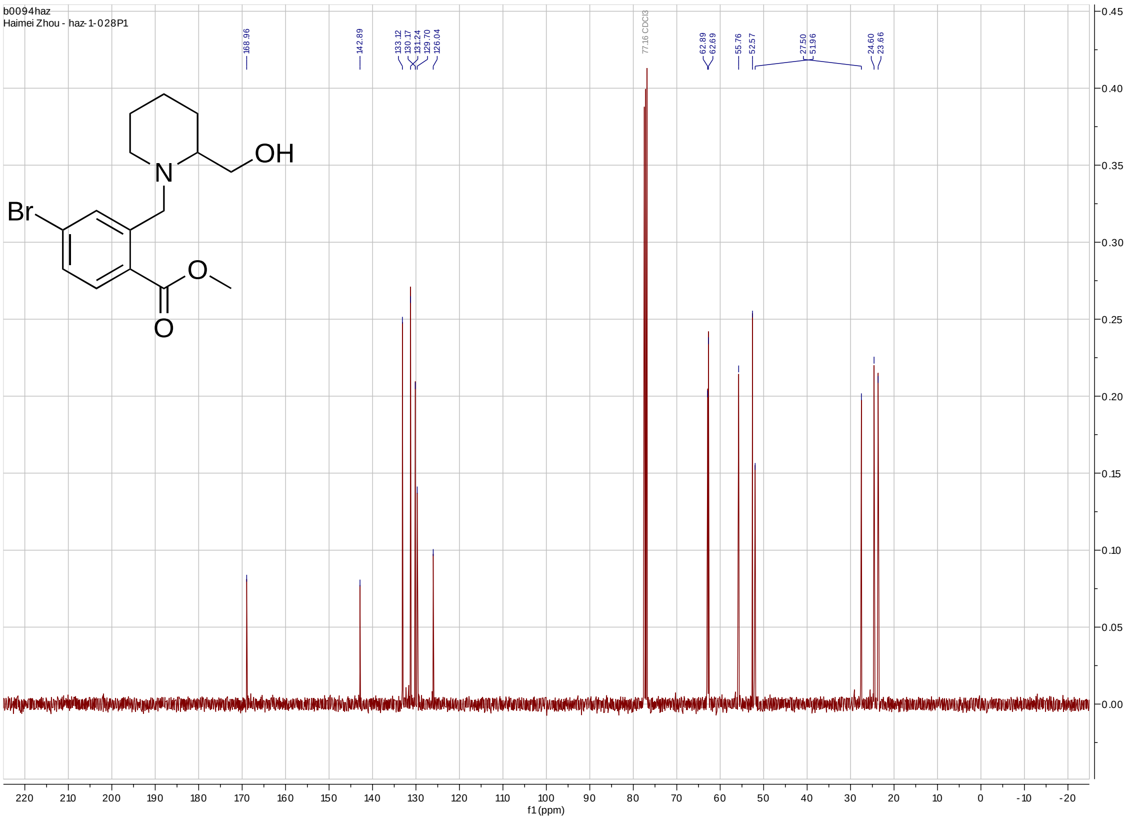

Methyl 3-bromo-2-((2-(hydroxymethyl)piperidin-1-yl)methyl)benzoate (8d) - δ_H (400 MHz) and $^{13}C\{^1H\}$ NMR (101 MHz)

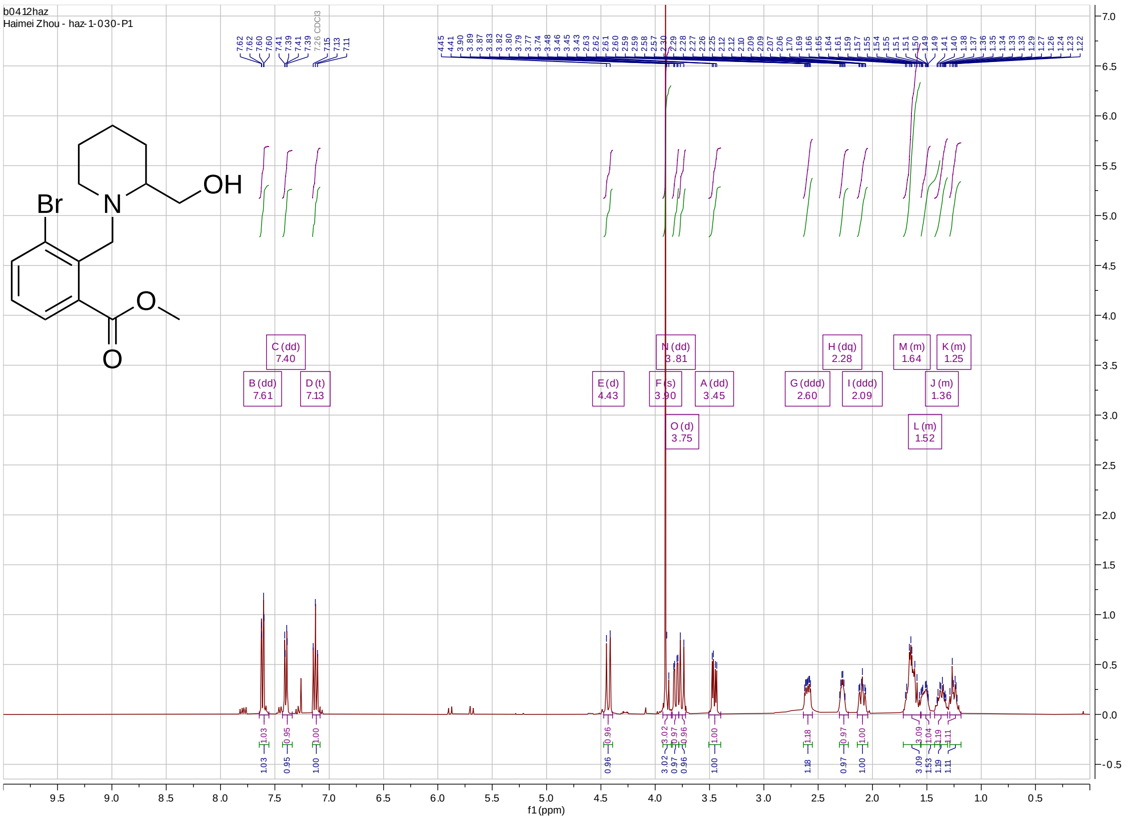

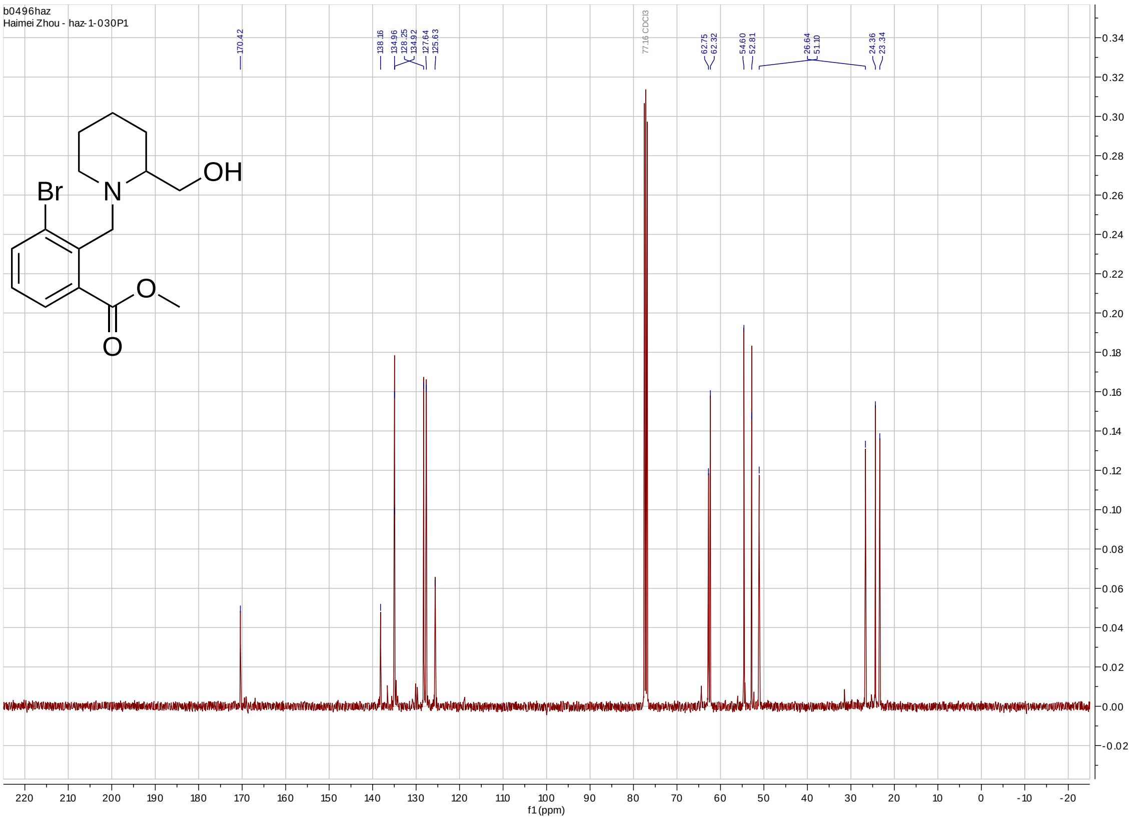

4-Bromo-7,7a,8,9,10,11-hexahydrobenzo[f]pyrido[2,1-c][1,4]oxazocin-5(13H)-one (4a) - δ_H (400 MHz) and $^{13}C\{^1H\}$ NMR (101 MHz)

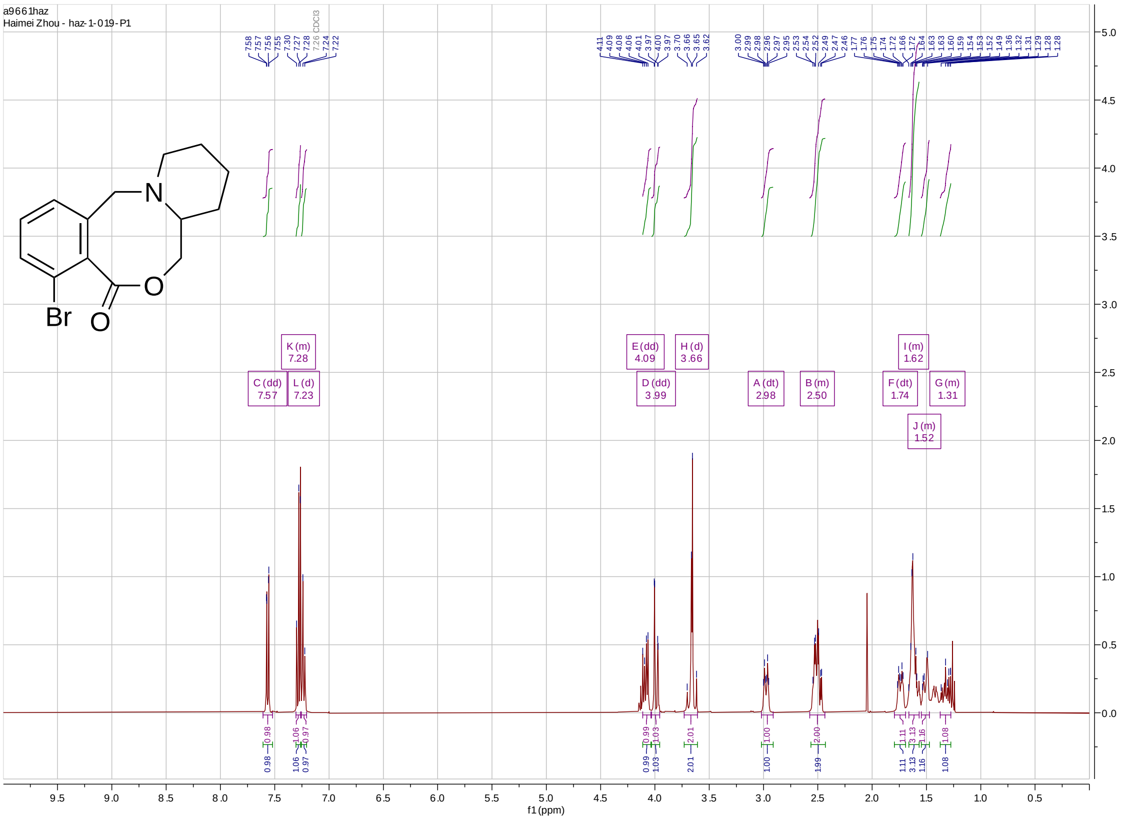

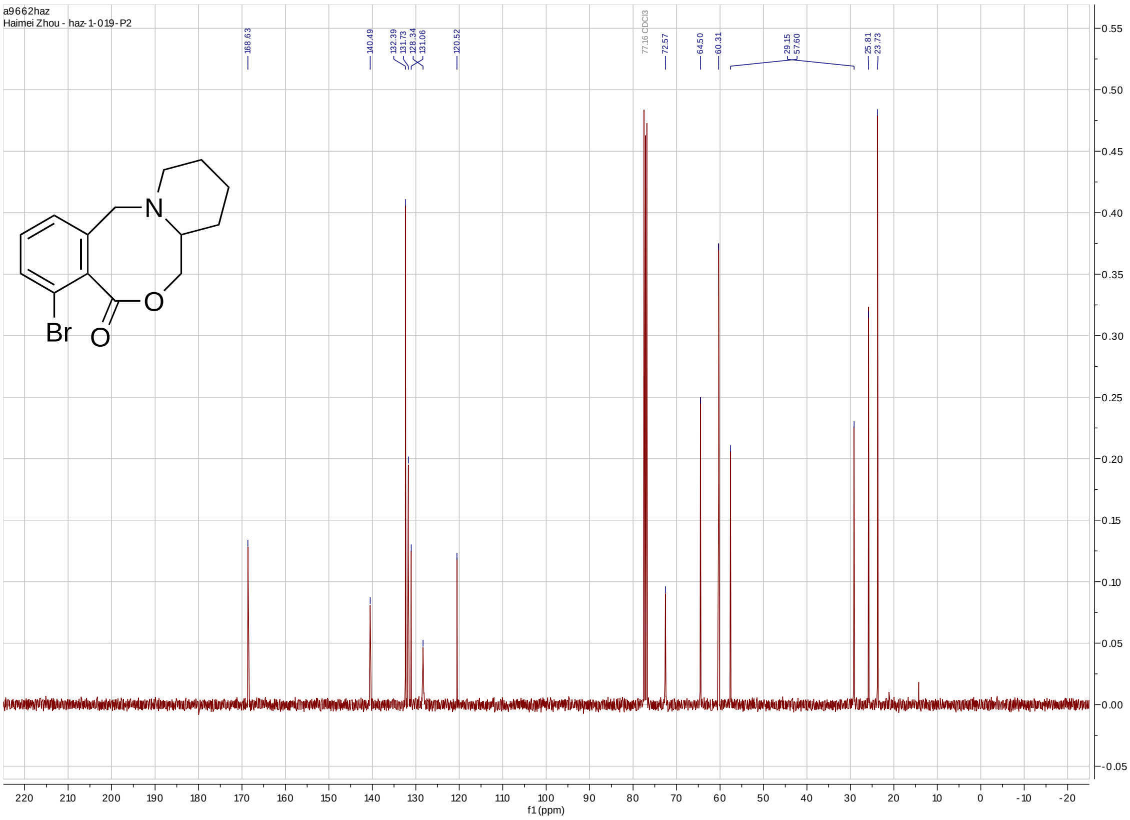

3-Bromo-7,7a,8,9,10,11-hexahydrobenzo[f]pyrido[2,1-c][1,4]oxazocin-5(13H)-one (4b) - δ_H (400 MHz) and $^{13}C\{^1H\}$ NMR (101 MHz)

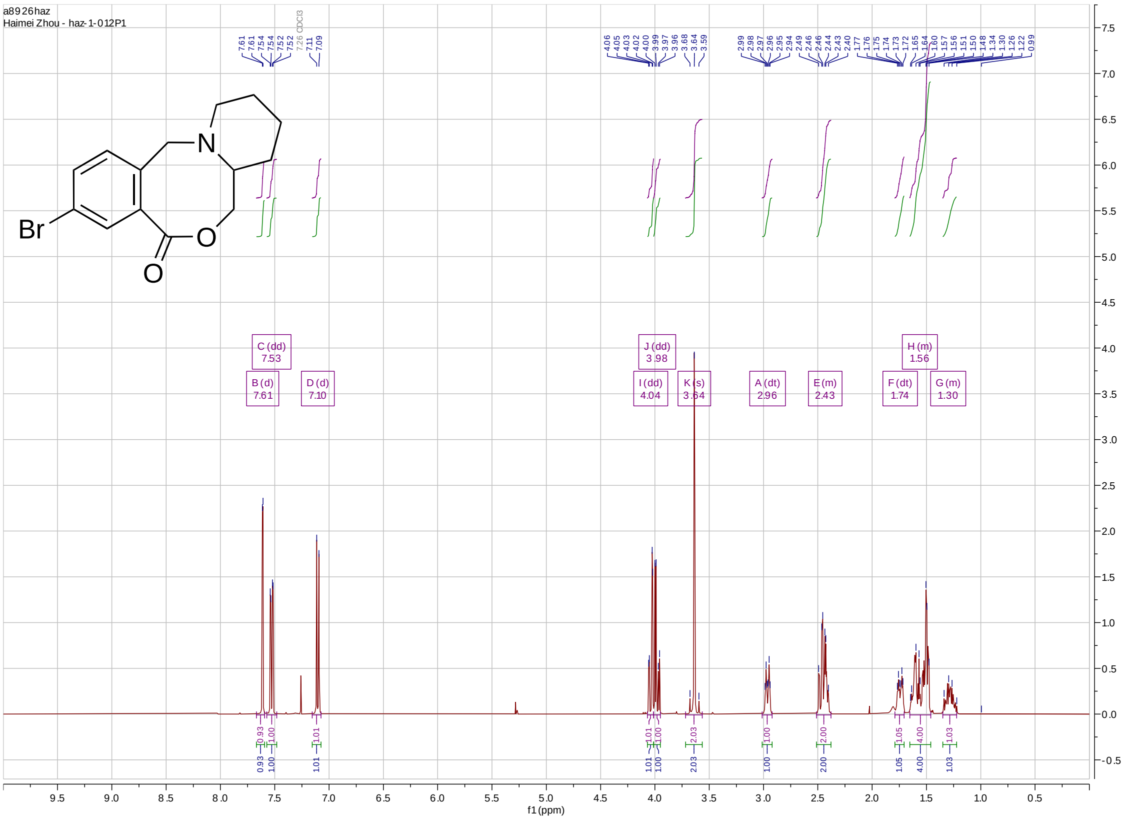

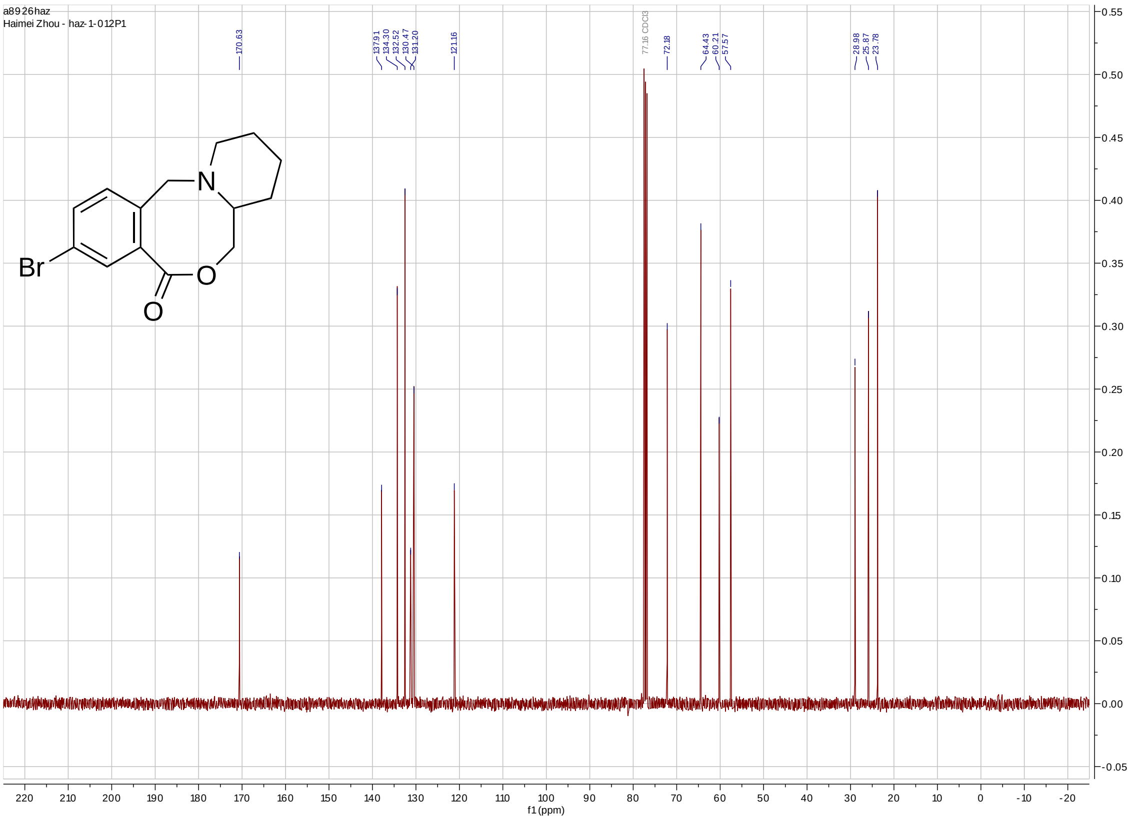

2-Bromo-7,7a,8,9,10,11-hexahydrobenzo[f]pyrido[2,1-c][1,4]oxazocin-5(13H)-one (4c) - δ_H (400 MHz) and $^{13}C\{^1H\}$ NMR (101 MHz)

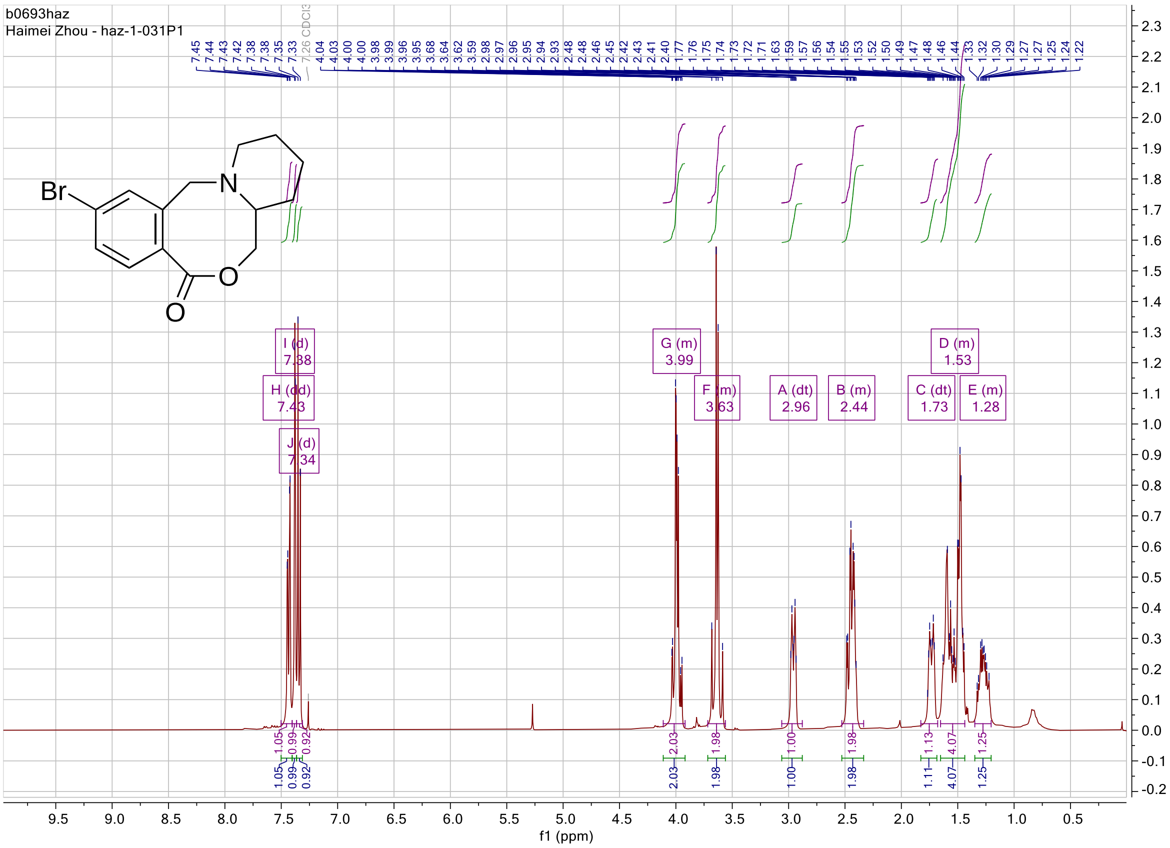

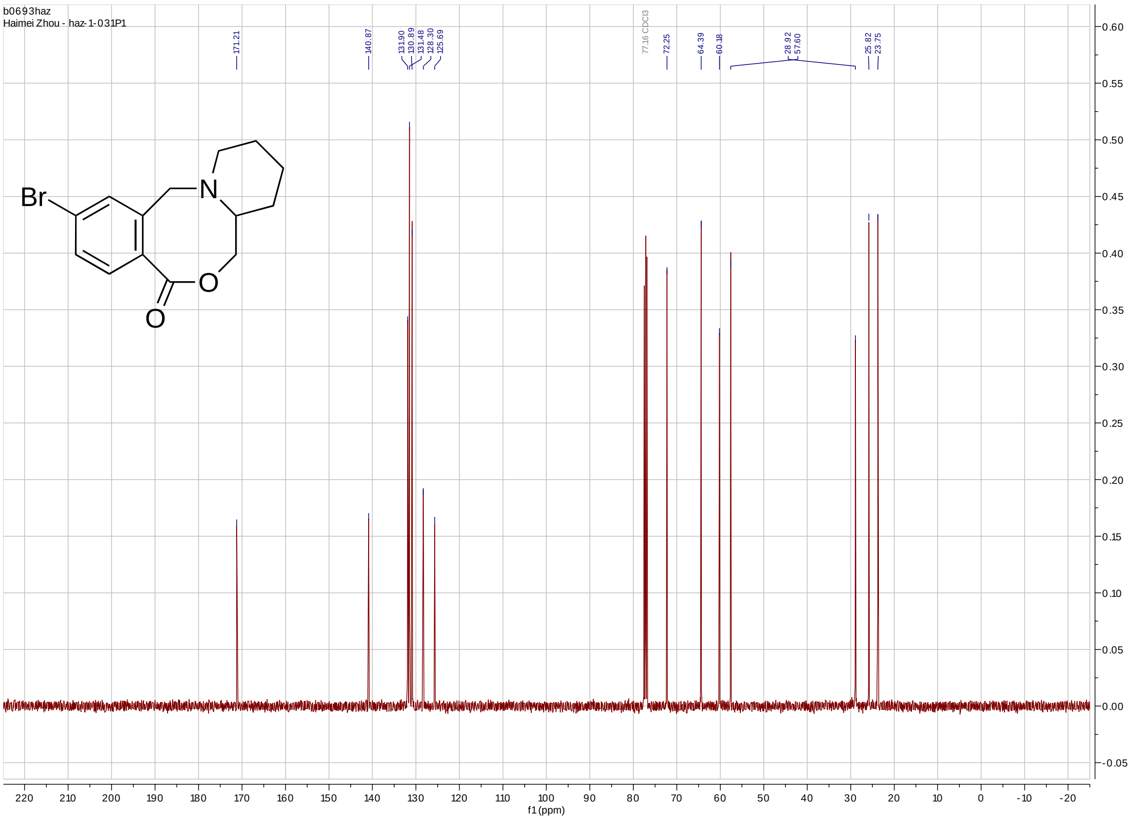

1-Bromo-7,7a,8,9,10,11-hexahydrobenzo[f]pyrido[2,1-c][1,4]oxazocin-5(13H)-one (4d) - δ_H (400 MHz) and $^{13}C\{^1H\}$ NMR (101 MHz)

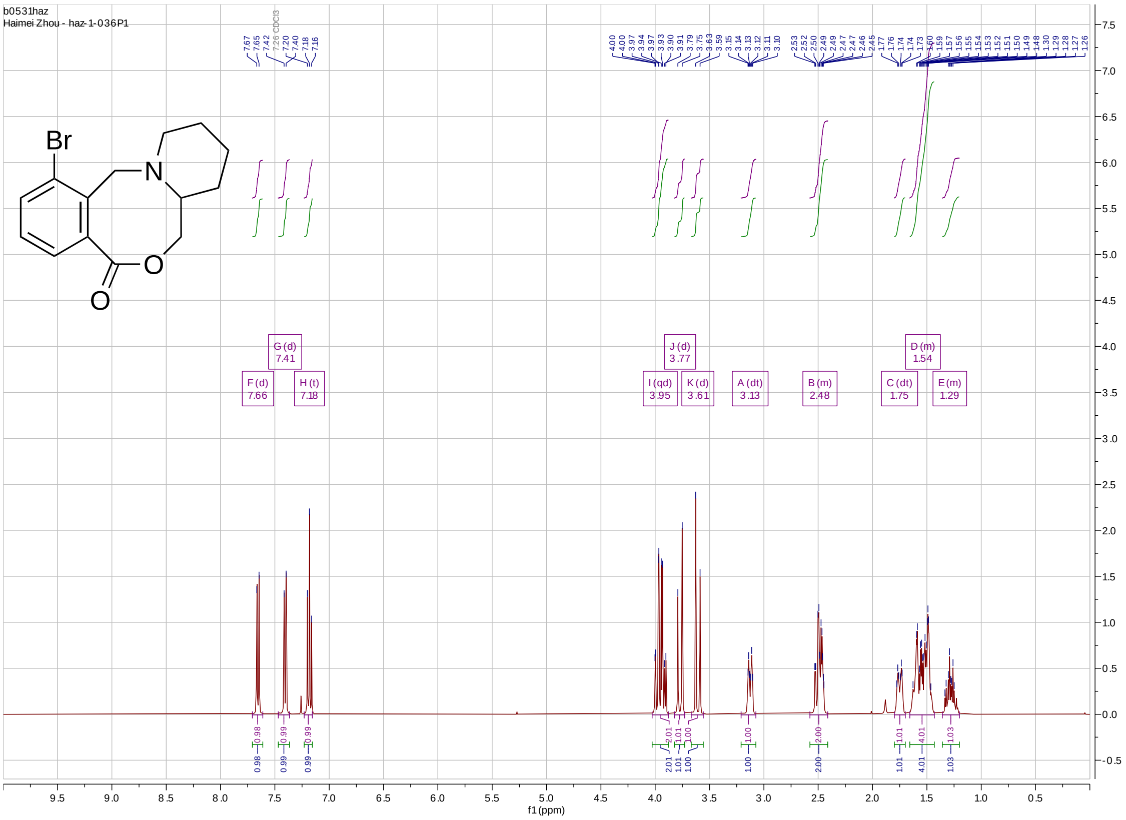

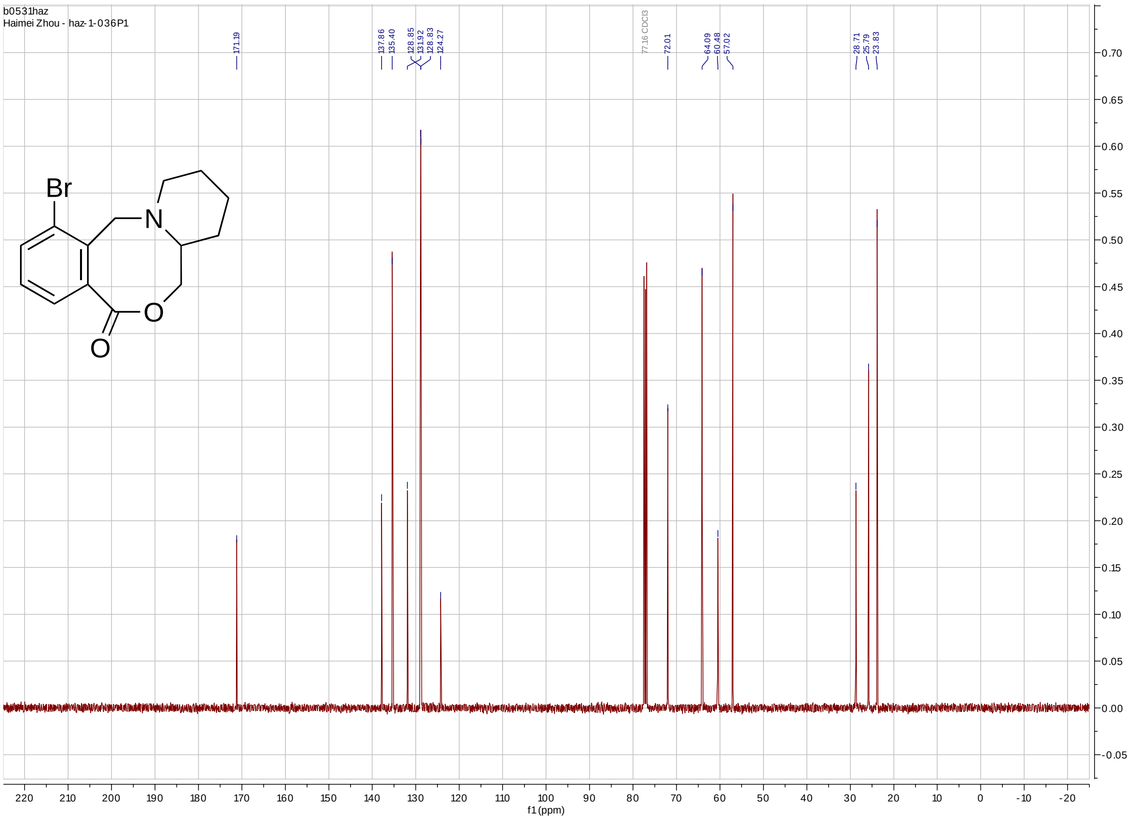

Methyl (S)-5-bromo-2-((2-(hydroxymethyl)pyrrolidin-1-yl)methyl)benzoate (8e) - δ_H (400 MHz) and $^{13}C\{^1H\}$ NMR (101 MHz)

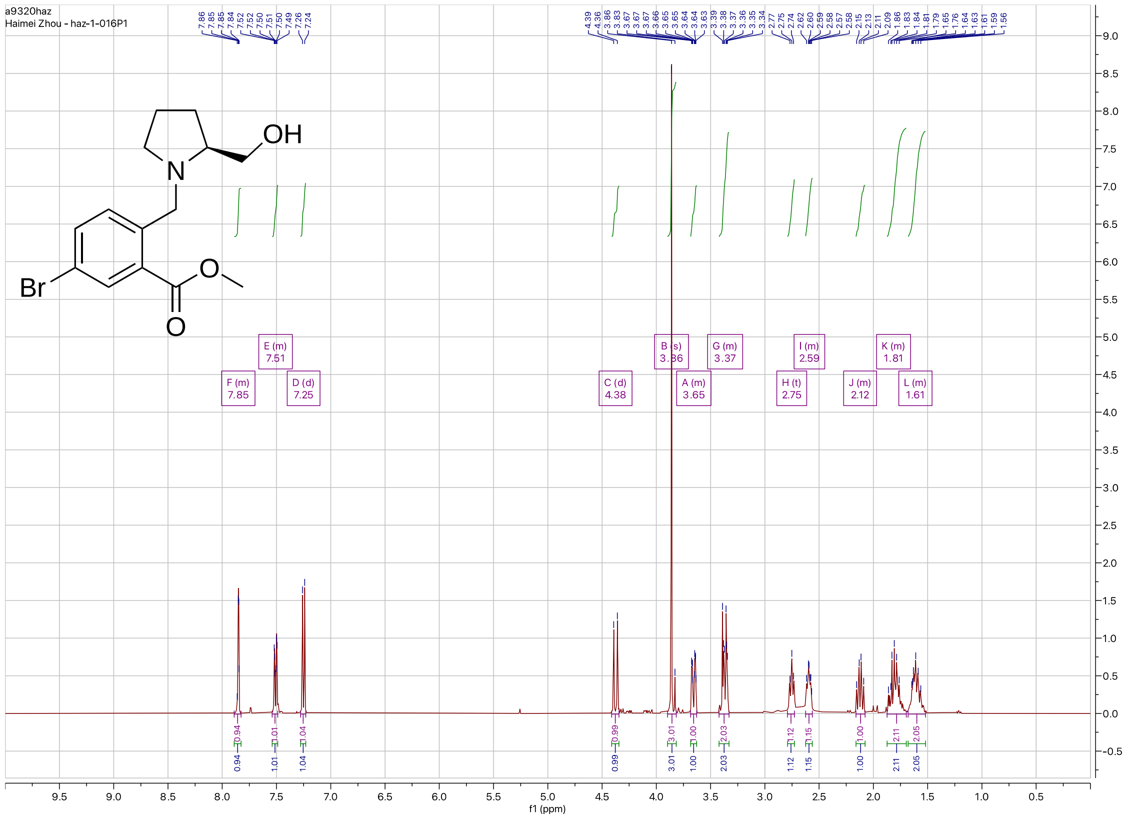

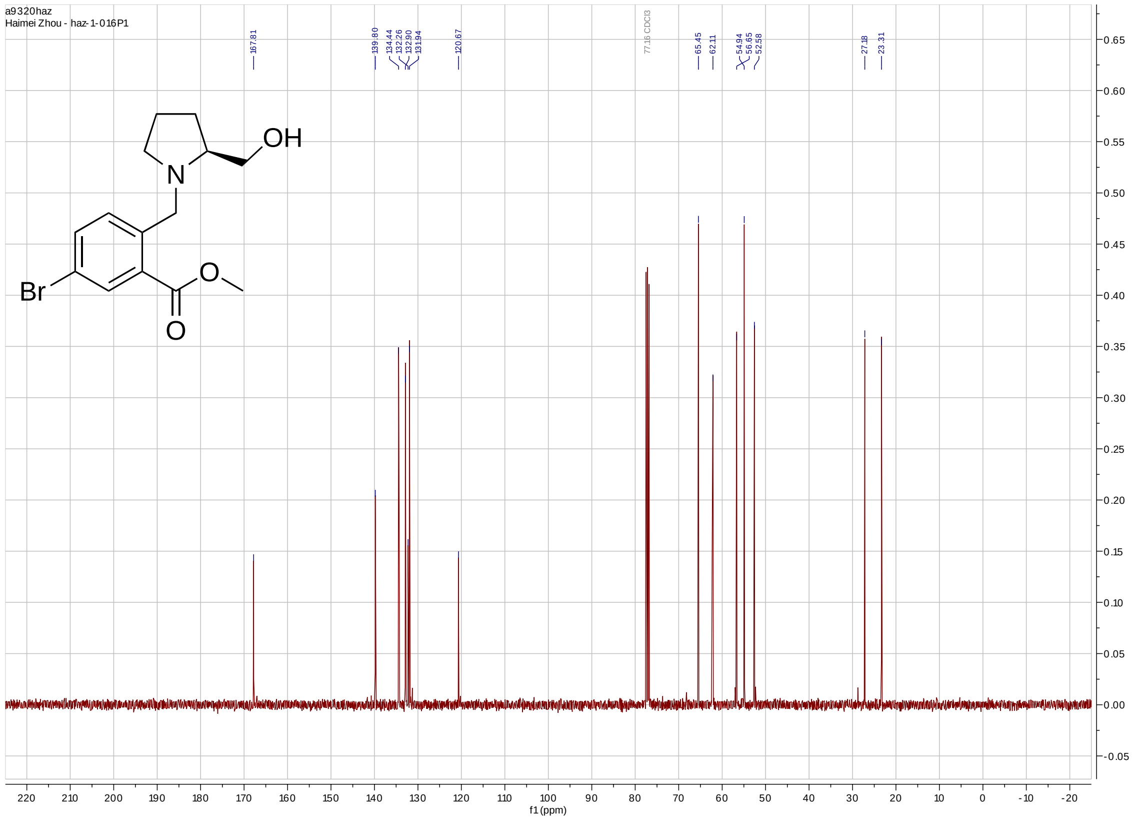

8-Bromo-2,3,3a,4-tetrahydro-1H-benzo[f]pyrrolo[2,1-c][1,4]oxazocin-6(11H)-one (4e) - δ_H (400 MHz) and $^{13}C\{^1H\}$ NMR (101 MHz)

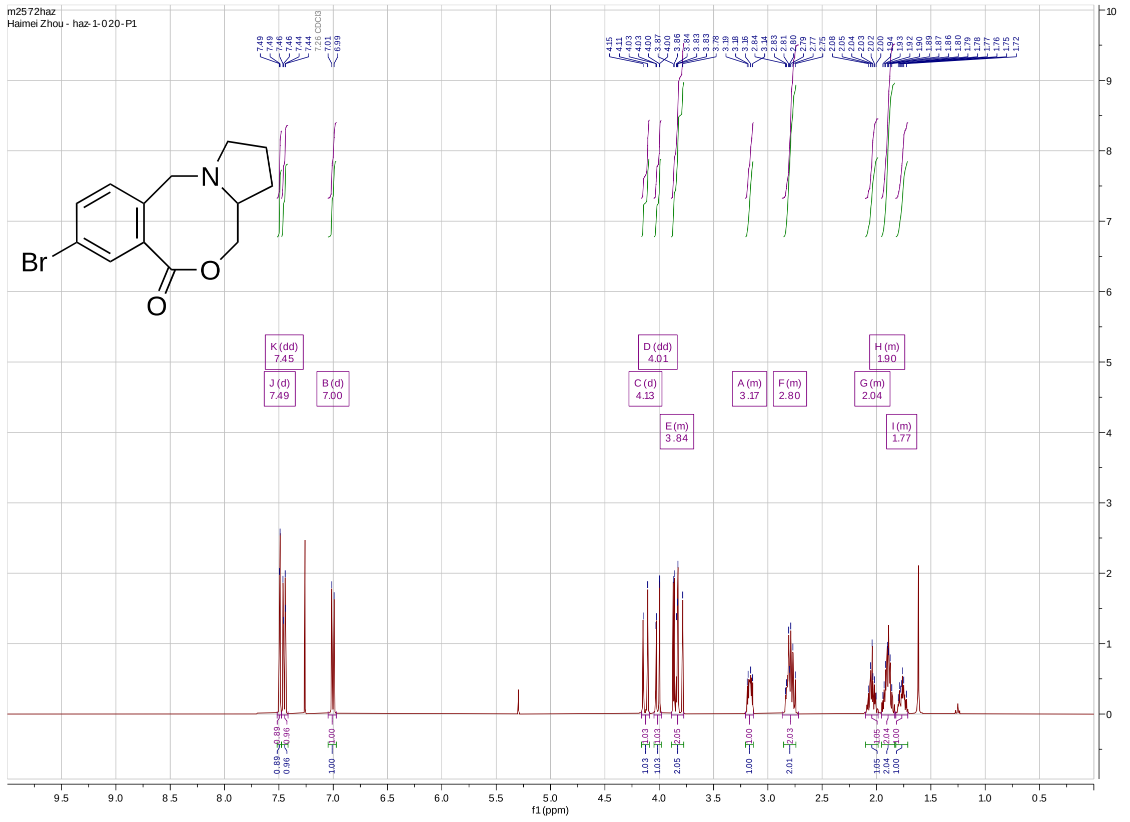

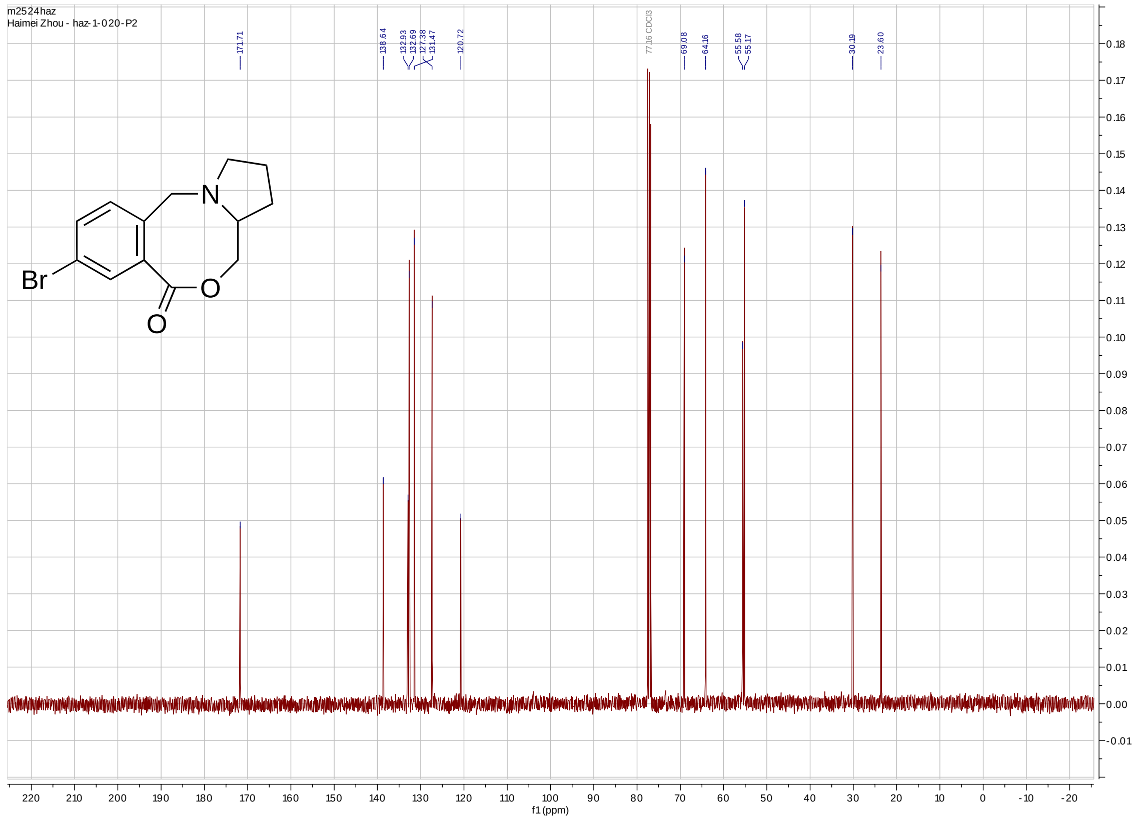

Methyl 5-bromo-2-((2-(2-hydroxyethyl)piperidin-1-yl)methyl)benzoate (8f) - δ_H (400 MHz) and $^{13}C\{^1H\}$ NMR (101 MHz)

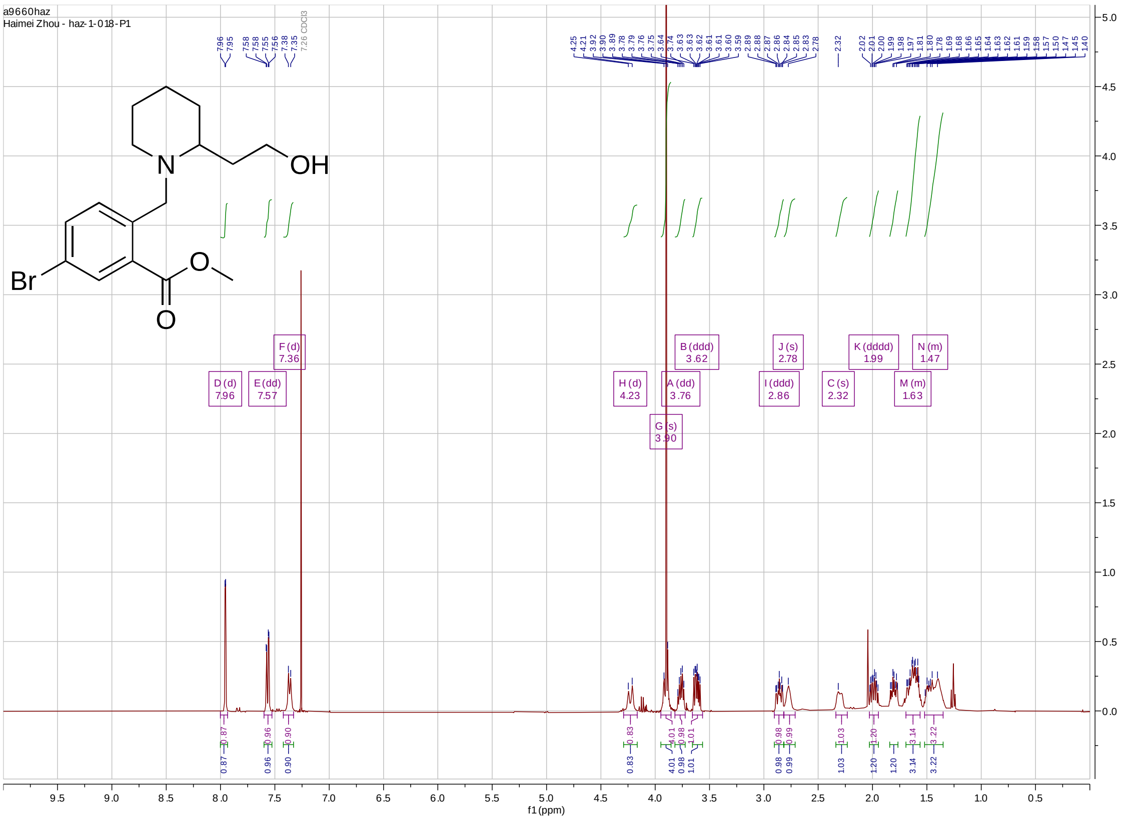

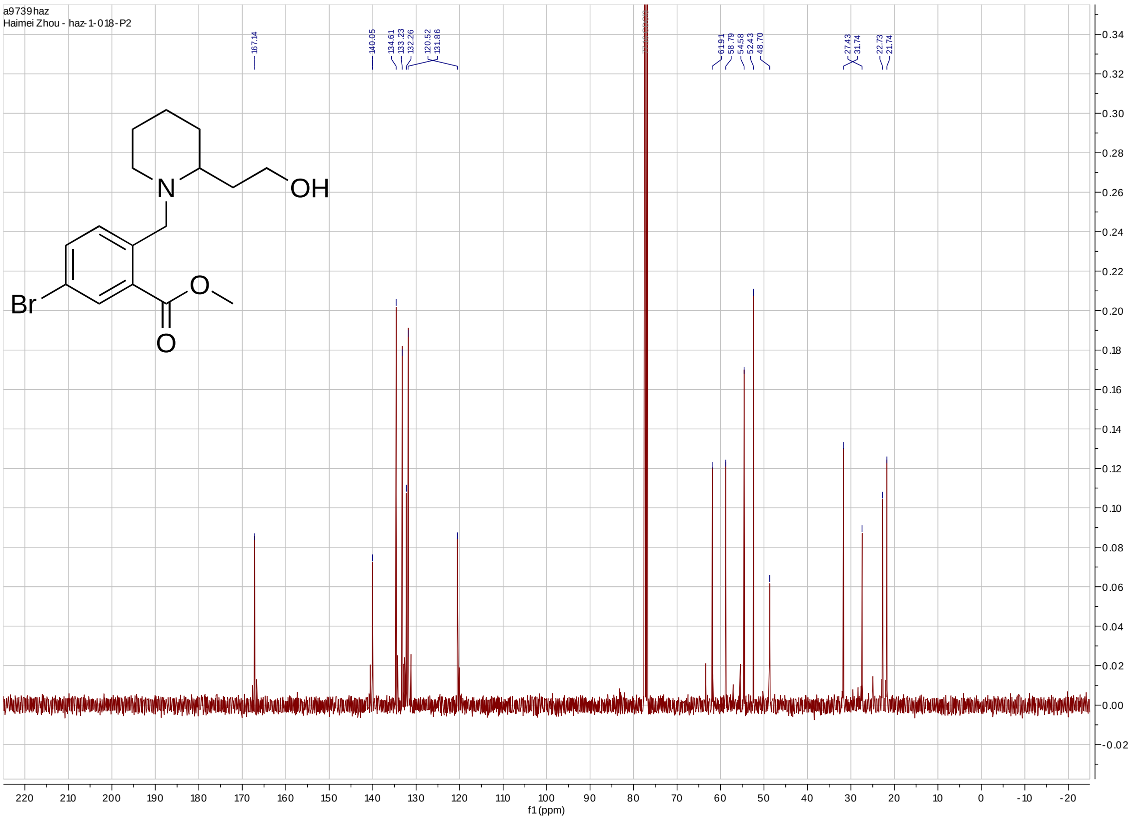

3-Bromo-8,8a,9,10,11,12-hexahydro-7H-benzo[g]pyrido[2,1-d][1,5]oxazonin-5(14H)-one (4f) - δ_H (400 MHz) and $^{13}C\{^1H\}$ NMR (101 MHz)

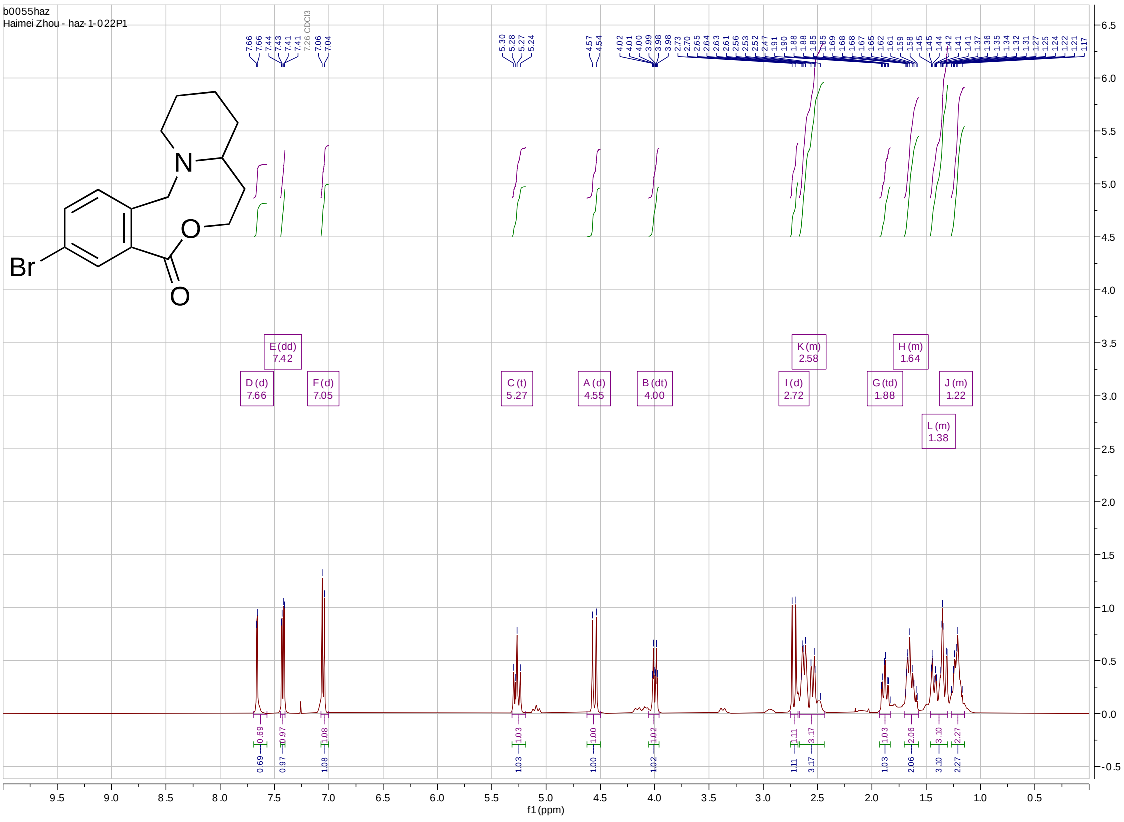

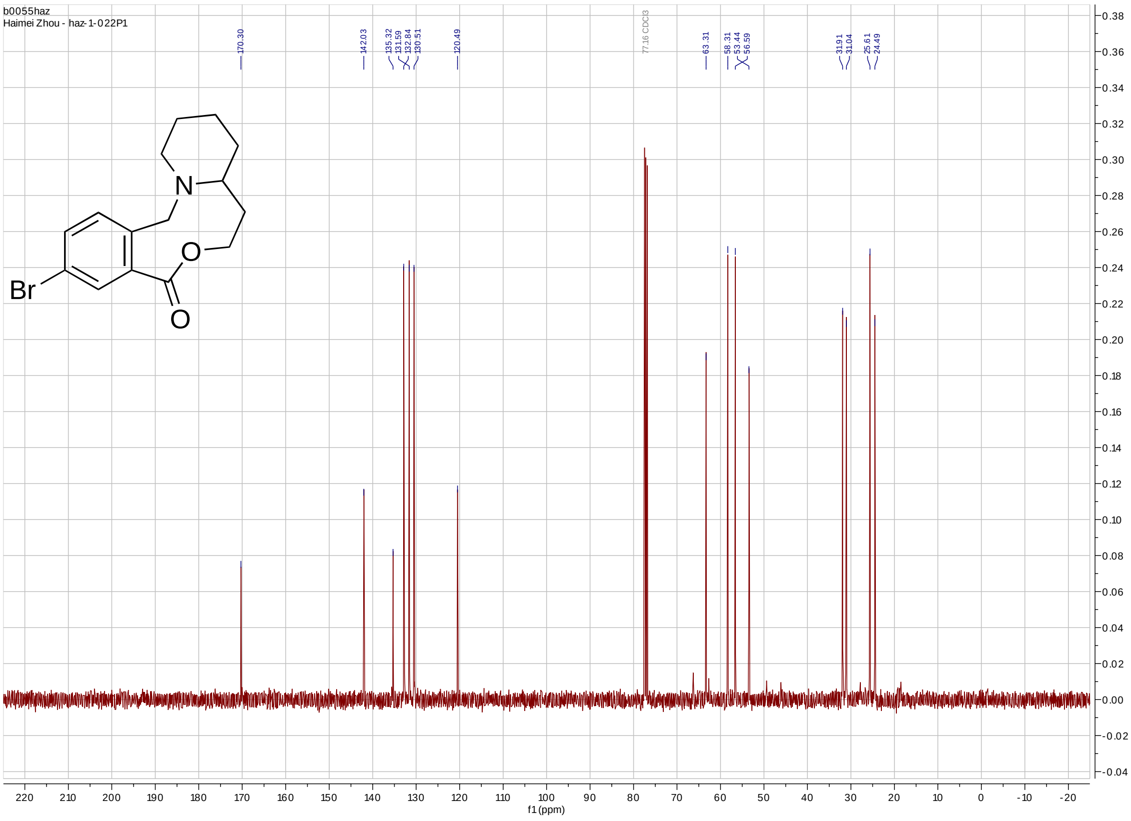

(5-Oxo-5,8,8a,9,10,11,12,14-octahydro-7H-benzo[g]pyrido[2,1-d][1,5]oxazonin-3-yl) boronic acid (4g) - δ_H (400 MHz) and $^{13}C\{^1H\}$ NMR (101 MHz)

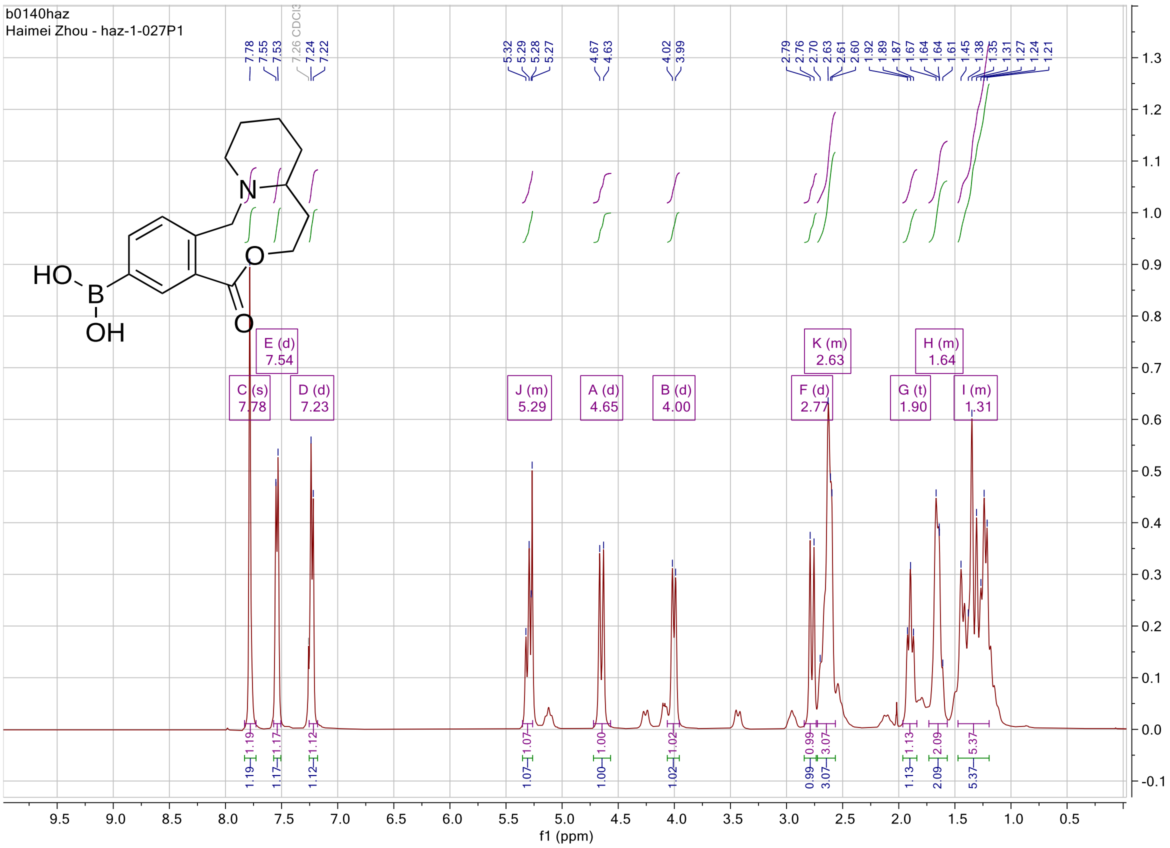

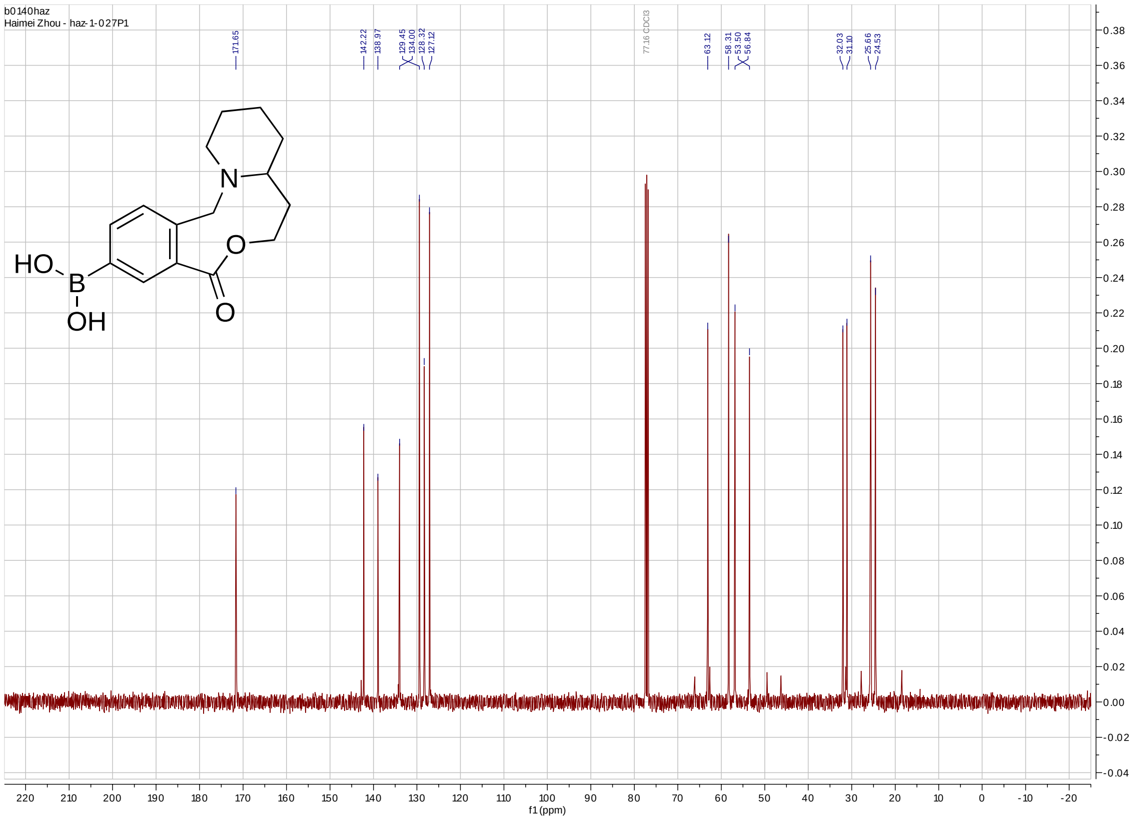

3-(4,4,5,5-Tetramethyl-1,3,2-dioxaborolan-2-yl)-8,8a,9,10,11,12-hexahydro-7H-benzo[g]pyrido[2,1-d][1,5]oxazonin-5(14H)-one (4h) - δ_H (400 MHz) and $^{13}C\{^1H\}$ NMR (101 MHz)

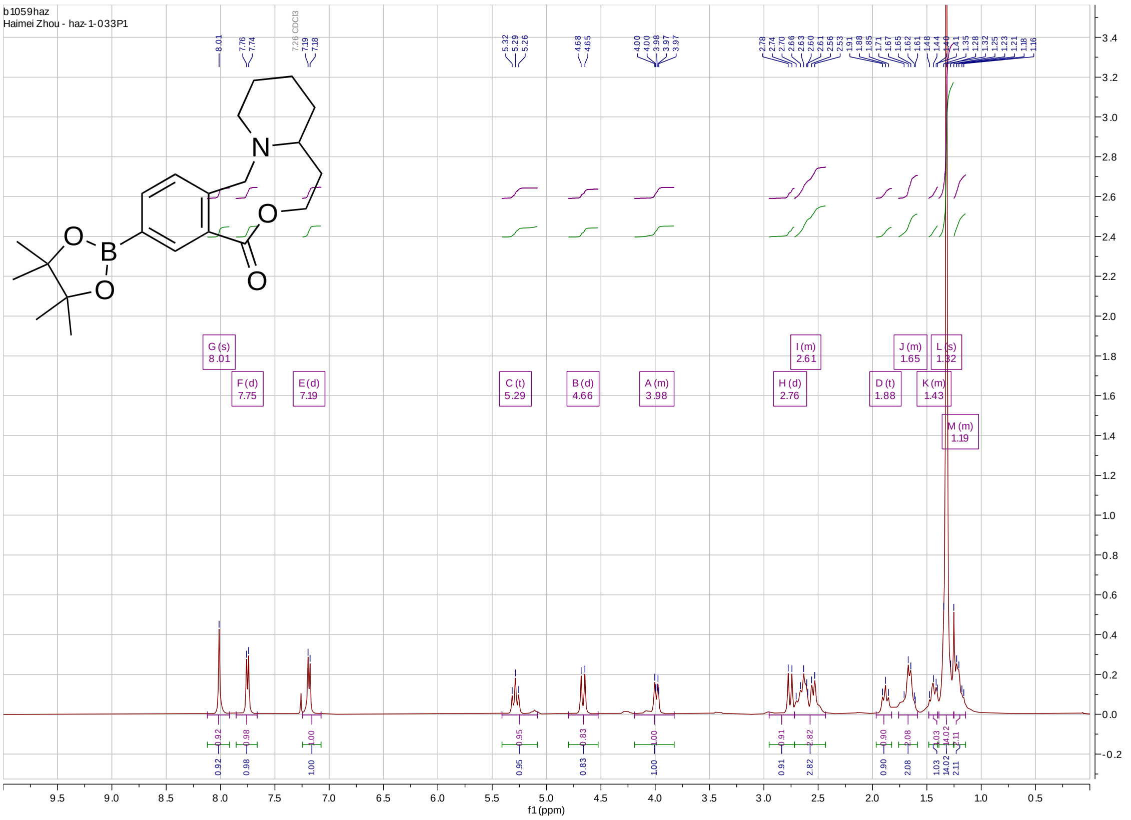

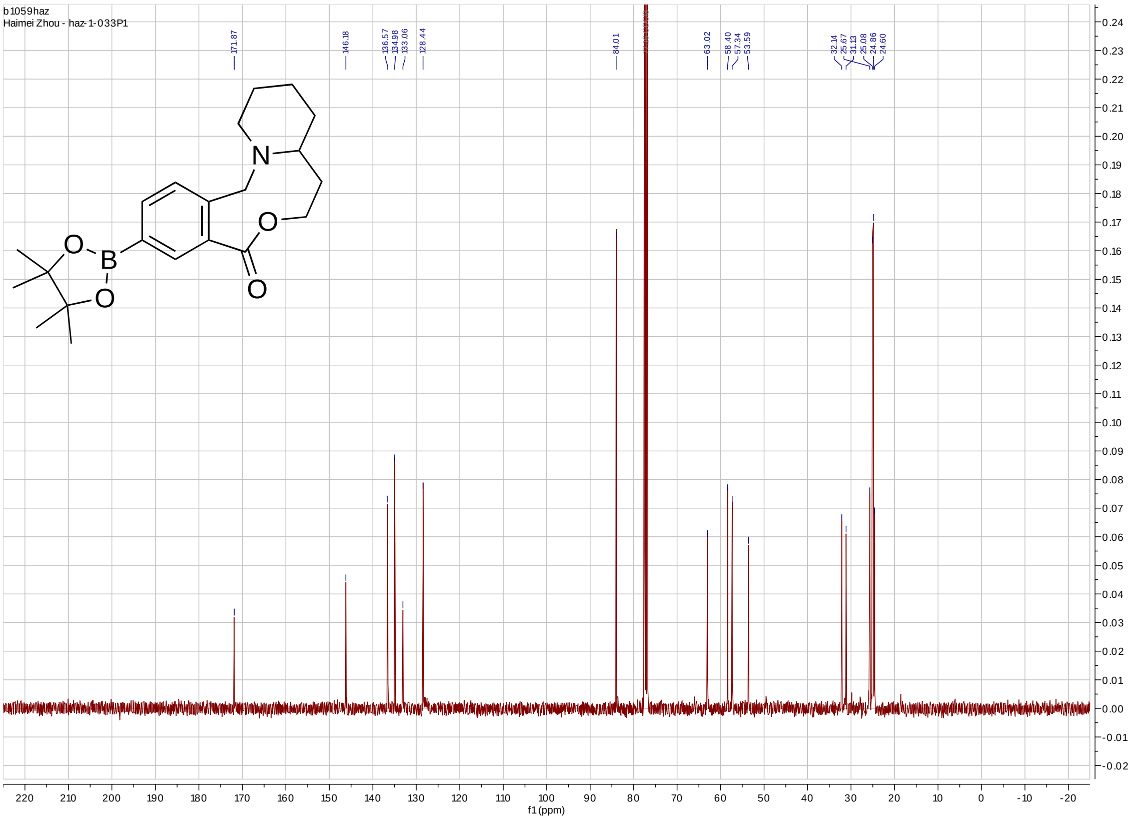

4-Phenyl-7,7a,8,9,10,11-hexahydrobenzo[f]pyrido[2,1-c][1,4]oxazocin-5(13H)-one (5a) - δ_H (400 MHz) and $^{13}C\{^1H\}$ NMR (101 MHz)

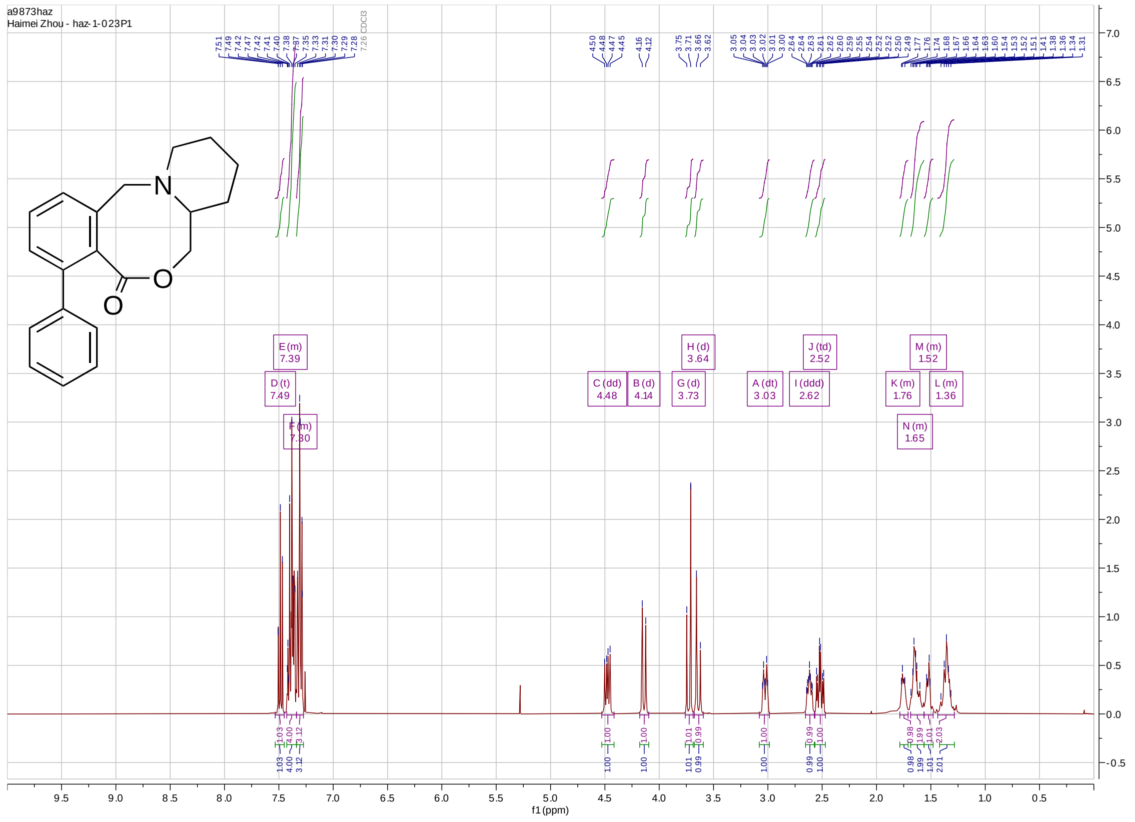

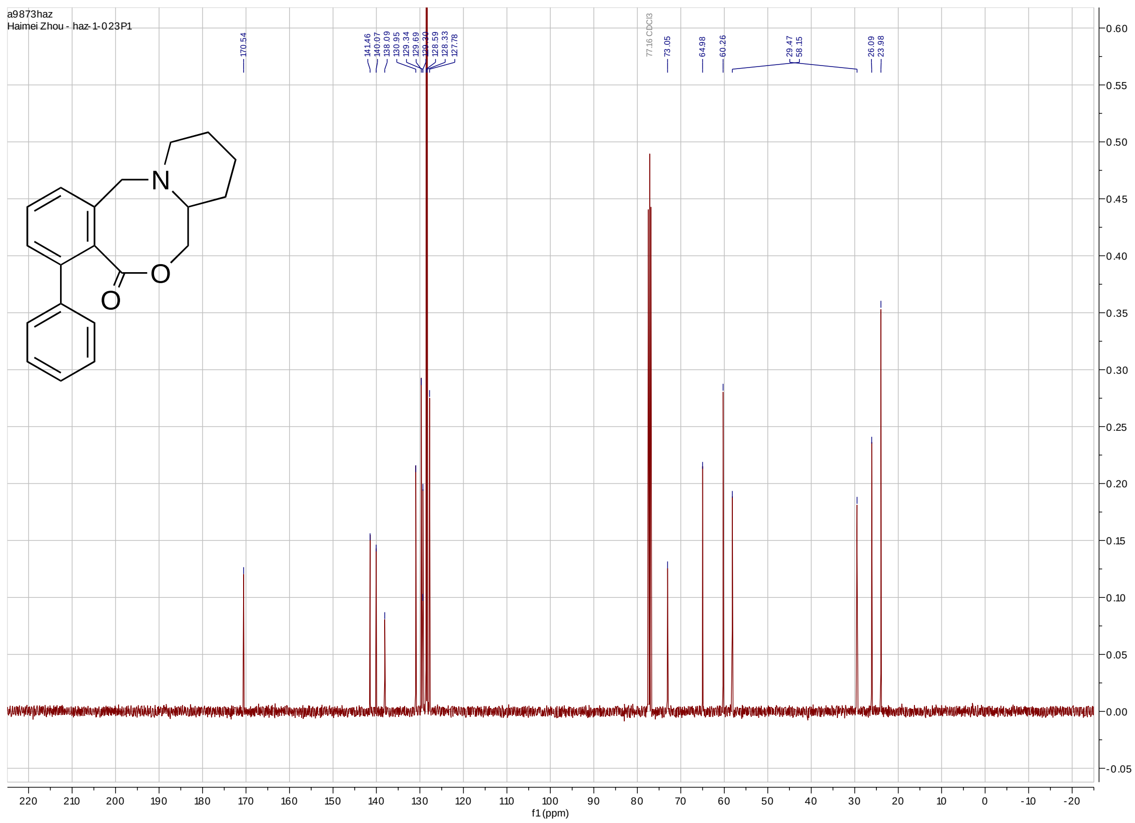

3-Phenyl-7,7a,8,9,10,11-hexahydrobenzo[f]pyrido[2,1-c][1,4]oxazocin-5(13H)-one (5b) - δ_H (400 MHz) and $^{13}C\{^1H\}$ NMR (101 MHz)

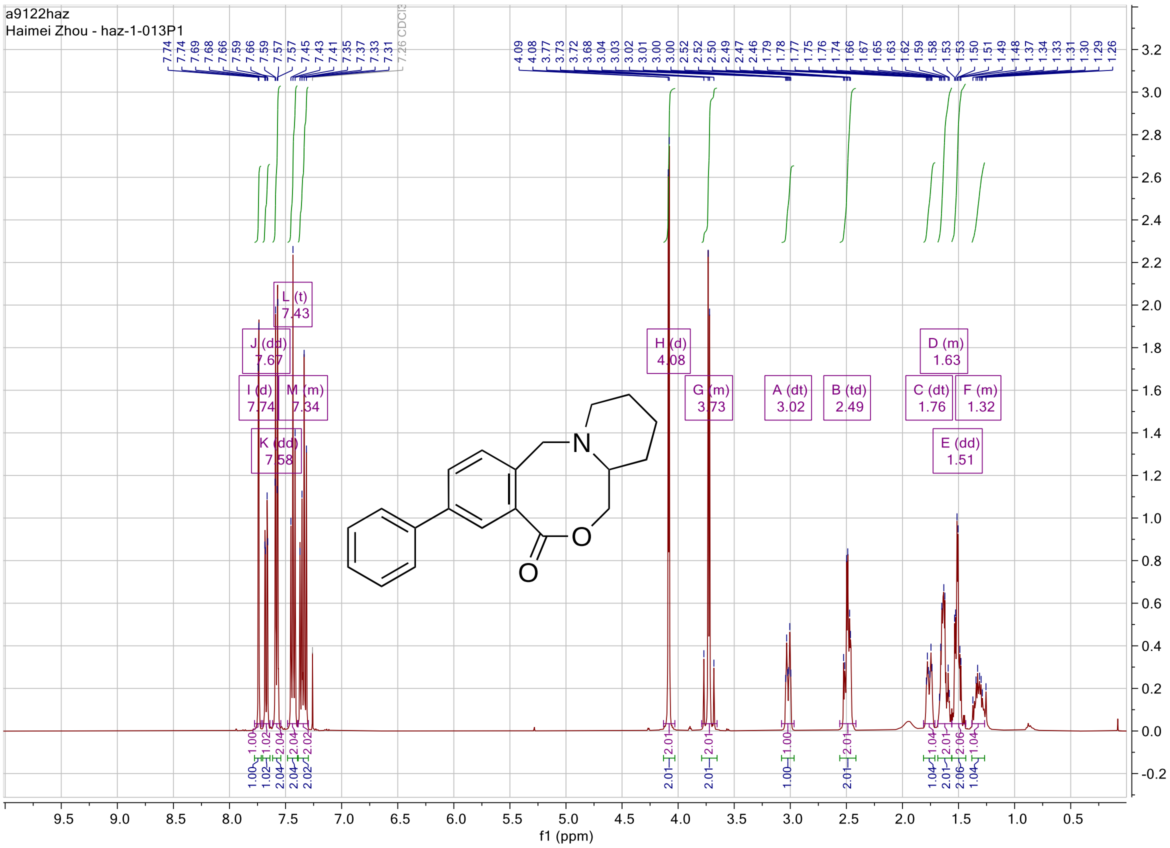

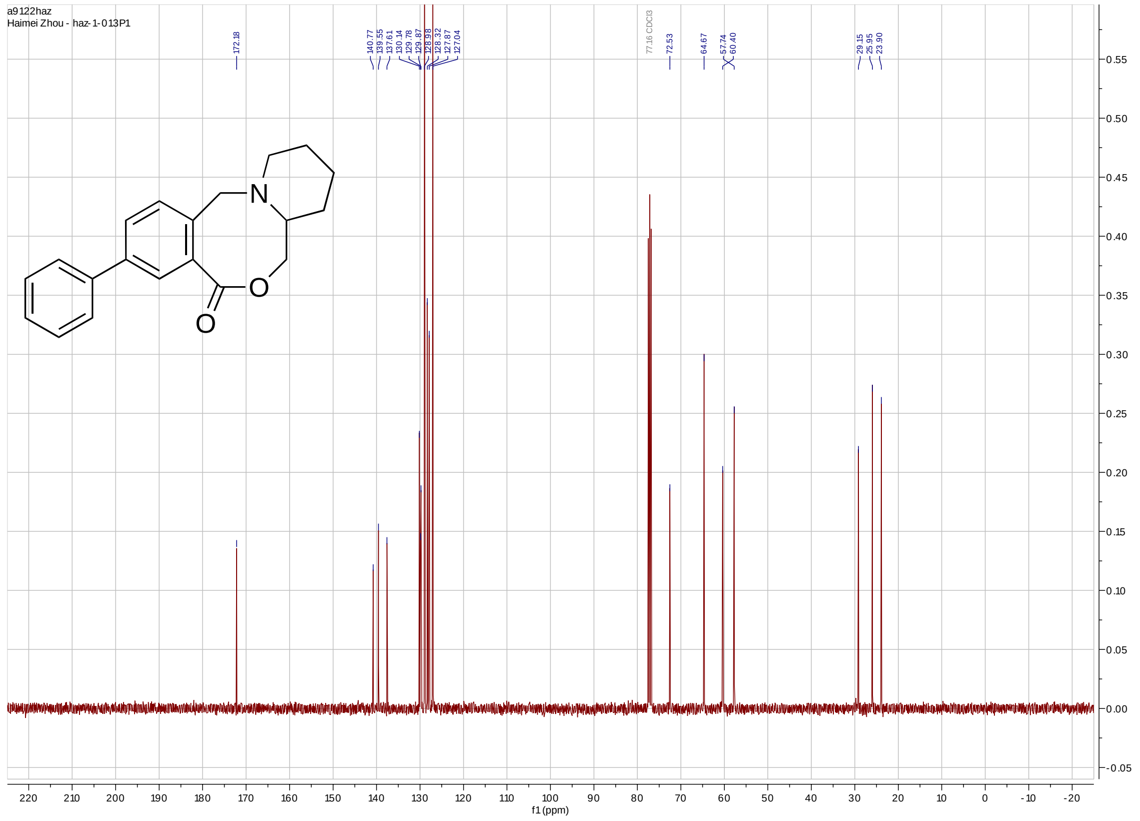

2-Phenyl-7,7a,8,9,10,11-hexahydrobenzo[f]pyrido[2,1-c][1,4]oxazocin-5(13H)-one (5c) - δ_H (400 MHz) and $^{13}C\{^1H\}$ NMR (101 MHz)

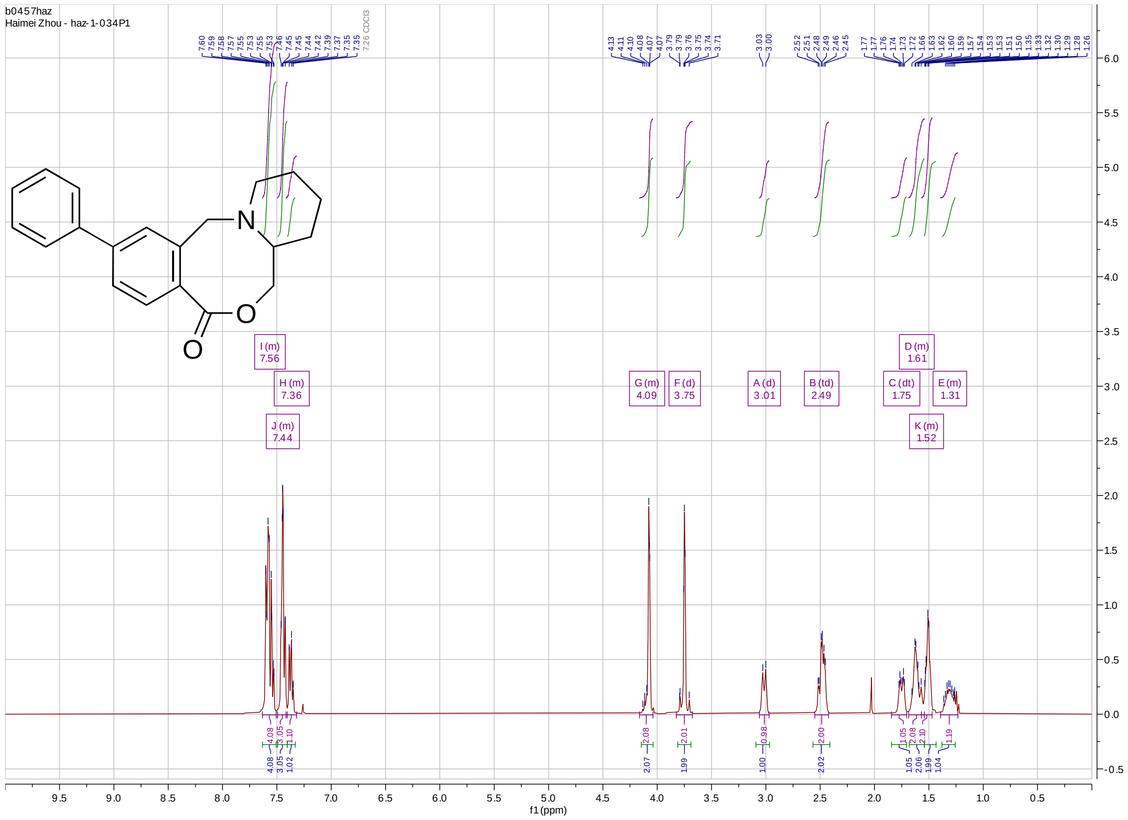

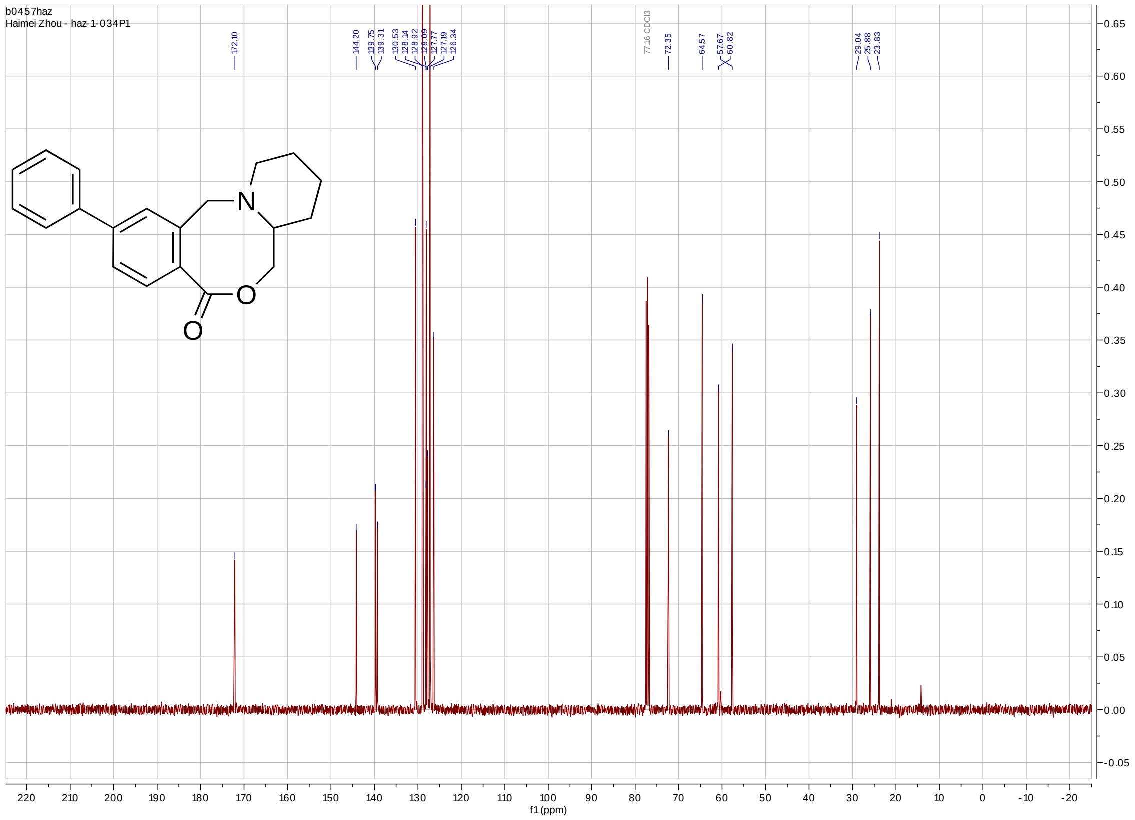

1-Phenyl-7,7a,8,9,10,11-hexahydrobenzo[f]pyrido[2,1-c][1,4]oxazocin-5(13H)-one (5d) - δ_H (400 MHz) and $^{13}C\{^1H\}$ NMR (101 MHz)

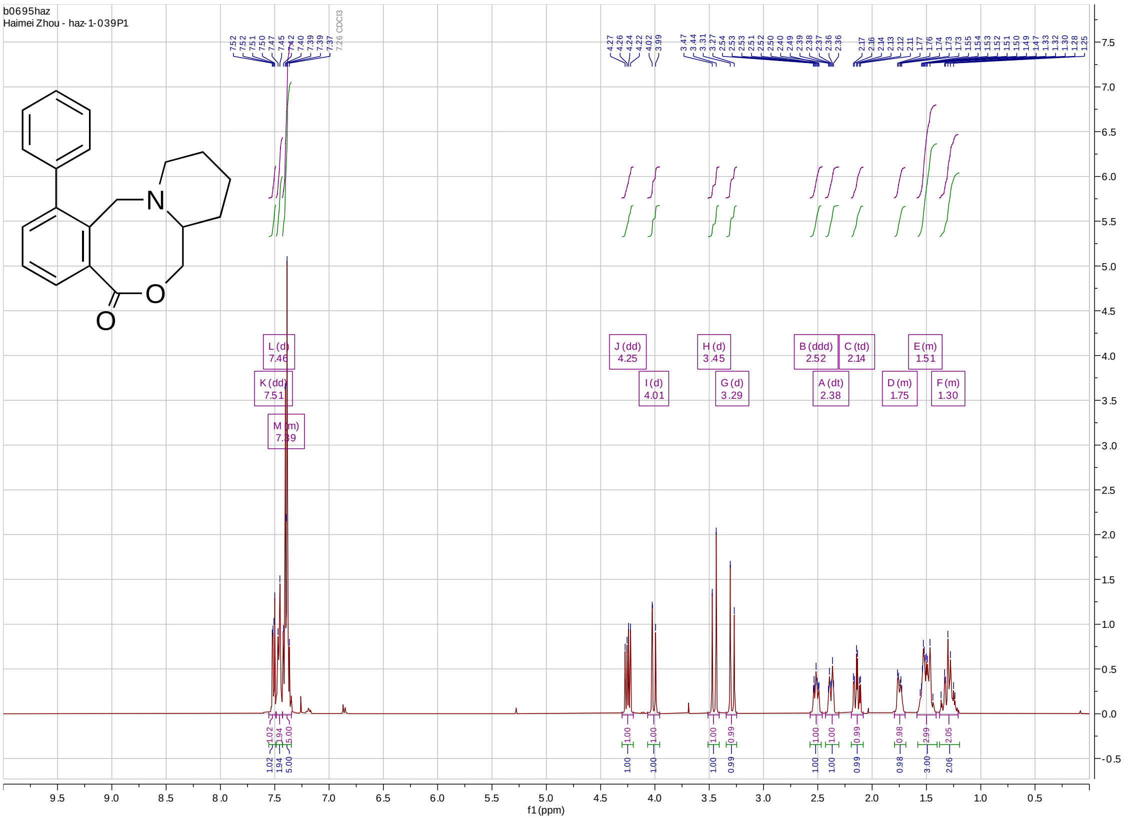

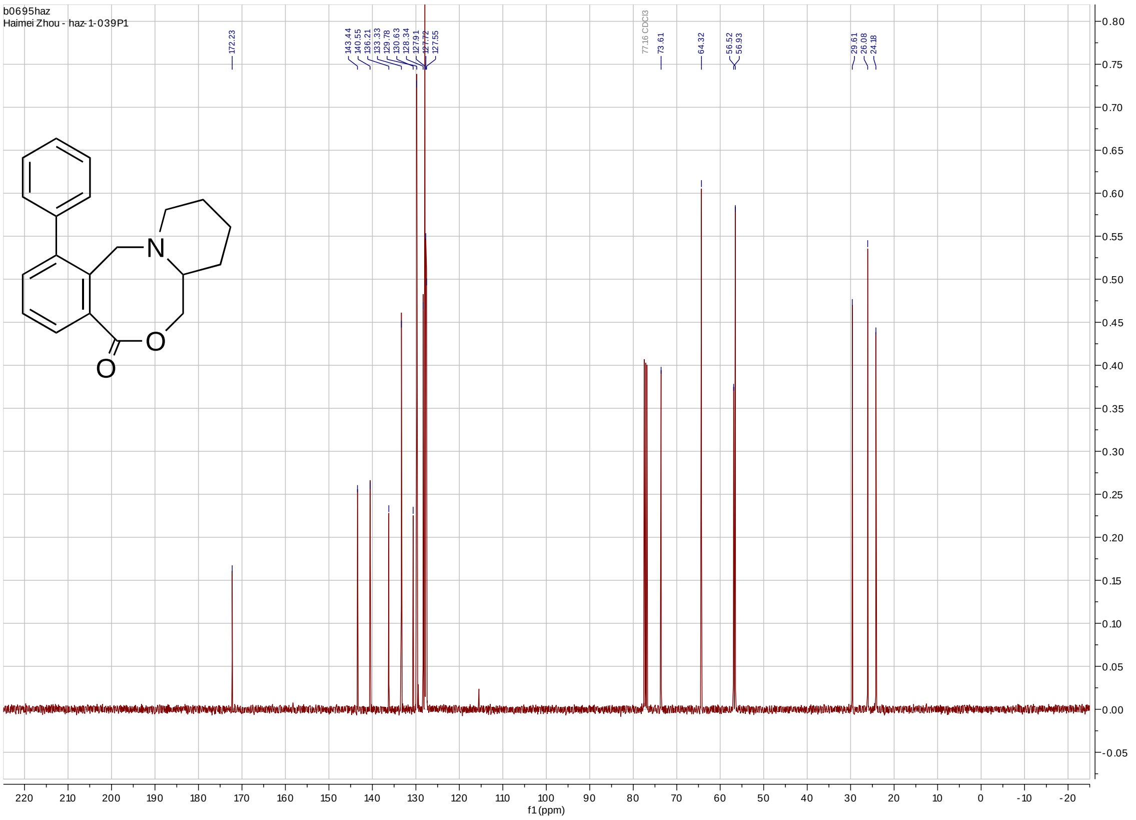

8-Phenyl-2,3,3a,4-tetrahydro-1H-benzo[f]pyrrolo[2,1-c][1,4]oxazocin-6(11H)-one (5e) - δ_H (400 MHz) and $^{13}C\{^1H\}$ NMR (101 MHz)

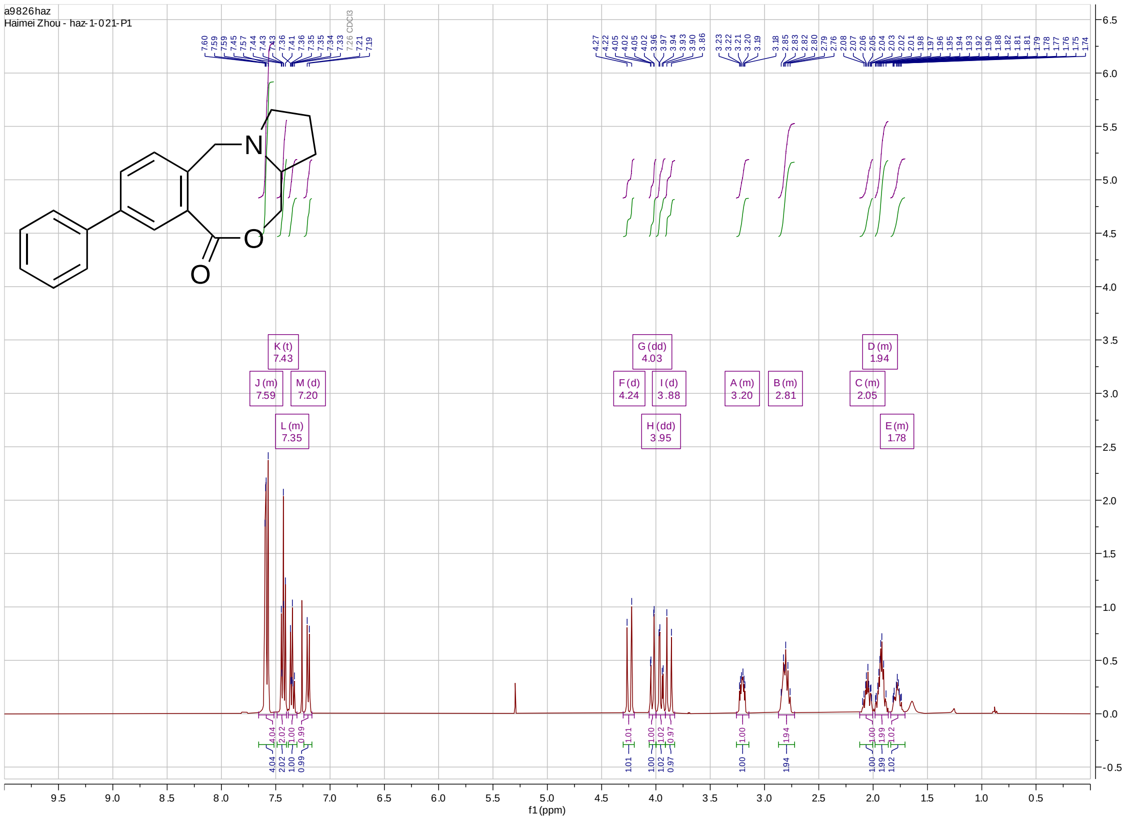

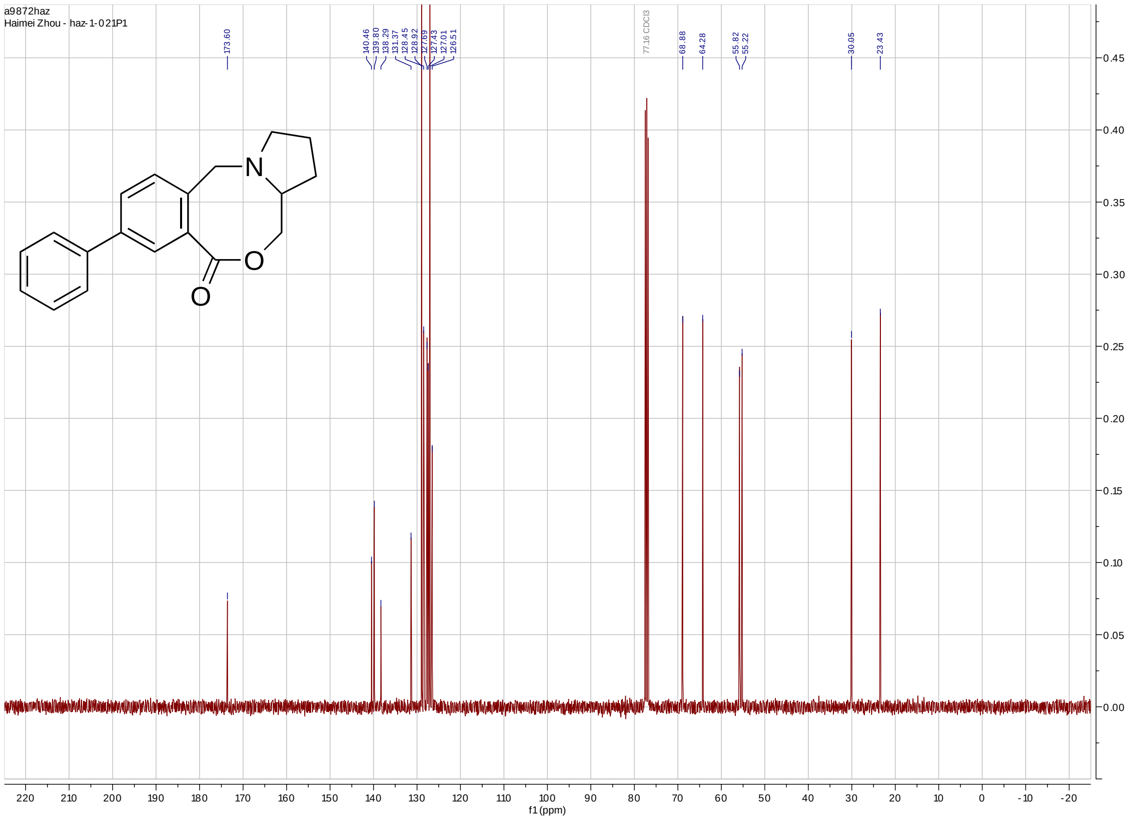

3-Phenyl-8,8a,9,10,11,12-hexahydro-7H-benzo[g]pyrido[2,1-d][1,5]oxazonin-5(14H)-one (5f) - δ_H (400 MHz) and $^{13}C\{^1H\}$ NMR (101 MHz)

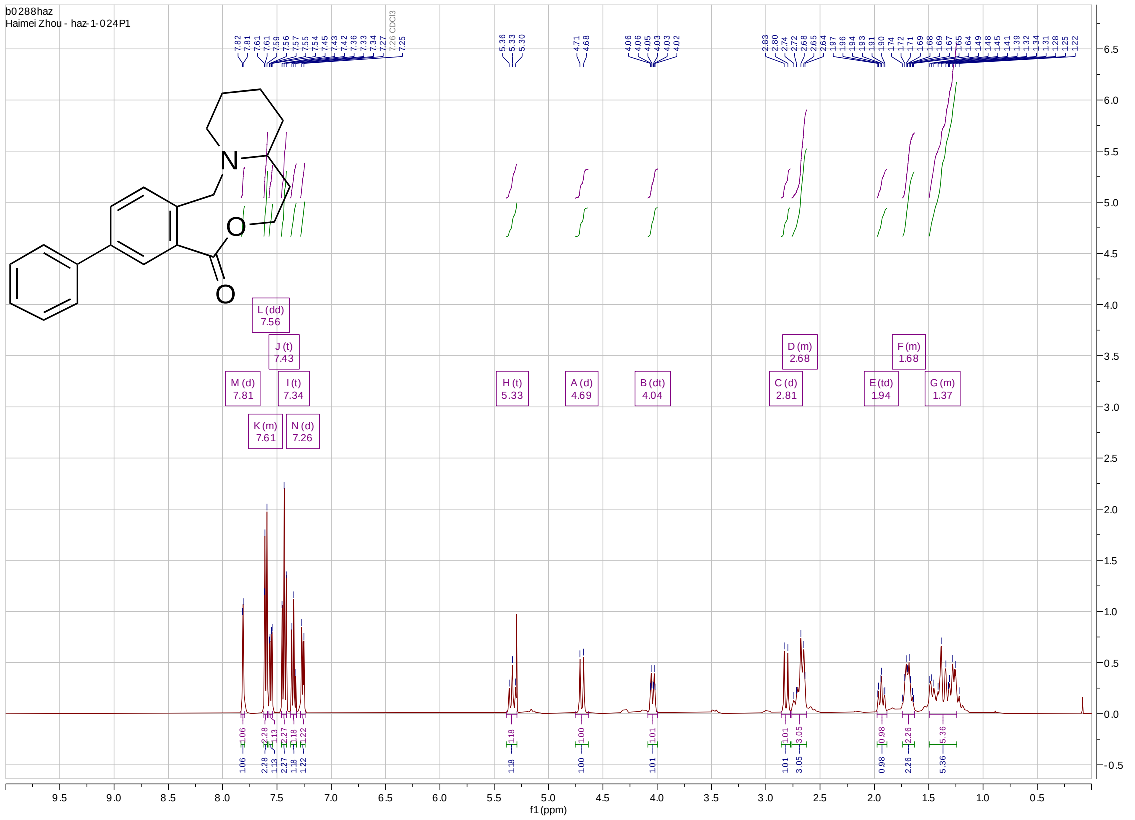

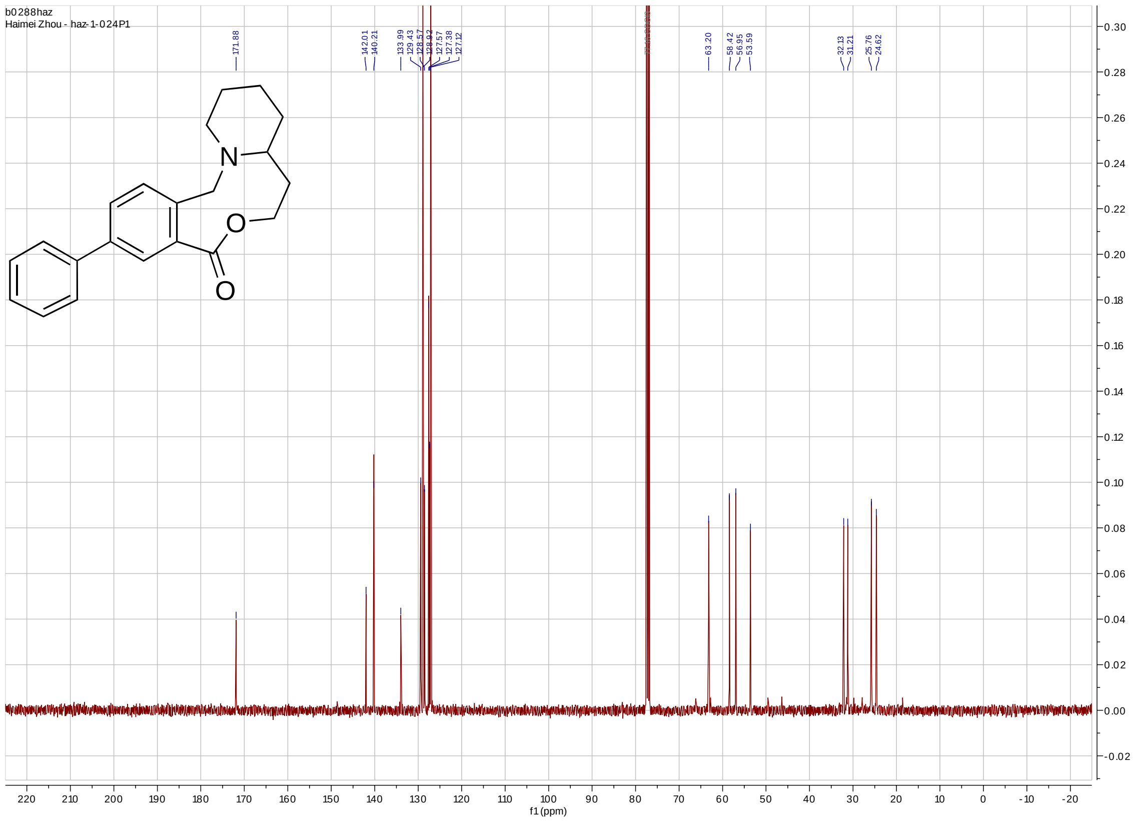

tert-Butyl 4-(5-bromo-2-(methoxycarbonyl)benzyl)-3-(hydroxymethyl)piperazine-1-carboxylate (8g) - δ$_H$ (400 MHz) and $^{13}C\{^1H\}$ NMR (101 MHz)

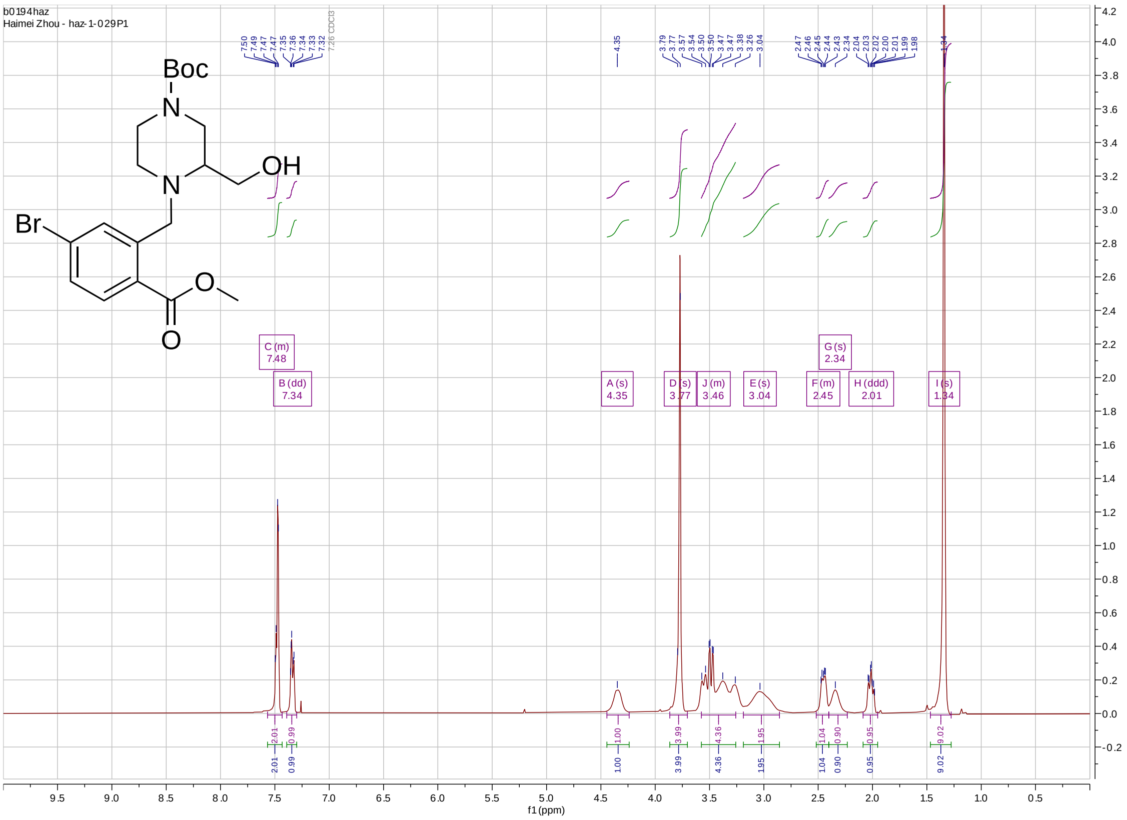

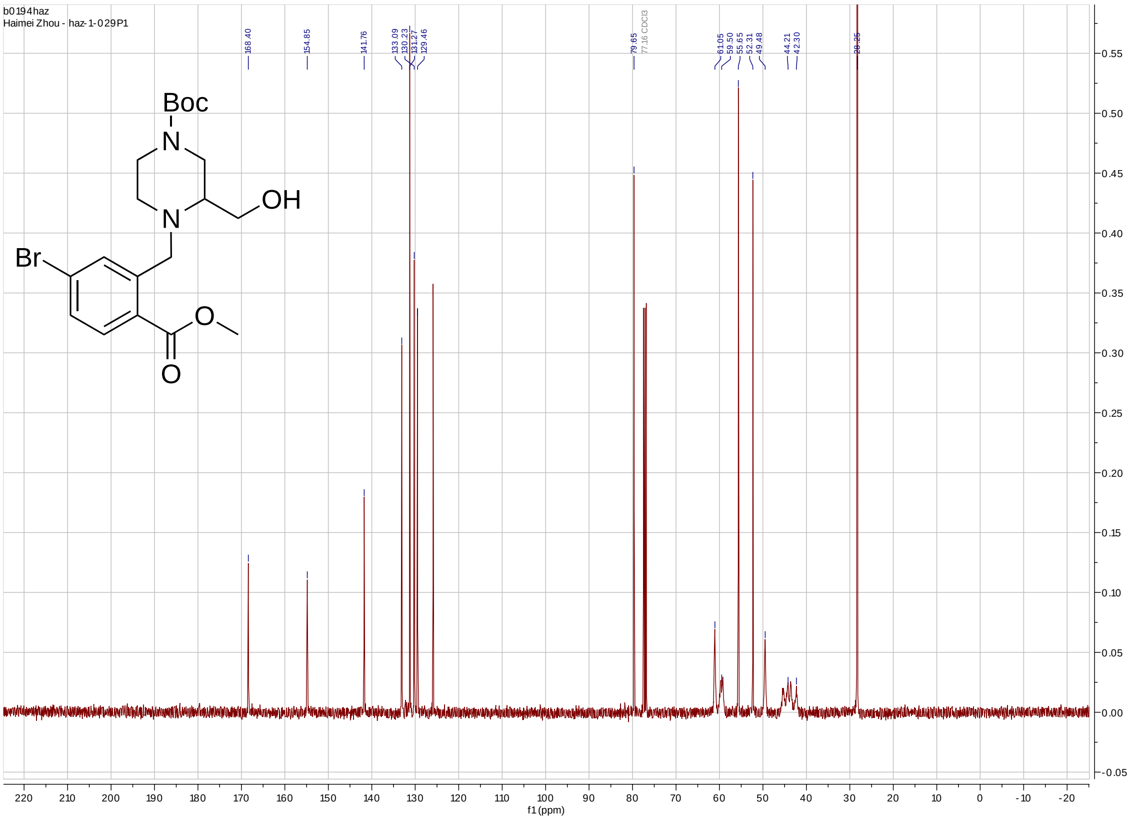

tert-Butyl 10-bromo-7-oxo-1,2,4a,5,7,12-hexahydrobenzo[f]pyrazino[2,1-c][1,4] oxazocine-3(4H)-carboxylate (4i) - δ_H (400 MHz) and $^{13}C\{^1H\}$ NMR (101 MHz)

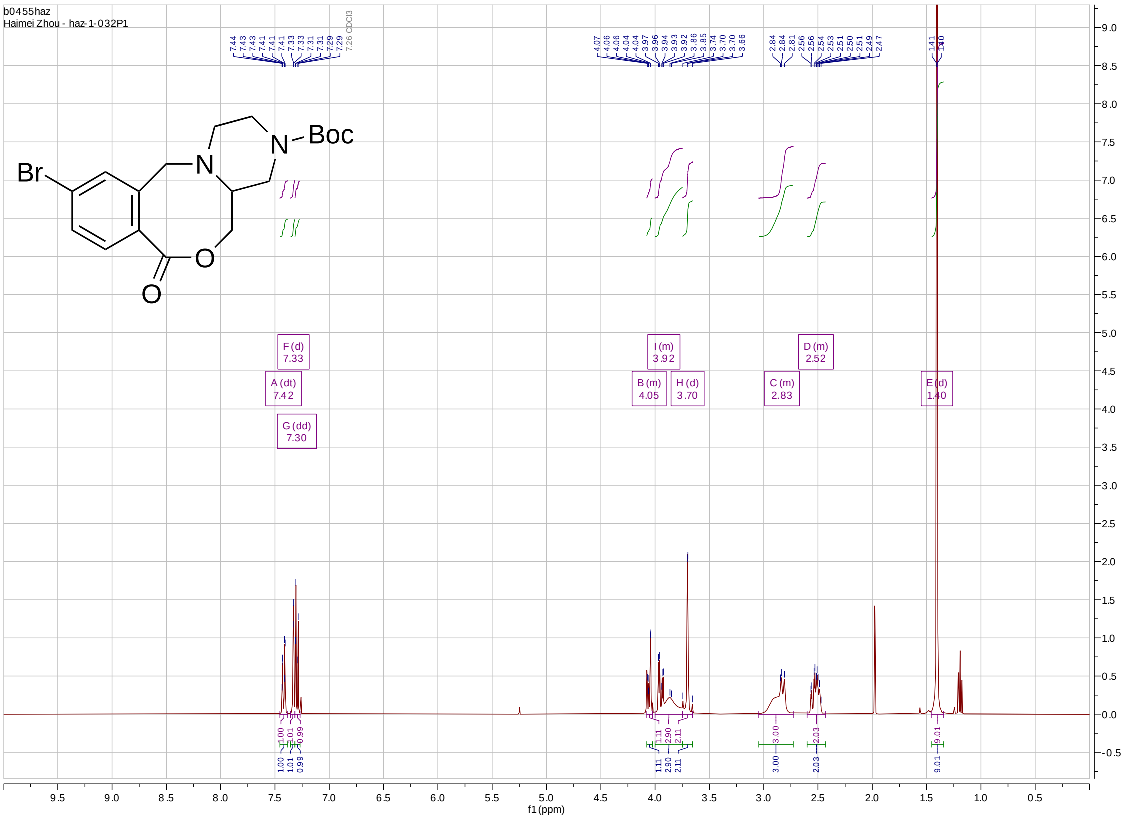

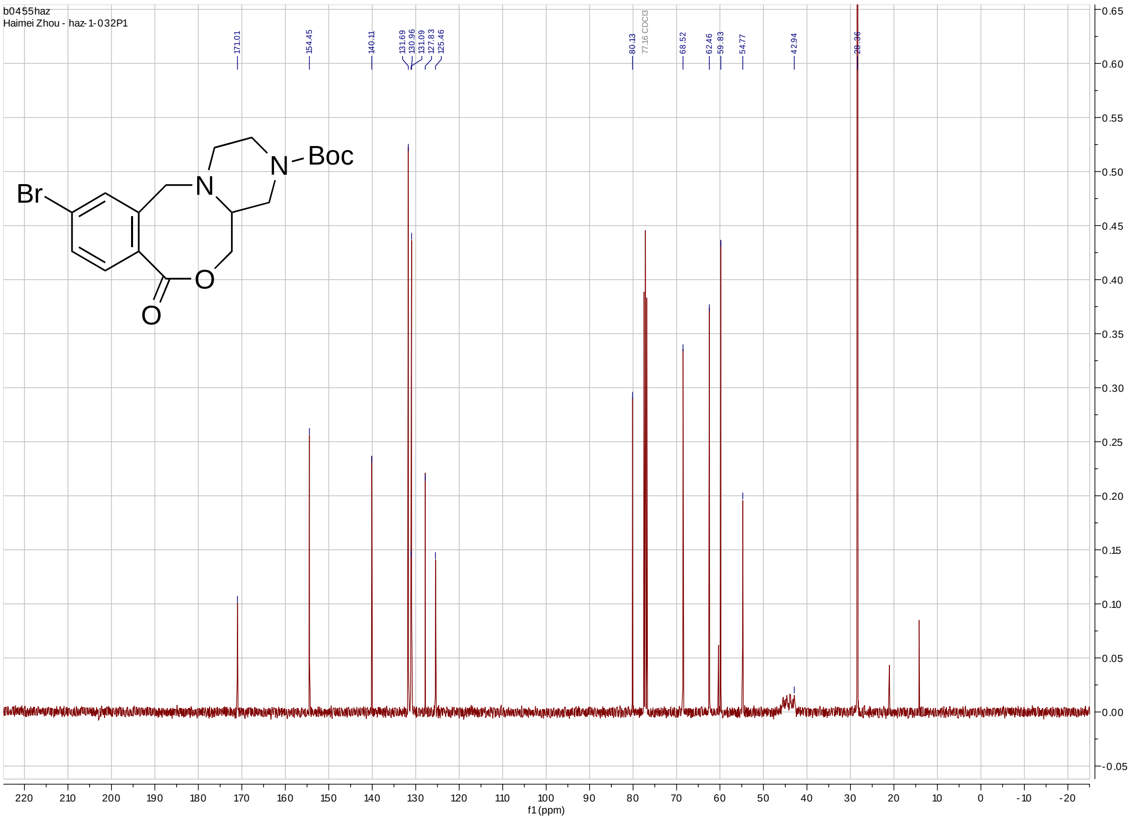

***tert*-Butyl 7-oxo-10-(4,4,5,5-tetramethyl-1,3,2-dioxaborolan-2-yl)-1,2,4a,5,7,12-hexahydrobenzo[f]pyrazino[2,1-c][1,4]oxazocine-3(4H)-carboxylate (4j)** - δ_H (400 MHz) and $^{13}C\{^1H\}$ NMR (101 MHz)

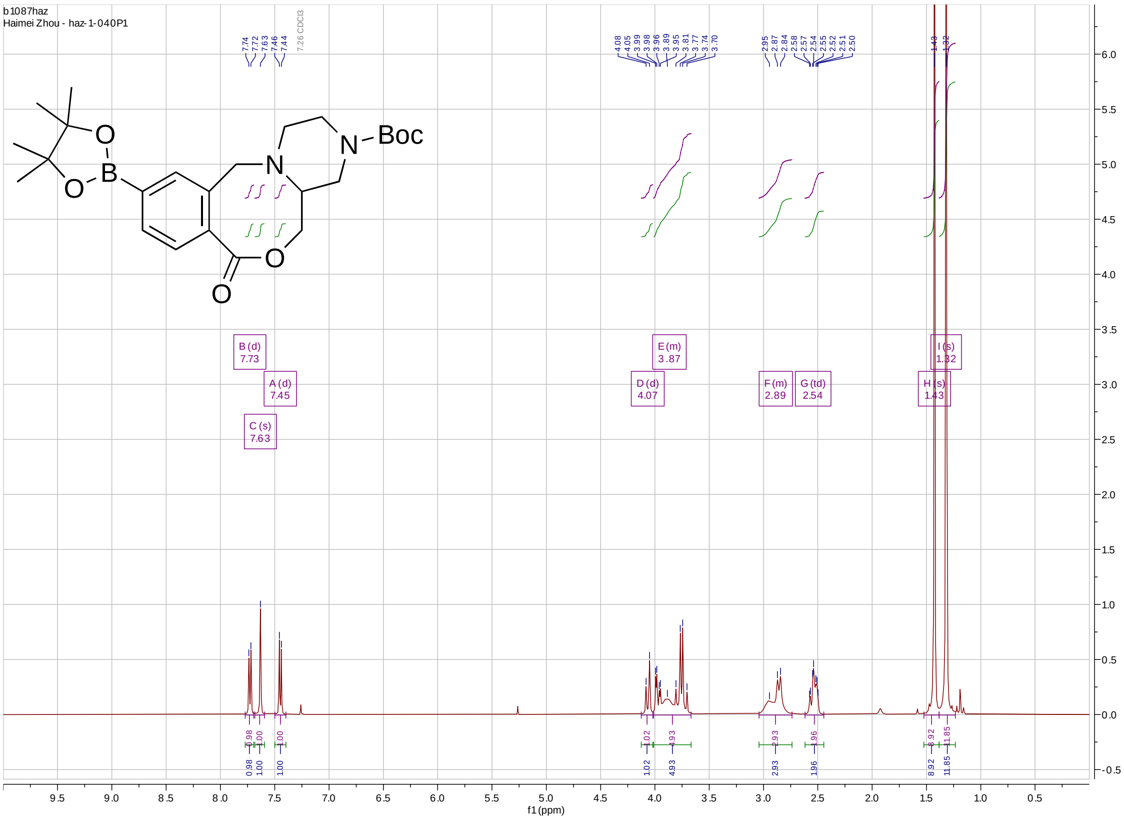

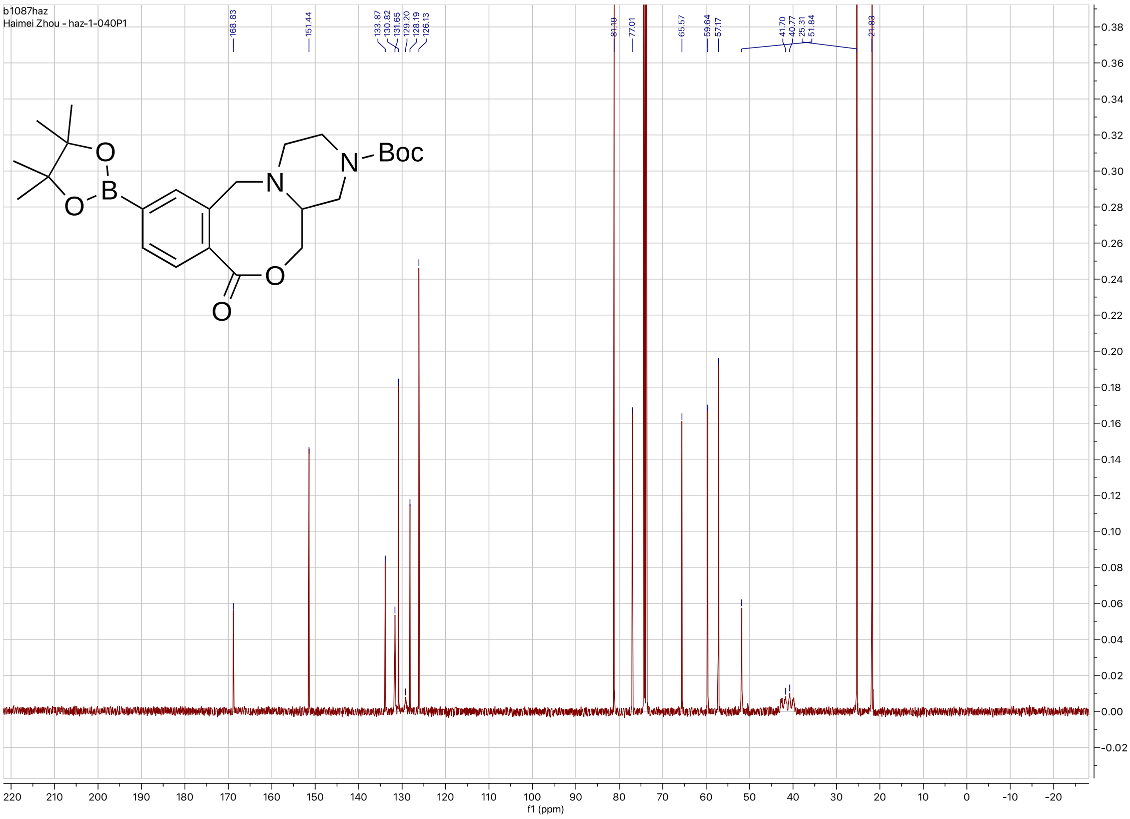

***tert*-Butyl 7-oxo-10-phenyl-1,2,4a,5,7,12-hexahydrobenzo[f]pyrazino[2,1-c][1,4]oxazocine-3(4H)-carboxylate (5g) -** δ_H (400 MHz) and $^{13}C\{^1H\}$ NMR (101 MHz)

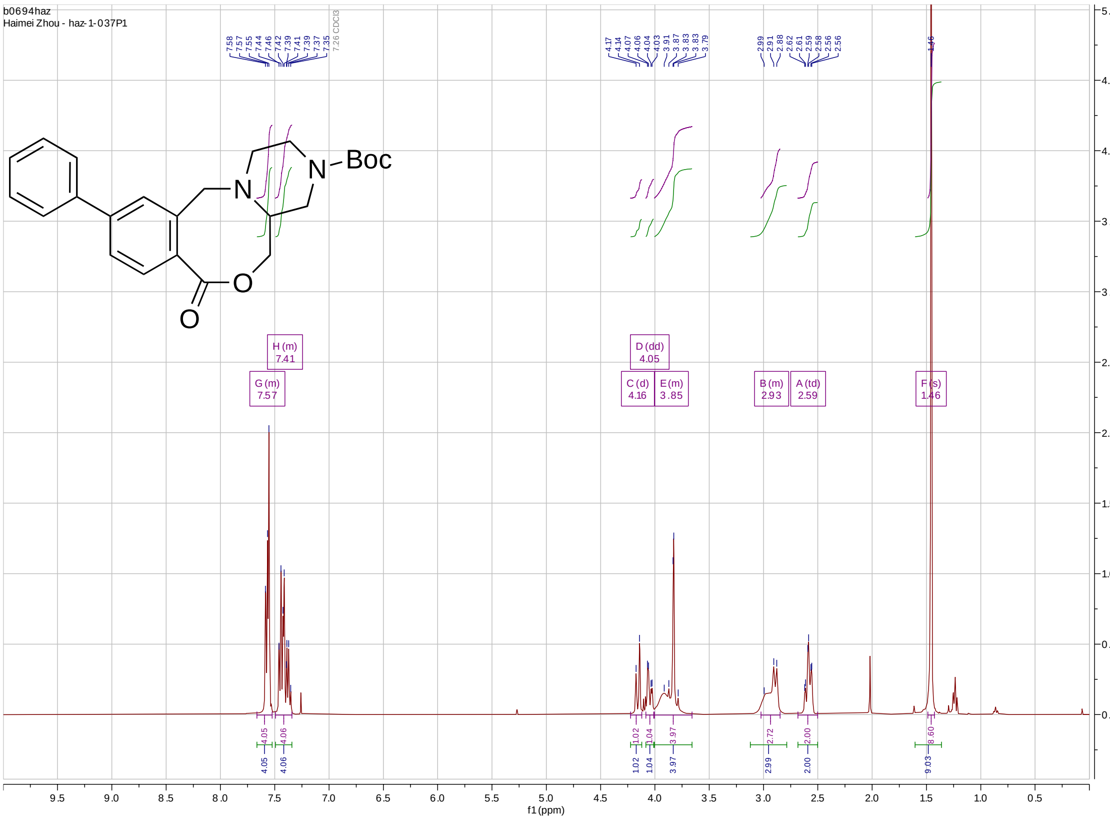

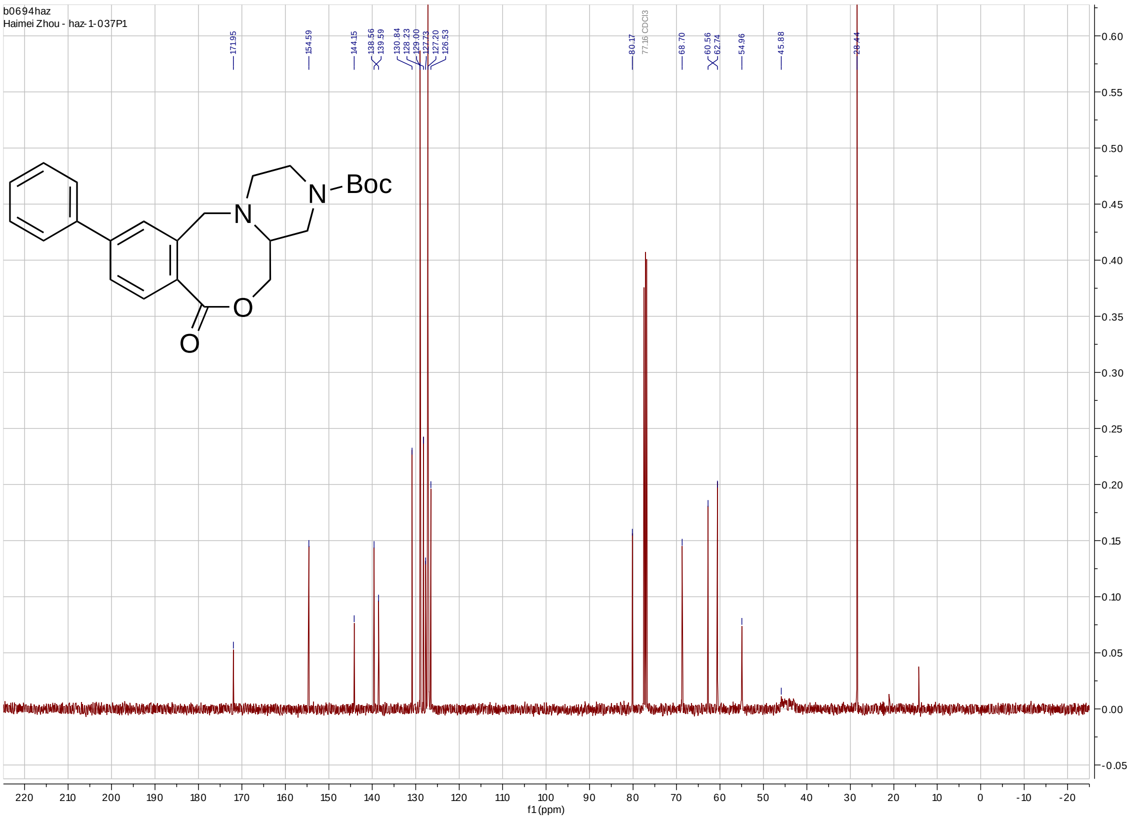

***tert*-Butyl 7-oxo-10-(pyridin-3-yl)-1,2,4a,5,7,12-hexahydrobenzo[f]pyrazino[2,1-c][1,4]oxazocine-3(4H)-carboxylate (5h) -** δ_H (400 MHz) and $^{13}C\{^1H\}$ NMR (101 MHz)

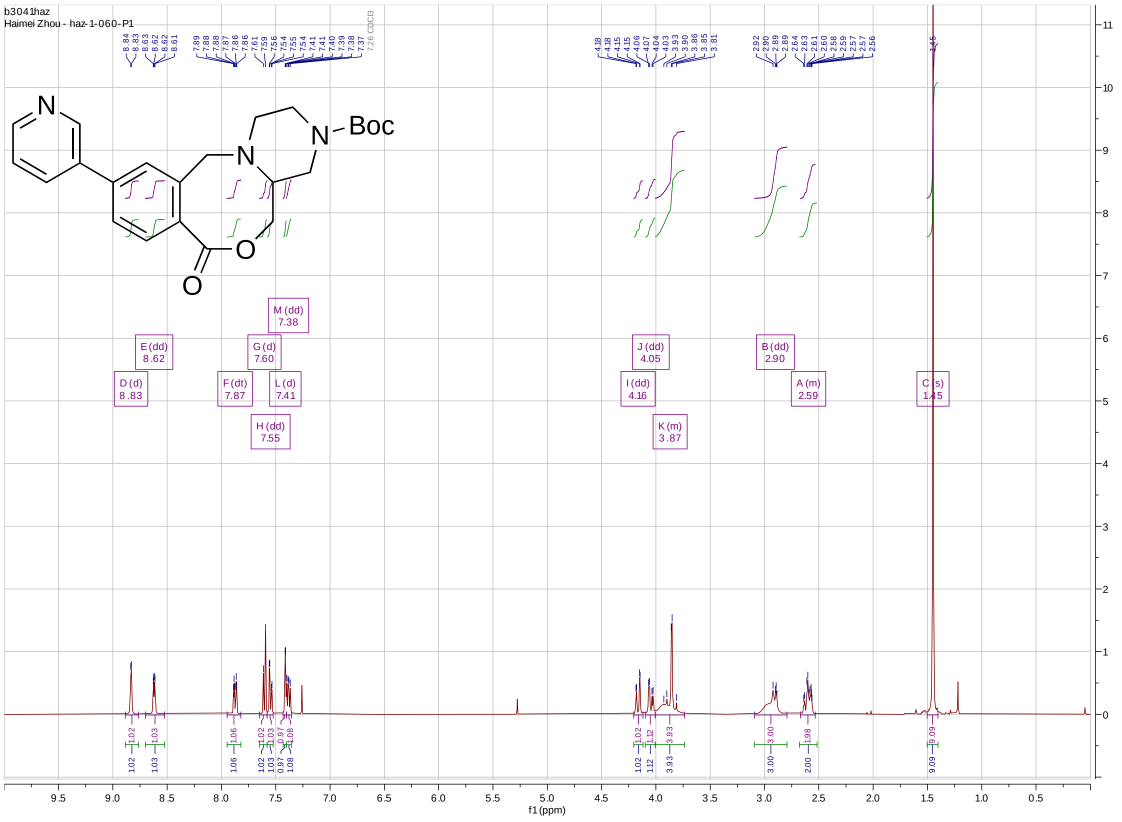

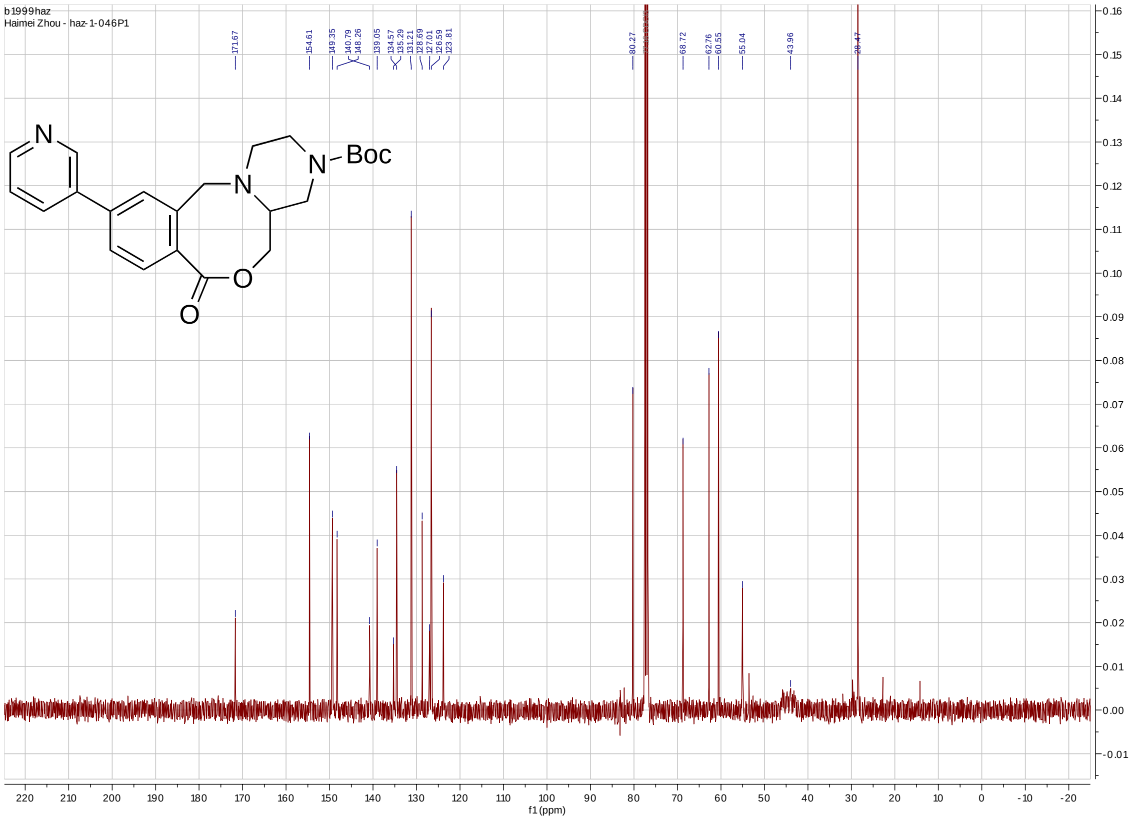

tert-Butyl 10-(1H-indol-5-yl)-7-oxo-1,2,4a,5,7,12-hexahydrobenzo[f]pyrazino[2,1-c][1,4]oxazocine-3(4H)-carboxylate (5i) - δ_H (400 MHz) and $^{13}C\{^1H\}$ NMR (101 MHz)

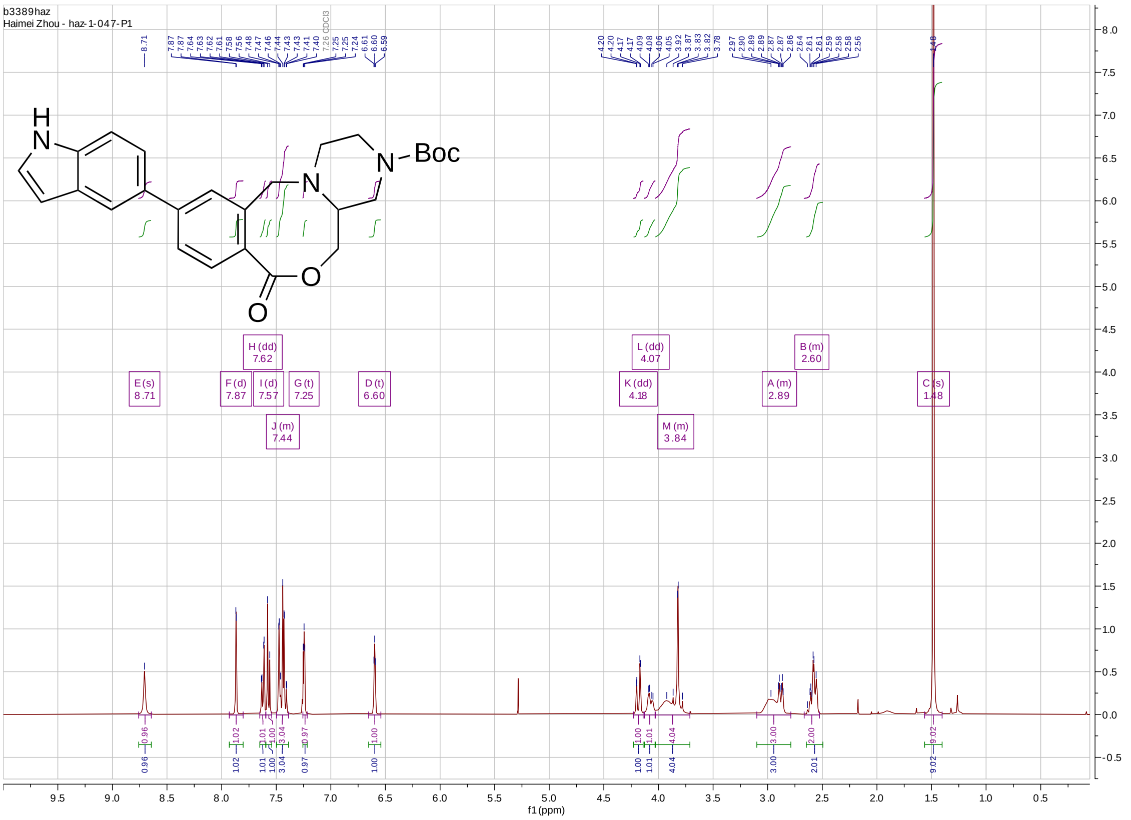

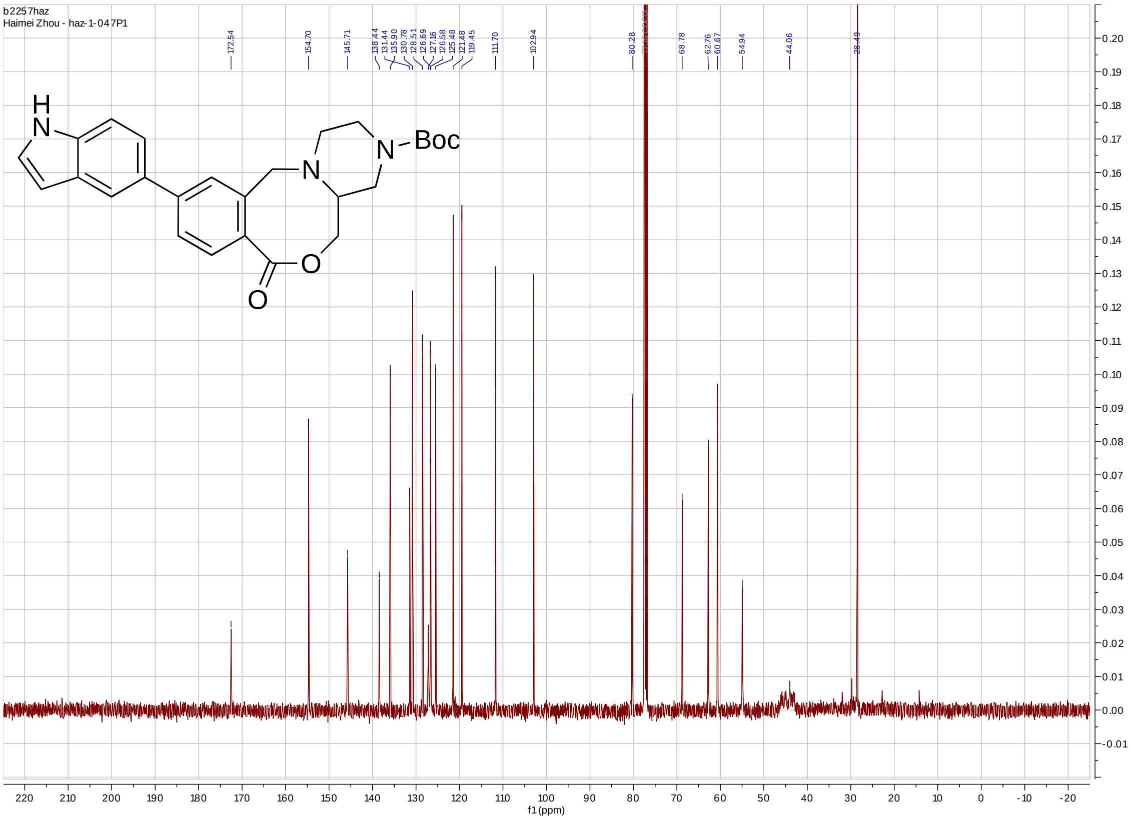

***tert*-Butyl 10-(1-methyl-1H-pyrazol-5-yl)-7-oxo-1,2,4a,5,7,12-hexahydrobenzo[f]pyrazino[2,1-c][1,4]oxazocine-3(4H)-carboxylate (5j) -** δ_H (400 MHz) and $^{13}C\{^1H\}$ NMR (101 MHz)

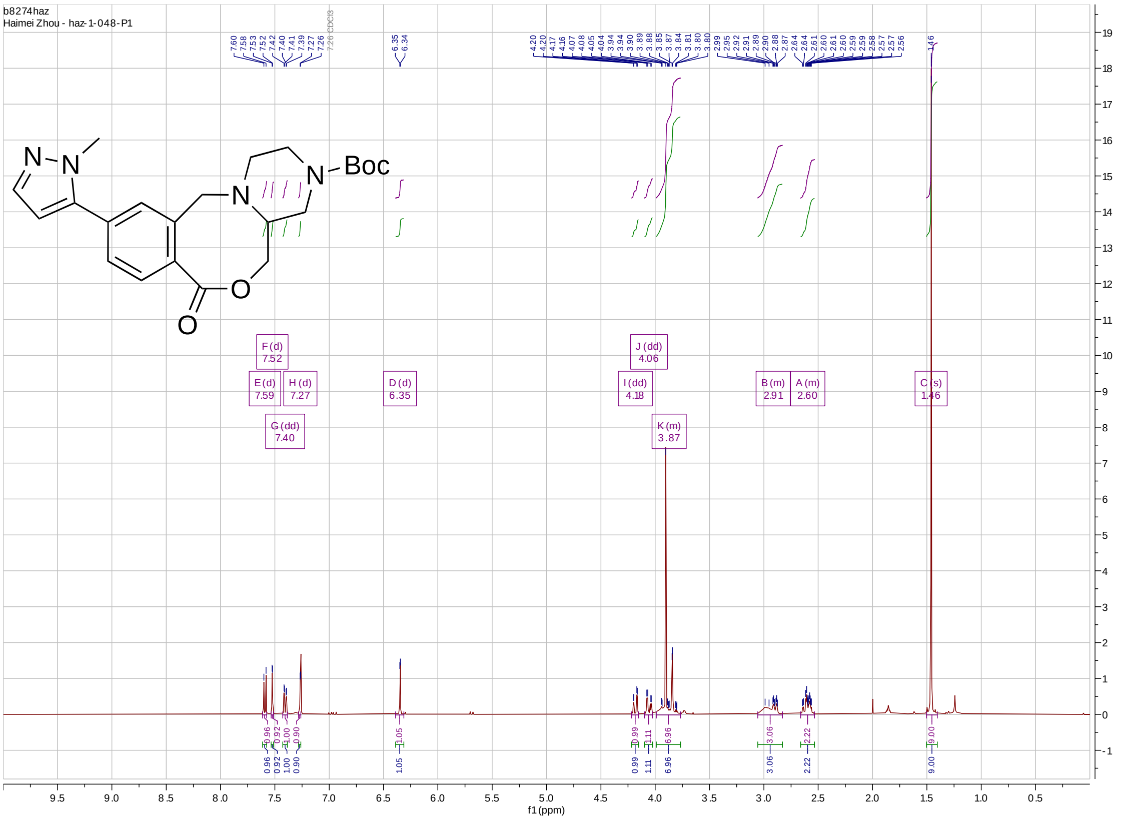

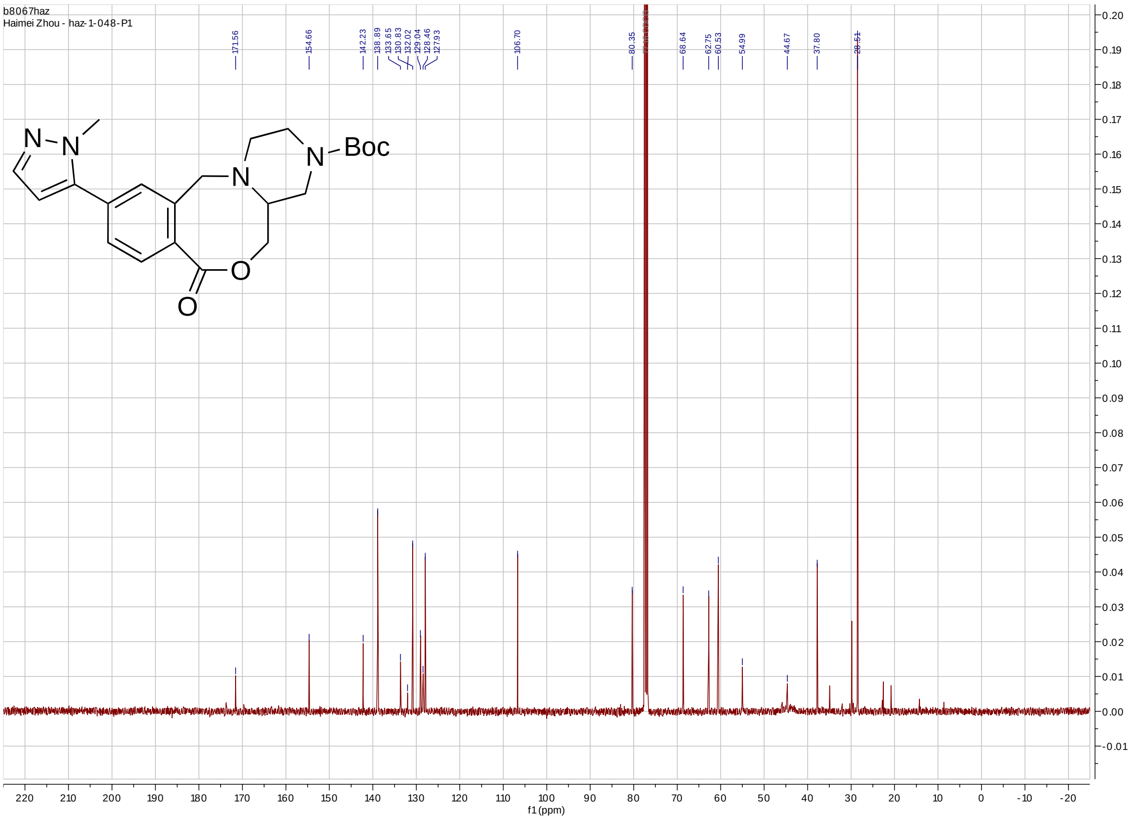

***tert*-Butyl 10-(4-fluoro-2-methoxyphenyl)-7-oxo-1,2,4a,5,7,12-hexahydrobenzo[f] pyrazino[2,1-c][1,4]oxazocine-3(4H)-carboxylate (5k) -** δ_H (400 MHz) and $^{13}C\{^1H\}$ NMR (101 MHz)

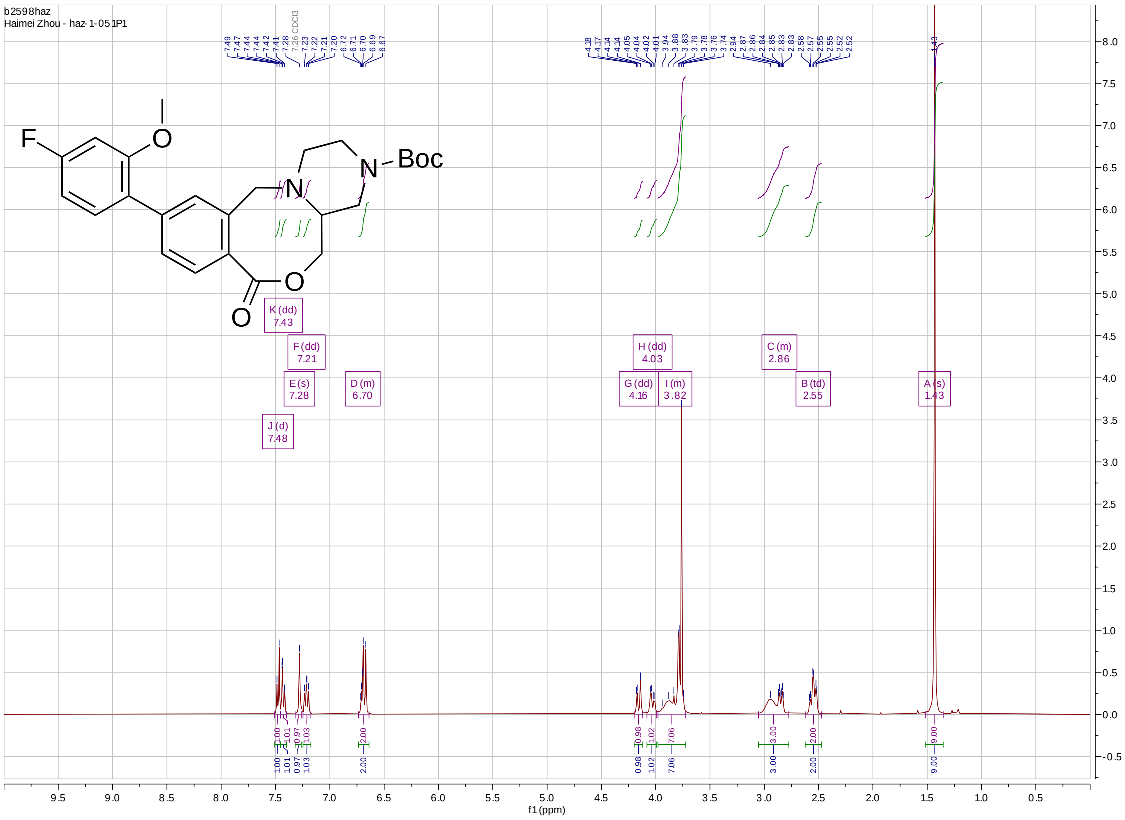

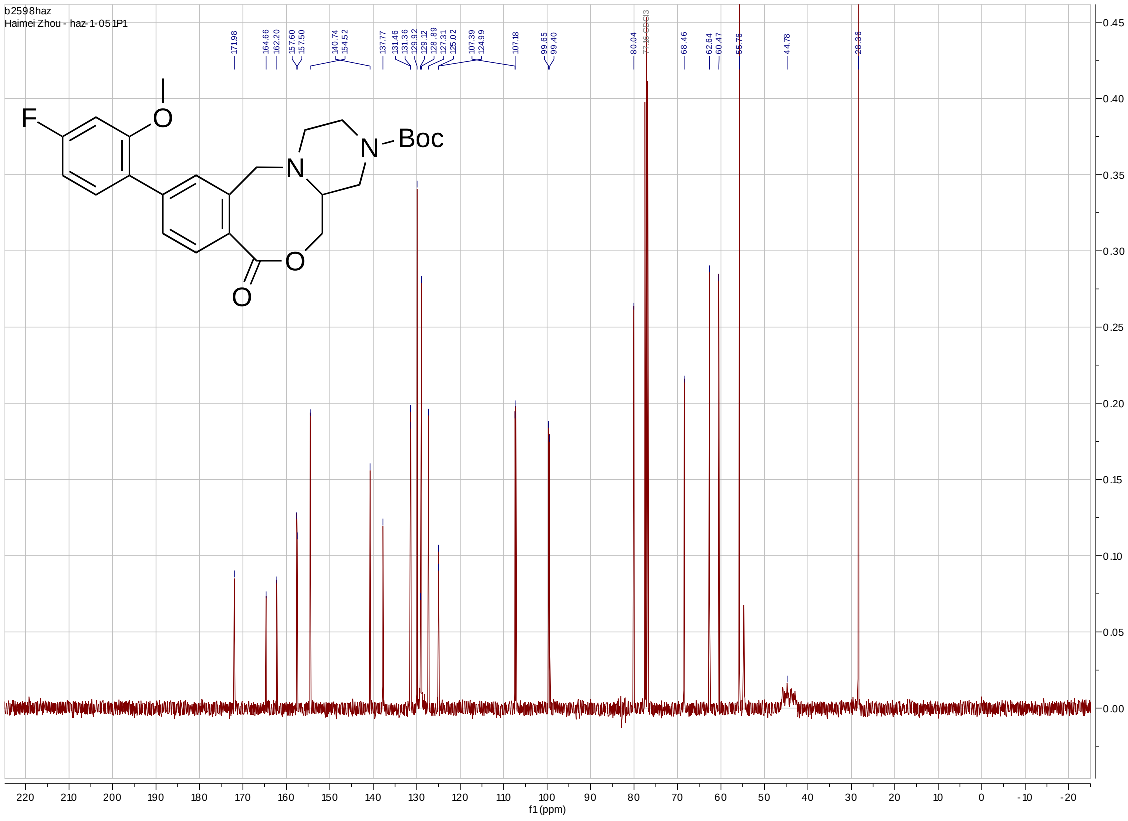

6-(4,4,5,5-Tetramethyl-1,3,2-dioxaborolan-2-yl)benzo[d]thiazole (S1) - δ_H (400 MHz) and $^{13}C\{^1H\}$ NMR (101 MHz)

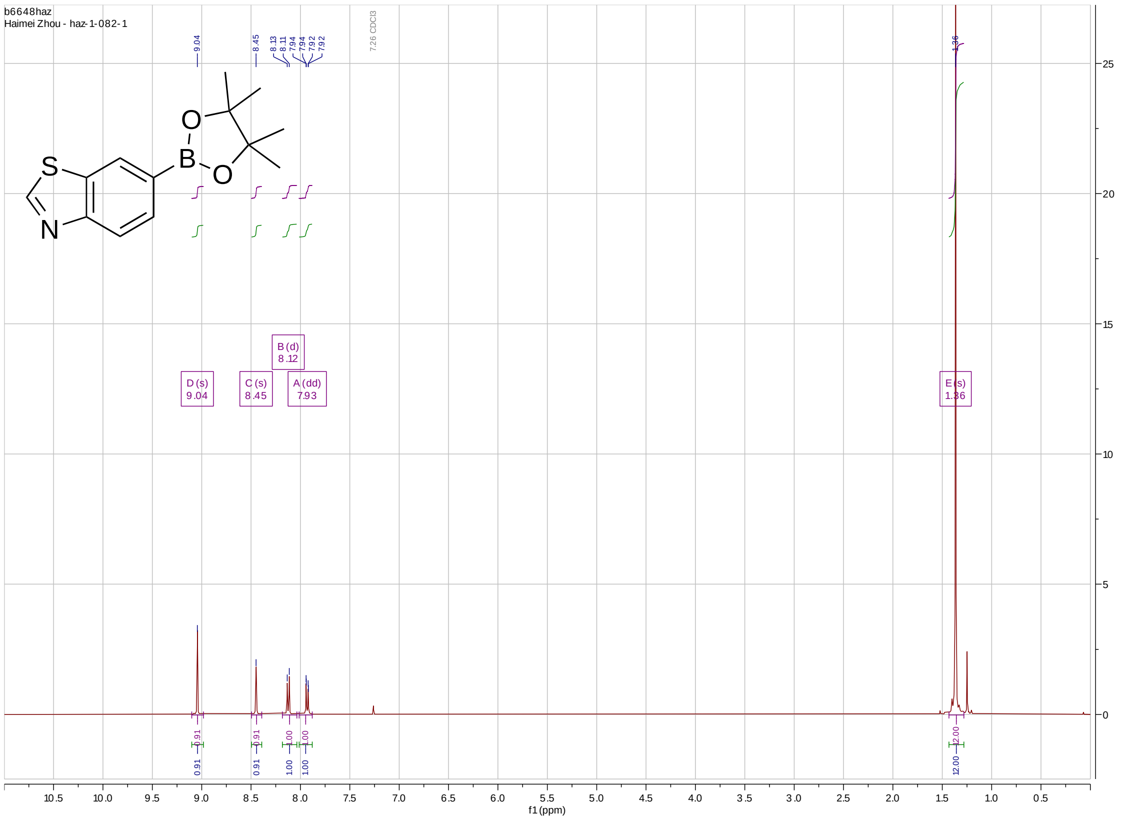

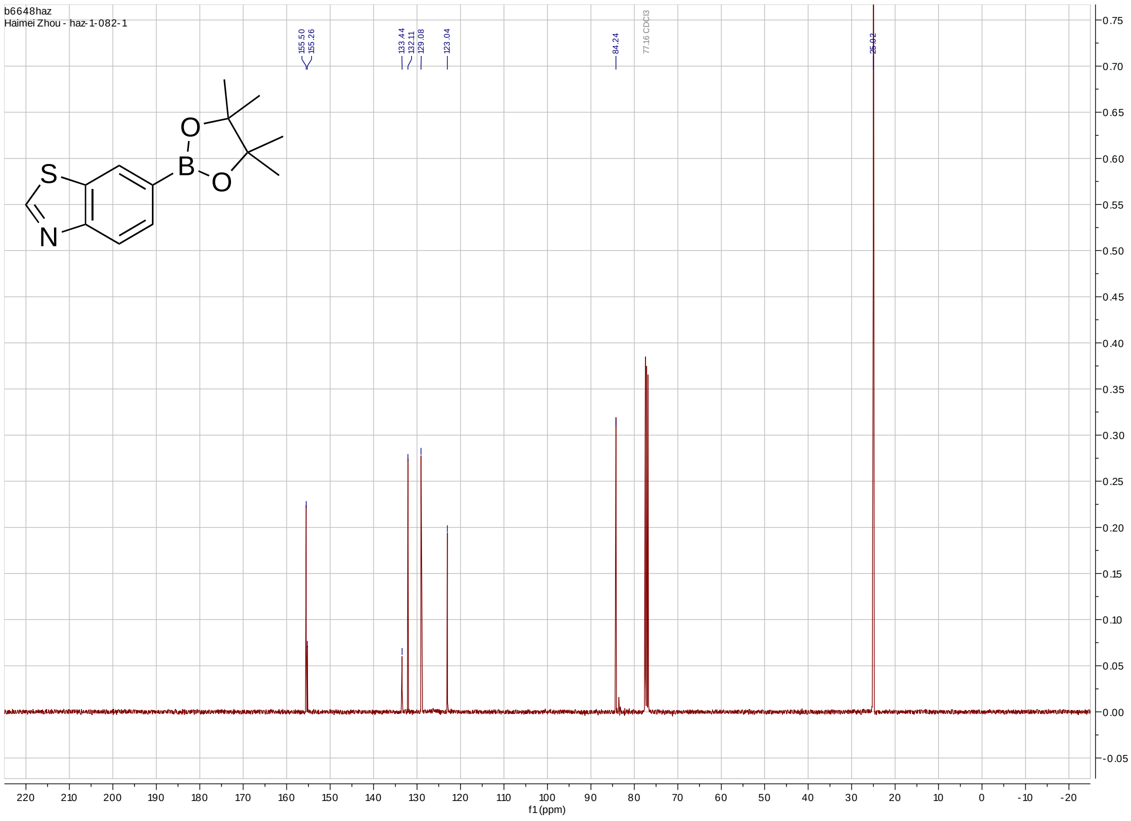

Benzo[d]thiazol-6-ylboronic acid (S2) - δ_H (400 MHz) and $^{13}C\{^1H\}$ NMR (101 MHz)

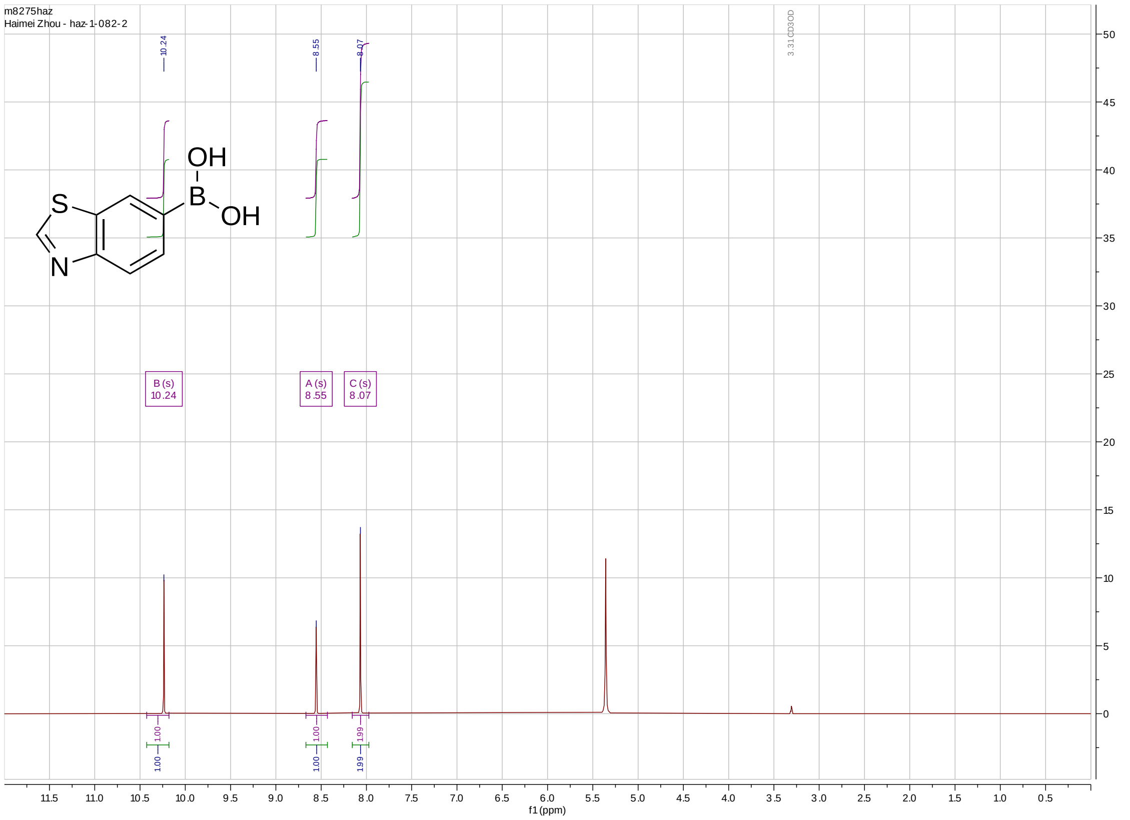

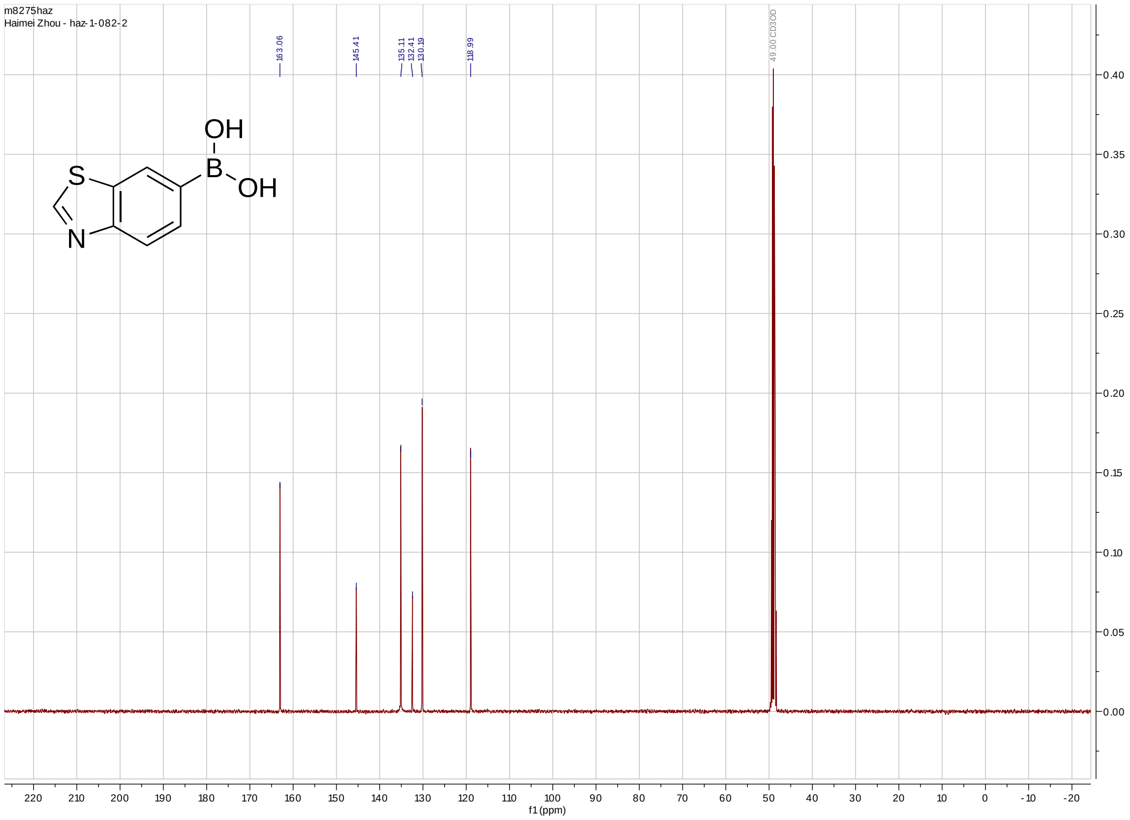

***tert*-Butyl 10-(benzo[d]thiazol-6-yl)-7-oxo-1,2,4a,5,7,12-hexahydrobenzo[f]pyrazino [2,1-c][1,4]oxazocine-3(4H)-carboxylate (5l) -** δ_H (400 MHz) and $^{13}C\{^1H\}$ NMR (101 MHz)

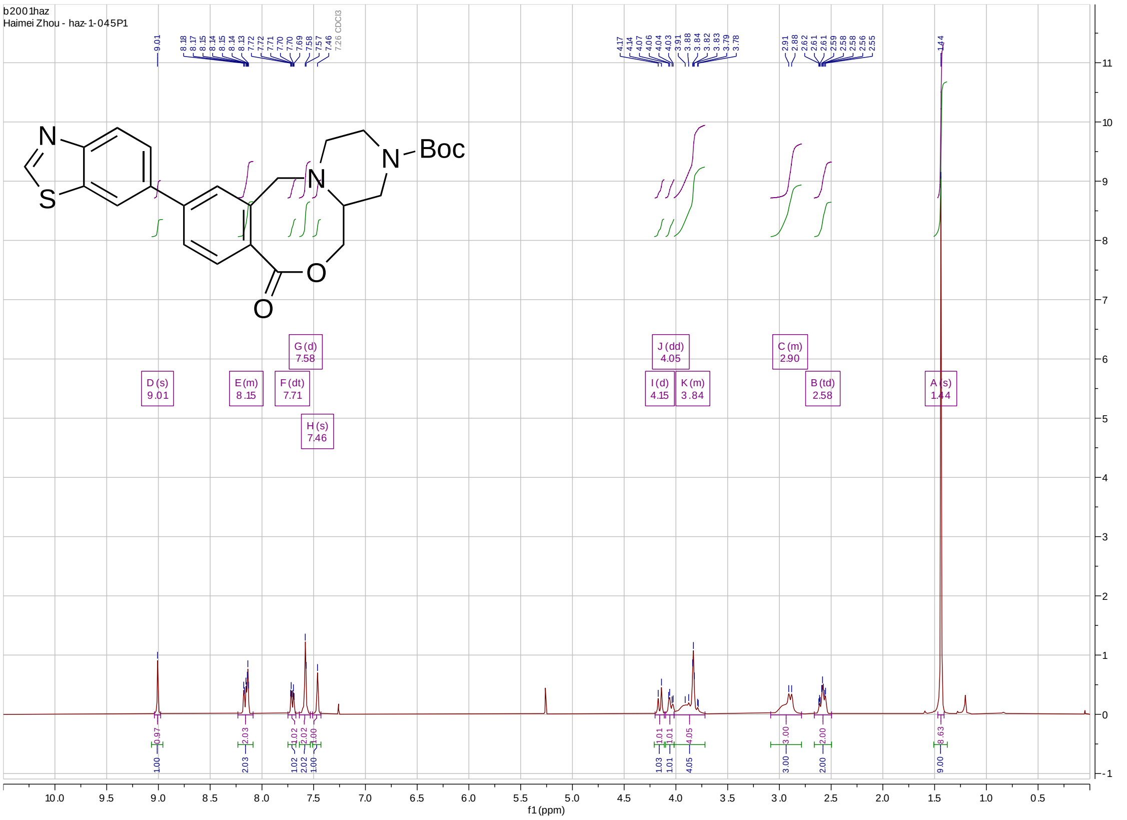

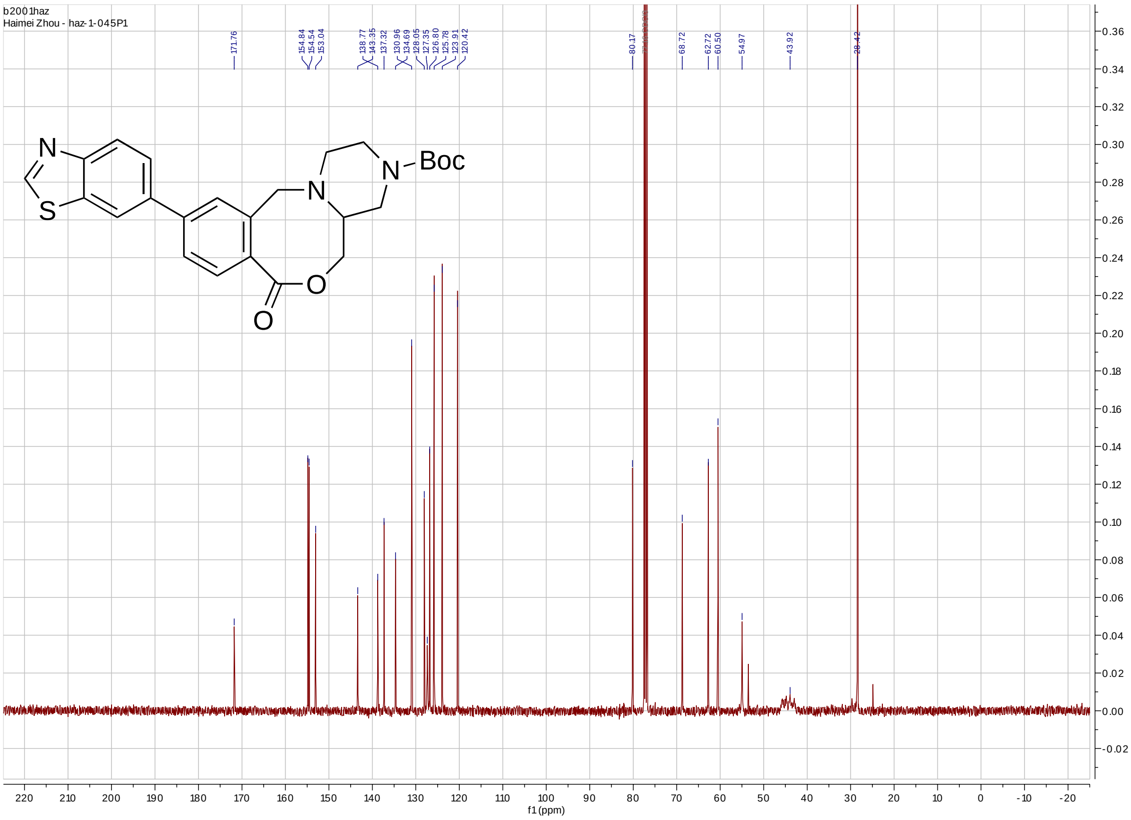

***tert*-Butyl 10-(isoquinolin-6-yl)-7-oxo-1,2,4a,5,7,12-hexahydrobenzo[f]pyrazino[2,1-c][1,4] oxazocine-3(4H)-carboxylate (5m) -** δ$_H$ (400 MHz) and $^{13}C\{^1H\}$ NMR (101 MHz)

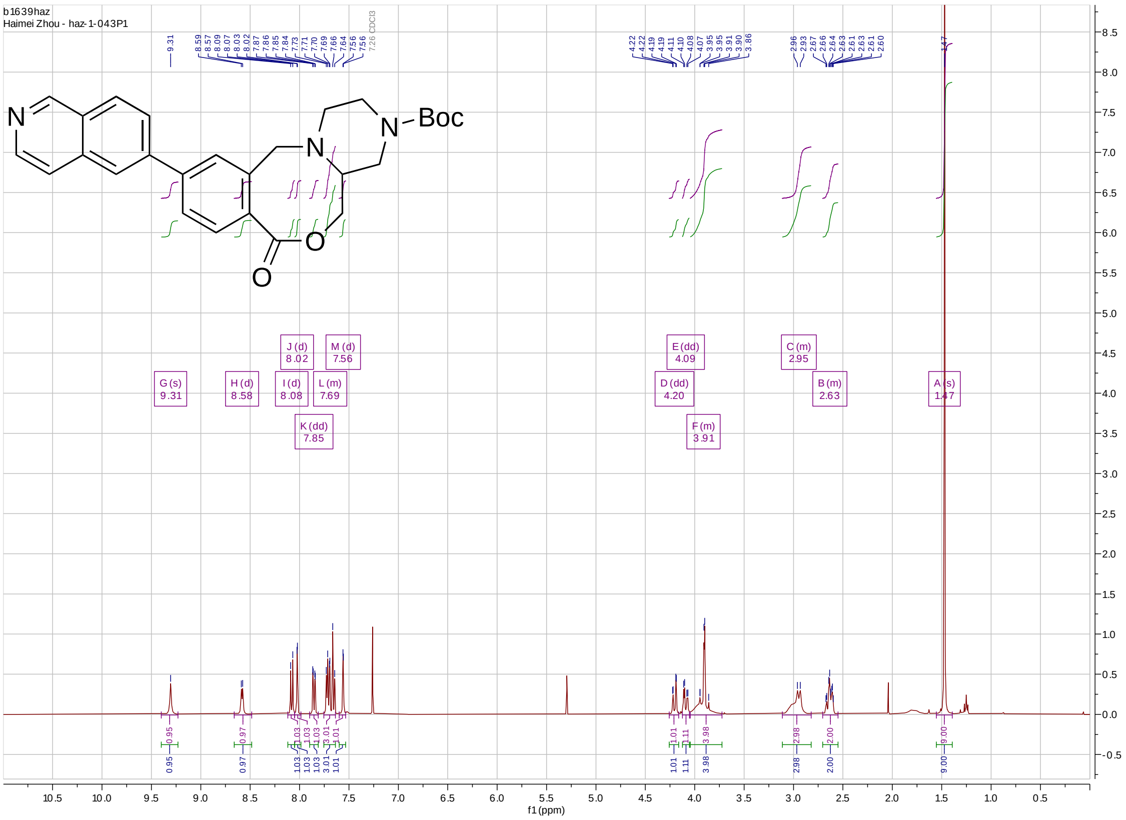

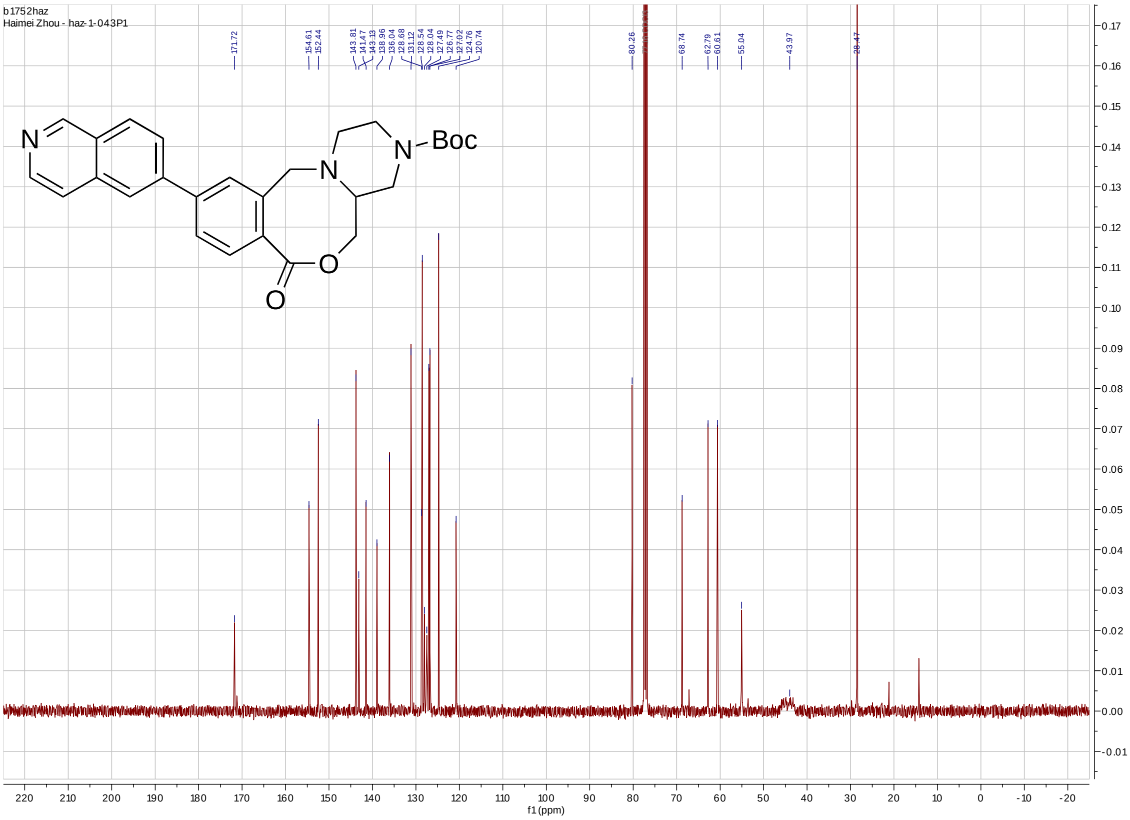

***tert*-Butyl 7-oxo-10-(quinoxalin-6-yl)-1,2,4a,5,7,12-hexahydrobenzo[f]pyrazino[2,1-c][1,4] oxazocine-3(4H)-carboxylate (5n) - δ_H (400 MHz) and $^{13}C\{^1H\}$ NMR (101 MHz)**

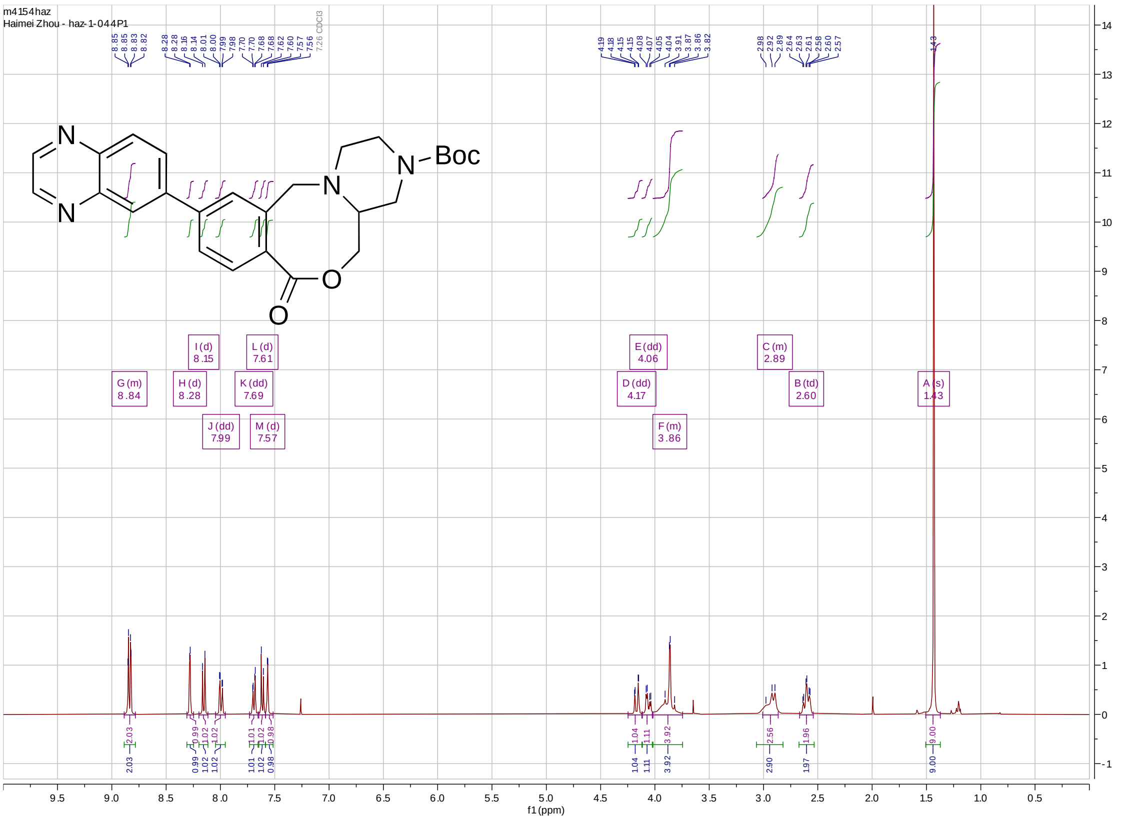

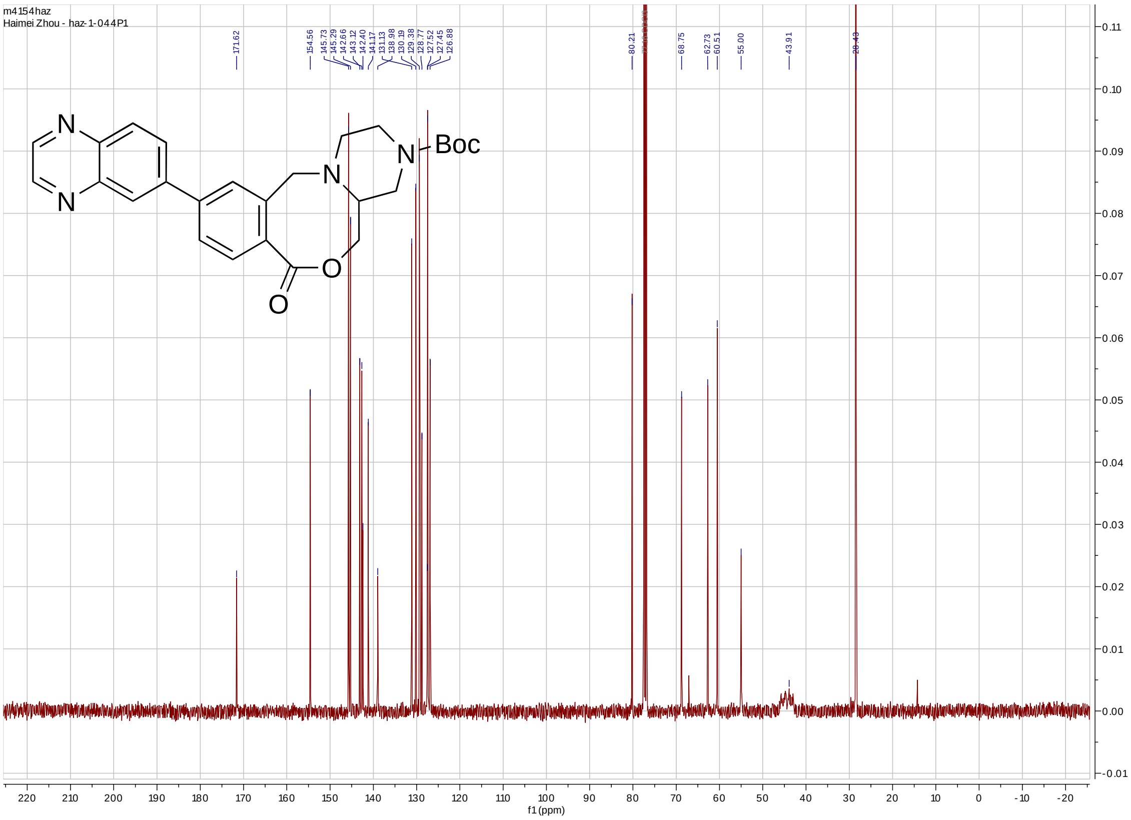

tert-Butyl 10-(1-methyl-1H-benzo[d]imidazol-6-yl)-7-oxo-1,2,4a,5,7,12-hexahydrobenzo[f]pyrazino[2,1-c][1,4]oxazocine-3(4H)-carboxylate (5o) - δ_H (400 MHz) and $^{13}C\{^1H\}$ NMR (101 MHz)

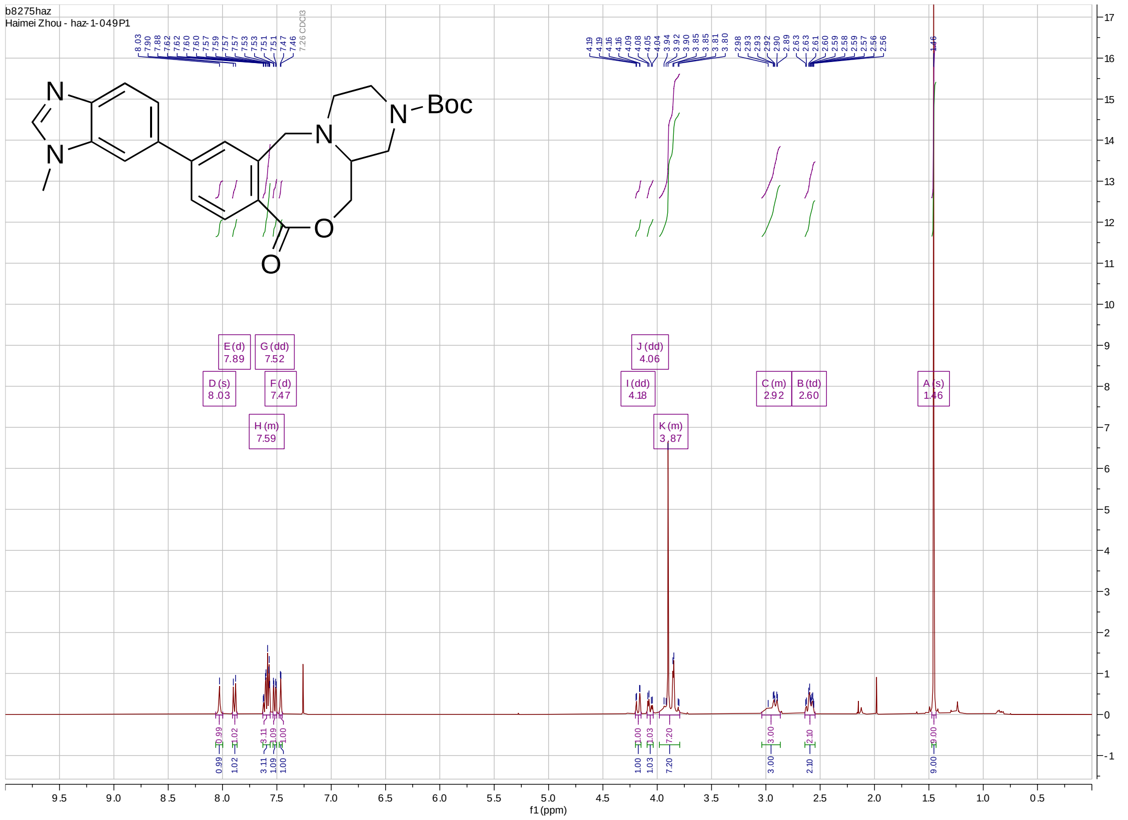

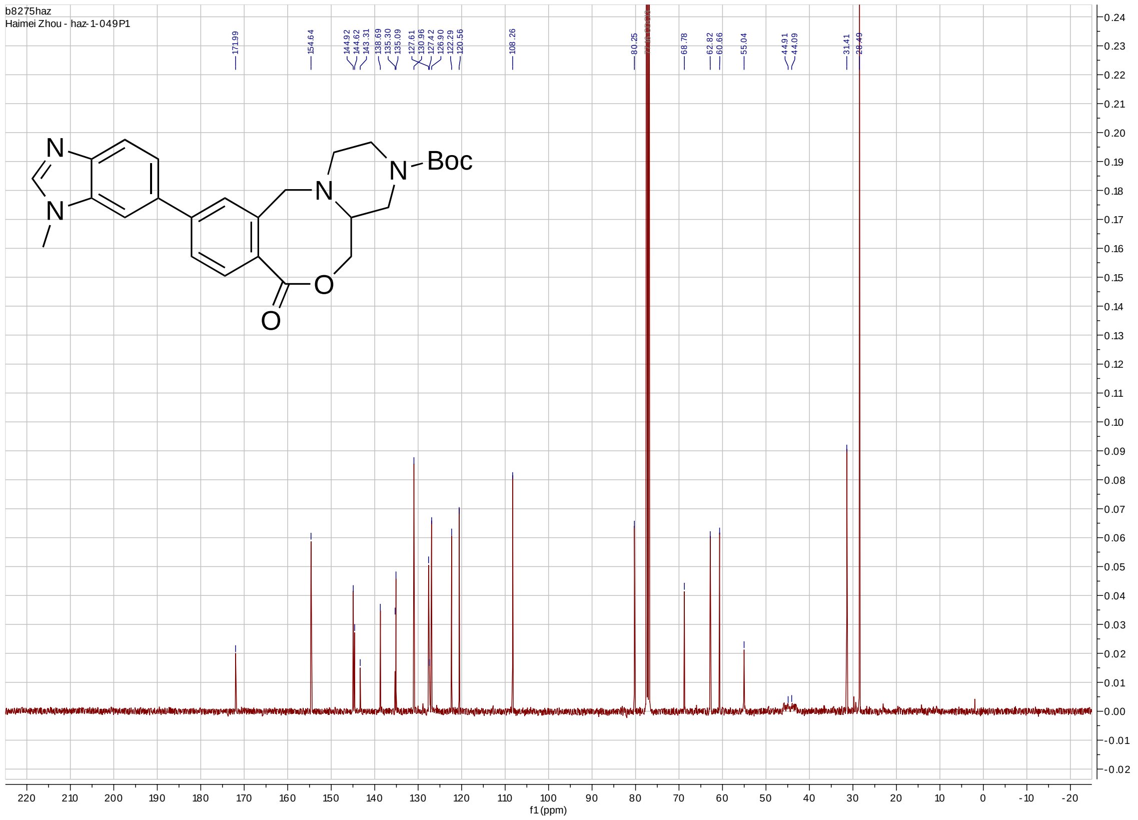

***tert*-Butyl 10-(3,4-dimethoxyphenyl)-7-oxo-1,2,4a,5,7,12-hexahydrobenzo[f] pyrazino [2,1-c][1,4]oxazocine-3(4H)-carboxylate (5p) -** δ_H (400 MHz) and $^{13}C\{^1H\}$ NMR (101 MHz)

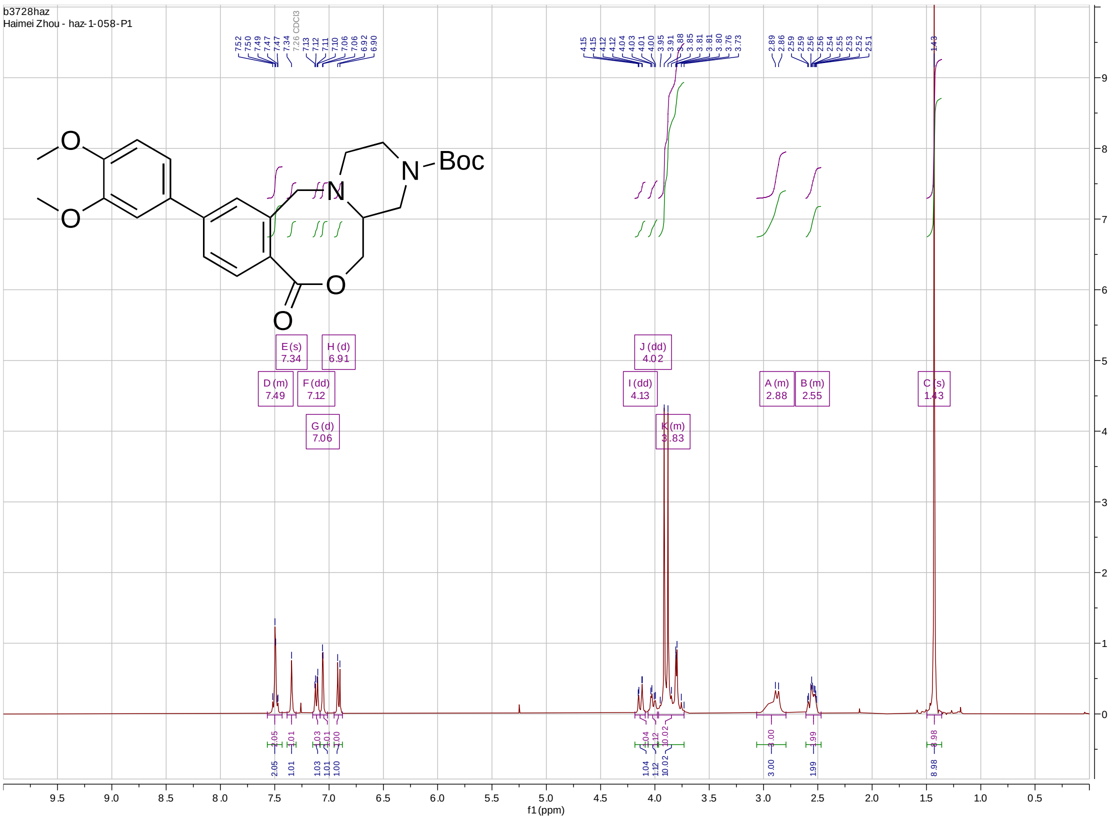

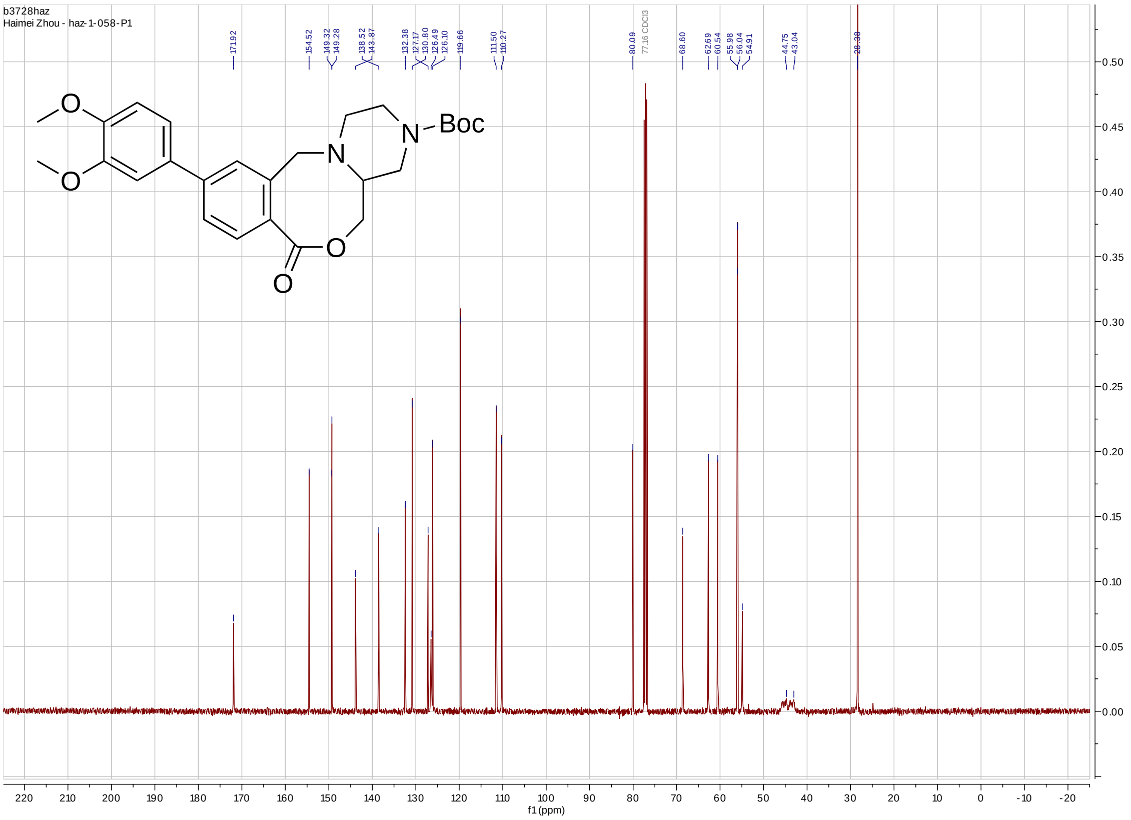

***tert*-Butyl 10-(5-fluoro-3-methylpyridin-2-yl)-7-oxo-1,2,4a,5,7,12-hexahydrobenzo[f]pyrazino[2,1-c][1,4]oxazocine-3(4H)-carboxylate (5q)** - δ_H (400 MHz) and $^{13}C\{^1H\}$ NMR (101 MHz)

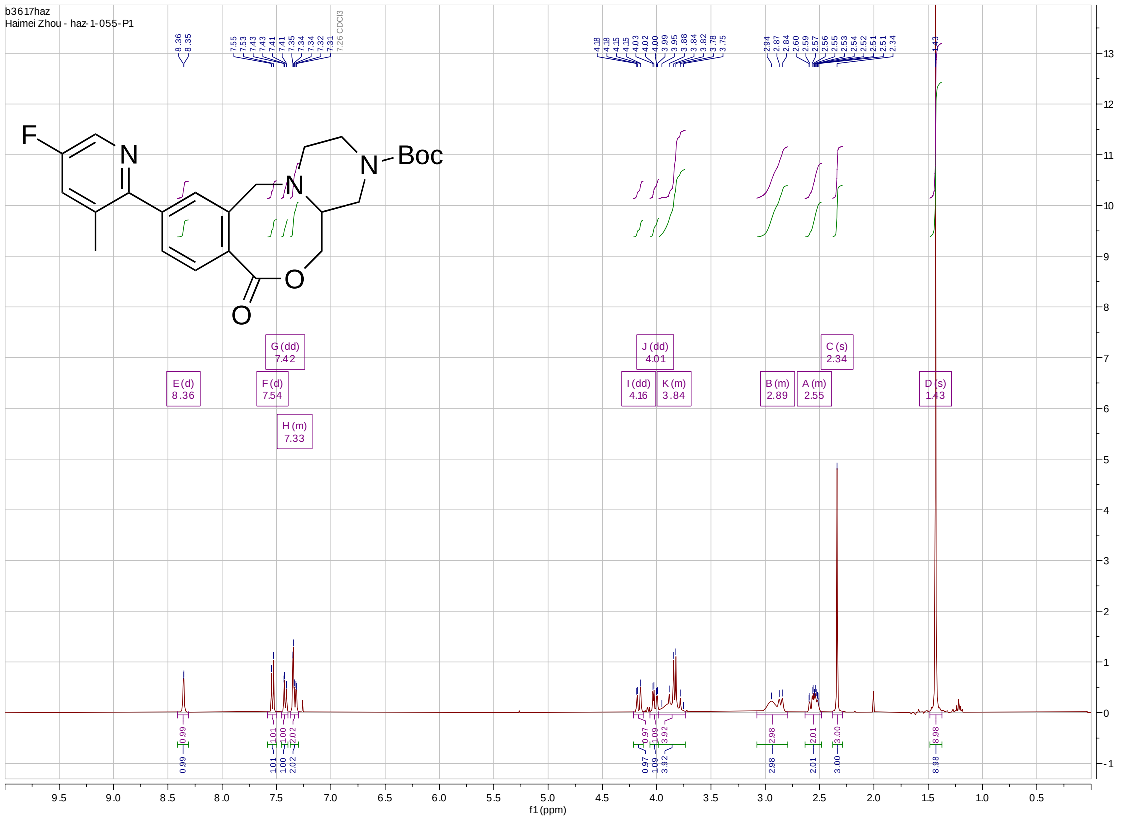

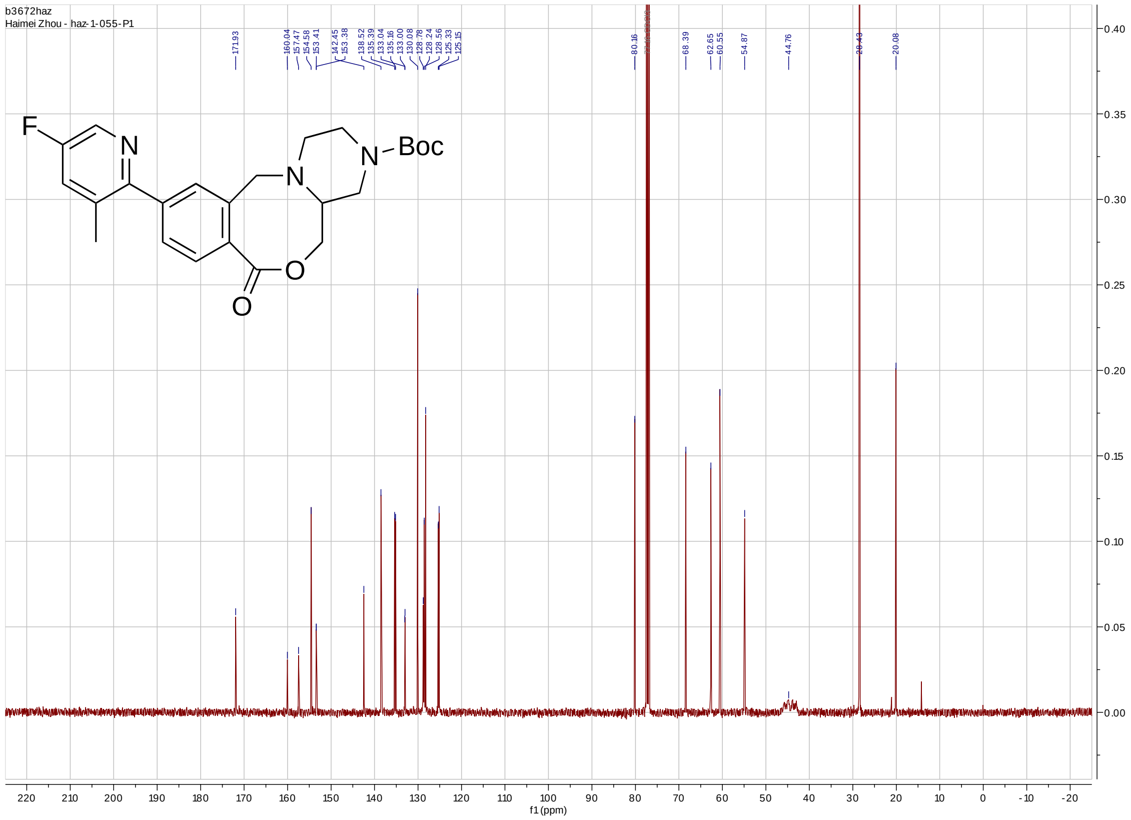

***tert*-Butyl 10-(3,5-dimethylisoxazol-4-yl)-7-oxo-1,2,4a,5,7,12-hexahydrobenzo[f]pyrazino[2,1-c][1,4]oxazocine-3(4H)-carboxylate (5r)** - δ_H (400 MHz) and $^{13}C\{^1H\}$ NMR (101 MHz)

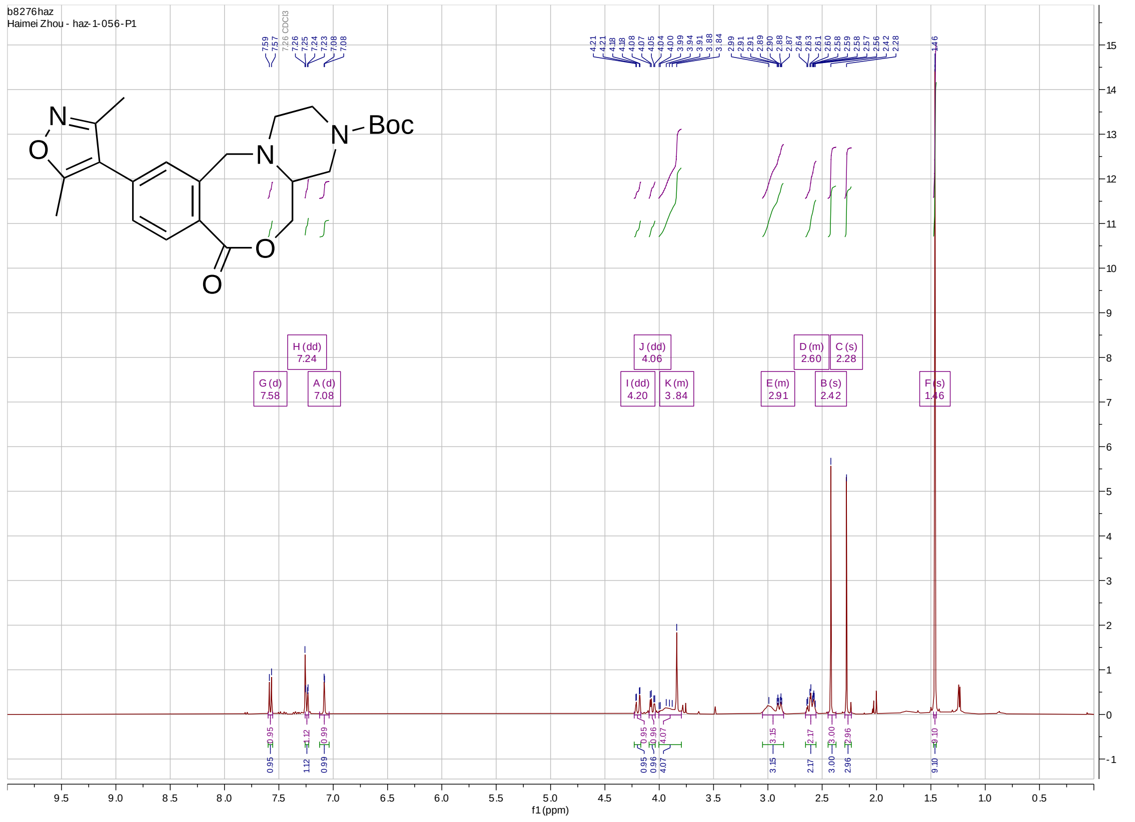

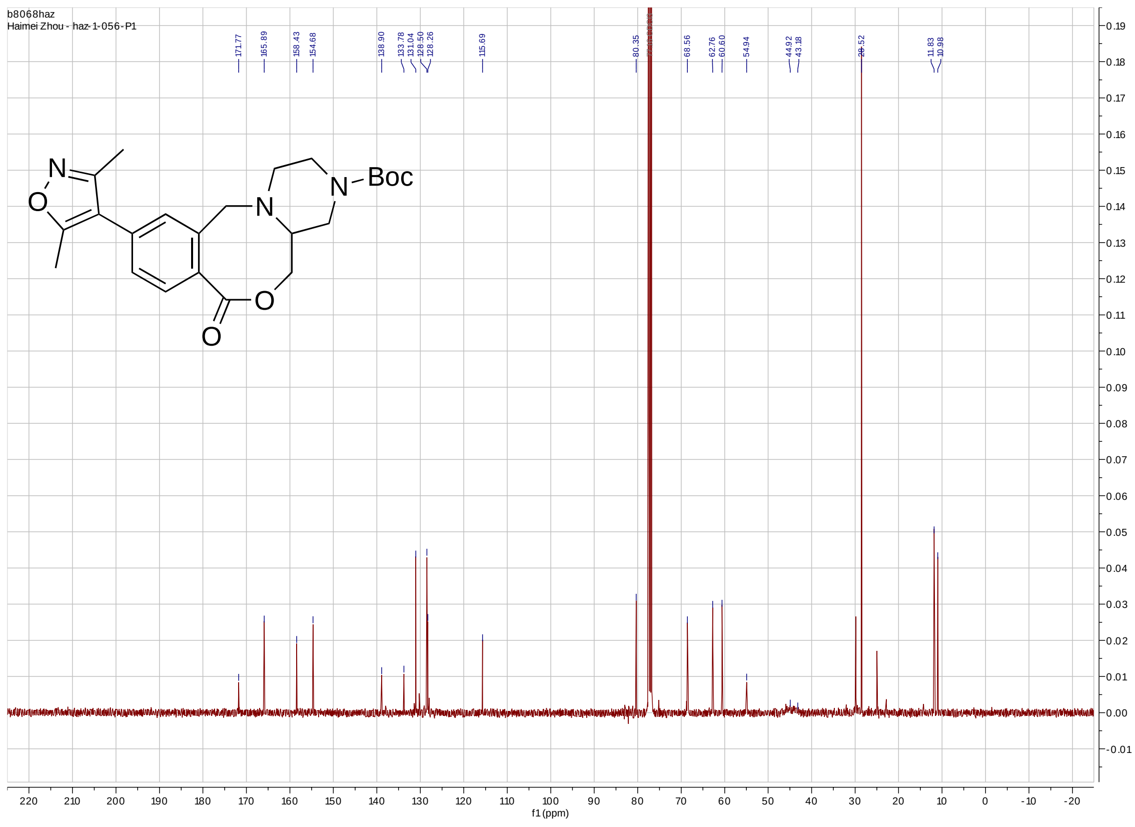

***tert*-Butyl 10-(2-methoxypyrimidin-5-yl)-7-oxo-1,2,4a,5,7,12-hexahydrobenzo[f]pyrazino[2,1-c][1,4]oxazocine-3(4H)-carboxylate (5s) -** δ_H (400 MHz) and $^{13}C\{^1H\}$ NMR (101 MHz)

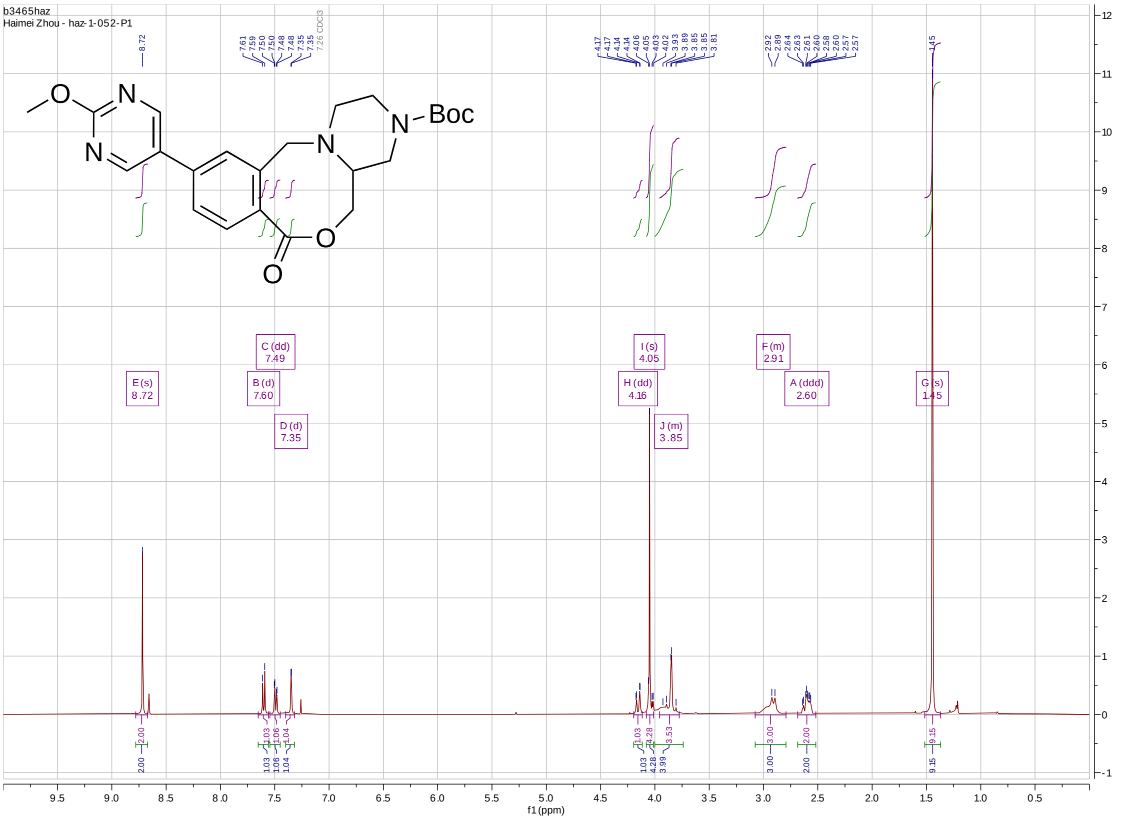

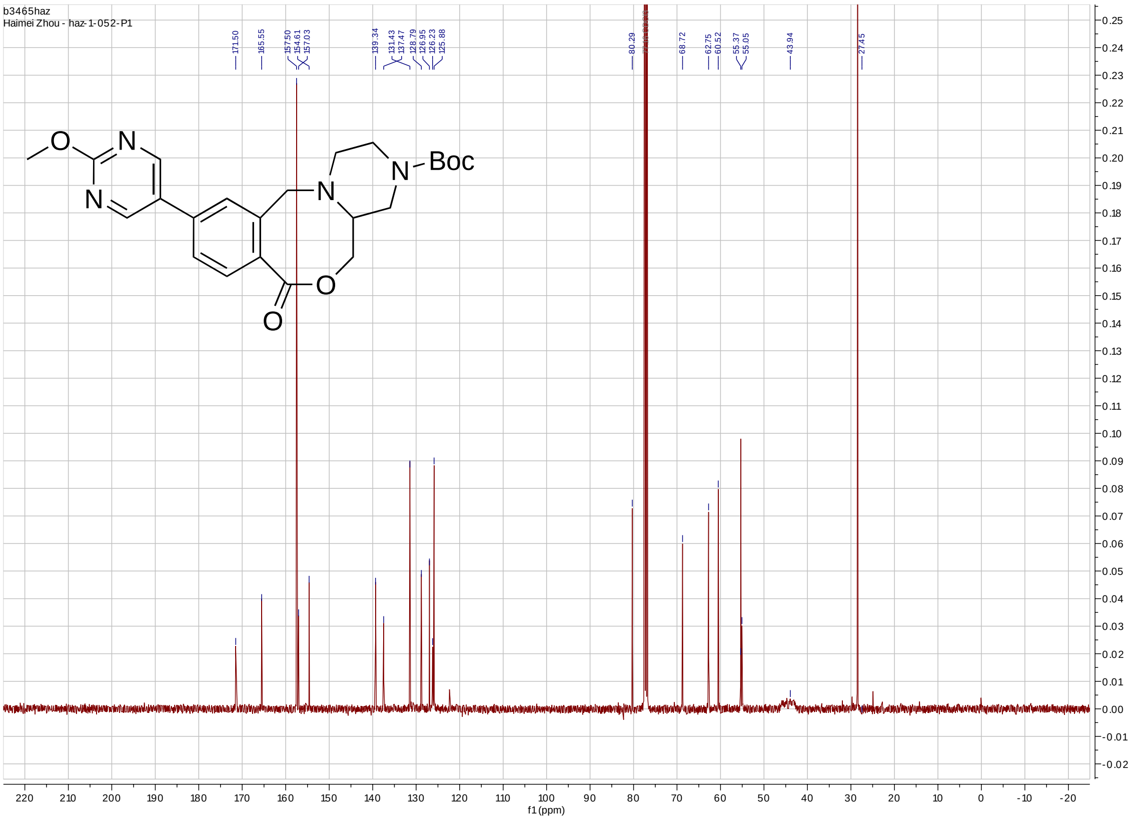

***tert*-Butyl 10-(1,3-dimethyl-2,4-dioxo-1,2,3,4-tetrahydropyrimidin-5-yl)-7-oxo-1,2,4a,5,7,12-hexahydrobenzo[f]pyrazino[2,1-c][1,4]oxazocine-3(4H)-carboxylate (5t) - δ_H** (400 MHz) and $^{13}C\{^1H\}$ NMR (101 MHz)

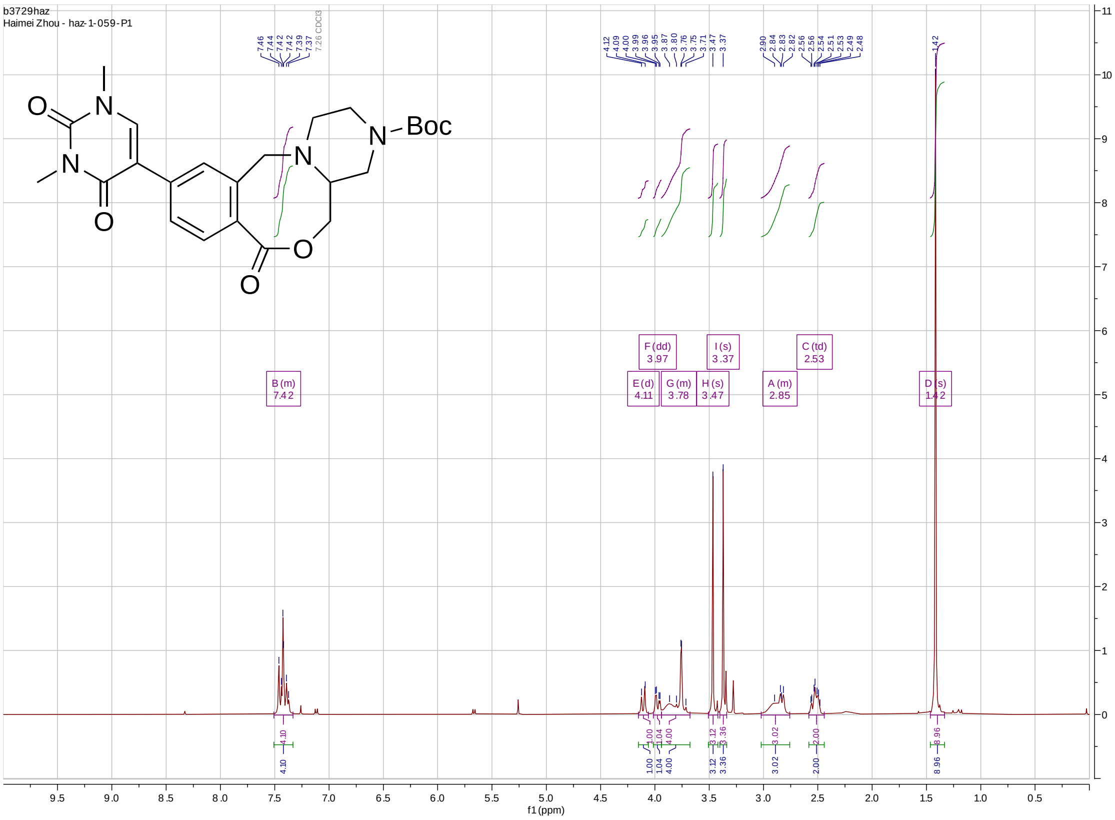

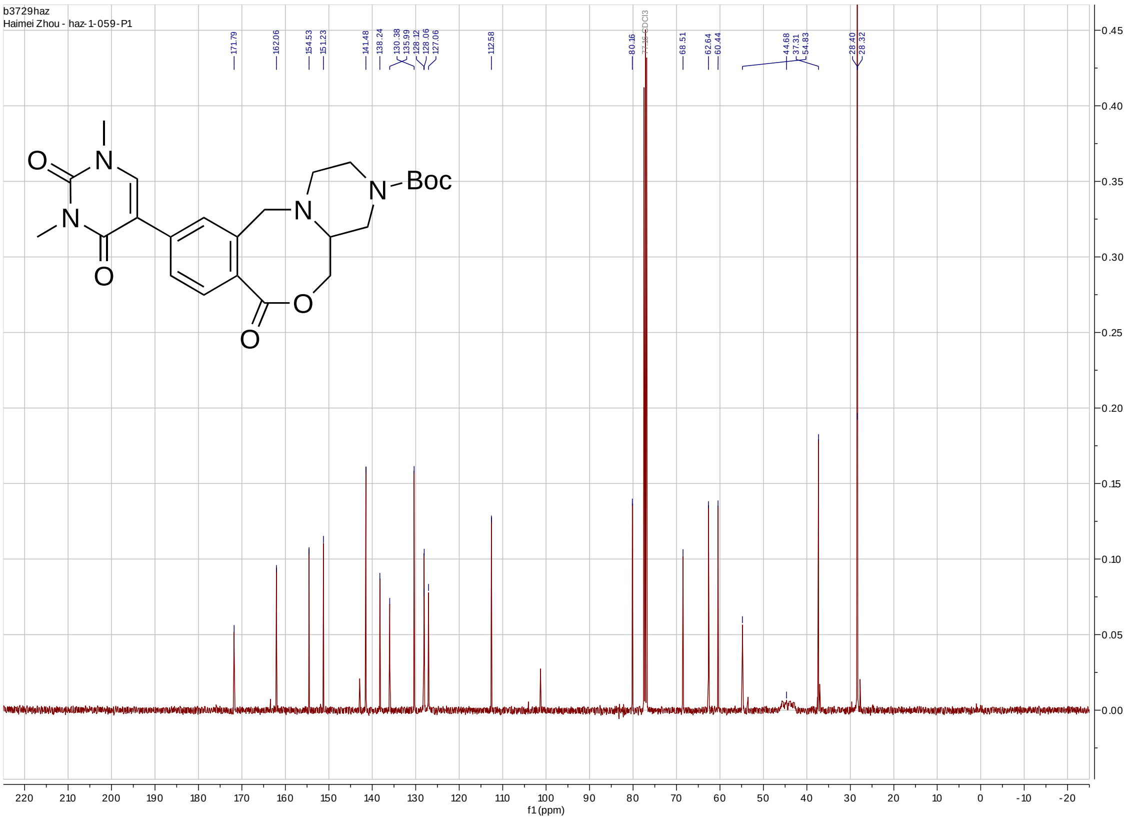

10-(4-Fluoro-2-methoxyphenyl)-1,2,3,4,4a,5-hexahydrobenzo[f]pyrazino[2,1-c][1,4] oxazocin-7(12H)-one (S3) - δ_H (400 MHz) and $^{13}C\{^1H\}$ NMR (101 MHz)

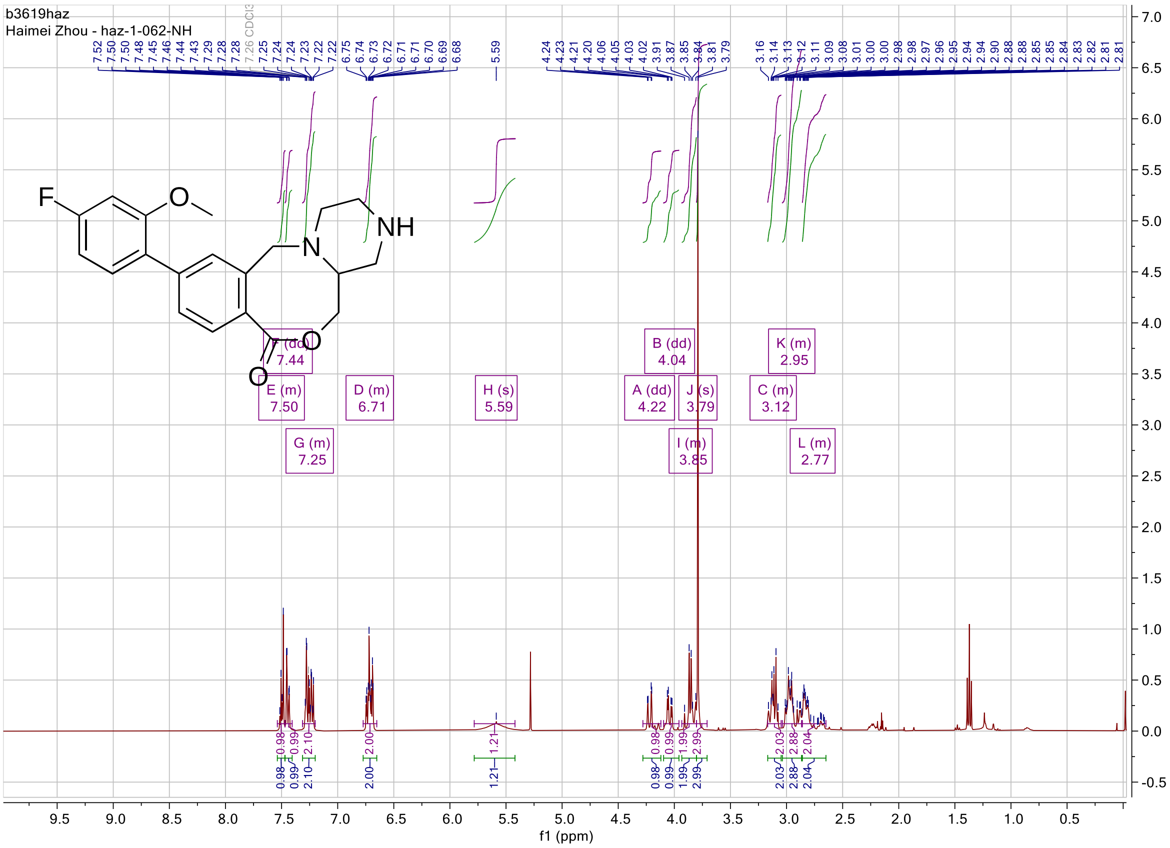

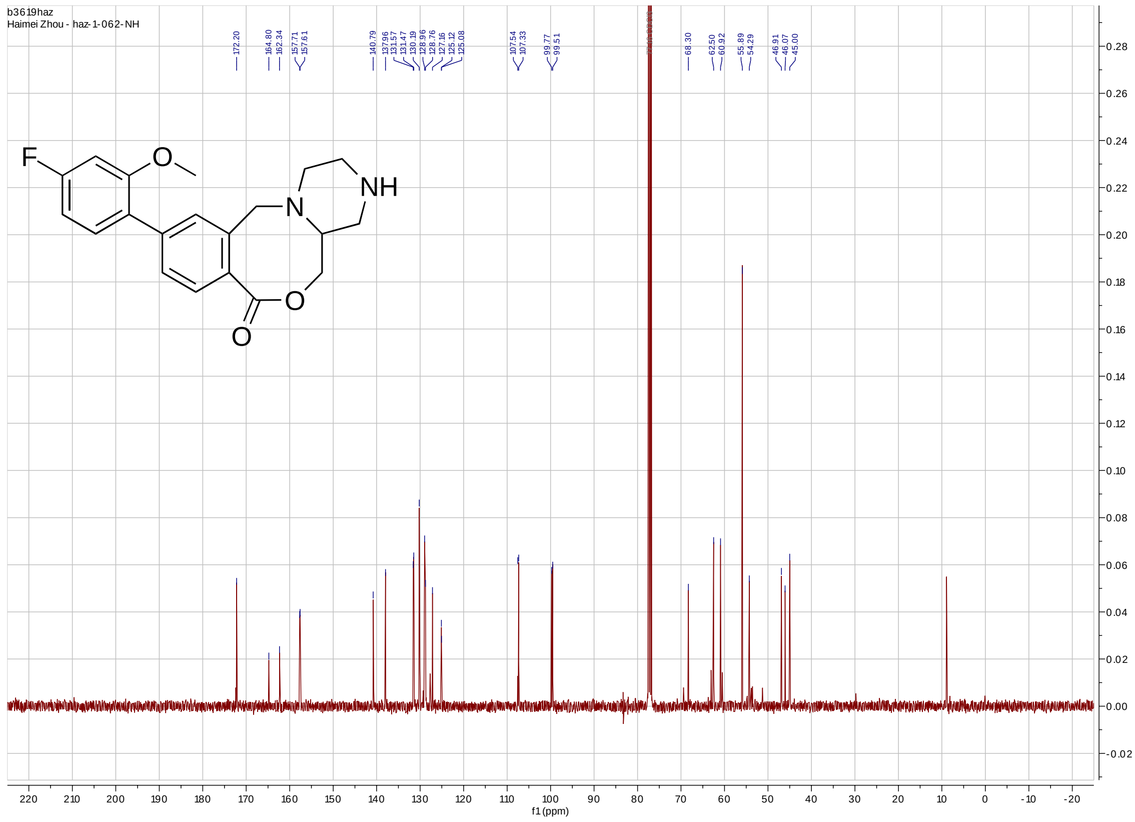

3-((3,5-Dimethylisoxazol-4-yl)ulfonyl)-10-(4-fluoro-2-methoxyphenyl)-1,2,3,4,4a,5-hexahydrobenzo[f]pyrazino[2,1-c][1,4]oxazocin-7(12H)-one (6a) - δ_H (400 MHz) and $^{13}C\{^1H\}$ NMR (101 MHz)

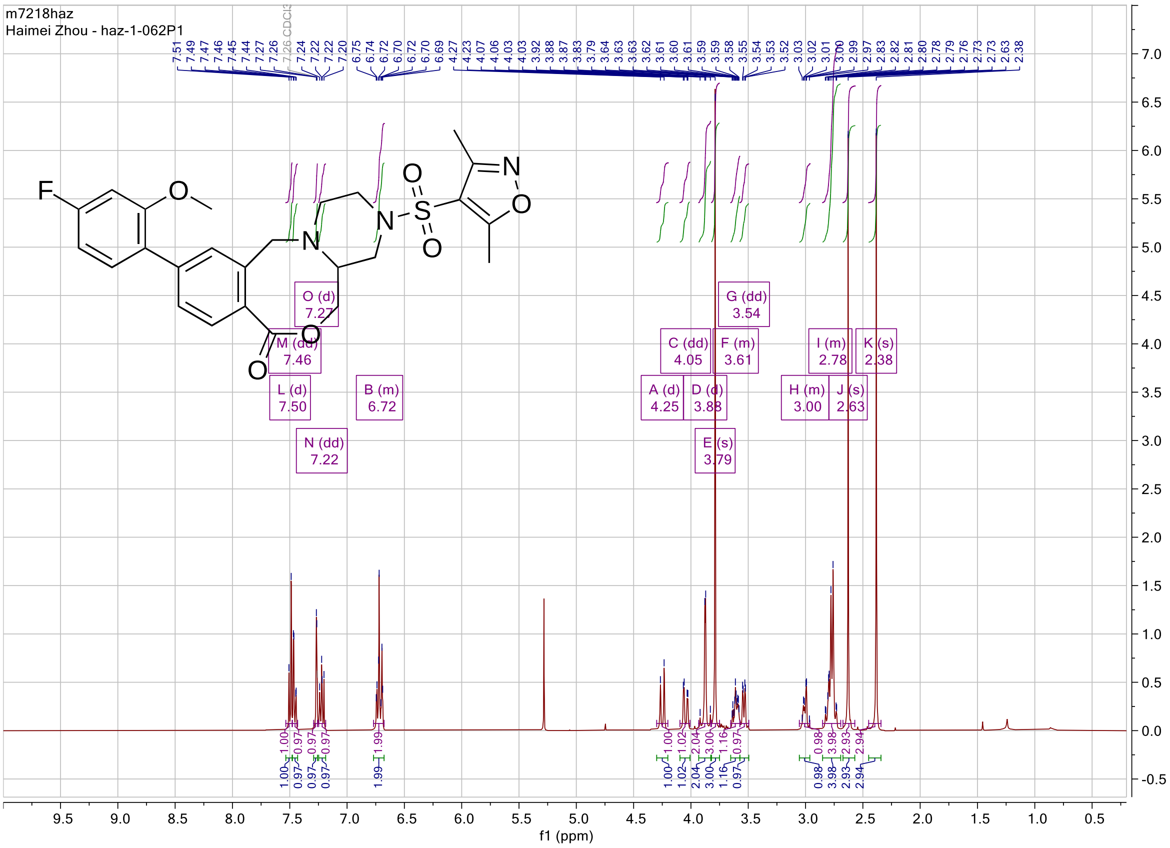

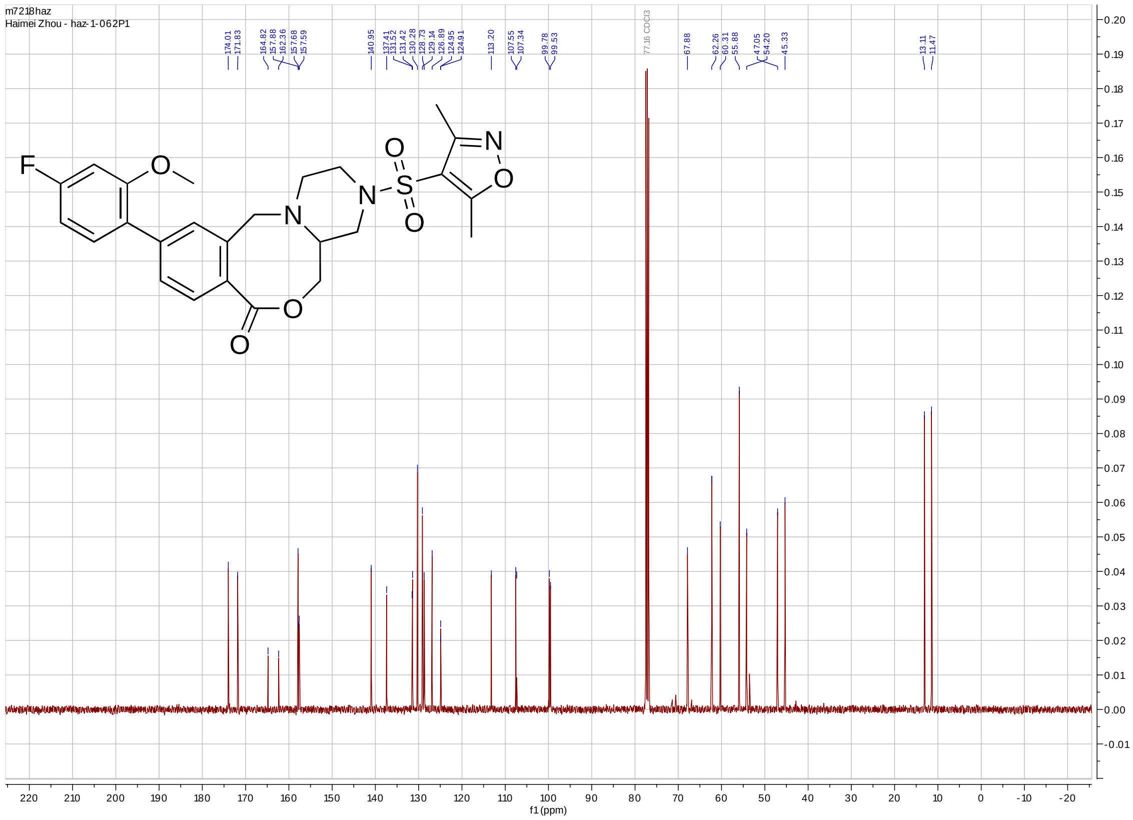

10-(4-Fluoro-2-methoxyphenyl)-3-((5-methylfuran-2-yl)methyl)-1,2,3,4,4a,5-hexahydrobenzo[f]pyrazino[2,1-c][1,4]oxazocin-7(12H)-one (6b) - δ_H (400 MHz) and $^{13}C\{^1H\}$ NMR (101 MHz)

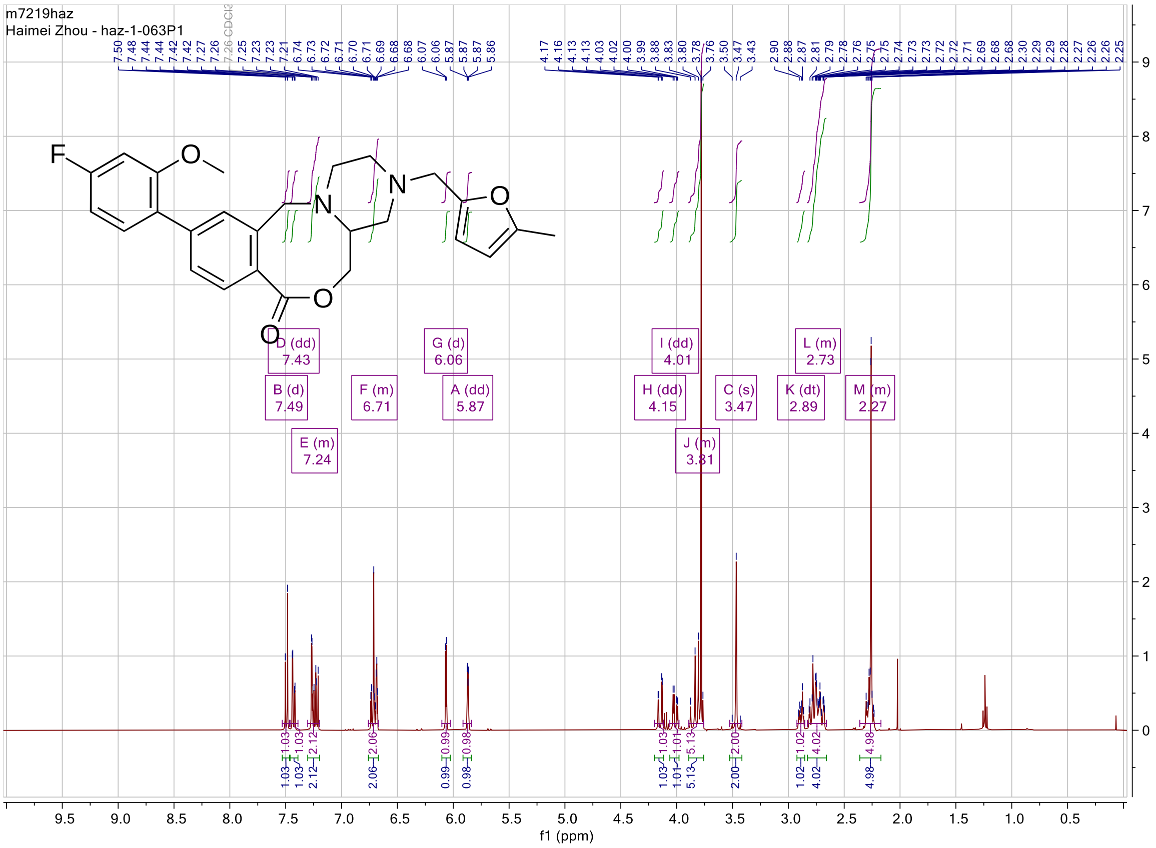

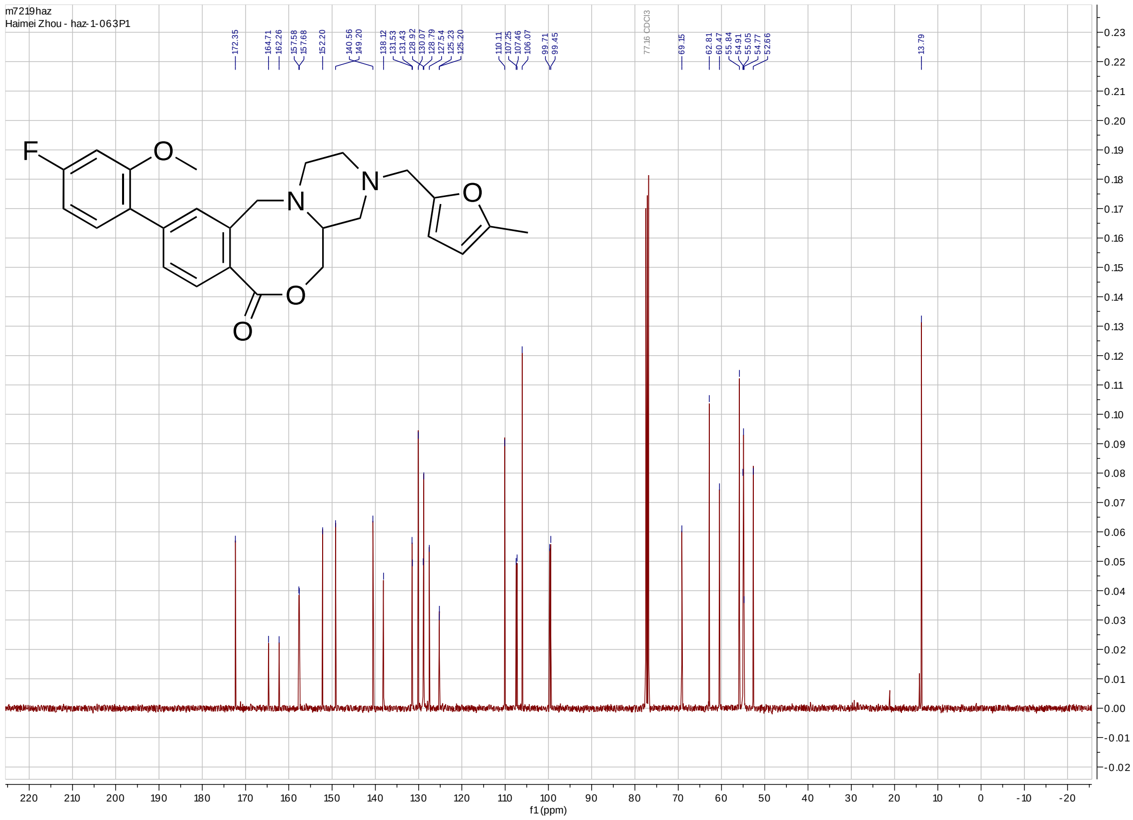

3-(cyclopentanecarbonyl)-10-(4-fluoro-2-methoxyphenyl)-1,2,3,4,4a,5-hexahydrobenzo[f]pyrazino[2,1-c][1,4]oxazocin-7(12H)-one (6c) - δ_H (400 MHz) and $^{13}C\{^1H\}$ NMR (101 MHz)

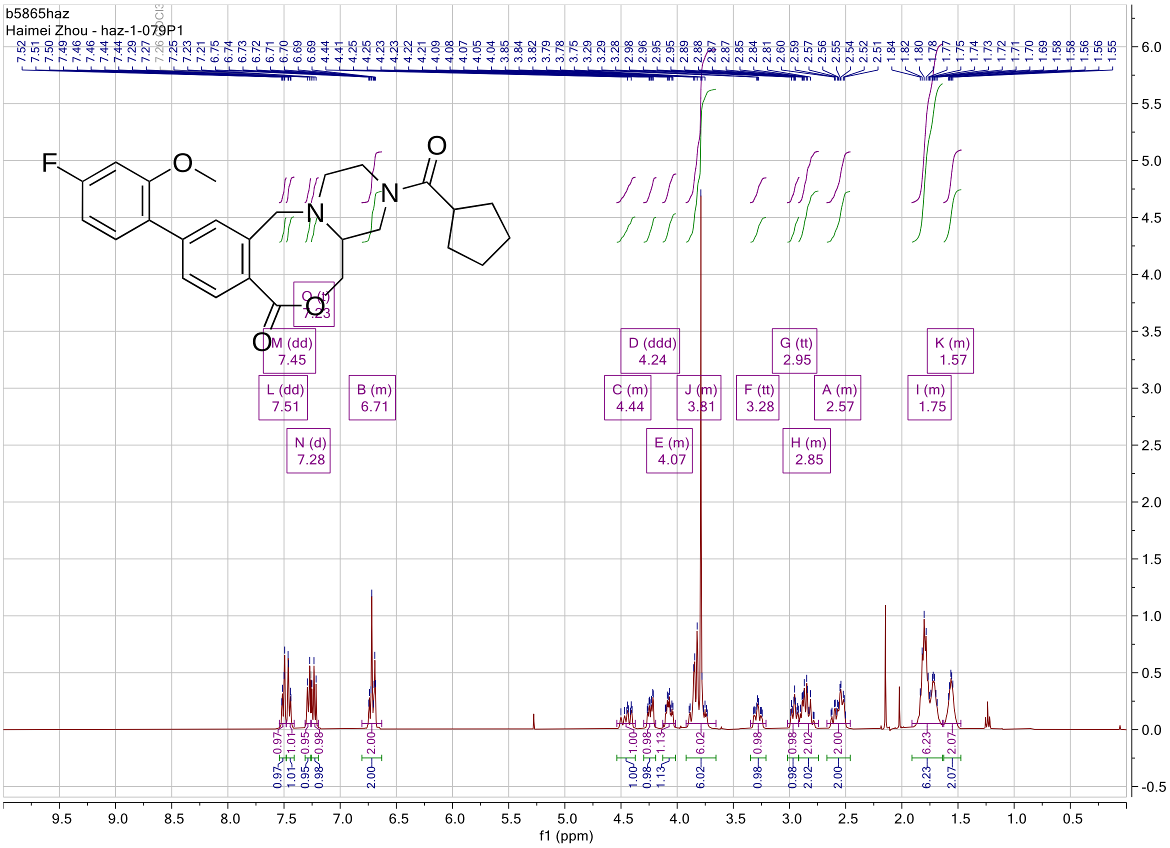

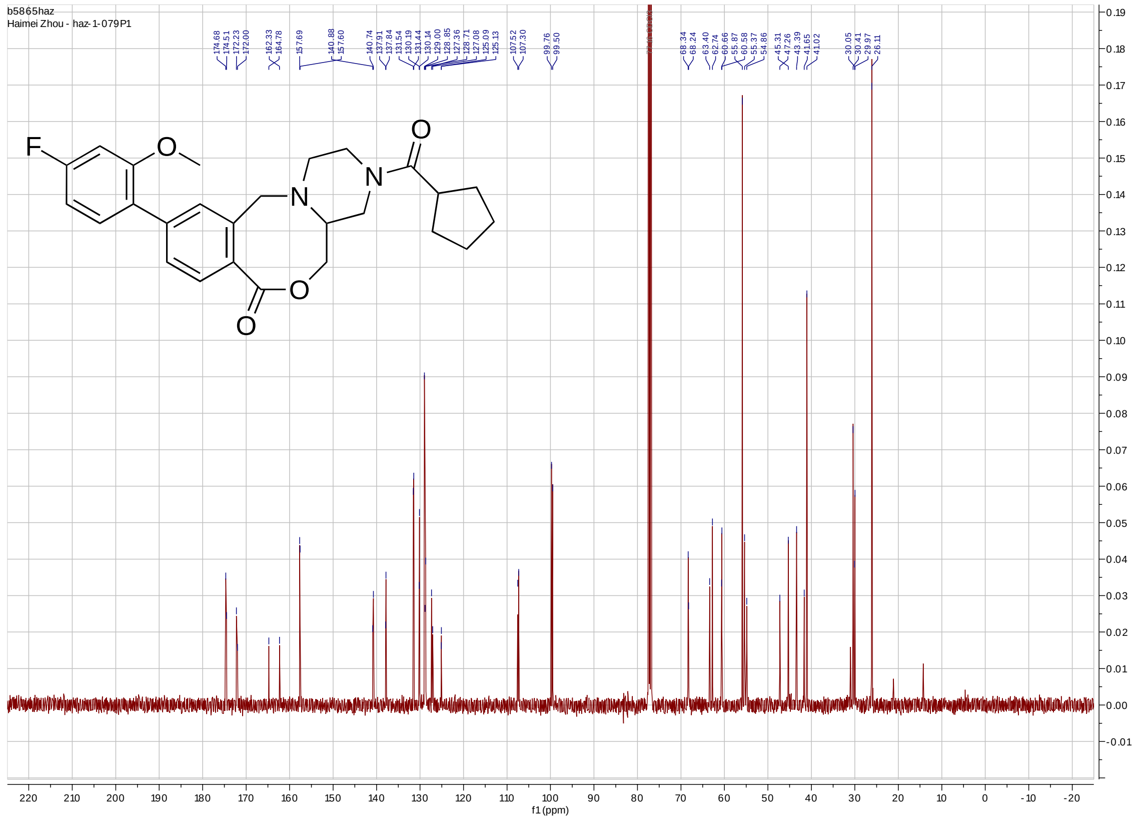

10-(4-Fluoro-2-methoxyphenyl)-3-(4-(trifluoromethyl)benzyl)-1,2,3,4,4a,5-hexahydrobenzo[f]pyrazino[2,1-c][1,4]oxazocin-7(12H)-one (6d) - δ_H (400 MHz) and $^{13}C\{^1H\}$ NMR (101 MHz)

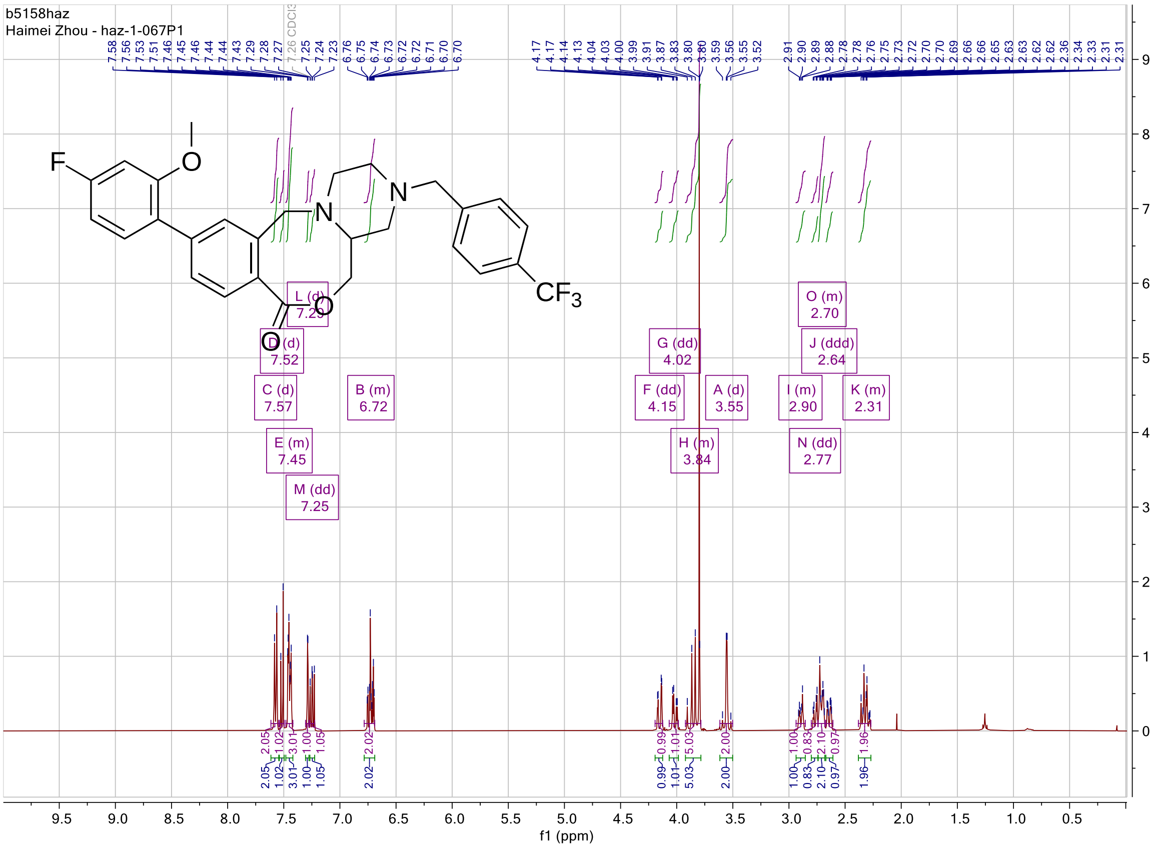

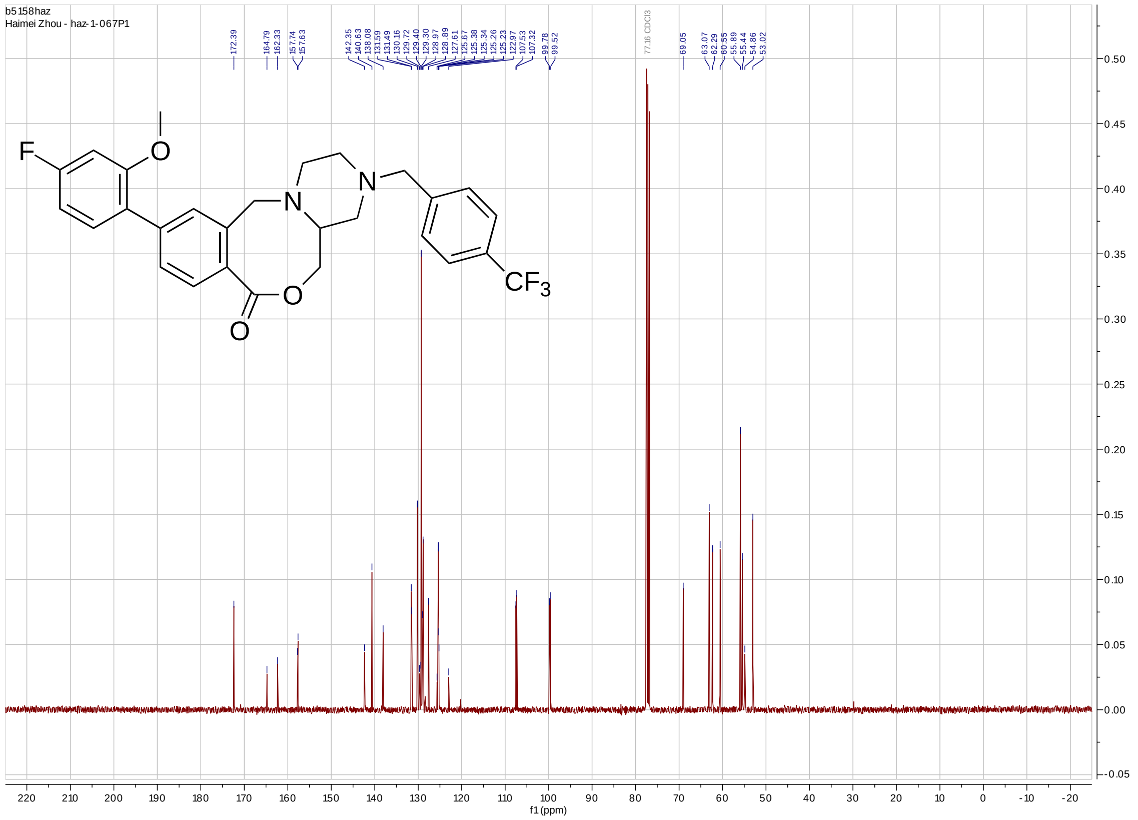

10-(4-fluoro-2-methoxyphenyl)-3-(pyrimidin-5-yl)-1,2,3,4,4a,5-hexahydrobenzo[f] pyrazino[2,1-c][1,4]oxazocin-7(12H)-one (6e) - δ_H (400 MHz) and $^{13}C\{^1H\}$ NMR (101 MHz)

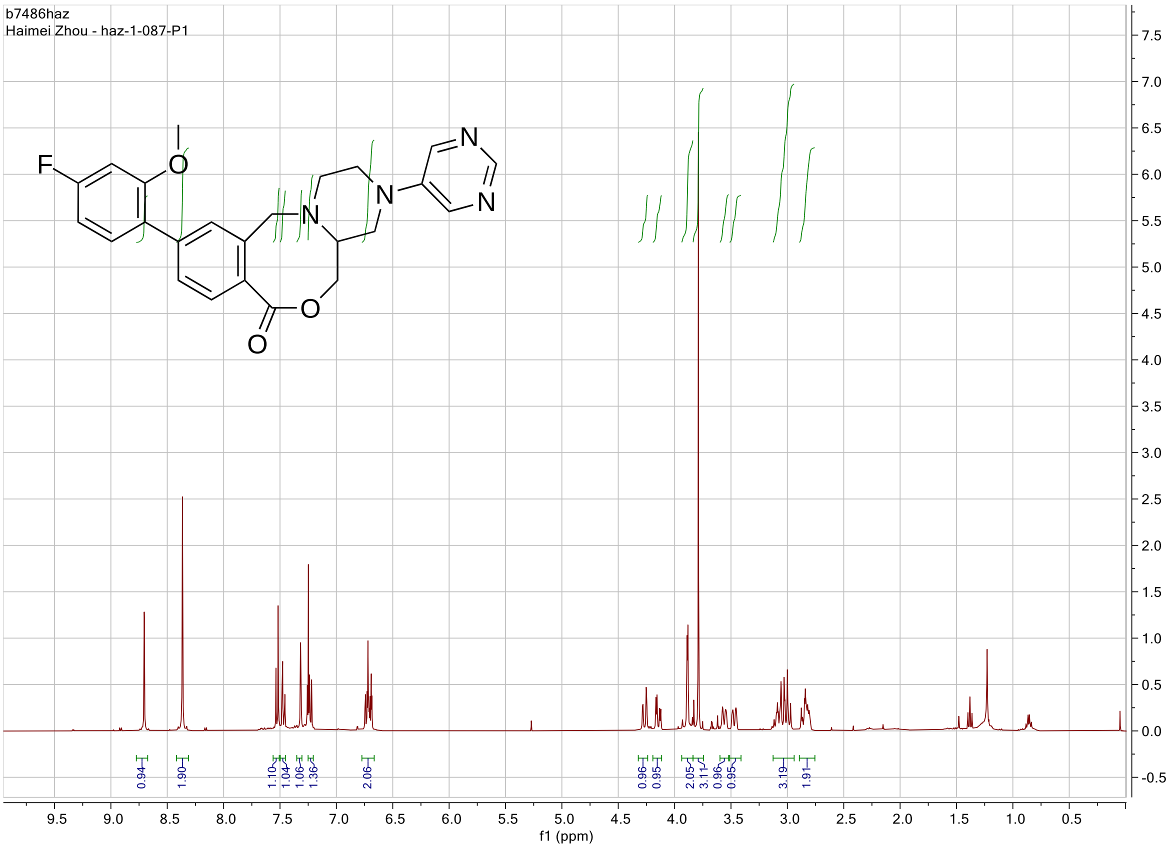

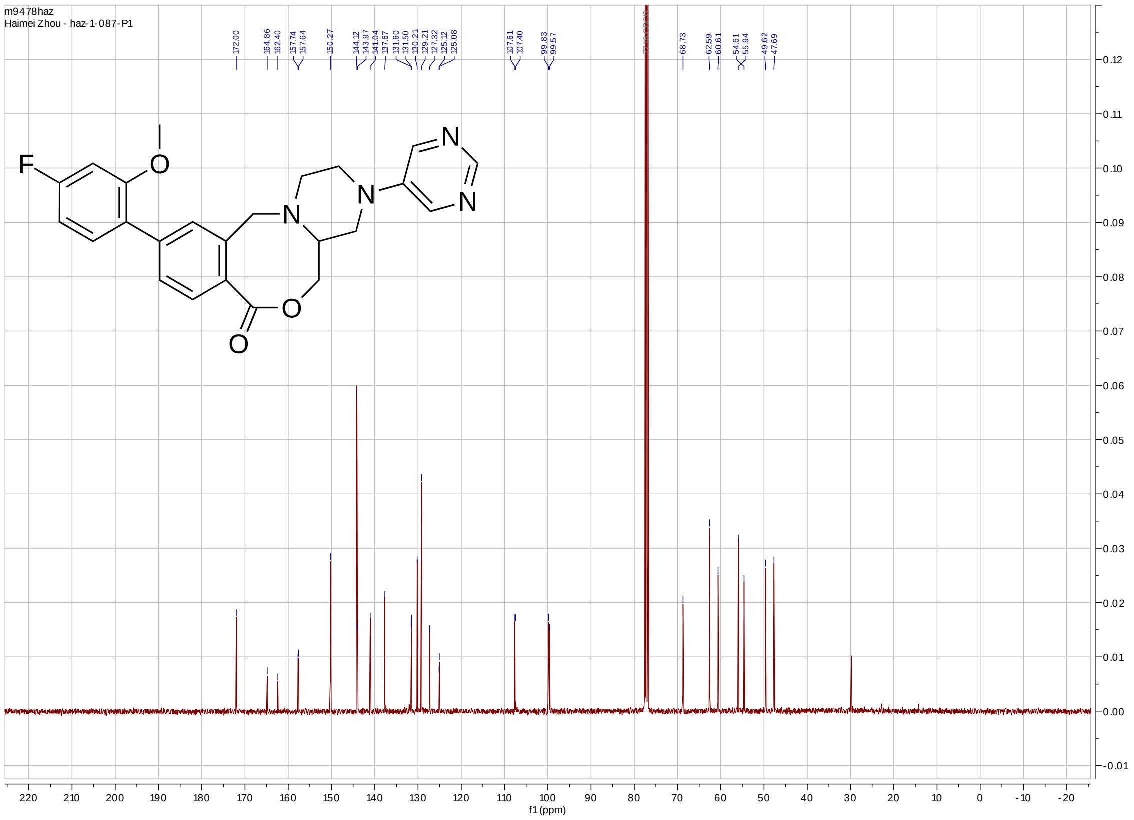

10-(Benzo[d]thiazol-6-yl)-1,2,3,4,4a,5-hexahydrobenzo[f]pyrazino[2,1-c][1,4] oxazocin-7(12H)-one (S4) - δ_H (400 MHz) and $^{13}C\{^1H\}$ NMR (101 MHz)

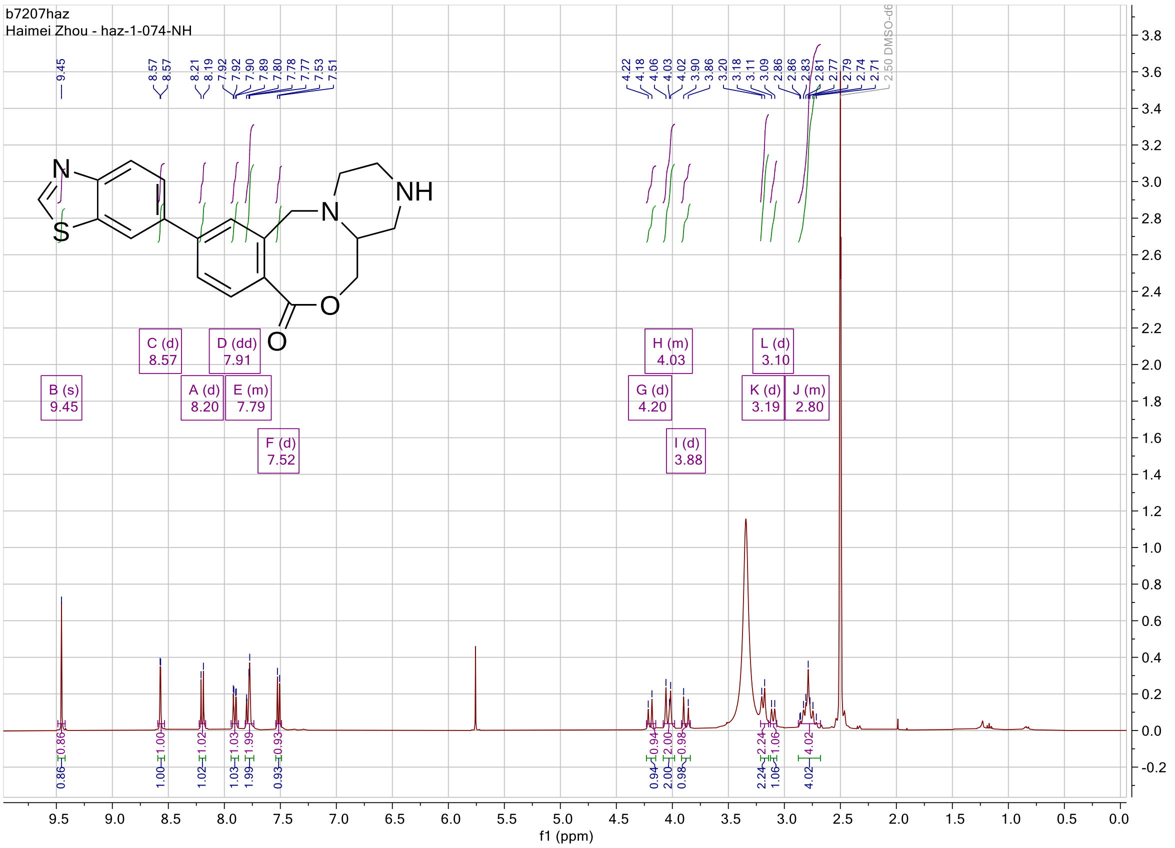

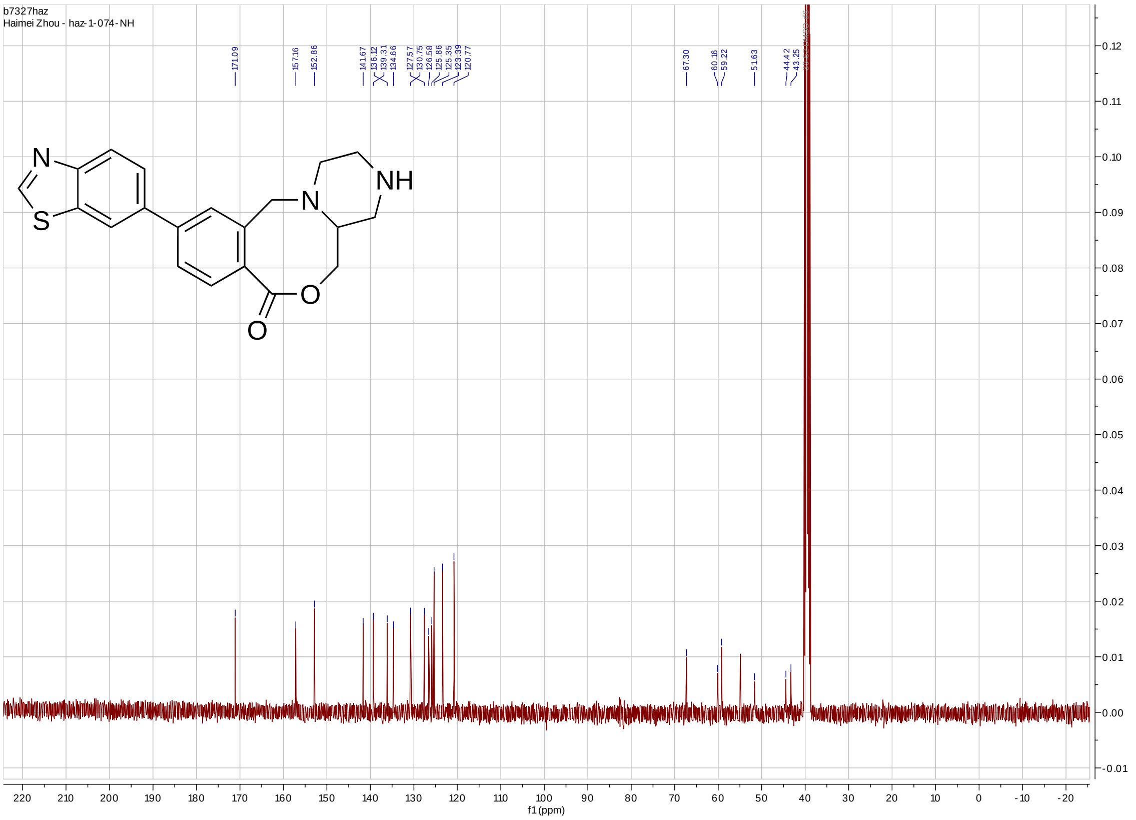

10-(Benzo[d]thiazol-6-yl)-3-(cyclopropylsulfonyl)-1,2,3,4,4a,5-hexahydrobenzo[f]pyrazino[2,1-c][1,4]oxazocin-7(12H)-one (6f) - δ_H (400 MHz) and $^{13}C\{^1H\}$ NMR (101 MHz)

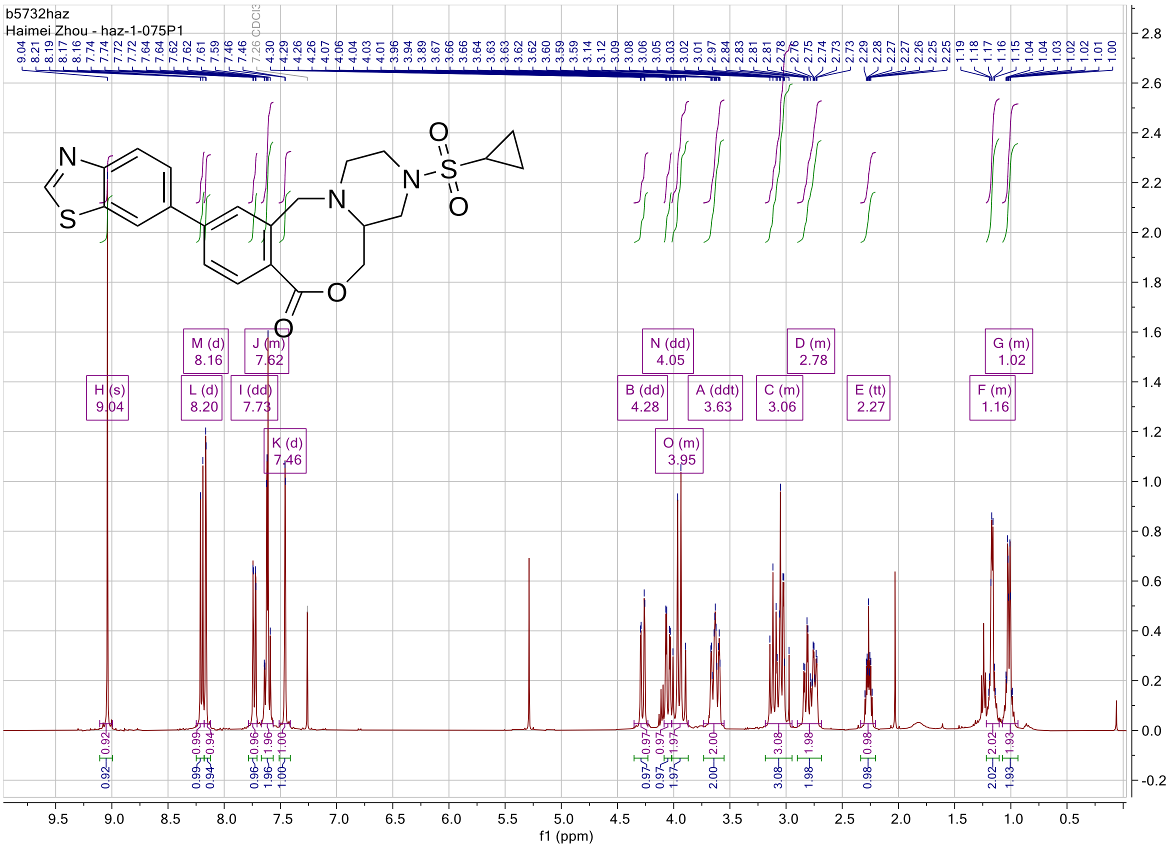

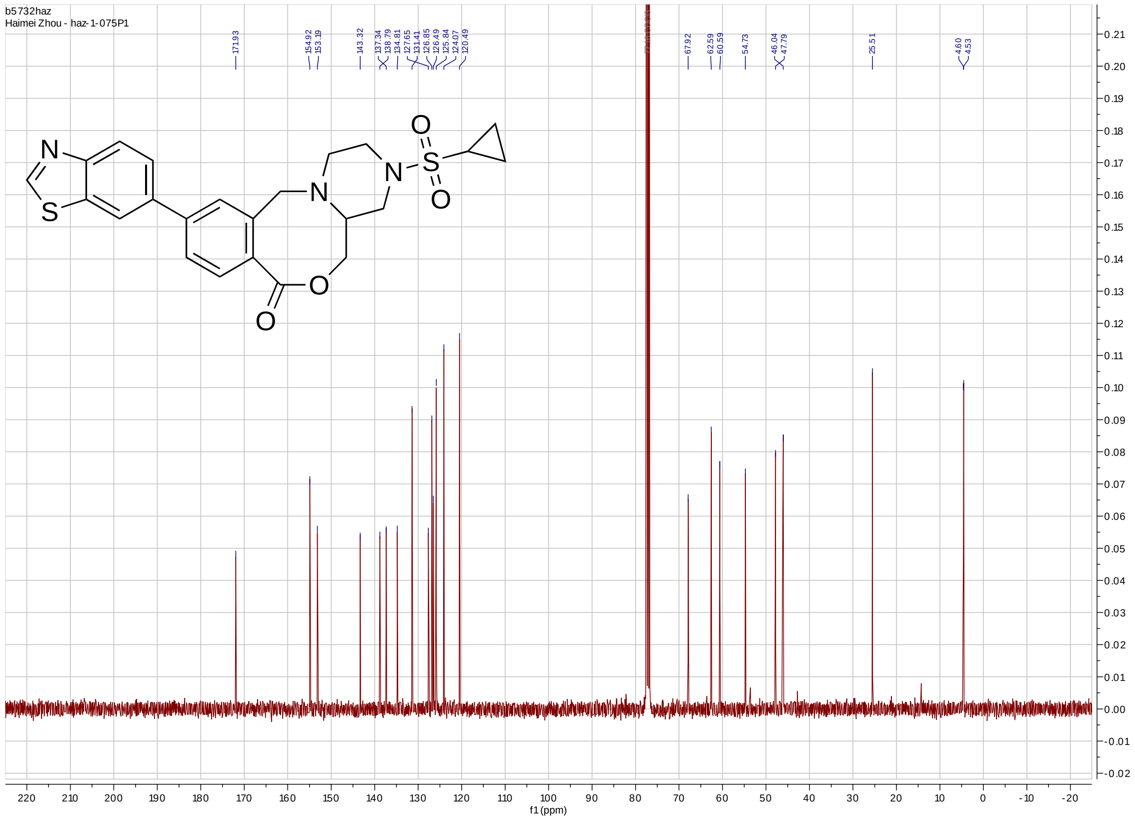

3-((1H-Pyrrolo[2,3-b]pyridin-3-yl)methyl)-10-(benzo[d]thiazol-6-yl)-1,2,3,4,4a,5-hexahydrobenzo[f]pyrazino[2,1-c][1,4]oxazocin-7(12H)-one (6g) - δ_H (400 MHz) and $^{13}C\{^1H\}$ NMR (101 MHz)

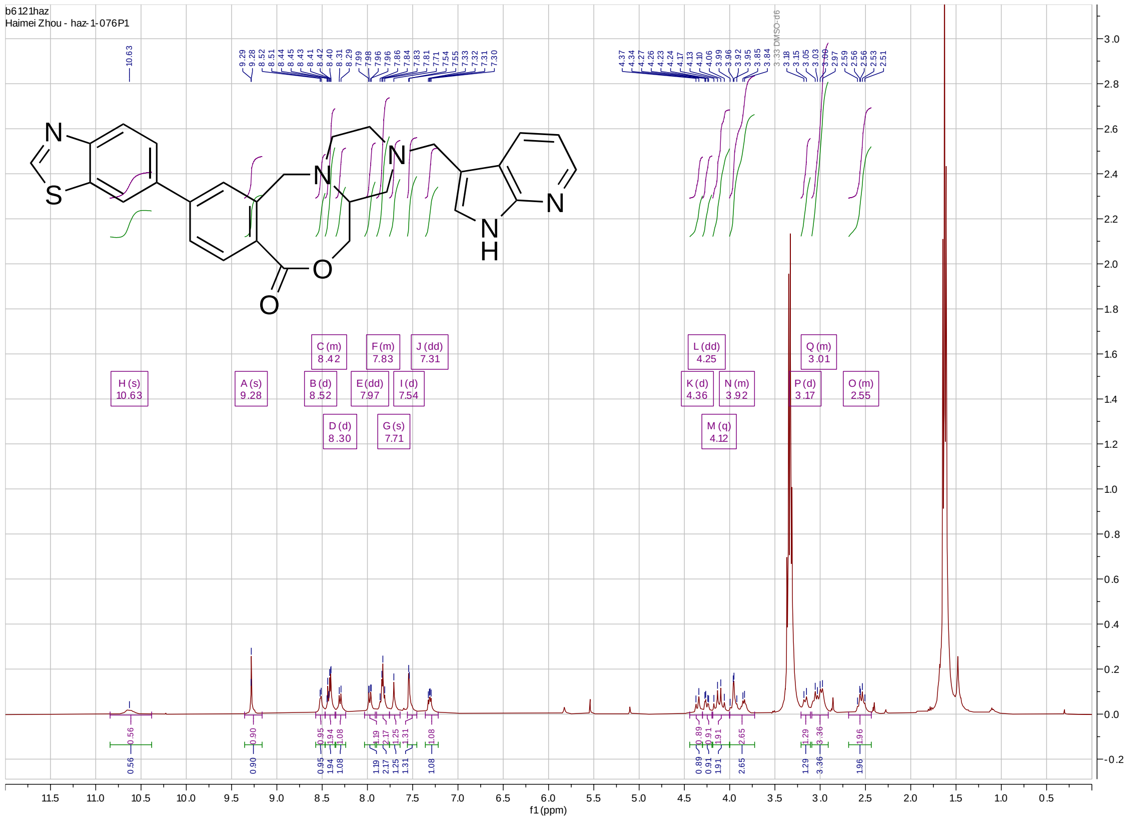

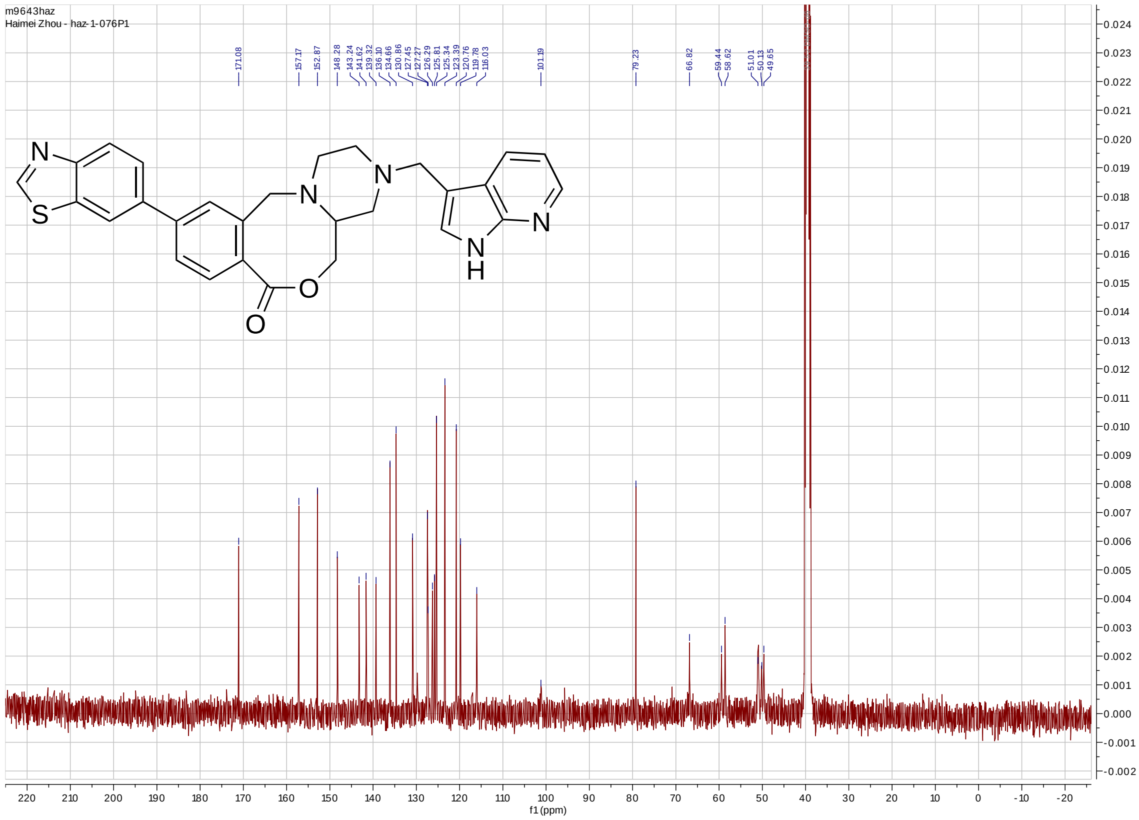

3-(2-(1H-Indol-3-yl)acetyl)-10-(benzo[d]thiazol-6-yl)-1,2,3,4,4a,5-hexahydrobenzo[f]pyrazino[2,1-c][1,4]oxazocin-7(12H)-one (6h) - δ_H (400 MHz) and $^{13}C\{^1H\}$ NMR (101 MHz)

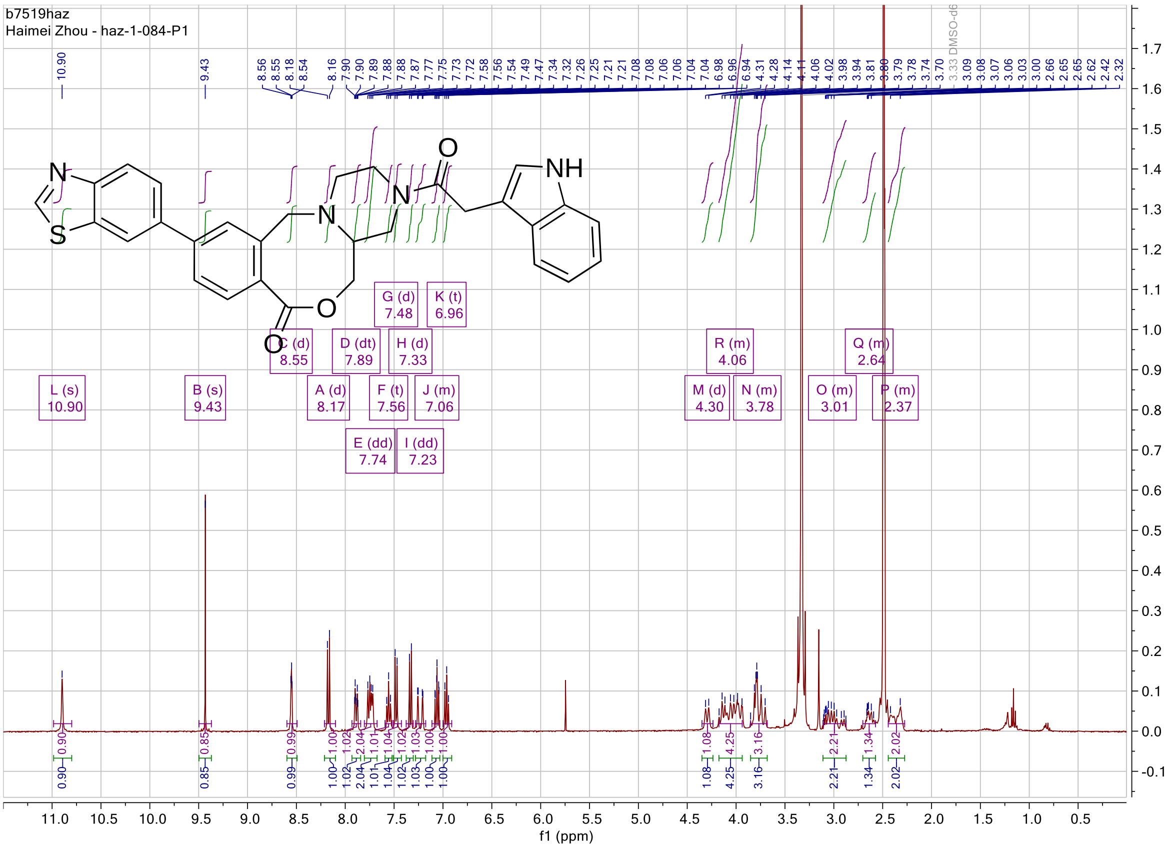

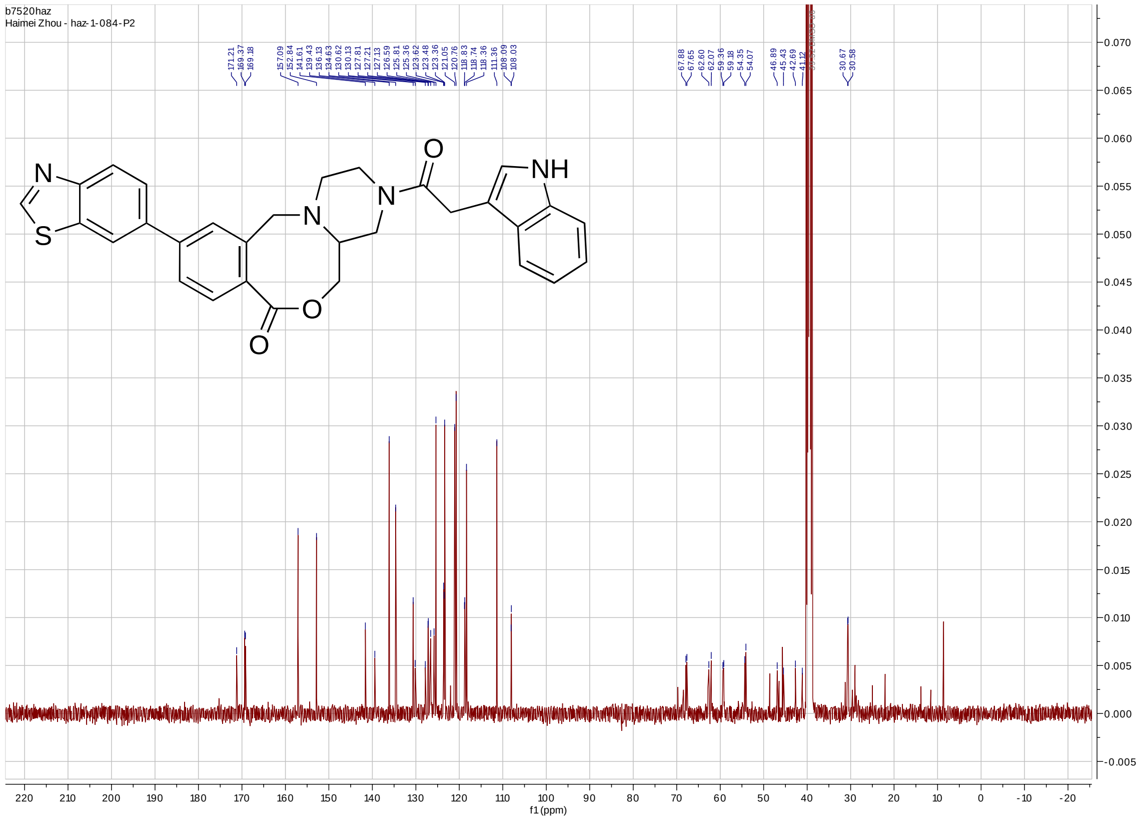

10-(Benzo[d]thiazol-6-yl)-3-(4-(trifluoromethyl)phenyl)-1,2,3,4,4a,5-hexahydrobenzo [f]pyrazino[2,1-c][1,4]oxazocin-7(12H)-one (6i) - δ_H (400 MHz) and $^{13}C\{^1H\}$ NMR (101 MHz)

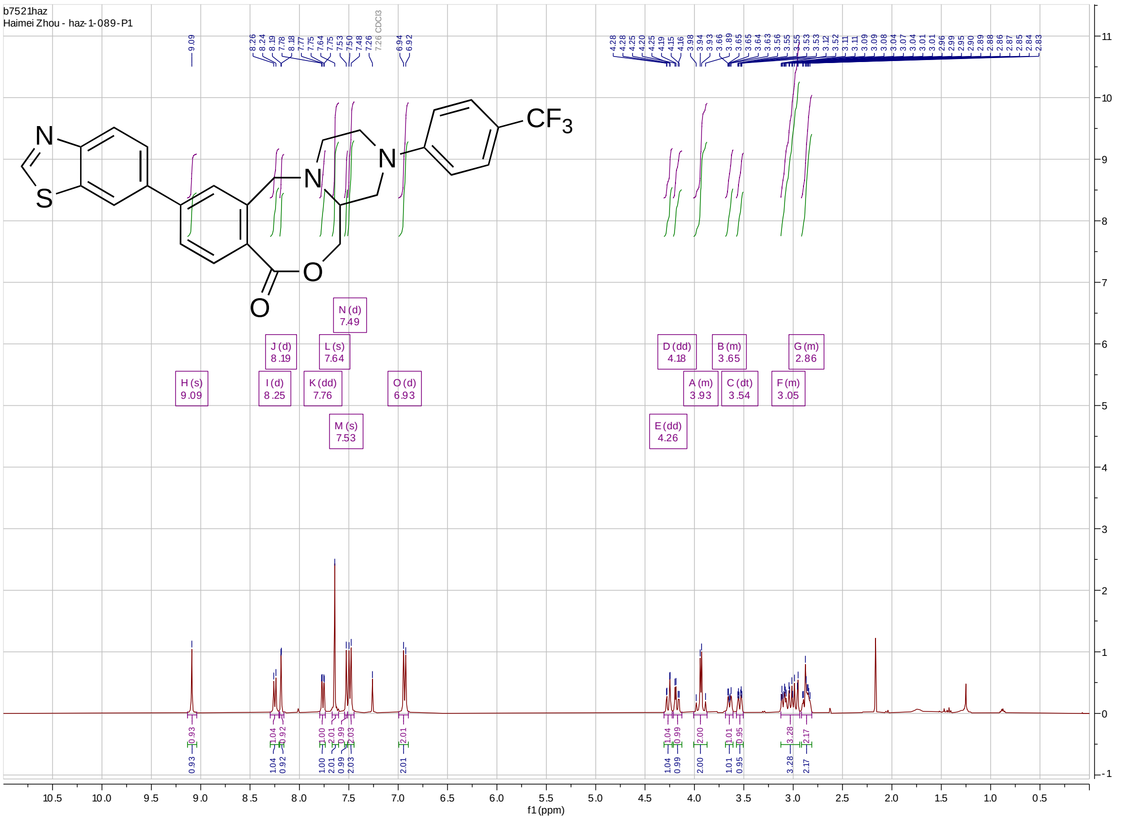

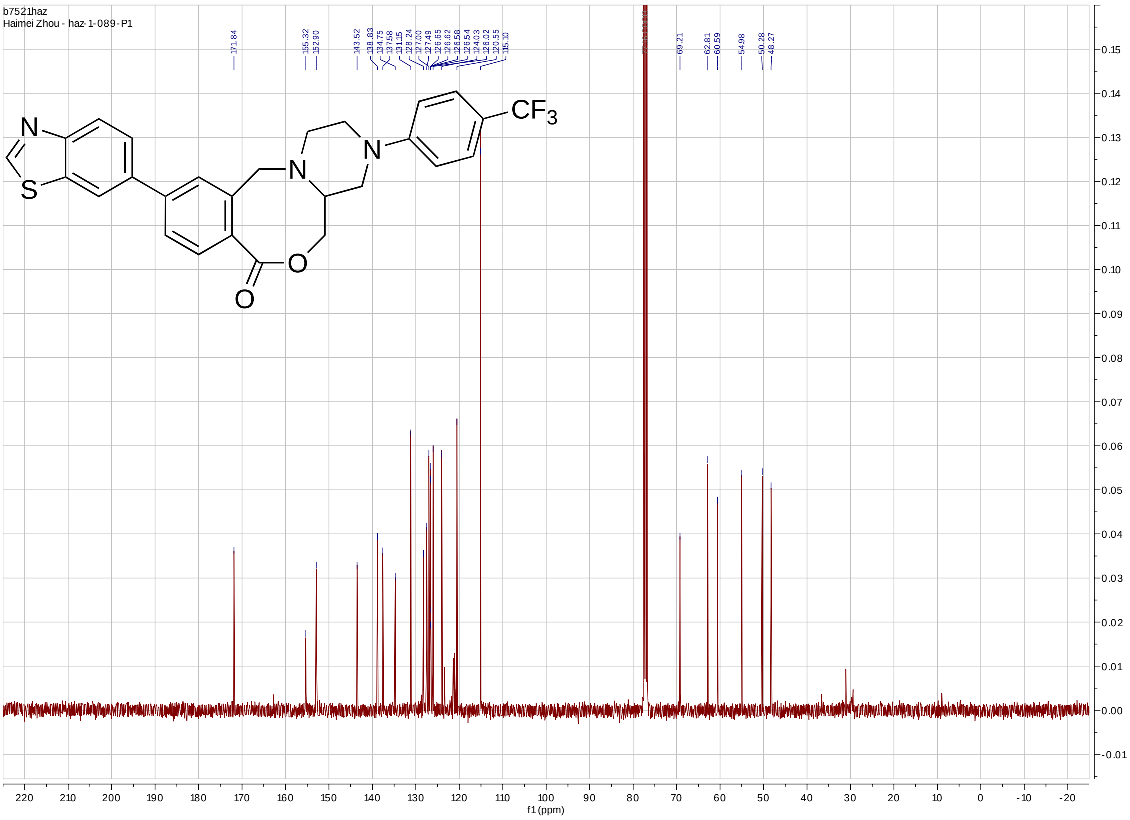

10-(benzo[d]thiazol-6-yl)-3-(5-nitropyridin-2-yl)-1,2,3,4,4a,5-hexahydrobenzo[f] pyrazino[2,1-c][1,4]oxazocin-7(12H)-one (6j) - δ_H (400 MHz) and $^{13}C\{^1H\}$ NMR (101 MHz)

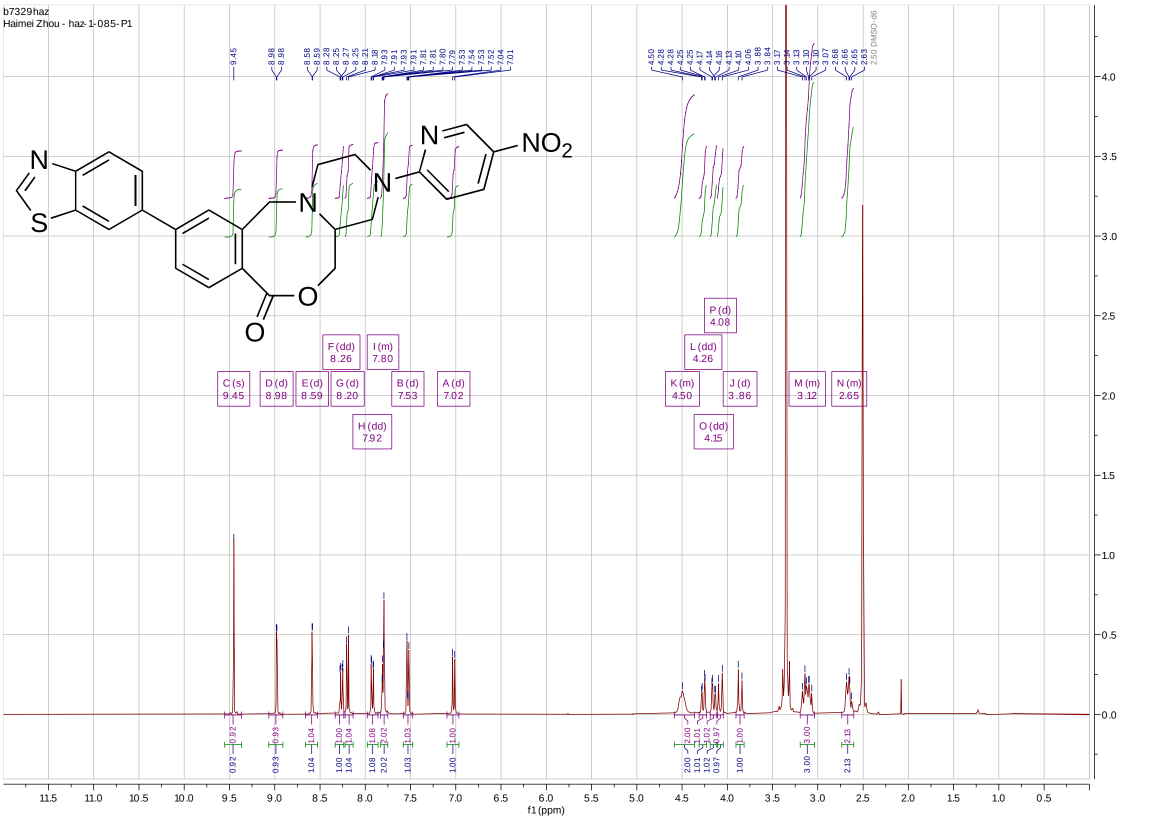

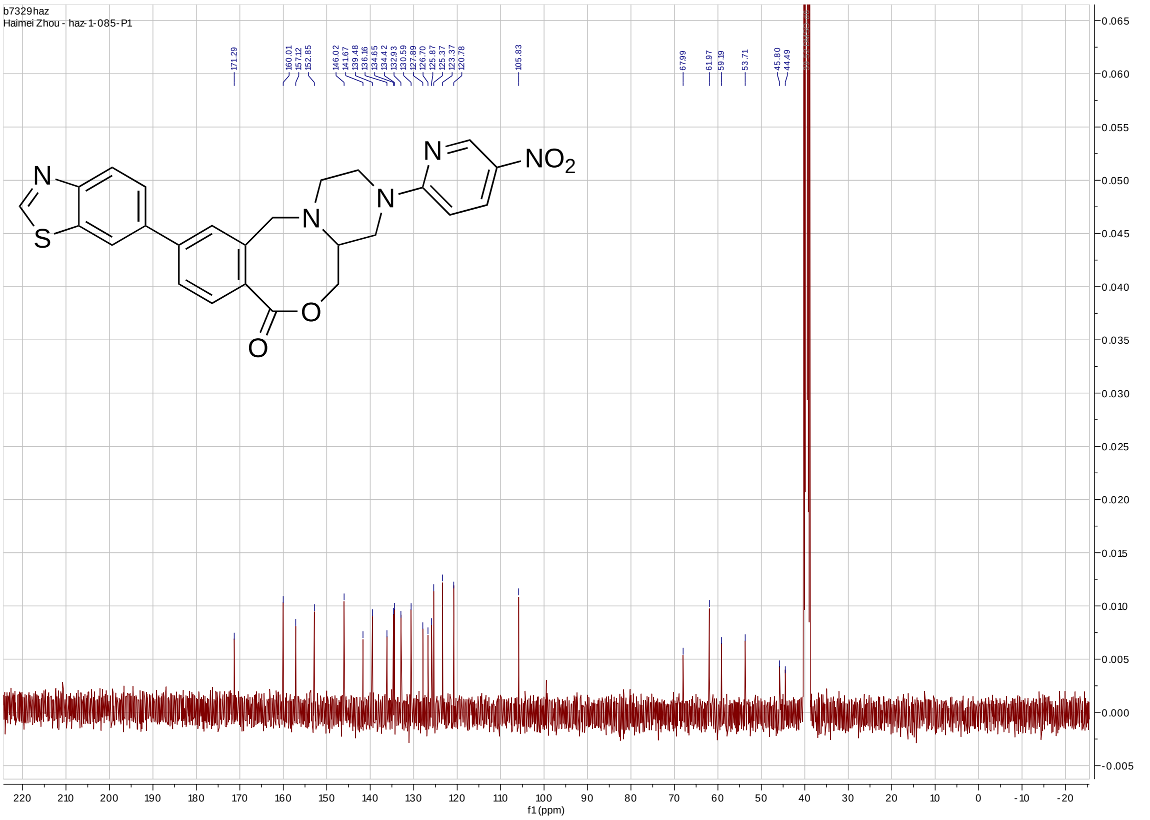

References

1. I. Zalessky, J. M. Wootton, J. K. F. Tam, D. E. Spurling, W. C. Glover-Humphreys, J. R. Donald, W. E. Orukotan, L. C. Duff, B. J. Knapper, A. C. Whitwood, T. F. N. Tanner, A. H. Miah, J. M. Lynam and W. P. Unsworth, *J. Am. Chem. Soc.*, **2024**, *146*, 5702–5711.

2. L. Hu, S. Shi, X. Song, F. Ma, O. Ji and B. Qi, *Eur. J. Med. Chem.*, **2024**, *265*, 116074.

3. L. Wang, R. Xiao, J. Song, L.-S. Zheng, Q. Lang, G.-Q. Chen and X. Zhang, *Chin. J. Chem*., **2024**, *42*, 43–47.

4. Y. Wang, Y. Liu, Y. Zhang, Z. Zhang, L. Xu, J. Wang, Y. Yang, B. Hu, Y. Yao, M. Wei, J. Wang, B. Tang, K. Zhang, S. Liu and G. Yang, *Eur. J. Med. Chem.*, **2024**, *271*, 116395.

5. B. Dupouy, M. Donzel, M. Roignant, S. Charital, R. Keumoe, Y. Yamaryo-Botté, A. Feckler, M. Bundschuh, Y. Bordat, M. Rottmann, P. Mäser, C. Y. Botté, S. A. Blandin, S. Besteiro and E. Davioud-Charvet, *ACS Infect. Dis.*, **2024**, *10*, 3553–3576.
